# Peritoneal ovarian cancer growth in the omentum proceeds independently of mature adipocytes

**DOI:** 10.1038/s41467-026-76471-x

**Published:** 2026-08-12

**Authors:** Rachel L. Mintz, Jichang Han, Emily G. Butka, Alexandre Gallerand, Shuai Gao, Ayoung Kim, Sarah Ning, Nicole K. H. Yiew, Mary Wohltmann, Galina Fedotova, Wei Zou, Nan Zhang, Samantha A. Morris, Bernd H. Zinselmeyer, Gwendalyn J. Randolph

**Affiliations:** 1 Department of Biomedical Engineering, Washington University, St. Louis, MO USA; 2 Department of Pathology and Immunology, Washington University, St. Louis, MO USA; 3 Vagelos Division of Biomedical and Biological Sciences, Washington University, St. Louis, MO USA; 4 Department of Genetics and Developmental Biology, Washington University, St. Louis, MO USA; 5 Immunology, Microenvironment and Metastasis Program, Ellen and Ronald Caplan Cancer Center, The Wistar Institute, Philadelphia, PA USA

**Keywords:** Ovarian cancer, Metastasis, Cancer microenvironment

## Abstract

The omentum, a specialized adipose tissue within the peritoneum, is a primary niche of ovarian cancer dissemination. Omental adipocytes are widely thought to promote tumor growth by supplying lipids, supported in part by studies using global FABP4 deficiency. Here, we directly test whether mature adipocytes are required for peritoneal ovarian cancer growth using mice congenitally lacking mature adipocytes within the peritoneal cavity, including the omentum. Across several ovarian cancer models (ID8p53^−/−^Brca2^−/−^, BPPNM, and KPCA), tumors preferentially seed adipose-associated regions despite the absence of mature adipocytes. Loss of mature adipocytes does not impair peritoneal tumor expansion, whereas removal of the adipocyte-free omentum significantly reduces tumor burden. Murine and human single-cell transcriptomic analyses reveal enrichment of lipid-handling gene expression, including FABP4, in omental endothelial cells at steady state and in tumor-bearing tissue. Endothelial-specific deletion of FABP4 reduces omental tumor expansion and limits tumor vascular complexity. These findings indicate that mature adipocytes are not required for omental ovarian cancer growth and highlight tumor-growth-favoring features of the omental niche endothelium.

## Abstract (web summary)

Ovarian cancer (OC) often spreads to the omentum, an adipose-rich tissue in the abdomen. Here, authors show that FABP4-expressing endothelial cells contribute to OC growth in the omentum independently of mature adipocytes.

## Introduction

At diagnosis, over 80% of ovarian cancer (OC) cases have metastasized to the peritoneal cavity, contributing to a five-year survival rate of only 25%^1^. The greater omentum, a highly vascularized, specialized visceral adipose tissue fold that arises off the greater curvature of the stomach, is an early and predominant site of transcoelomic metastasis in OC^2^. As cancer cells home to the omentum, tumor nodules eventually coalesce and progressively replace the underlying adipose tissue, resulting in profound omental caking. Structurally, the human omentum derives from a four-layered fold of peritoneum and comprises two recognizable regions with distinct cellular compositions: a vascularized, adipocyte-rich compartment populated by adipocytes and stromal cells, and a translucent peritoneal membrane composed primarily of mesothelial cells, fibroblasts, and macrophages^3,4^. As omental tumor burden increases, these compartments undergo remodeling, during which mature adipocytes are, to varying extents, replaced by fibroblasts, lymphocytes, and macrophages, thereby altering the underlying matrix structure^5^. This remodeling reflects a broader loss of homeostatic cell states and acquisition of cancer-associated transcriptional programs across multiple resident lineages^6^. Consequently, the omentum is removed during debulking surgeries as standard of care for advanced disease, and selective omentectomies are debated for earlier disease stages^7,8^. Furthermore, omentectomy reduces the growth of experimental OC in the peritoneal cavity of mice^9,10^. Still, it remains unknown why peritoneal metastases gravitate toward this specific omental niche.

Bolstered by the conclusions of a 2011 landmark study from the Lengyel group^11–13^, the notion that adipocytes drive OC peritoneal metastasis has prevailed^14,15^. In addition to secreting IL-8, IL-6, monocyte chemoattractant protein-1, and adiponectin, mature adipocytes can transfer lipids to OC tumor cells in vitro^11,16^. The concept that local lipid transfer provides metabolic support to tumors has been applied to other cancers^17–19^, particularly those in which mature white adipocytes are found near organs containing the primary tumor, such as breast, prostate, colon, and skin cancers. A key mechanistic observation supporting this model was that OC expansion in the mouse peritoneal cavity was markedly suppressed in mice lacking fatty acid-binding protein 4 (FABP4), also known as adipocyte protein 2, in all host cells^11^. Given that FABP4 accounts for 1–3% of soluble protein in mature adipocytes^20^, this finding in FABP4 knockout mice, in conjunction with images of tumor cell-adipocyte proximity in patient samples, fueled the concept that fatty acids from host adipocytes support tumor seeding and progression.

The human omentum is the largest visceral fat depot. Accordingly, the concept that omental adipocytes facilitate OC spread in patients has been discussed^21^. Relatedly, a possible link between obesity-induced metabolic syndrome and OC has been made^22,23^. Because it is difficult to assess adipocyte content in clinical practice directly, numerous translational studies have examined body mass index and other correlates of visceral adiposity. The use of these metrics is limited when OC patients present with ascites, tumor masses, or small bowel obstructions that can affect absolute body weight and mask underlying alterations in body composition^24^. While some studies have shown that obesity may influence chemotherapeutic potency^25–27^, most recent clinical data indicate that, ultimately, adiposity is not strongly associated with OC risk, prognosis, or survival^24,28–34^.

Notably, the deficiency of FABP4 in mice is not equivalent to the loss of mature adipocytes. Thus, whether mature adipocytes are themselves required for peritoneal OC progression remains unresolved. Here, we used a mouse model selectively lacking mature adipocytes within the peritoneal cavity to directly test their necessity for tumor seeding and outgrowth in the omentum following intraperitoneal injection. Across multiple models, loss of mature adipocytes did not impair tumor establishment or expansion in the omentum, nor did it alter therapeutic response. Single-cell transcriptomic analyses revealed that, among the non-adipocyte populations profiled, endothelial cells exhibited enriched FABP4 expression at steady state and in the tumor-bearing omentum. Endothelial-specific deletion of FABP4 reduced omental tumor expansion without disrupting baseline vascular architecture. Together, these findings indicate that mature adipocytes are not strictly required for omental OC growth and point to adipocyte-independent features of the omental niche.

## Results

### Adipose-rich regions within peritoneal organs are preferential sites of OC localization

After intraperitoneal (i.p.) injection of ID8p53^−/−^Brca2^−/−^ GFP Luc tumor cells, comprehensive biodistribution studies were conducted at 7 days (Suppl. Fig. 1a) and 6 weeks (Suppl. Fig. 1b) to identify anatomical sites of tumor localization. We compared tumor burden in the omentum with that in other organs, including adipose-rich peritoneal organs, such as the mesentery, perirenal adipose depot, and gonadal fat pads. Fat pads outside the peritoneal cavity, including axillary and inguinal depots, and organs in the thoracic cavity, including the heart and lungs, showed little to no signal, indicating confinement of tumor growth to the peritoneal cavity, as expected, given the route of tumor administration.

**Fig. 1:**
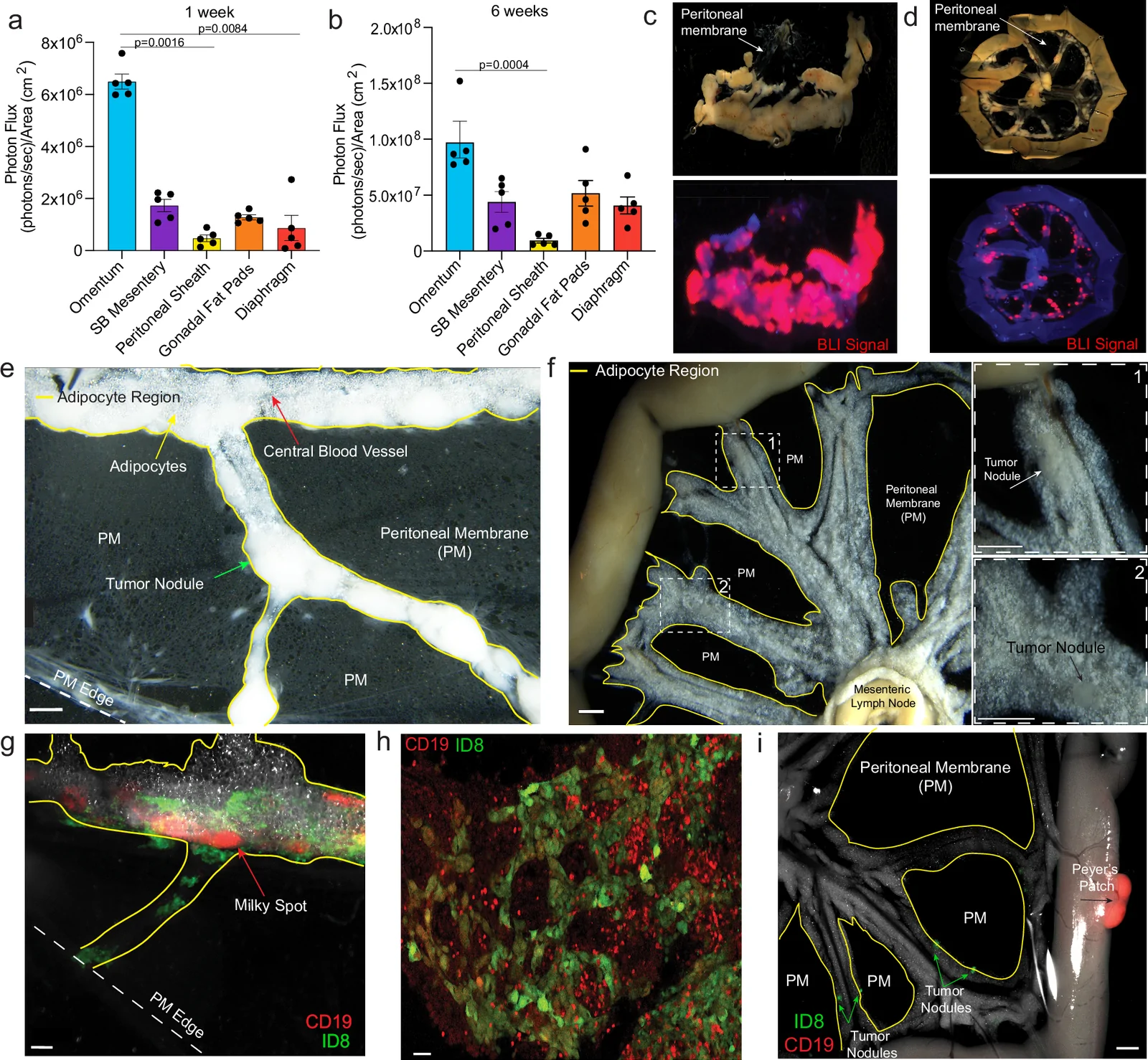
OC localizes to adipose-rich regions of peritoneal organs. Biodistribution of ID8p53^−/−^Brca2^−/−^ GFP Luc tumor burden in organs of C57BL/6 J wild-type (WT) mice. **a** One week (*n* = 5 mice Kruskal–Wallis H(4) = 17.98, *p* = 0.0012) and (**b**) Six weeks (*n* = 5 mice, Kruskal–Wallis H(4) = 17.49, *p* = 0.0016) post i.p. injection of tumor cells. Tumor burden was quantified as photon flux per organ area by ex vivo bioluminescence imaging (BLI). Kruskal–Wallis test, followed by Dunn’s multiple comparison test. Exact *p*-values for significant pairwise comparisons are shown in the panels. SB small bowel. Brightfield stereoscope image (top) and corresponding ChemiDoc MP Imaging System bioluminescence (BLI) image (bottom) of the (**c**). Omentum and (**d**) Small-bowel mesentery six weeks after tumor injection. The BLI signal corresponding to tumor burden is shown in red, and blue is used for contrast. **e** Representative stereoscope image of an omentum one week post tumor injection in a WT mouse. The adipocyte-rich region is outlined in yellow, and the surrounding peritoneal membrane (PM) is labeled. Scale bar, 1 mm. **f** Representative stereoscope image of a small bowel mesentery one week after tumor injection in a WT mouse. The adipocyte-rich region is outlined in yellow, and the surrounding peritoneal membrane (PM) is labeled. Scale bar, 1 mm. **g** Stereoscope (scale bar, 200 μm) and (**h**) 2-photon (scale bar, 40 μm) images of ID8p53^−/−^Brca2^−/−^ GFP Luc tumor cells within a milky spot of the adipocyte-rich region of the omentum in a CD19^Cre/+^Tomato (TD) mouse three days after i.p. tumor cell injection. GFP^+^ tumor cells (green) and CD19^+^ B cells (red). **i** Stereoscope image of tumor nodules in the small bowel mesentery of a CD19^Cre/+^TD mouse three days after i.p. tumor injection. CD19^+^ Peyer’s patch is indicated. PM, peritoneal membrane. Scale bar, 1 mm. Data are presented as mean ± SEM. Images in (**g**, **h**) are representative of three independent experiments with similar results. Throughout, n indicates the number of individual mice, each an independent biological replicate.

Across all time points, the omentum exhibited the highest tumor burden, with 2–3-fold greater signal than any other site, both early (Fig. 1a) and late (Fig. 1b) after implantation. The small bowel mesentery, peritoneal sheath, gonadal fat pads, and the diaphragm separating the peritoneal and thoracic cavities were also among the top five sites of tumor seeding. These peritoneal sites were therefore selected for analysis in subsequent experiments. Other peritoneal organs, such as the stomach, spleen, small intestines, colon, cecum, kidneys, adrenal gland, and liver, maintained low signal throughout the tumor course (Suppl. Fig. 1a, b). Six weeks after tumor implantation, tumor nodules persisted and expanded in regions of adiposity within the omentum and mesentery, as shown in the bright-field and corresponding ex vivo bioluminescence images (Fig. 1c, d). This localization pattern aligns with clinical findings in OC patients^2^.

Having established that tumor burden preferentially targeted peritoneal adipose tissue, we examined tumor seeding within these adipocyte-rich compartments in greater detail. The omentum is covered by a continuous mesothelial layer, and the underlying tissue can be anatomically partitioned into two regions: an adipose-rich, vascularized compartment and a translucent, peritoneal membrane largely devoid of adipocytes but composed of fibroblasts, mesothelial cells, and macrophages^35^. These distinct tissue types are macroscopically and histologically identifiable in both humans and mice^3^. In the present study, we leverage this anatomical framework to map tumor localization within the omentum and to examine how regional structures may influence metastatic seeding. We therefore mapped tumor distribution relative to these anatomically distinct regions.

After acute tumor seeding, these adipose and peritoneal membrane regions in the mouse omentum are demarcated in Fig. 1e. Similar variations between the adipocyte and nonadipocyte peritoneal membrane regions characterize the human omentum^3,36^. Like the omentum, the mesentery is also organized into regions enriched in adipose tissue or peritoneal membrane (Fig. 1f) with a similar composition of fibroblasts, macrophages, and mesothelial lining cells^10,37^. Both the omentum and small bowel (SB) mesentery contain adipose-resident lymphoid aggregates, collectively termed fat-associated lymphoid clusters. In the omentum, these structures are commonly referred to as the milky spots^38,39^ and expand in response to an inflammatory challenge^40^. Thus, given their structural similarities, mutual location in the peritoneal cavity, and susceptibility to tumor seeding, the mesentery served as a comparator for the omentum to explore preferential omental tumor homing within the peritoneal cavity.

Within days of i.p. injection of ID8p53^−/−^Brca2^−/−^ GFP Luc tumor cells in CD19^Cre/+^Tomato (TD) mice, which were used to visualize B-cell–rich omental milky spots as structural landmarks, stereomicroscopy (Fig. 1g) or intravital two-photon imaging (Fig. 1h) demonstrated that the GFP^+^ ID8 tumor cells had already migrated to the adipocyte region of the omentum. CD19^+^ B cells delineated lymphoid-rich milky spots (Fig. 1g), revealing tumor lattices over adipocytes (Fig. 1h). Tumor cells were also detected within adipocyte-rich regions of the mesentery, frequently adjacent to branching vasculature and at a distance from CD19⁺ Peyer’s patches (Fig. 1i). Although adipose-rich regions of both the omentum and mesentery contained tumor cells, overall tumor burden was greater in the omentum than in the mesentery (Fig. 1a–i). Together, these data demonstrate preferential localization of ovarian tumor cells to adipose-rich regions within peritoneal organs.

### Mature adipocytes are not required for OC seeding and growth within the peritoneal cavity

To formally test the role of mature adipocytes in tumor seeding and expansion, we crossed mice expressing Cre driven by the adiponectin promoter with mice bearing lox-stop-lox-Rosa26 diphtheria toxin A (DTA), resulting in systemic constitutive ablation of mature white and brown adipocytes, with adipocytes retained only in the bone marrow, as previously described in ref. ^41^. Adiponectin expression is initiated only during late, terminal adipocyte differentiation and is absent in pre-adipocytes or early adipogenic progenitors^42–44^. Accordingly, Adiponectin Cre selectively targets fully differentiated adipocytes and does not drive recombination in adipocyte precursor populations. In the Adiponectin^Cre/+^ RosaDTA^f/+^ mice, any cell that activates the adiponectin promoter is constitutively eliminated upon terminal differentiation, preventing the accumulation or persistence of mature adipocytes throughout development and adulthood. While adipocyte progenitors are present, they lack the defining metabolic and endocrine functions of mature adipocytes, such as lipid storage, fatty acid mobilization, and adipokine secretion, which are acquired only upon terminal differentiation and are unlikely to recapitulate mature adipocyte functions in this model. Littermate Adiponectin^+/+^ RosaDTA^f/+^ mice, which retain normal adipose tissue development, were used as controls.

Bright-field imaging of the gonadal fat pad, inguinal fat pad, mesentery, and omentum revealed that, despite the absence of mature adipocytes, lipoatrophic mice retain anatomically recognizable tissue in canonical adipose depot locations. However, these structures are markedly reduced in size and substantially less conspicuous compared to littermate controls (Suppl. Fig. 2a). While mature adipocytes are absent in this model, the remaining depot-associated tissue is composed of mesothelial cells, fibroblasts, endothelial cells, macrophages, stem cells, and pre-adipocytes that collectively define these regions^45^.

Lipoatrophy is associated with metabolic complications, including hepatic steatosis, dysregulated glucose tolerance, and elevated circulating triglyceride levels, due to a lack of fat depots to store excess triglycerides^41,46–48^. To normalize these metabolic parameters while retaining a complete loss of mature adipocytes in the omentum and other tissues within the peritoneal cavity, we performed subcutaneous fat pad transplants into the scruff of the lipoatrophic mice, leaving the peritoneal cavity devoid of mature adipocytes and supplying an adipocyte-rich tissue to the mice systemically. Sham surgeries were also performed on littermate control mice, which retained their standard fat depots in the peritoneal cavity. Following fat transplant rescue surgery, we refer to Adiponectin^Cre/+^DTA^fl/+^ mice as **d**istal **a**dipocyte**-r**eceiving **l**ipoatrophic mice (DARL) and the sham surgery-receiving littermate control mice as **p**eritoneal **a**dipocyte-**r**eplete **c**ontrol mice (PARC).

When recovered weeks later, the engrafted fat in the scruff resembled healthy adipose tissue on H&E staining (Suppl. Fig. 2b) and expanded to more than double the starting weight at the time of transplant (Suppl. Fig. 2c). The mice tolerated the surgery well, as there was no significant reduction in body weight after surgery (Suppl. Fig. 2d). Serum triglycerides normalized after surgery and became statistically equivalent to that of fat-bearing control littermates (Suppl. Fig. 2e). Total serum cholesterol was elevated in lipoatrophic mice. Still, it remained below hypercholesterolemic ranges and was decreased toward control levels following fat transplantation (Suppl. Fig. 2f). Lipoatrophic mice initially had elevated fasting glucose levels compared to littermate controls, which became more pronounced with increasing duration (Suppl. Fig. 2g, h). The generation of DARL mice reduced fasting glucose levels so that they resembled PARC mice to a greater extent (Suppl. Fig. 2i, j). As previously reported, lipoatrophic mice had dysregulated responses to insulin and glucose^41^. These aberrations were largely corrected after surgery, as indicated by insulin and glucose tolerance tests (Suppl. Fig. 2i–l).

**Fig. 2:**
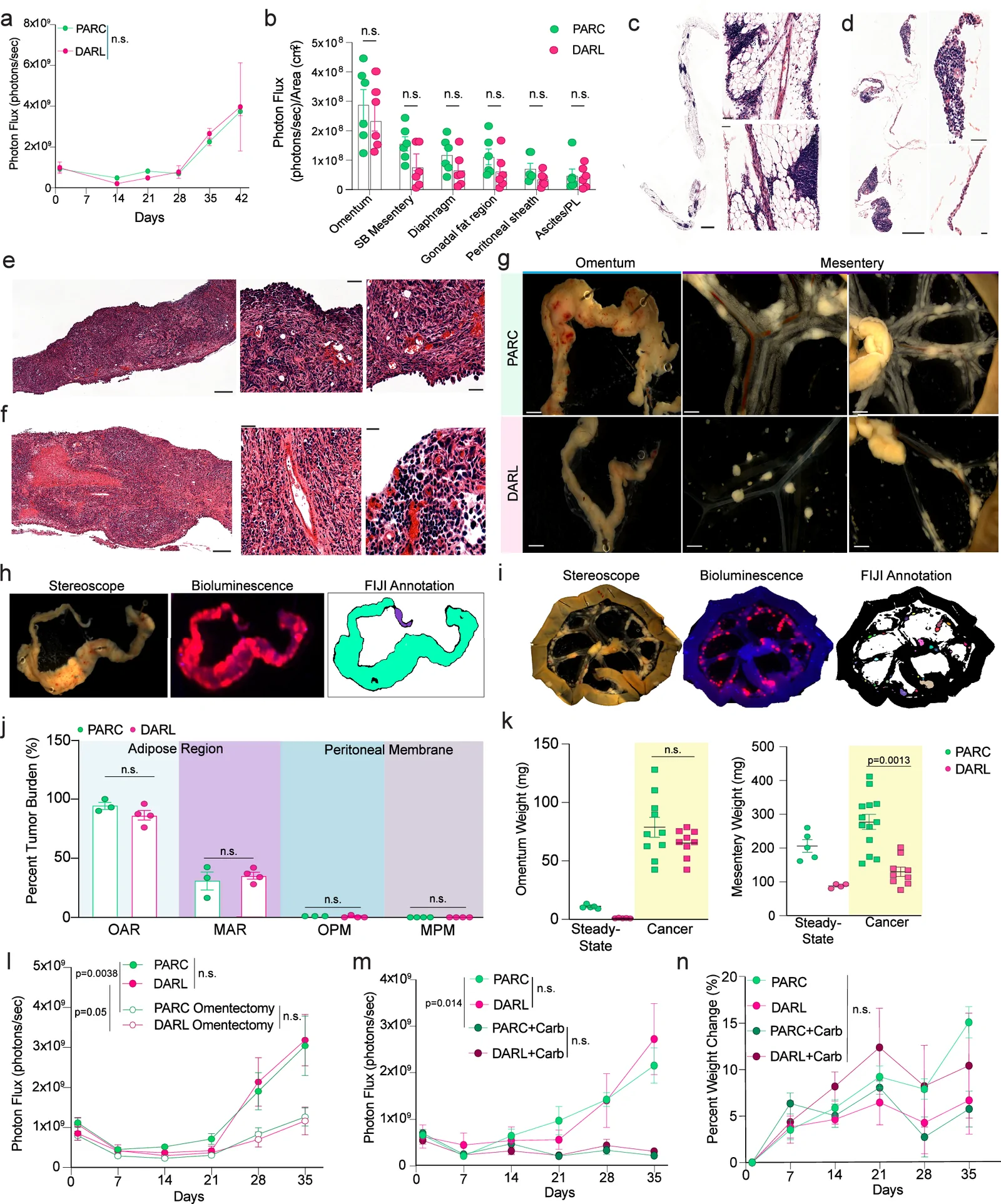
Omental adipocytes are not required for OC expansion in the peritoneal cavity. All experiments were performed following intraperitoneal injection of 5 × 10^6^ ID8p53−/−Brca2−/− GFP Luc tumor cells unless otherwise indicated. **a** In vivo peritoneal tumor burden (bioluminescence imaging, BLI) in PARC (*n* = 4) and DARL (*n* = 4) mice; area under the curve (AUC) of total photon flux compared by two-tailed Mann–Whitney test, n.s. **b** Tumor burden across peritoneal organs (ex vivo photon flux per organ area) in PARC (*n* = 6) and DARL (*n* = 6) mice at six weeks; per-organ two-tailed Mann–Whitney tests with Holm–Šídák correction, n.s. SB small bowel, PL peritoneal lavage. **c**, **d** H&E of steady-state omentum from PARC (**c**) and DARL (**d**) mice (full thickness). **e**, **f** H&E of tumor-bearing omentum from PARC (**e**) and DARL (**f**) mice at six weeks; insets show tumor infiltration perpendicular to the peritoneal surface. Scale bars: (**c**) 1 mm and 0.05 mm (insets); (**d**) 0.2 mm, 0.05 mm and 0.02 mm (insets); e and f, 0.2 mm and 0.05 mm (insets). **g** Stereoscope images of omentum (left) and SB mesentery (right) at six weeks; scale bars, 2 mm. **h**, **i** Representative stereoscope images, bioluminescence, and FIJI particle-analysis masks quantifying tumor nodules in omentum (**h**) and SB mesentery (**i**); each nodule is a distinct colored mask. **j** Percent tumor area per compartment (FIJI particle analysis): omentum adipose region (OAR), mesentery adipose region (MAR), omentum peritoneal membrane (OPM), mesenteric peritoneal membrane (MPM); PARC (*n* = 4) versus DARL (*n* = 4), per-compartment two-tailed Mann–Whitney tests with Holm–Šídák correction. **k** Omentum and SB mesentery weights from age-matched PARC and DARL mice at steady state or six weeks after tumor (cancer); Kruskal–Wallis (omentum *p* = 0.0002; mesentery *p* = 0.0001) with Dunn’s test (exact *p*-values shown in the panel). **l** In vivo tumor burden (BLI) after omentectomy (PARC *n* = 8, DARL *n* = 8) or sham surgery (PARC *n* = 7, DARL *n* = 6); AUC by Kruskal–Wallis (*p* = 0.0016) with Dunn’s test (exact *p*-values shown in the panel). **m** In vivo tumor burden (BLI) with carboplatin (30 mg/kg i.p. weekly; PARC *n* = 5, DARL *n* = 5) or saline (PARC *n* = 5, DARL *n* = 4); AUC by Kruskal–Wallis (*p* = 0.017) with Dunn’s test (exact *p*-values shown in the panel). **n** Percent body weight change with carboplatin; AUC by Kruskal–Wallis with Dunn’s test, n.s. Data are mean ± SEM, and all tests are two-sided. Micrographs in (**c**–**f**) are representative of > 50 mice across at least five independent experiments with similar results. Throughout, n indicates the number of individual mice, each an independent biological replicate.

After confirming normalization of metabolic parameters six weeks after fat transplant, PARC and DARL mice were injected i.p. with ID8p53^−/−^Brca2^−/−^ GFP Luc tumor cells. Unexpectedly, weekly bioluminescence imaging (BLI) revealed comparable tumor burden over time (Fig. 2a), with the endpoint chosen to avoid the onset of profound ascites. Ex vivo photon flux quantification demonstrated similar regional tumor distributions across peritoneal organs in PARC and DARL mice (Fig. 2b). This pattern mirrored that observed in unmanipulated C57BL/6 J mice (Fig. 1a, b), with the omentum exhibiting the highest tumor burden, followed by the mesentery. Thus, preferential tumor omental localization and expansion were preserved even in the absence of mature adipocytes.

We also evaluated other OC models. We i.p. injected PARC and DARL mice with 1×10^6^ KPCA (*Trp53*^−/−R172H^*Ccne1*^OE^*Akt2*^OE^*KRAS*^G12V^)-Luc cells or 3 × 10^6^ BPPNM (*Trp53*^−/−*R172H*^*Brca1*^−/−^*Pten*^−/−^*Nf1*^−/−^*Myc*^OE^) - Luc cells (Suppl. Fig. 3), both derived from fallopian epithelial cells^49^. Representative endpoint images and tumor burden quantification for KPCA- and BPPNM-injected PARC and DARL mice (Suppl. Fig. 3) showed that the omentum becomes encapsulated by tumor nodules in both genotypes, regardless of the presence of mature adipocytes (Suppl. Fig. 3a–d). The KPCA tumors quickly saturated the bioluminescence readout and were therefore harvested on day 7, by which point it was already clear that the omentum was a potent reservoir for tumor growth, regardless of the presence of mature adipocytes (Suppl. Fig. 3a, b). While the KPCA model exhibits particularly rapid growth kinetics that limit temporal resolution of early modulatory events, concordant results across slower-growing models support the conclusion that mature adipocytes are not required for omental tumor establishment or expansion. BPPNM tumors, appearing to grow less aggressively than the KPCA model, were harvested at day 21, where again, the omentum was the dominant site of tumor residence regardless of the presence of mature adipocytes (Suppl. Fig. 3c, d). These findings, consistent with results obtained using ID8p53^−/−^Brca2^−/−^ GFP Luc tumor cells (Fig. 2a, b), indicate that OC cells expand efficiently and robustly within the omentum in the absence of mature adipocytes, irrespective of tumor cell-of-origin (fallopian tube vs ovarian surface epithelium), genetic mutations, or the number of tumor cells injected.

Histological evaluation of the omentum at steady state revealed marked differences between PARC and DARL mice. In PARC mice, the omentum contained abundant mature adipocytes interspersed with milky spots and an extensive vascular network (Fig. 2c). In contrast, the omentum from DARL mice was approximately fivefold smaller and lacked mature adipocytes yet retained clearly demarcated milky spots connected by stromal elements and blood vessels (Fig. 2d). Following six weeks of ID8p53^−/−^Brca2^−/−^ GFP Luc tumor growth, the omenta of PARC and DARL mice exhibited a convergent histological appearance characterized by dense fibrosis and prominent vascularization, despite their distinct baseline architectures (Fig. 2e, f). Notably, mature adipocytes were no longer discernible within tumor-bearing omentum at endpoint, consistent with the displacement and remodeling of the adipose compartment during progression. Despite this dense fibrotic remodeling, blood vessels remained readily identifiable in both PARC and DARL omenta.

Macroscopic examination after pinning out the omentum and mesentery further highlighted the convergence in tumor localization between PARC and DARL mice. In both genotypes, tumor lesions preferentially localized to regions corresponding to the former adipose compartment of the omentum, despite the absence of mature adipocytes in DARL mice (Fig. 2g). Although tumor lesions were also evident along adipose-associated regions of the mesenteric branches, the overall tumor burden was consistently greater in the omentum than in the mesentery, consistent with quantitative bioluminescence measurements (Fig. 1a, b). Together, these observations reinforce the omentum as the dominant site of peritoneal tumor growth, even in the absence of mature adipocytes.

Tumor nodules within the adipose-associated regions and peritoneal membrane of the omentum and mesentery were quantified using particle analysis in FIJI/ImageJ, allowing measurement of both nodule number and size distribution (Fig. 2h, i). In the PARC mice with retained mature adipocytes, the omental adipose region was extensively replaced by tumor nodules, characterized by a small number of large tumor nodules encompassing the structure (Fig. 2h). In contrast, the mesenteric adipose region contained a greater number of smaller nodules (Fig. 2i). Nearly 100% of the omental adipose-associated region was occupied by tumor, even in the DARL mice lacking mature adipocytes (Fig. 2j). In contrast, less than 5% of the omentum peritoneal membrane was colonized (Fig. 2j). Similarly, approximately 30% of the mesenteric adipose-associated region was occupied by tumors in both genotypes, while the mesenteric peritoneal membrane was largely spared (Fig. 2j). Thus, although mature, lipid-laden adipocytes are not required for tumor establishment, adipose-associated regions of the omentum and mesentery remain the predominant sites of tumor accumulation at endpoint. Notably, relative sparing of the peritoneal membrane at endpoint does not preclude an early functional role for mesothelial cells but is consistent with prior work showing that tumor cells engage and remodel the mesothelial surface before invading deeper stromal compartments^35,50^.

To further assess the impact of adipocyte loss on tissue permissiveness, we compared depot weights at steady state and after tumor growth. At baseline, the absence of mature adipocytes resulted in a more than tenfold reduction in omental weight and a greater than twofold reduction in mesenteric weight in DARL mice relative to age-matched PARC controls (Fig. 2k), consistent with histological differences observed at steady state (Fig. 2c, d; Suppl. Fig. 2a). Despite this substantial reduction in starting tissue mass, tumor growth resulted in near-equivalent omental weights between DARL and PARC mice at endpoint, indicating comparable dominance of the omentum by tumor tissue (Fig. 2k, left). In contrast, mesenteric weights in DARL mice remained significantly lower than those in PARC mice following tumor growth (Fig. 2k, right). Collectively, these findings demonstrate that the space normally occupied by lipid-laden adipocytes is highly permissive to tumor expansion. However, this permissiveness persists even in the absence of mature adipocytes, indicating that mature adipocytes themselves are not required to sustain tumor growth within these anatomical niches.

We next wondered whether the omentum itself remained critical for robust tumor expansion in the absence of mature adipocytes. After recovery from sham or subcutaneous fat transplant surgeries, PARC and DARL mice underwent omentectomy or sham peritoneal surgery followed by i.p. injection of ID8p53^−/−^Brca2^−/−^ GFP Luc tumor cells. Omentectomized DARL and PARC mice had reduced tumor burdens compared with mice that underwent sham peritoneal surgery, indicating that the omentum remains a key determinant of peritoneal tumor burden when it lacks mature adipocytes (Fig. 2l).

To consider the further possibility that adipocytes may impact therapeutic efficacy or tolerance, even if they are not required for tumor expansion, we treated PARC and DARL mice receiving ID8p53^−/−^Brca2^−/−^ GFP Luc tumor cells with carboplatin, a chemotherapeutic agent that is part of the first-line standard of care for HGSOC^51^. Carboplatin treatment resulted in a comparable decrease in tumor burden in PARC and DARL mice (Fig. 2m). In addition, chemotherapy-induced changes in body weight did not differ between genotypes (Fig. 2n). Therefore, the absence of mature adipocytes does not alter chemotherapeutic efficacy or toxicity.

In conclusion, the absence of mature adipocytes did not detectably alter tumor growth in the peritoneal cavity, regardless of whether omentectomy or chemotherapy was performed. Yet, the area where adipocytes usually reside remained the most conducive to tumor seeding and expansion. Even without mature adipocytes, the omentum remained a key driver of tumor expansion in the peritoneal cavity.

### Natural killer cell and lymphocyte deficiency do not impact omentum-centric OC growth in the peritoneal cavity

**Fig. 3:**
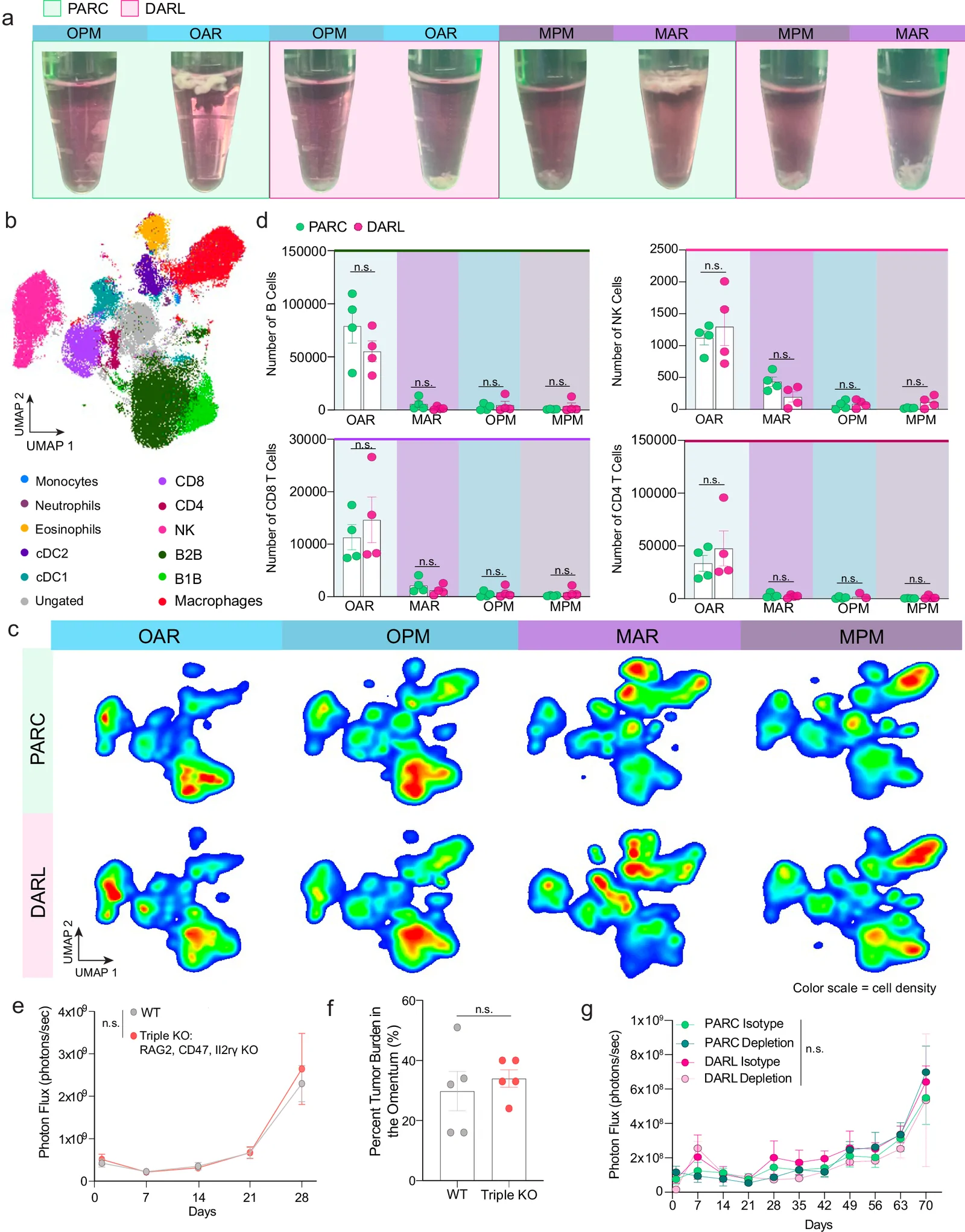
Immune composition of the omentum and mesentery is preserved after adipocyte depletion. **a** Representative image showing the separation of adipose and peritoneal membrane compartments of the omentum and mesentery of PARC and DARL mice to generate four distinct tissue compartments: omentum adipose region (OAR), omentum peritoneal membrane (OPM), mesenteric adipose region (MAR), and mesenteric peritoneal membrane (MPM). **b** UMAP dimensional reduction of CD45^+^ immune cells isolated from PARC (*n* = 4) and DARL (*n* = 4) mice across all tissue samples (OAR, OPM, MAR, MPM) revealed 12 immune cell clusters based on spectral flow cytometry. **c** Density projections of CD45^+^ immune cells from PARC (*n* = 4) and DARL (*n* = 4) mice overlaid on the reference UMAP shown in panel b, illustrating the distribution of immune populations across OAR, OPM, MAR, and MPM compartments. **d** Quantification of NK cells, CD8^+^ T cells, CD4^+^ T cells, and B cells in PARC (*n* = 4) and DARL (*n* = 4) mice across the OAR, OPM, MAR, and MPM. Pairwise comparisons between genotypes were performed for each tissue using two-tailed Mann–Whitney tests with Holm–Šídák correction for multiple comparisons, n.s. **e** Quantification of in vivo peritoneal tumor burden by bioluminescence imaging (BLI) in age-matched WT (*n* = 5) and Triple KO mice lacking RAG2, CD47, and IL-2Rγ (*n* = 5) after i.p. injection of 5×10^6^ ID8p53^−/−^Brca2^−/−^ GFP Luc tumor cells. Area under the curve (AUC) of total photon flux over time was calculated per mouse and compared using a two-tailed Mann–Whitney test, n.s. **f** Percent tumor burden in the omentum of WT (*n* = 5) and Triple KO (*n* = 5) mice quantified as photon flux per organ area from ex vivo BLI four weeks after tumor injection. Two-tailed Mann–Whitney test, n.s. **g** Quantification of in vivo tumor burden by BLI in PARC and DARL mice treated weekly with CD8, CD4, and NK1.1 depleting antibodies (PARC depletion, *n* = 5; DARL depletion, *n* = 3) or isotype controls (PARC Isotype, *n* = 5; DARL isotype, *n* = 5) starting one week before tumor injection and continuing weekly thereafter. Kruskal–Wallis test, n.s. Data are presented as mean ± SEM. Throughout, n indicates the number of individual mice, each an independent biological replicate.

The omentum’s unique lymphoid aggregates, known as milky spots, are implicated in adaptive immunity and have been proposed to influence tumor growth by affecting regulatory T cells^50^. We wondered whether the absence of mature adipocytes affected the overall immune cell composition of the omentum or mesentery. Approximately ten weeks after subcutaneous fat transplant surgery, the omentum and mesentery from eight DARL and PARC mice were collected. The tissues’ peritoneal membrane and adipose regions (Fig. 3a) were carefully separated before pooling tissue regions for spectral flow cytometry, generating four unique sample types: (i) omental adipose region (OAR), (ii) mesenteric adipose region (MAR), (iii) omental peritoneal membrane (OPM), and (iv) mesenteric peritoneal membrane (MPM). Samples containing mature adipocytes were readily identified because mature adipocytes floated (Fig. 3a), further confirming that the peritoneal cavity tissues from DARL mice lacked mature adipocytes.

Manual flow cytometry gating (Suppl. Fig. 4a, b) was used to identify CD45^+^ immune populations overlaid on a dimensionality reduction plot, identifying 12 clusters (Fig. 3b). Shifts in these clusters were visualized in OAR, OPM, MAR, and MPM dimensionality reduction plots (Fig. 3c). Among the CD45^+^ cells, natural killer (NK), CD4^+^, and CD8^+^ T cells showed modest increases. In contrast, B1b cells were slightly reduced in the omentum adipose region of DARL compared with PARC mice. Quantifications of these four cell types are shown in Fig. 3d. In contrast, the composition of the omentum peritoneal membrane was similar across groups. Macrophage abundance was comparable between genotypes, and macrophages were most abundantly retrieved in the mesenteric peritoneal membrane (Fig. 3c). Mesenteric but not omental adipose regions contained abundant eosinophils, many of which were still present in the DARL mice that lacked mature adipocytes (Fig. 3c).

To consider the possibility that the adaptive cytolytic immune compartment might influence ID8p53^−/−^Brca2^−/−^ GFP Luc tumor cell seeding in the peritoneal cavity, we studied tumor growth in triple knockout mice that do not express genes encoding recombination activating gene 2 (Rag2), CD47, or the X-linked interleukin 2 receptor gamma chain (Il2rg) that comprises part of the receptor for several cytokines impacting lymphocyte and NK cell homeostasis. These mice are genetically deficient in NK cells and T or B lymphocytes. When these mutant mice or control mice were i.p. injected with ID8p53^−/−^Brca2^−/−^ GFP Luc tumor cells, tumor expansion kinetics were comparable between WT and triple KO mice (Fig. 3e). At the endpoint, importantly, there was also no difference in tumor burden in the omentum quantified by ex vivo BLI (Fig. 3f).

To reinforce this finding and to determine if the lack of adipocytes, coupled with the lack of T lymphocytes and NK cells, affected the tumor expansion, PARC and DARL mice were treated with depleting CD8, CD4, and NK1.1 antibodies after injection with the less aggressive ID8 parental tumor cell line labeled with firefly luciferase. Blood samples were analyzed via flow cytometry to confirm lymphocyte or NK cell depletion throughout the experiment (Suppl. Fig. 5). Tumor progression itself was associated with reduced circulating NK cells but not T cells (Suppl. Fig. 5). These depleting antibodies used in PARC mice, replete with adipose tissue, recapitulated the lack of an impact on tumor expansion observed in the triple knockout mice. Conducting this immune cell depletion experiment in DARL mice lacking mature adipocytes also did not alter tumor expansion in the peritoneal cavity (Fig. 3g).

Altogether, these data indicate that the absence of mature adipocytes does not substantially alter the abundance of major immune populations within the omentum or mesentery at steady state. While differences in functional activation states cannot be excluded, genetic ablation or antibody-mediated depletion of lymphocytes and NK cells did not significantly alter peritoneal tumor expansion or omental tumor burden. These findings suggest that broad modulation of lymphocyte or NK cell abundance is unlikely to account for the omentum’s dominant role in ovarian cancer localization and growth in this model.

### Metabolic syndrome does not exacerbate OC growth in the peritoneum

Although overall tumor burden, organ distribution, and spatial localization were comparable between PARC and DARL mice following metabolic normalization by fat pad transplant surgery, systemic metabolic dysregulation or metabolic syndrome could independently alter omental tumor growth. To evaluate this possibility, Adiponectin^Cre/+^ DTA^fl/+^ lipoatrophic mice with persistent metabolic abnormalities (Suppl. Fig. 2) that were not corrected by fat transplant were injected i.p. with ID8p53^−/−^Brca2^−/−^ GFP Luc tumor cells. Tumor growth was compared with that of female littermate controls lacking Cre expression and therefore retaining normal adipose tissue distribution.

**Fig. 4:**
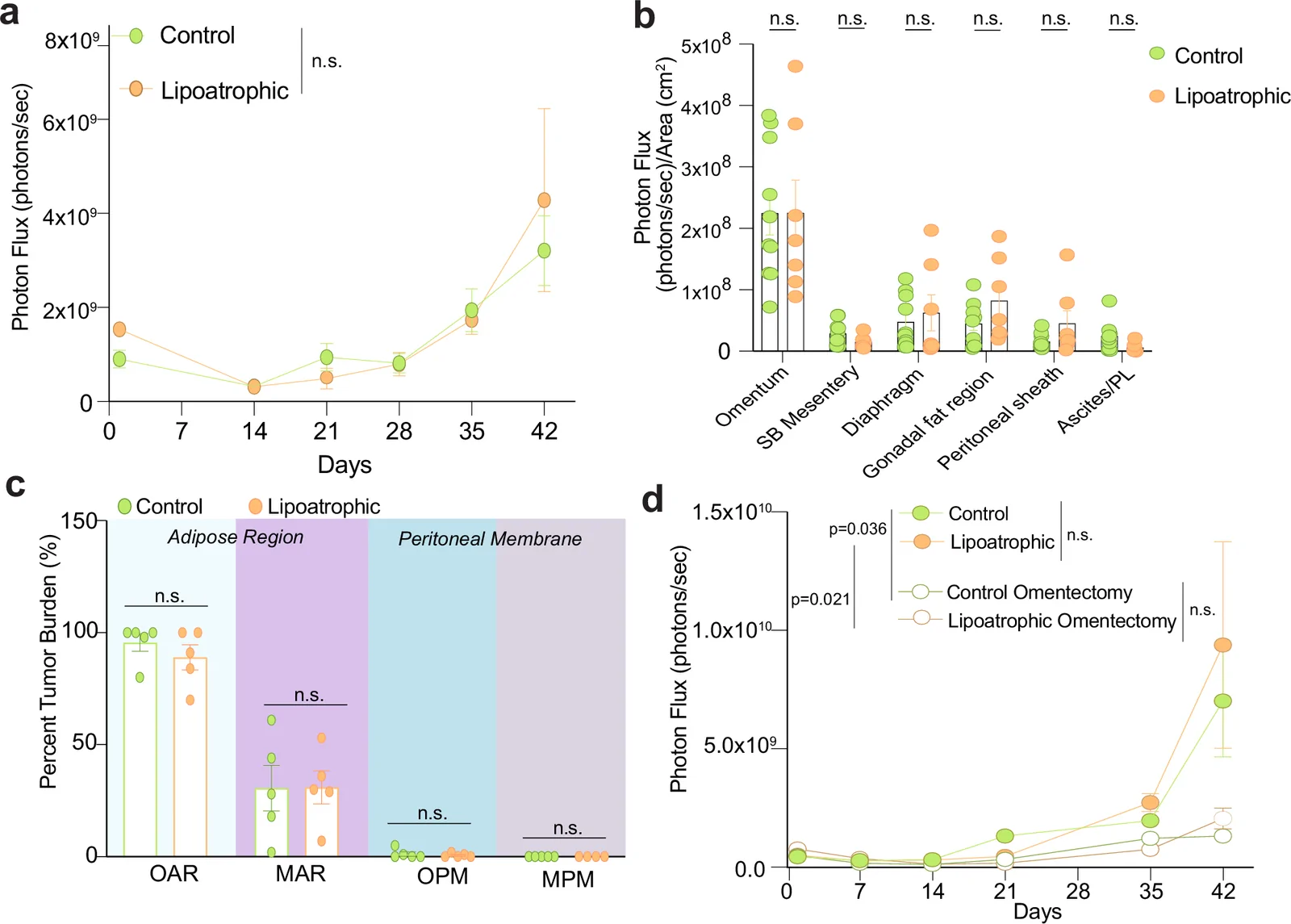
Metabolic syndrome and lipoatrophy do not mediate OC omental tropism. All experiments were performed following intraperitoneal injection of 5×10^6^ ID8p53^−/−^Brca2^−/−^ GFP Luc tumor cells. **a** Quantification of in vivo tumor burden by bioluminescence imaging (BLI) in control (*n* = 4) and lipoatrophic (*n* = 5) mice following i.p. tumor cell injection. Area under the curve (AUC) of total photon flux over time was calculated per mouse and compared between groups using a two-tailed Mann–Whitney test, n.s. **b** Distribution of tumor burden across peritoneal organs based on quantification of photon flux per organ area from ex vivo BLI of pinned peritoneal organs in control (*n* = 10) and lipoatrophic (*n* = 7) mice six weeks after tumor injection. Pairwise comparisons of absolute photon flux between control and lipoatrophic mice were performed for each organ using two-tailed Mann–Whitney tests with Holm–Šídák correction for multiple comparisons. SB small bowel, PL peritoneal lavage. **c** Percent tumor burden across anatomical compartments of the peritoneum quantified from FIJI particle-analysis measurements of tumor nodules in ex vivo bioluminescence images of pinned peritoneal organs from control (*n* = 5) and lipoatrophic (*n* = 5) mice six weeks after tumor injection. Compartments include the omentum adipose region (OAR), mesenteric adipose region (MAR), omentum peritoneal membrane (OPM), and mesenteric peritoneal membrane (MPM). Pairwise comparisons between genotypes were performed using two-tailed Mann–Whitney tests with Holm–Šídák correction for multiple comparisons (n.s.). **d** Quantification of in vivo tumor burden by BLI in control and lipoatrophic mice that received either sham surgery (control, *n* = 10; lipoatrophic, *n* = 7) or omentectomy (control, *n* = 13; lipoatrophic, *n *= 7), followed by i.p. injection of tumor cells. AUC of total photon flux over time was calculated for each mouse and compared using a Kruskal–Wallis test (*p* = 0.0002) followed by Dunn’s multiple comparison tests (exact *p*-values shown in the panel). Data are presented as mean ± SEM. Throughout, n indicates the number of individual mice, each an independent biological replicate.

Despite elevated circulating triglycerides and impaired glucose tolerance (Suppl. Fig. 2), Adiponectin^Cre/+^ DTA^fl/+^ lipoatrophic mice exhibited comparable overall tumor burden relative to control littermates (Fig. 4a). Omentectomy reduced the pace of tumor growth in both groups (Fig. 4b). The omentum still comprised 50-60% of total tumor burden regardless of metabolic status (Fig. 4c). Approximately 80% of the omental adipose-associated region was occupied by tumor nodules in both cohorts. In contrast, less than 10% of the adjacent peritoneal membrane was colonized. Similarly, ~30% of the mesenteric adipose-associated region was occupied by tumors in both groups, with minimal involvement of the corresponding peritoneal membrane (Fig. 4d). Relative sparing of the peritoneal membrane at endpoint does not exclude a transient early interaction with mesothelial cells but indicates that sustained tumor accumulation occurs predominantly within adipose-associated regions.

Together, these findings indicate that systemic hypertriglyceridemia and glucose dysregulation did not detectably alter peritoneal tumor growth or the omentum’s dominant contribution to tumor burden in this model.

### PARC and DARL mice have similar cell composition and high FABP4 endothelial expression in the omentum compared to the mesentery

The persistent dominance of the omentum as the primary site of tumor growth in both PARC and DARL mice suggested that intrinsic features of the omentum itself, independent of mature adipocytes, immune depletion, or systemic metabolic factors, may underlie its unique permissiveness to metastasis. We therefore sought to define the cellular composition and gene expression programs of the omentum relative to the mesentery, to identify tissue-intrinsic mechanisms that could explain why the omentum remains a preferential metastatic niche even in the absence of mature adipocytes.

We performed single-cell RNA sequencing to profile the omentum and mesentery in lipoatrophic mice following fat transplantation and in their control littermates that received sham surgeries. The peritoneal membrane and adipose-associated regions of the omentum and mesentery from PARC (*n* = 8) and DARL (*n* = 8) mice were disaggregated to generate cell suspensions for single-cell RNA sequencing. CD45^+^ and CD45^-^ cells were sorted separately, combined, and submitted for single-cell RNA sequencing to allow for unbiased assessment of any population, immune or stromal, that might differentially shift between the PARC and DARL mice peritoneal organs (Suppl. Fig. 4b). As a result, the relative proportions of CD45⁺ and CD45⁻ cells observed in the scRNA-seq dataset reflect native tissue composition together with known differences in recovery between immune and stromal populations, rather than artificial enrichment of specific lineages. The resulting dataset included 69,807 cells classified in 14 Louvain clusters, of which 25,617 were *Cd45*^*-*^ and 44,190 were *Cd45*^+^.

To enable multiplexing of biological replicates, hashtags or antibody-derived tags (ADTs) were used for cell hashing^52^, as the small size of peritoneal membrane tissues necessitated pooling samples across animals while preserving the identity of each mouse and tissue of origin. Using this approach, eight biological replicates per tissue were individually labeled before pooling. This approach was effective, as association with 1 of the 8 hashtags collectively accounted for 62.2% of the data after standard demultiplexing (see Methods). The two most abundant ADT hashtags per cell differed by a median of 630 counts, enabling confident assignment of a single hashtag per cell (Suppl. Fig. 6a) and the identification of biological replicates across tissues. The remaining data were identified as multiplet (20.7%) or lacked a confident hashtag signal (17.1%; Suppl. Fig. 6b, c). Biological variability across tissues and cell types was preserved across replicates (Suppl. Fig. 6d–f).

**Fig. 5:**
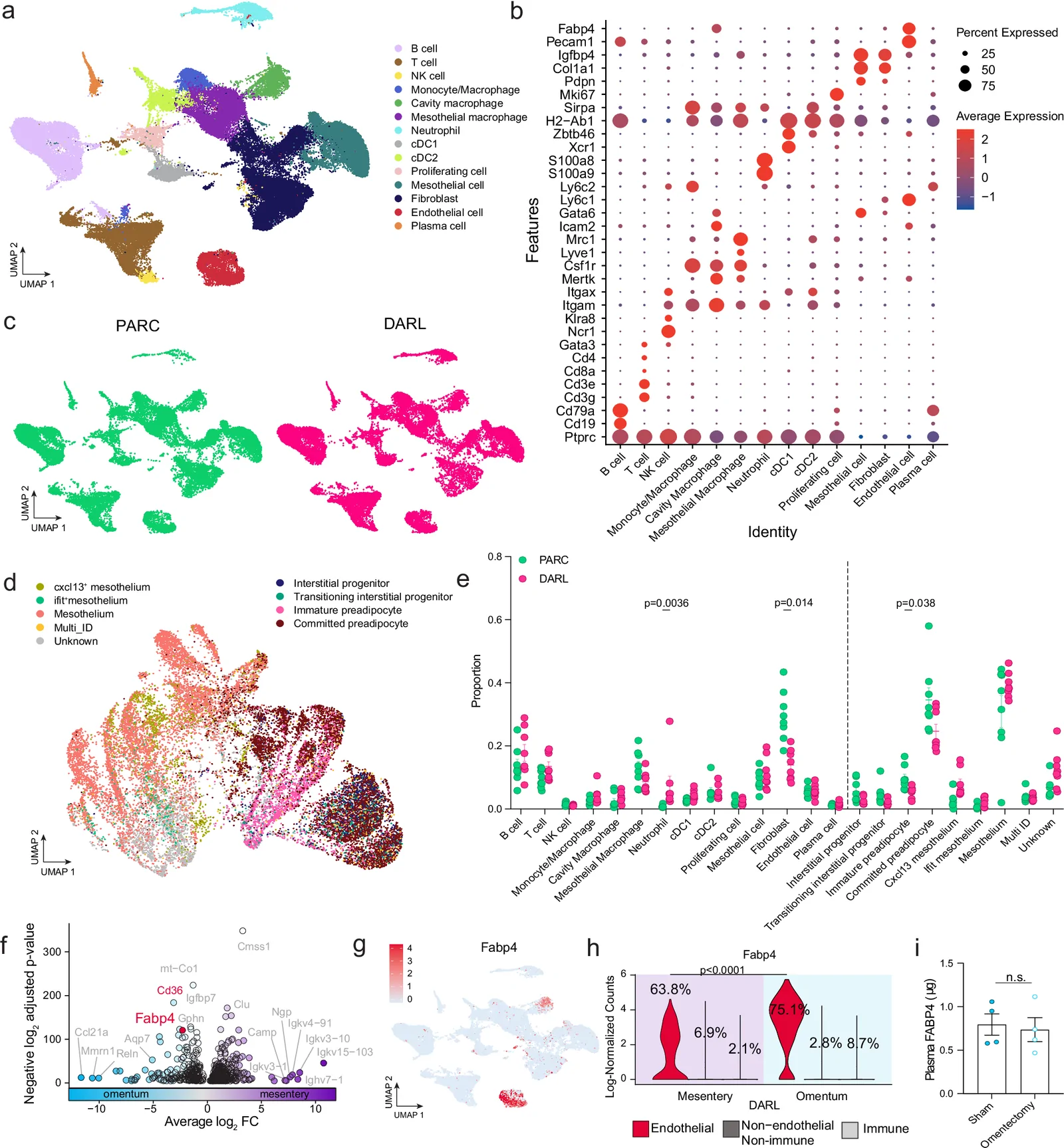
Cellular composition is preserved after mature adipocyte depletion with FABP4 enrichment in omental endothelial cells. **a** UMAP visualization of all single cells collected from separately isolated adipose and peritoneal membrane regions of the omentum and mesentery in PARC (*n* = 8) and DARL (*n* = 8) mice. **b** Dot plot showing expression of canonical marker genes used to annotate major cell populations identified in panel a. Dot size indicates the percentage of cells expressing each gene, and color indicates average expression level. **c** UMAP visualization of PARC (left) and DARL (right) cells displayed separately. **d** Subclassification of CD45^-^ non-endothelial stromal cells. Adipocyte progenitor populations were annotated using established APC markers (Lin^−^ CD29⁺ CD34⁺) that distinguish progenitors from mature adipocytes, derived from stromal vascular fraction datasets of naïve mouse adipose tissue^57^. Mesothelial subtypes were annotated using CD45^−^/CD41^−^/Ter119^−^/CD31/PDPN^+/−^ stromal cells previously described in the naive mouse omentum^35^. **e** Comparison of the proportion of cell type in PARC and DARL mice. Each dot represents an individual mouse. Pairwise comparisons between genotypes were performed using two-tailed Mann–Whitney tests with Holm–Šídák correction for multiple comparisons **f** Differential gene expression analysis in the omentum (blue) and mesentery (purple) identifies transcripts enriched in the omentum, including Fabp4. **g** UMAP visualization of Fabp4 expression across all cells from the omentum and mesentery in PARC (*n* = 8) and DARL (*n* = 8) mice. **h** Violin plots of log-normalized Fabp4 expression stratified by tissue and major cell class. Fabp4 expression is highest in endothelial cells within the omentum compared with the mesentery. Two-tailed Mann–Whitney test, *p* < 0.0001. **i** Quantification of circulating FABP4 protein levels in plasma collected from C57BL/6 J sham (*n* = 4) or omentectomized mice (*n* = 4). Two-tailed Mann–Whitney test, n.s. Data are presented as mean ± SEM. Throughout, n indicates the number of individual mice (single-cell data, *n* = 8 mice per genotype), each an independent biological replicate.

Cells from the omenta (peritoneal membrane and adipose region) and mesentery (peritoneal membrane and adipose region) of PARC and DARL mice were clustered based on differentially expressed genes, with each cluster defined by canonical markers for specific immune and stromal populations (Fig. 5a, 5b). The marker genes used for cluster assignments are shown in the dot plot in Fig. 5b. Overall, there were minimal differences in global cell clustering between PARC and DARL mice (Fig. 5c), with no cell cluster uniquely present or absent in either genotype. As anticipated, mature adipocytes were not recovered in the single-cell RNA-sequencing dataset, including in PARC samples, due to their large size and fragility^53^, consistent with known limitations of droplet-based single-cell platforms in capturing large lipid-laden cells.

To refine CD45⁻ stromal heterogeneity further, we subsequently applied Capybara, a computational single-cell classifier^54^, to improve mesothelial and adipose-lineage identities using biologically relevant reference datasets, guided by prior evidence implicating these populations in OC metastasis^55,56^. Specifically, we leveraged an omentum-focused stromal atlas to refine mesothelial populations^35^ and a high-resolution adipose lineage reference to classify pre-adipocytes into defined precursor states, including interstitial progenitors and committed pre-adipocytes^57^ (Fig. 5d). These refined assignments were cross-validated using canonical marker expression (Suppl. Fig. 6g). Importantly, mesothelial cell abundance did not differ significantly between PARC and DARL mice in tumor-naïve tissue (Fig. 5e), indicating preservation of mesothelium independent of mature adipocyte status. We anticipated that pre-adipocyte populations would be amplified in DARL mice, given the absence of mature adipocytes that express adiponectin. However, pre-adipocyte populations were similar between the cohorts, except for the proportion of immature preadipocytes, which was modestly increased in PARC mice (Fig. 5e). The proportions of most immune and stromal cells were comparable between PARC and DARL mice as well, except for fibroblasts, which were reduced in DARL mice, and neutrophils, which were increased in DARL mice (Fig. 5e).

Having established that immune and stromal cell composition was largely conserved between PARC and DARL mice, we next examined differential gene expression across genotypes. Differential gene expression analysis identified Retnla and Cfd (complement factor D) as enriched in PARC relative to DARL cells. This differential gene expression serves as a strong positive control in validating our model, given that these genes are well-established adipokines^58,59 ^(Suppl. Fig. 6h, Suppl. Data 1). Supplementary Data 1 summarizes pooled differential gene expression across all captured cells from both the omentum and mesentery in PARC and DARL mice, rather than performing organ- or cell-type-specific comparisons.

We next explored organ-specific transcriptional differences between the omentum and mesentery to identify tissue-intrinsic programs that may contribute to tumor growth patterns. Notably, *Fabp4* expression was significantly higher in the omentum than in the mesentery, including in DARL mice lacking mature adipocytes (Fig. 5f). Because FABP4 has previously been linked to adipocyte-mediated lipid transfer in OC peritoneal tumor growth^11,60,61^, this adipocyte-independent enrichment, together with our finding that mature adipocytes are not required for tumor growth, prompted us to investigate alternative cellular sources of FABP4 in the omentum. Among the populations captured by single-cell RNA sequencing, Fabp4 transcripts were detected in cavity macrophages and endothelial cells, with the highest expression observed in endothelial cells (Fig. 5a, b, g, h).

To further resolve endothelial heterogeneity, endothelial cells were classified into arterial, venous, capillary, and lymphatic subtypes using canonical markers and Capybara-based matching to a curated endothelial reference^62^ (Suppl. Fig. 6i, j). These assignments were consistent with canonical gene expression (Suppl. Fig. 6j). Most of the endothelial cells in our dataset were blood-vascular rather than lymphatic endothelium, as indicated by Lyve1 expression (Suppl. Fig. 6i, j). *Fabp4* was broadly expressed in the endothelium but was exceptionally high in endothelial cells with lower levels of *Cxcl12* (Suppl. Fig. 6i, j). Importantly, *Fabp4* expression was significantly higher in omental endothelial cells compared to mesenteric endothelial cells and immune populations from either tissue, including in DARL mice that lack mature adipocytes yet retain tumor growth (Fig. 5h).

Because endothelial cells have been reported to contribute to circulating FABP4^63^, we next examined whether the omentum might substantially influence systemic FABP4 levels. However, plasma FABP4 levels measured by ELISA did not differ between mice that underwent omentectomy and those that received sham surgery (Fig. 5i). Thus, although omentectomy reduced tumor burden (Fig. 2l), this effect is unlikely to be mediated by changes in systemic FABP4 levels. Instead, these data are consistent with a local role for endothelial FABP4 within the omental microenvironment.

Collectively, these findings demonstrate that endothelial cells are a prominent source of Fabp4 transcripts in the omentum among the profiled populations and provide a framework for interpreting FABP4-dependent tumor phenotypes independent of mature adipocytes.

### Endothelial FABP4 contributes to omental tumor burden and marks a vascular population conserved across steady-state and metastatic human omentum

**Fig. 6:**
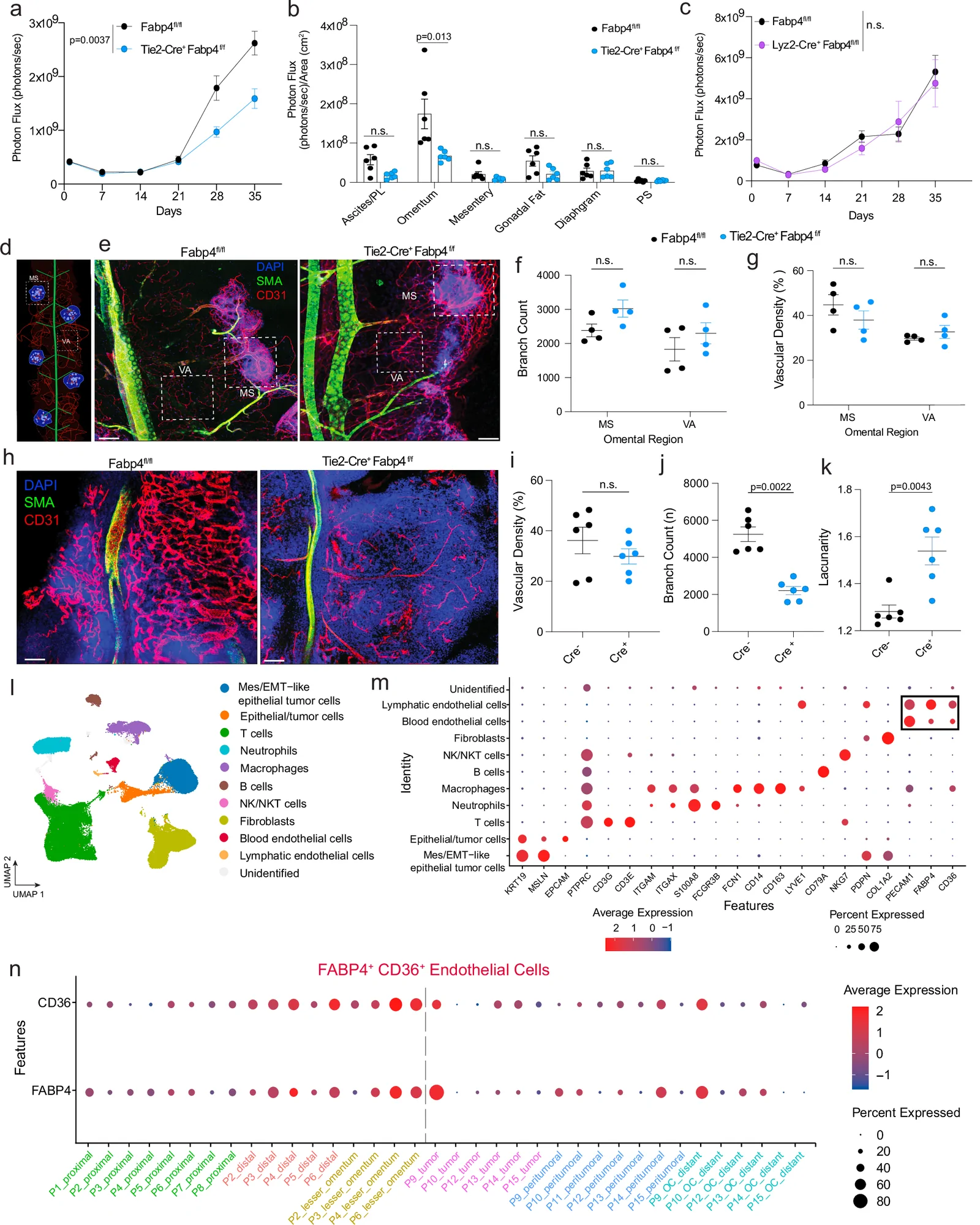
Endothelial Fabp4 promotes omental tumor growth and defines a conserved human vascular population. Mice received i.p. 5 × 10^6^ ID8p53^−/−^Brca2^−/−^ GFP Luc tumor cells. **a** In vivo peritoneal tumor burden (bioluminescence imaging, BLI) of Tie2-Cre^+^ Fabp4^fl/fl^ mice (*n* = 14) and Fabp4^fl/fl^ littermate controls (*n* = 12); area under the curve (AUC) of total photon flux, two-tailed Mann–Whitney test, *p* = 0.0037. **b** Tumor burden across peritoneal organs (ex vivo photon flux per organ area) at day 35 (*n* = 6/group); per-organ two-tailed Mann–Whitney tests with Holm–Šídák correction, omentum *p* = 0.013. PS peritoneal sheath, SB small bowel, PL peritoneal lavage. **c**. In vivo tumor burden (BLI) in Lyz2-Cre^+^ Fabp4^fl/fl^ mice and Fabp4^fl/fl^ controls (*n* = 6/group); two-tailed Mann–Whitney test, n.s. **d** Schematic of omental microanatomy showing milky spot (MS) and vascular area (VA) compartments. **e** Representative 3D confocal images of steady-state omental vasculature in Fabp4^fl/fl^ (left) and Tie2-Cre^+^ Fabp4^fl/fl^ (right) mice; CD31, red; SMA, green; DAPI, blue; scale bars, 100 μm. **f** Vascular density (CD31^+^ volume/region of interest) in MS and VA of tumor-free omentum (*n* = 4/genotype); two-tailed Mann–Whitney test, n.s. **g** Total branch count in MS and VA of tumor-free omentum (*n* = 4/genotype; each point is the per-mouse mean of 3–5 regions); two-tailed Mann–Whitney test, n.s. **h** Representative 3D confocal images of tumor-associated vasculature at five weeks; labels and scale bars as in (**e**). **i** Vascular density of tumor-associated vasculature (*n* = 6/genotype); two-tailed Mann–Whitney test, n.s**. j** Total branch count within tumor nodules (*n* = 6/genotype; each point is the per-mouse mean of three nodules); two-tailed Mann–Whitney test, *p* = 0.0022. **k** Lacunarity of tumor-associated vasculature (*n* = 6/genotype; higher values, greater gap-size heterogeneity); two-tailed Mann–Whitney test, *p* = 0.0043. **l** UMAP of human omental single-cell RNA-sequencing: non-metastasized omentum (benign or non-malignant, *n* = 8 patients) and omental metastases (OC or borderline tumors, *n* = 7 patients), reanalyzed from GSE286057 (ref. ^6^). **m** Dot plot of canonical markers identifying cell clusters. **n** Dot plot of CD36 and FABP4 in blood endothelial cells (PTPRC^−^ PECAM1^+^ LYVE1^−^ PDPN^−^) by spatial location; dashed line separates non-cancerous omentum (left) from OC metastases (right); dot size, percent of cells expressing; color, mean expression. Data are mean ± SEM; scale bars are defined in the panels. Throughout, n indicates the number of individual mice (panels **a**–**k**) or patients (panels **l**–**n**), each an independent biological replicate.

To test the role of FABP4 expressed in endothelium in impacting the growth of OC in the peritoneal cavity, we bred endothelial-specific FABP4 knockout mice (Tie2-Cre⁺ Fabp4^fl/fl^) along with littermate controls^63^. Following i.p. injection of ID8p53^−/−^Brca2^−/−^ GFP Luc tumor cells, tumor burden and expansion lagged in mice lacking endothelial FABP4 (Fig. 6a). During the exponential growth phase (days 21–35), tumor burden in control mice grew at 0.137 ± 0.014 day^−^^1^ compared with 0.097 ± 0.011 day^−^^1^ in Tie2-Cre⁺ Fabp4^fl/fl^ mice (doubling time 5.08 vs 7.12 days), yielding a significant time-by-genotype interaction in a mixed-effects model (b = −0.039 day^−^^1^, 95% CI − 0.069 to −0.009, *p* = 0.010). This corresponds to an approximately 29% reduction in the exponential tumor growth rate. In contrast, instantaneous growth rates during the final week (days 28–35) were comparable between genotypes, indicating that endothelial Fabp4 loss primarily affects early tumor expansion rather than late-stage growth velocity. This early attenuation in exponential growth was associated with an approximately 1.9-fold lower endpoint tumor burden in Tie2-Cre⁺ Fabp4^fl/fl^ mice. Consistent with this temporal shift in tumor expansion, ex vivo BLI revealed a marked reduction in tumor signal within the omentum (Fig. 6b), supporting a role for FABP4-expressing endothelial cells in omental tumor expansion.

Because FABP4 was also expressed in macrophages, although to a lesser extent, in the murine omentum (Fig. 5g), and Tie2 can be induced in some hematopoietic cells^64^, we tested whether myeloid-lineage FABP4 played a comparable role. Lyz2-Cre⁺ Fabp4^fl/fl^ mice were injected i.p. with ID8p53−/− Brca2−/− GFP Luc tumor cells. In contrast to the endothelial cell deletion model, no significant difference in tumor burden was observed (Fig. 6c), indicating that the tumor-promoting function of FABP4 in endothelial cells is unlikely to be explained solely by myeloid lineage FABP4.

A natural question arising from these cell-type-specific genetic data is whether the reduced tumor burden in Tie2-Cre⁺ Fabp4^fl/fl^ mice reflects an antecedent defect in vascular development rather than a context-dependent function engaged during tumor colonization. The omental vascular network is organized into milky spot (MS) and vascular area (VA) compartments (Fig. 6d). Three-dimensional whole-mount imaging of tumor-naive omentum at steady state indicated that the vasculature was qualitatively comparable between Fabp4^fl/fl^ controls and Tie2-Cre⁺ Fabp4^fl/fl^ mice, with a central large SMA^+^ vessel traversing the tissue and a fine CD31^+^ capillary network distributed around it in both genotypes (Fig. 6e). Branch count and vascular density were statistically equivalent in both the milky spot and vascular area compartments (Fig. 6f,g), indicating that constitutive absence of endothelial FABP4 does not disrupt the baseline omental vascular network.

In the context of tumor colonization, however, omental architectural differences became apparent. Fabp4^fl/fl^ controls displayed a dense, highly branched CD31^+^ vascular network radiating outward from major vessels in tortuous, proliferative trajectories consistent with tumor-associated angiogenesis, whereas Tie2-Cre⁺ Fabp4^fl/fl^ omentum retained the main vessel architecture but showed sparser microvasculature, with fewer and simpler vessel trajectories extending into the surrounding tissue (Fig. 6h). Quantitative analysis captured this distinction across two complementary measures of network architecture. Vascular density did not differ significantly between genotypes (Fig. 6i), indicating that these differences reflect altered network geometry rather than reduced vessel volume. Total branch count, however, was approximately 58% lower in Tie2-Cre⁺ Fabp4^fl/fl^ tumor-bearing omentum relative to controls (Fig. 6j), reflecting reduced angiogenic branching. Consistent with this, lacunarity, which quantifies spatial heterogeneity in the vascular gap-size distribution, was significantly higher in Tie2-Cre⁺ Fabp4^fl/fl^ omentum than in Fabp4^fl/fl^ controls (Fig. 6k), indicating that endothelial FABP4-deficient tumors contained a sparser vascular network with larger and more irregular avascular spaces.

Having established that endothelial FABP4 regulates tumor-associated vascular architecture in the omentum, we next asked whether a similar endothelial population exists in the human omentum. To address this, we reanalyzed published single-cell RNA-sequencing datasets from human omental tissue. We reanalyzed a spatially annotated human omentum single-cell RNA-sequencing dataset comprising 36 samples from 15 patients, including benign omental nodules and OC omental metastases, with matched regions classified as proximal to the tumor, peritumoral, or distal to the primary tumor^6^ (Fig. 6l–n). This spatial annotation enabled direct comparison of endothelial transcriptional programs across tumor-associated and non-tumor-associated omental niches (Fig. 6l, Suppl. Fig. 7a, b).

Across all spatial regions and disease states, *FABP4* and *CD36* expression was enriched in endothelial cells relative to other stromal and immune populations, including macrophages, which expressed these genes at lower levels (Fig. 6n). Owing to the size of this multi-patient dataset and the large number of endothelial cells captured, blood and lymphatic endothelial populations could be readily distinguished and analyzed separately. In both endothelial compartments, coordinated *FABP4* and *CD36* expression was observed across non-metastasized omental regions, including tissue proximal and distal to benign disease, indicating that this expression pattern is present in the non-metastasized omental vasculature of the human omentum (Fig. 6n).

To independently confirm endothelial *FABP4* expression in the absence of malignancy and using an analysis framework aligned with our murine single-cell studies, we analyzed benign human omental tissue obtained from the Washington University OB/GYN tissue bank (Suppl. Fig. 7c–f). Intraoperatively, the omentum appeared grossly unremarkable, and final pathology confirmed the absence of macroscopic or microscopic omental tumor involvement, with disease confined to the ovary. Although subtle surgery-associated remodeling cannot be excluded, this specimen lacked overt tumor-associated architectural or cellular changes and therefore provides a near-steady-state comparator for human omental tissue, meeting the same criteria as the non-metastasized omentum samples used as controls in the published dataset^6^.

This human sample enabled separate profiling of the peritoneal membrane and adjacent adipocyte-rich region (GSE300038) (Suppl. Fig. 7), similar to our earlier separation of these distinct regions in mice (Fig. 3). Unsupervised clustering of the adipocyte-rich region identified 11 distinct cell populations based on canonical gene expression (Suppl. Fig. 7d-e). *FABP4* expression was enriched in endothelial cells expressing PECAM1, as well as in vascular mural cells expressing contractile and smooth muscle–associated genes, including ACTA2 and PDGFRB (Suppl. Fig. 7d). Notably, *CD36* was co-expressed within the same endothelial population, and blended feature plots confirmed coordinated *FABP4* and *CD36* expression (Suppl. Fig. 7f). Although the limited number of endothelial cells in this single-patient dataset precluded subdivision into blood endothelial populations, *FABP4* and *CD36* expression was nonetheless readily detected within the endothelial compartment, corroborating the presence of this population in the human omentum at near steady-state conditions.

We next examined whether this endothelial signature is maintained in the context of metastatic disease. In tumor-associated omental regions, *FABP4* and *CD36* expression within both blood endothelial cells became more heterogeneous, with peritumoral regions displaying intermediate and variable expression states (Fig. 6n). Importantly, endothelial cells across tumor-associated regions retained expression of canonical lineage markers, indicating preservation of endothelial identity. *FABP4* and *CD36* expression remained detectable across benign, peritumoral, and metastatic regions, suggesting that this endothelial expression state is established before tumor implantation and persists as tumors develop and expand.

Together, these data indicate that the *Fabp4*⁺ endothelial cells identified in murine models are conserved in the human omental blood endothelium and are maintained under both steady-state and metastatic conditions. In mice, endothelial-specific *Fabp4* deletion attenuated early tumor expansion and was associated with reduced vascular branching and altered network geometry during tumor-associated angiogenesis. When considered alongside the reduced omental tumor growth observed in Tie2-Cre⁺ Fabp4^fl/fl^ mice, these findings link endothelial FABP4 expression to vascular remodeling that may alter tumor expansion within the omentum.

## Discussion

Omental metastasis is a defining feature of ovarian cancer and has long been attributed to adipocyte-mediated metabolic support. Here, we directly tested this concept and found that mature adipocytes are dispensable for omental tumor localization, growth, and response to carboplatin. Instead, our data identify endothelial FABP4-associated vascular programs as contributors to early metastatic expansion within this niche, indicating that omental permissiveness is governed primarily by non-adipocyte features of the local microenvironment.

Although the omentum is often regarded as a uniformly adipose organ, it is anatomically heterogeneous. Adipose-rich regions are embedded within well-vascularized connective tissue and are covered by a mesothelial lining that rests continuously on the basement membrane, except in regions where milky spots form. In addition to these adipose-associated regions, the omentum also includes a thin, net-like, fenestrated peritoneal membrane composed of mesothelial cell layers that are poorly vascularized and enclose diffuse collagen fibers^3^.

Multiple omental cell populations have been implicated in metastatic progression. Mesothelial cells form the initial barrier to the dissemination of tumor cells and are reprogrammed to secrete pro-metastatic factors such as ANGPTL4 and STC1^55^. Additional studies suggest that neutrophil extracellular traps^65^, omental macrophages^9,66^, blood vessels^67^, fibroblasts^68^, or milky spots^69–73^ support tumor retention, survival, or expansion within the peritoneum. Adipocytes have been widely regarded as key determinants of omental permissiveness, given their capacity to support tumor fatty acid metabolism^74–76^, which motivated us to directly test whether mature adipocytes themselves are required for OC growth in the omentum.

Tumors preferentially engrafted in adipose-associated regions of the omentum and mesentery rather than in the adipose-free peritoneal membrane, yet the mature adipocytes demarcating these regions were not required for colonization. Because Adiponectin-Cre is active only in terminally differentiated adipocytes, the Adiponectin-Cre x Rosa-DTA model selectively eliminates mature adipocyte functions, including lipid storage and adipokine secretion, the functions central to prior adipocyte-driven models^11^, while preserving adipocyte progenitors. Remarkably, even in the complete absence of mature adipocytes, tumors continued to localize to and expand within regions normally occupied by adipose tissue.

Indeed, surgical removal of the omentum reduced tumor burden even in lipoatrophic mice, indicating that anatomical features of the omentum itself, rather than mature adipocytes, govern niche permissiveness. This central finding was concordant across multiple fallopian tube-derived HGSOC models, including BPPNM and KPCA, despite the known heterogeneity among syngeneic OC models^77^. The absence of mature adipocytes also did not alter carboplatin response.

Because the omentum contains unique lymphoid aggregates or milky spots, we next asked whether immune cell populations could account for tumor localization in this niche. Although flow cytometry and transcriptomic analyses revealed differences in immune composition between the omentum and mesentery, depletion or genetic ablation of T, B, and NK cells did not increase omental tumor burden, unlike settings where immune surveillance constrains growth in lymphocyte-deficient mice^78^. This interpretation is consistent with findings from multiple fallopian tube-derived HGSOC cell lines, such as the KPCA and BPPNM lines^49^, and with clinical findings for checkpoint inhibitors in HGSOC^79^. While adipocytes have been proposed to be immunosuppressive^23,80^, adaptive immune depletion did not affect tumor progression even in adipocyte-depleted mice, arguing against this mechanism here.

Consistent with the equivalent tumor phenotypes between control and lipoatrophic mice, it is unsurprising that single-cell RNA sequencing did not reveal major differences in immune or stromal populations between the omentum and mesentery, regardless of whether mature adipocytes were present. Prior work implicating FABP4-expressing adipocytes in OC progression^11^ relied on whole-body knockout models, which cannot separate adipocyte FABP4 from its expression in endothelium and macrophages^81,82^. Recent work has also demonstrated that macrophage-intrinsic FABP4 promotes lipid metabolism and metastatic progression in breast cancer^83^, highlighting that the dominant cellular source of FABP4 function can vary by tumor type and metastatic context.

Seeking an alternative basis for permissiveness, we found that omental FABP4 expression exceeded mesenteric levels even in adipocyte-depleted mice. Within the omentum, endothelial cells represented the predominant non-adipocyte source of *Fabp4* transcripts among the successfully profiled cell populations in PARC and DARL mice. This finding indicates that elevated endothelial FABP4 is an intrinsic feature of the omental microenvironment, rather than a consequence of local adiposity. Importantly, these endothelial lipid-handling programs extended beyond FABP4 and persisted even in the complete absence of mature adipocytes. Because Fabp4 expression in adipocyte progenitors is minimal^42,84^, and adipocyte depletion did not reduce tumor burden, adipocyte-lineage Fabp4 alone cannot account for tumor growth.

Extending these findings, we show that genetic deletion of FABP4 in endothelial cells (Tie2-Cre⁺ Fabp4^fl/fl^)^63^ significantly reduces omental tumor burden, primarily by attenuating early tumor expansion. Although Tie2-Cre can recombine in some hematopoietic cells, including myeloid lineages^64^, the absence of a phenotype in Lyz2-Cre Fabp4^fl/fl^ mice argues against a dominant myeloid contribution. Although macrophages play important roles in OC metastasis^9,10^ and FABP4 has been shown to promote metastasis in other tumor contexts^83^, the absence of a phenotype in Lyz2-Cre Fabp4^fl/fl^ mice indicates that *Fabp4*-expressing macrophages are not required for omental tumor growth in this model. Together with the marked enrichment of FABP4 in omental endothelial cells observed in murine and human single-cell RNA sequencing, these data support a functional role of endothelial FABP4 in promoting omental tumor growth. This role is local rather than systemic, as omentectomy reduced tumor burden without changing plasma FABP4, which has been implicated in systemic metabolic regulation^63,85,86^.

These findings fit growing evidence that lipid-handling proteins serve distinct endothelial and adipocyte functions. For example, CD36, SR-BI, and Caveolin-1 each support lipid storage or systemic lipid handling in adipocytes but mediate vascular fatty acid uptake, lipoprotein transcytosis, and membrane and vascular regulation in the endothelium, respectively^87–95^. Endothelial FABP4 is thus best interpreted as a vascular metabolic program rather than a surrogate of adipocyte biology.

Given the endothelial targeting of Tie2-Cre^+^, we asked whether loss of FABP4 perturbs baseline vasculature. Three-dimensional whole-mount imaging of tumor-naïve omentum in Tie2-Cre^+^ Fabp4^fl/fl^ mice demonstrated preserved baseline vascular architecture, arguing against a developmental defect. In tumor-bearing omentum, however, FABP4 deficiency produced a less expansive angiogenic response, with reduced branching and a sparser, more spatially heterogeneous network. These findings are consistent with a context-dependent role for endothelial FABP4 during tumor-associated vascular remodeling rather than during baseline vessel formation and maintenance.

This phenotype supports a model in which endothelial FABP4 promotes tumor-associated angiogenic remodeling during early colonization. FABP4 has been reported to act downstream of VEGFA–DLL4–NOTCH signaling, a pathway central to angiogenic switching dynamics^82^. Tumor-vascular FABP4 knockdown reduces angiogenesis and peritoneal spread without complete vessel ablation^96^, supporting a role in modulating angiogenic capacity rather than vascular survival. Whether our tumor vasculature results reflect altered tip-cell dynamics, endothelial metabolic fitness, or barrier function remains to be determined.

A role for FABP4-mediated lipid transfer cannot be ruled out, but systemic lipid abundance does not account for omental tropism here. Elevated circulating triglycerides in the lipoatrophic mice did not increase tumor burden, indicating that bulk lipid availability is not rate-limiting. Consistent with evidence that colonization reflects multifactorial niche interactions rather than single-nutrient dependencies^97^, endothelial FABP4 likely contributes to, rather than solely determines, the vascular and stromal adaptations of early metastasis.

The identification of a conserved *FABP4*⁺*CD36*⁺ endothelial population in human omentum, present both at steady state and in metastatic disease at varying locations from the primary tumor, indicates that this vascular program persists throughout tumor colonization. Because the metastatic omentum undergoes extensive remodeling with suppression of adipocyte-associated programs^6^, mature adipocytes were absent from our endpoint tumors. Together, these data reinforce the notion that adipocytes mark the permissive niche but are not required to sustain it, while endothelial cells remain a conserved feature across metastatic states.

Collectively, these findings challenge a long-standing adipocyte-centric model of OC omental metastasis. Rather than serving as essential drivers of metastatic growth, mature adipocytes appear to mark a permissive anatomical niche whose function is maintained independently of adipocyte-derived lipid storage or adipokine signaling. Our data instead identify endothelial FABP4-dependent vascular remodeling as a contributor to early metastatic expansion, shifting attention from adipocyte–tumor interactions toward the vascular and stromal mechanisms that govern omental metastases.

## Methods

This study complied with all relevant ethical regulations. All animal experiments were approved by the Washington University in St. Louis Institutional Animal Care and Use Committee (IACUC, protocol 25-0333), and human omental tissue was obtained under Washington University Institutional Review Board approval (IRB 201903051). Only female mice were used because ovarian cancer is a sex-specific disease. Sex was therefore not an experimental variable, and data are reported only for female animals. Unless otherwise stated, mice were housed at 20–22 °C with 30–70% relative humidity on a 12 h light/dark cycle.

### Mice

Adiponectin^Cre^ mice (The Jackson Laboratory, Bar Harbor, ME, USA: 028020, kindly provided by Dr. Steven Teitelbaum) were crossed with lox-stop-lox-Rosa26 diphtheria toxin (DTA) mice (The Jackson Laboratory: 010527: RRID: IMSR_JAX:010527), resulting in the ablation of mature adipocytes and consequent lipoatrophy in Adiponectin^Cre/+^ RosaDTA^f/+^ mice^.^ These lipoatrophic mice, which constitutively lack brown and white adipose tissue, and their littermate controls were maintained at thermoneutrality (30 °C) from birth on a 12 h light/dark cycle. In this model, adiponectin-expressing adipocytes are constitutively ablated upon terminal differentiation, resulting in a lifelong absence of mature adipocytes. CD19^Cre^ mice (Jackson Laboratory, Bar Harbor, ME, USA: 006785: RRID: IMSR_JAX:006785) were crossed with Rosa-TDTomato^fl/fl^ mice (Jackson Laboratory, Bar Harbor, ME, USA: 007914: RRID: IMSR_JAX:007914) to generate CD19^Cre/+^TD reporter mice, in which cells derived from the B cell lineage were Tomato^+^. Triple knockout B6.129S-Rag2^tm1Fwa^, Cd47^tm1Fpl^, Il2rg^tm1Wjl^/J mice (The Jackson Laboratory, Bar Harbor, ME, USA: 025730: RRID: IMSR_JAX:025730) and age-matched C57BL/6 J (The Jackson Laboratory, Bar Harbor, ME, USA: 000664: RRID: IMSR_JAX:000664) were purchased directly. Tie2-Cre^+^ Fabp4^fl/fl^ and Lyz2-Cre+ Fabp4^fl/fl^ mice were provided via MTA by Gökhan S. Hotamişligil, who generated them^63^. Animals were provided a standard chow diet (LabDiet 5053 – PicoLab Rodent Diet 20) and water ad libitum. All experiments and procedures were conducted in accordance with procedures and protocols approved by the Washington University in St. Louis Institutional Animal Care and Use Committee.

### Fat transplantation

Mature gonadal, inguinal, and axillary adipose depots collected from donor C57BL/6 J WT female mice or littermates, approximately 14–18 weeks old, were transplanted into the scruff of 7–10 week old lipoatrophic Adiponectin^Cre/+^ RosaDTA^fl/+^ female recipient mice, termed **d**istal **a**dipocyte **r**eceiving **l**ipoatrophic (DARL) mice post-surgery. The corresponding littermate control mice born with standard fat depots received parallel sham surgeries and are referred to as **p**eritoneal **a**dipocyte-**r**eplete **c**ontrol (PARC) mice post-sham surgery. The surgeries were conducted as previously described^98^ with a few modifications. After collection, the fat pads were placed in sterile saline on ice until the recipient mouse was prepared for surgery. The lipoatrophic mice and littermate controls were anesthetized by continuous inhalation of 1-2% isoflurane and placed on a heating pad in the prone position. A small 1–2 mm incision was made in the dorsal scruff of transplant-receiving mice. Three small pockets were made bilaterally. In the lipoatrophic mice, one graft (150–300 mg) was implanted subcutaneously into each pocket, for a total of six fat pads per mouse, all confined to the subcutaneous dorsal space. In the littermate control mice, these six pockets were made but not engrafted. The incision was closed with 5-0 nylon sutures. The mice were given a subcutaneous injection of extended-release buprenorphine (1 mg/kg) and placed overnight in an infant incubator warmed to 27 °C. Then, all mice were housed individually for a week to facilitate recovery before returning to five mice per cage. Adipose tissue was transplanted subcutaneously into the dorsal scruff, anatomically separated from the peritoneal cavity, in which tumors were later established by intraperitoneal injection.

### Omentectomy

Mice were anesthetized by continuous inhalation of 1–2% isoflurane and placed on a heating pad in the supine position. A small incision was made midline in the skin and then through the linea alba, a thin midline band of connective tissue along the peritoneal wall, to avoid bleeding. The greater omentum, including its associated peritoneal membrane, was carefully exposed and cauterized through this incision. The blood vessels supplying the omentum were cauterized to avoid bleeding. The peritoneal incision was closed with 4-0 Vicryl absorbable continuous sutures. The skin was closed with non-absorbable 5-0 nylon sutures. The mice were given a subcutaneous injection of extended-release buprenorphine (1 mg/kg) and placed in an infant incubator warmed to 27 °C overnight for recovery, then returned to room temperature.

### Tumor cell culture

All tumor cell lines are of female origin, derived from murine ovarian surface epithelium (ID8 and ID8p53^−/−^Brca2^−/−^) or fallopian tube epithelium (KPCA and BPPNM). The original ID8 parental cell line, derived from C57BL/6 mouse (RRID: IMSR_JAX:000664) ovarian surface epithelial cells and transfected with firefly luciferase, was shared directly via MTA with Katherine Roby, who developed the cell line^99^. These ID8 tumor cells labeled with firefly luciferase (ID8-Luc) recapitulate the dissemination of high-grade serous ovarian cancer (HGSOC) upon intraperitoneal injection in immunocompetent mice. Patients with HGSOC frequently have germline mutations in the BRCA and p53 genes^100^. Hence, ID8p53^−/−^Brca2^−/−^ cells labeled with green fluorescent protein (GFP) and firefly luciferase (Luc) were kindly donated by the Khabele lab (WashU) through an MTA with the McNeish lab^101^. The ID8p53^−/−^Brca2^−/−^ GFP Luc cells were maintained in high-glucose DMEM supplemented with 4% FBS, 1% Insulin-Transferrin-Selenium X, and 1% penicillin/streptomycin. The BPPNM (*Trp53*^−/−*R172H*^*Brca1*^−/−^*Pten*^−/−^*Nf1*^−/−^*Myc*^OE^ genotype) and KPCA (*Trp53*^−/−R172H^*Ccne1*^OE^*Akt2*^OE^*KRAS*^G12V^) murine fallopian tube epithelial cells^49^ were obtained by MTA with Robert Weinberg. The cells were maintained in DMEM containing 4% FBS, 2 ng/mL epidermal growth factor, 1% Insulin-Transferrin-Selenium X, and 1% penicillin/streptomycin. Modified BPPNM-Luc cells and sorted KPCA-Luc cells, which express firefly luciferase uniformly, were provided by MTA with Nan Zhang. All cells were sub-cultured twice before each tumor injection. Cells were tested every six months for mycoplasma.

### Tumor expansion in vivo

Mice were injected intraperitoneally (i.p.) with 5 × 10^6^ ID8-Luc, 5 × 10^6^ ID8p53^−/−^Brca2^−/−^ GFP Luc, 1 × 10^6^ KPCA, or 3 × 10^6^ BPPNM tumor cells in 200 µl of sterile Hanks’ Balanced Salt Solution (HBSS) to mimic HGSOC dissemination in the peritoneal cavity. Bioluminescence imaging (BLI) was performed weekly to noninvasively quantify tumor burden in the peritoneal cavity. Mice were treated weekly with i.p. carboplatin at 30 mg/kg (Selleckchem, Cat#S1215) as indicated. Mouse weights were tracked weekly, and mice were sacrificed within a week of the onset of detectable ascites, which served as a predefined humane endpoint in accordance with our IACUC-approved protocol. This tumor limit was not exceeded in any experiment.

### In vivo bioluminescence imaging (BLI)

In vivo BLI was performed on the days indicated using the IVIS50 system (PerkinElmer, Waltham, MA) with Living Image 4.3.1 up to 1 min exposure(s), eight bin, FOV12cm, f/stop1, and open filter. Mice were injected i.p. with D-luciferin (150 mg/kg in PBS; Gold Biotechnology, St. Louis, MO) based on mouse weight and imaged 10 minutes later while under isoflurane anesthesia (2% vaporized in O_2_). Total photon flux (photons/sec) was measured from fixed regions of interest (ROIs) over the peritoneal cavity using Living Image 2.6 (RRID: SCR_014247).

### Ex vivo bioluminescence and stereoscope imaging pipeline for tumor quantification

Immediately before sacrifice, tumor-bearing mice were injected i.p. with D-luciferin (150 mg/kg in PBS; Gold Biotechnology, St. Louis, MO) according to body weight. Mice were sacrificed 10 min post-injection. Peritoneal organs were washed in PBS and pinned in Sylgard plates containing CO_2_-independent media with 5% FBS on ice. When the plates were ready for imaging on a ChemiDoc MP Imaging System (Bio-Rad) or an IVIS50, the media were replaced with fresh CO_2_-independent media containing diluted D-luciferin (1:100) to ensure a uniform substrate concentration across all samples and maintain a stable pH. The plates were warmed at room temperature until the temperature equilibrated for optimal signal detection^102^.

Images generated from the IVIS50 and the corresponding Living Image software were used to quantify tumor burden in each organ. Because organs vary in size, unique regions of interest (ROIs) were created that matched the size of each pinned organ. The total photon flux (photons/sec) was normalized to the specific area of that organ, resulting in photon flux/area measurements for all ex vivo organ analyses.

Bioluminescence and corresponding colorimetric images acquired with the ChemiDoc reader were used to quantify tumor nodule number and size and to determine their anatomical localization within the adipose or peritoneal membrane region. Images were imported into FIJI software 2.14.0/1.54p for analysis. A global scale was applied to all images to quantify nodule size in cm. The auto-threshold features binarized the bioluminescent images to distinguish the nodules from the background, thereby eliminating user bias. The Analyze Particles feature was used to calculate the nodule count, individual nodule area, and cumulative area. Particle masks were overlaid on the corresponding brightfield stereoscope images to confirm localization. For regional quantification, adipose and peritoneal membrane regions were traced as ROIs, and measurements were restricted to each ROI to compute the percent area occupied by tumor nodules in the omentum and mesentery.

### Two-photon microscopy

CD19^Cre/+^TD reporter mice injected with ID8p53^−/−^Brca2^−/−^ GFP Luc cells were anesthetized with a ketamine/xylazine cocktail (0.1 mL/25 g). The omentum was exposed through a 1–2 mm incision in the skin and peritoneum. The mice or fixed omentum were positioned under the customized Leica SP8 2-Photon microscope equipped with a 325/0.95 NA water-dipping objective and a Mai Tai HP DeepSee Laser (Spectra-Physics) tuned to 810 nm. Fluorescence emission was separated by 3 high-efficiency dichroic mirrors (Semrock) cut at 458, 495, and 560 nm and directed to 4 supersensitive external detectors.

### H&E staining

Tissue samples were fixed overnight in 4% paraformaldehyde and transferred to PBS the next day. The samples were processed and stained with hematoxylin and eosin by iHisto (Boston, Massachusetts) and the Washington University Musculoskeletal Histology and Morphometry Core.

### Serum triglyceride measurements

At 5–6 weeks post adipose tissue transplant, serum was collected from whole blood via cheek bleed in BD Microtainer® Blood Collection Tubes with Serum Separator Gel. The serum was allowed to clot at room temperature for approximately 60 min. The clot was removed by centrifugation at 1500 × g for 10 minutes at 4 °C. Following centrifugation, the serum was collected. Serum triglycerides were measured per the manufacturer’s instructions (Wako L-Type TG M), and the remaining samples were stored at −80 °C.

### Glucose and insulin tolerance tests

Serial blood glucose measurements were taken from venous blood (Glucocard, Vital) at 0, 15, 30, 60, and 90 min following intraperitoneal injection of D-glucose (1.5 g/kg) or insulin (0.75 U/kg). Mice were fasted for six hours before the insulin tolerance test or overnight for the glucose tolerance test. Mice were weighed before and after fasting. During fasting, mice were placed in clean cages with a metal grid on the bottom to prevent contact with the floor, thereby limiting coprophagy. To calculate the area of the curve (AOC), the glucose measurements were plotted, and the area under the baseline was subtracted from the area under the curve^103^.

### FABP4 ELISA

Mouse FABP4 was measured in plasma using a commercially available ELISA kit (Abcam, Cat# ab277426).

### In vivo antibody depletion

Anti-Mouse CD4 (clone GK1.5; Leinco Technologies Cat# C1333, RRID: AB_2737452), anti-Mouse CD8b.2 (clone 53-5.8; Leinco Technologies Cat# C2832, RRID: AB_2737471) and anti-Mouse NK1.1 (clone PK136; Leinco Technologies Cat# N123, RRID: AB_2737553) purified functional-grade in vivo gold antibodies were purchased from Leinco. Starting one week before cancer injection, mice were intraperitoneally injected weekly with 250 µg of the CD4 (RRID: AB_2737452) and CD8 (RRID: AB_2737471) depleting antibodies and 100 µg of the NK depleting (RRID: AB_2737553) antibody. Antibody depletion was validated throughout the experiment using flow cytometry of plasma blood samples collected via cheek bleeds.

### Murine Mesentery and Omentum Tissue Collection with Separation of Adipose and Peritoneal Membrane Compartments

Whole mesenteries and omenta from PARC and DARL mice, eight weeks post-surgery, were pinned onto Sylgard dishes and kept hydrated in PBS. The adipose region in PARC mice and the corresponding areas in DARL mice were carefully separated from the peritoneal membrane for each omentum and mesentery using a scalpel under a dissection microscope and placed in RPMI with 5% FBS on ice. Each peritoneal membrane or adipose region collected from every mouse was minced with scissors and digested separately using 1 mg/mL collagenase IV, 0.1 mg/mL DNase, and 0.5% BSA for 30 minutes on a shaker at 150 rpm. The samples were filtered, centrifuged, and resuspended for downstream processing.

### Human omentum tissue collection and processing for single cell sequencing

Benign omentum was collected under IRB approval number 201903051 via the Washington University Ovarian Cancer Tissue Bank. The tissue was de-identified before research use. The specimen was obtained during standard surgical staging for a non-malignant, benign tumor of the ovary. Intra-operatively, the omentum appeared grossly unremarkable, and final pathology confirmed no macroscopic or microscopic omental tumor involvement. Sex was self-reported, and only female tissue was studied, given the sex-specific nature of the disease. Written informed consent was obtained under IRB 201903051, and participants received no compensation. Using a scalpel, the tissue was dissected into omentum, peritoneal membrane, and adipose regions, which were then minced separately and transferred into tubes for enzymatic digestion. Digestion media consisted of RPMI 1640 supplemented with 1 mg/mL collagenase IV and 0.1 mg/mL DNase, and samples were incubated for 45 min at 150 rpm. Digestion was then stopped by adding chilled FACS buffer, and the cell suspension was passed through a 70 µm filter before centrifugation at 500 g for 5 min at 4 °C. Cell pellets were resuspended in FACS buffer containing DAPI, and DAPI-negative (live) cells were sorted. All sorted live cells were then submitted for single-cell RNA sequencing. RNA libraries were prepared using the Chromium Next GEM Single Cell 5’ Kit v2 (10x Genomics, #1000263) according to the manufacturer’s instructions. In brief, cells were encapsulated into droplets, barcoded during reverse transcription, and the emulsions were broken to enable cDNA amplification via PCR, following the V(D)J enrichment protocol (10x Genomics, #1000252). Libraries were prepared according to 10x Genomics guidelines and sequenced on an Illumina NovaSeq 6000 platform, with samples pooled and barcoded to ensure sufficient read depth. Raw sequencing data were processed using Cell Ranger (versions 3.1.0, 6.1.1, or 7.0.1) with the GRCh38 human reference genome to produce gene expression matrices from single-cell 5′ RNA-seq data.

### Spectral flow cytometry

Mesentery and omentum samples were prepared as described (see the above section, Mesentery and Omentum Tissue Collection with Separation of Adipose and Peritoneal Membrane Compartments). Blood samples were collected in tubes containing 15 ml of 500 mM EDTA, and red blood cells were lysed using BD PharmLyse lysis buffer. Blood cells were stained with acridine orange, and leukocytes were counted on a Nexcelom Cellometer Auto X4 cell counter. Quantification of omental and mesothelium-associated cells was based on counts obtained with the Cytek Aurora spectral cytometer. For flow cytometry analysis, cells were washed in PBS, stained with ZombieNIR (cat# 423106), washed again, and resuspended with the antibody mix diluted in flow cytometry buffer (HBSS containing 2% FBS and 5 mM EDTA) containing 20% BD Horizon^TM^ Brilliant Stain Buffer (cat#563794) for 30 min at 4 °C. The flow antibodies used at a concentration of 1:300 were as follows: CD45 (clone 30-F11; BD Biosciences Cat# 748370, RRID:AB_2872789), F4/80 (clone BM8; BioLegend Cat# 123168, RRID: AB_2924461), CD64 (clone X54-5/7.1; BioLegend Cat# 139313, RRID: AB_2563903), ICAM-2 (clone 3C4; BD Biosciences Cat# 740864, RRID:AB_2740516), CD169 (clone 3D6.112; BioLegend Cat# 142416, RRID: AB_2565620), Lyve1 (clone ALY7; Thermo Fisher Scientific Cat# 53-0443-82, RRID: AB_1633415), MHCII (clone M5/114.15.2; BD Biosciences Cat# 750281, RRID: AB_2874472), CD31 (clone MEC13.3; BD Biosciences Cat# 741262, RRID: AB_2870809), CD11b (clone M1/70; BD Biosciences Cat# 564443, RRID: AB_2738811), Ly6g (clone 1A8; BioLegend Cat# 127641, RRID: AB_2565881), Ly6c (clone HK1.4; BioLegend Cat# 128029, RRID: AB_10896061), CD11c (clone N418; Thermo Fisher Scientific Cat# 363-0114-82, RRID: AB_2925254), NK-1.1 (clone S17016D; BioLegend Cat# 156519, RRID: AB_2894654), CD4 (clone GK1.5; BioLegend Cat# 100467, RRID: AB_2734150), CD8a (clone 53-6.7; BioLegend Cat# 100791, RRID: AB_2876397), CD19 (clone eBio1D3 (1D3), Thermo Fisher Scientific Cat# 47-0193-82, RRID: AB_10853189), podoplanin (clone 8.1.1; BioLegend Cat# 127411, RRID: AB_10613294), PDGFR-α or CD140a (clone APA5; BioLegend Cat# 135913, RRID: AB_2715986), and CD9 (clone KMC8; BD Biosciences Cat# 751253, RRID: AB_2875270). Cells were washed twice in flow buffer before data acquisition. Flow cytometry data were acquired on a Cytek Aurora spectral cytometer (5 laser configuration). Flow cytometry data analysis, including unsupervised analysis, was conducted using FlowJo software v10.10 (RRID:SCR_008520).

### Cell hashing and sorting for murine single-cell RNA sequencing

Following digestion of the omentum and mesentery from PARC and DARL mice, the samples were washed, passed through 100 µm filters, and stained with CD45 (clone 30-F11, AB_312980, BioLegend Cat. No. 103115), DAPI, and TER-119 (AB_313706, BioLegend Cat. No. 116205) antibodies in a single antibody mixture for all samples. Each sample was then individually stained with one of 8 hashtags or antibody-derived tags (ADTs). The eight TotalSeq antibodies used were AB_2800693 (BioLegend Cat. No. 155861), AB_2800694 (BioLegend Cat. No. 155863), AB_2800695 (BioLegend Cat. No. 155865), AB_2800696 (BioLegend Cat. No. 155867), AB_2800697 (BioLegend Cat. No. 155869), AB_2819910 (BioLegend Cat. No. 155871), AB_2819911 (BioLegend Cat. No. 155873), and AB_2819912 (BioLegend Cat. No. 155875). These ADTs are conjugated with a DNA oligo containing a barcode to target distinct ubiquitous cellular surface epitopes and were used to identify independent biological replicates for each tissue. The samples were washed three times after hashtag staining to remove unbound antibodies before being pooled into four separate tissue groups, mesenteric adipose region (MAR), mesenteric peritoneal membrane (MPM), omentum adipose region (OAR), and omentum peritoneal membrane (OPM), for both PARC and DARL mice before sorting. Samples were kept on ice until sorted on the FACSAria II with a 100-μm nozzle (Becton Dickinson). To ensure sufficient representation of stromal CD45⁻ populations, CD45^–^ cells were sorted first for each pooled sample. Sorting then continued for CD45⁺ cells until the collection tubes were exhausted. Cells positively stained with DAPI were gated out during cell sorting to remove dead cells. Similarly, cells that were positively stained for TER-119 were excluded to remove red blood cells from our cell quantifications and increase the collection of the desired cells. All viable CD45^-^ and CD45^+^ cells that passed these control gates were collected and submitted to the McDonnell Genome Institute Core for 10X Genomics single-cell RNA sequencing.

### Murine single-cell RNA sequencing

Raw gene expression matrices were processed using the Seurat package in R (V5.1.0). Cells expressing at least 200 genes and genes present in at least three cells were included in the analysis. Antibody capture data from the ADTs were added as a separate assay to facilitate multiplexing analysis, and cell identifiers were renamed to ensure uniqueness across all samples. Single-cell BCR and TCR data were also acquired. The individual datasets were merged into a single Seurat object for integrated analysis. Following sequence alignment, counts of these tags were used to assign a hashtag to each cell. The standard Seurat workflow developed by the original authors of this technology was used to demultiplex hashtag oligos (https://satijalab.org/seurat/articles/hashing_vignette.html).

Cells with no hashtags (negatives) or multiple hashtags (multiplets) were removed from the analysis. Quality control metrics were calculated, and cells with more than 50,000 counts, fewer than 200 features, more than 6000 features, or mitochondrial gene expression exceeding 15% were excluded. Data normalization was performed using the standard log-normalization procedure implemented by the NormalizeData() function with the default Seurat parameters, and 3000 highly variable features were identified via variance-stabilizing transformation. To correct for batch effects, the Harmony integration algorithm was applied, with principal component analysis used for dimensionality reduction. Harmony was performed using default parameters and the IntegrateLayers() workflow in Seurat V5.1.0. The top 30 principal components were used for downstream analysis. Clustering was performed across a range of resolutions to identify cell populations with varying levels of granularity. Ultimately, we proceeded using a clustering resolution of 0.3 to perform cell-type classification on the full dataset. Dimensionality reduction for visualization was achieved using Uniform Manifold Approximation and Projection (UMAP) on the integrated data and 30 dimensions. Metadata was updated to include genotype (PARC or DARL), tissue type (omentum or mesentery), and region (peritoneal membrane or adipose region) based on the sample origin. UMAP plots were generated to visualize the distribution of cells grouped by these categories. The fully processed and integrated dataset was saved for subsequent analyses.

### Capybara analysis and custom reference generation for stromal cell identification

Cells were initially clustered using canonical lineage markers (Fig. 5), including Cd19 and Cd3g (lymphocytes), Csf1r and Itgam (myeloid cells), and Ncr1 (NK cells). Endothelial cells were identified by Pecam1 expression and further subclassified using a curated endothelial reference. To resolve endothelial diversity, we used the Brulois et al^62^. lymph node blood vascular endothelium atlas, which captures the arterial–capillary–venous continuum at single-cell resolution and uniquely includes high endothelial venule subsets and a capillary-resident progenitor population implicated in endothelial renewal (raw counts and metadata: GEO GSE140348). This reference enables higher-resolution endothelial annotation than generic vascular atlases.

The remaining non-endothelial stromal compartment was defined by excluding Ptprc-expressing cells. It was initially organized using canonical stromal markers, including Ly6a, Wt1, Cd9, Dpp4, Pdgfra, and Col1a1, to establish coarse stromal categories before high-resolution annotation. To obtain biologically interpretable stromal sub-classifications beyond de novo clustering alone, we used Capybara (R package v0.0.0.9; https://github.com/morris-lab/Capybara), a reference-mapping framework that compares each query cell to curated reference cell types. Rather than performing tissue-level classification, we constructed custom high-resolution references from selected published datasets using the Capybara function construct.high.res.reference(). Each query cell was assigned quantitative similarity scores that reflect transcriptome-wide resemblance to reference cell types, enabling classification based on the closest-matching population while retaining information about cells with mixed or transitional identities. Each reference was applied independently to preserve lineage- and tissue-specific definitions without imposing a unified taxonomy.

To refine mesothelial states in the murine omentum and mesentery, we used mesothelial cell annotations from the single-cell RNA-sequencing dataset by Jackson-Jones et al.^35^ as a reference. This dataset was chosen because it profiles murine omental tissue and therefore captures the relevant mesothelial and stromal populations present in our system, while explicitly resolving mesothelial cell states from PDGFRa⁺ fibroblast populations. This axis was central to our analysis. In particular, this reference distinguishes Cxcl13⁺ mesothelium associated with fat-associated lymphoid clusters from interferon-responsive, Ifit-high mesothelium, enabling structured annotation of mesothelial heterogeneity. Cell type definitions from Jackson-Jones et al.^35^ are shown in Suppl. Fig. 8.

Annotations for the adipocyte–fibroblast lineage were informed by Rivera-Gonzalez & Butka et al.^57^, which integrates single-cell transcriptomics with lineage tracing across murine skin and inguinal fat depots to define a precursor hierarchy encompassing interstitial progenitors, immature pre-adipocytes, and committed pre-adipocytes (raw counts and metadata: GEO GSE241627). This reference was particularly important because, although DARL mice lack mature adipocytes, adipocyte progenitor populations are retained, necessitating a framework that resolves early adipogenic states.

### 3D vascular image acquisition

Omentum tissues were harvested and rinsed in ice-cold PBS, then fixed in 4% paraformaldehyde (pH 9.0) overnight at 4 °C. Fixed tissues were washed in PBS supplemented with 10 units/mL heparin overnight to reduce background. Samples were subsequently processed for 3D immunofluorescence using the ADAPT-3D pipeline^104^. Briefly, tissues were sequentially incubated in decolorization and partial delipidation buffers overnight, then blocked and stained in ADAPT-3D blocking buffer containing the following primary antibodies overnight at room temperature: anti-CD31 (goat polyclonal, R&D Systems Cat# AF3628, RRID: AB_2161028; 1:200), anti-α-smooth muscle actin (α-SMA) conjugated to Alexa Fluor 488 (clone 1A4, eBioscience Cat# 53-9760-82, RRID: AB_2574461; 1:200), and DAPI (D5942, Sigma-Aldrich; 1:500). Tissues were washed in PBS containing 0.2% Tween-20 and incubated overnight with Alexa Fluor 568-conjugated donkey anti-goat IgG secondary antibody (Invitrogen Cat# A-11057, RRID: AB_2534104; 1:500). Tissues were cleared and mounted in ADAPT-3D refractive index matching solution before imaging. Confocal z-stacks were acquired on an inverted Leica SP8 microscope equipped with seven laser lines and full-spectral hybrid detectors, using a 10× objective (NA 0.4). Three-dimensional reconstruction, visualization, and quantitative analysis were performed in Imaris v10.1.1 (Bitplane).

### 3D vascular image analysis

Vascular architecture was quantified using two complementary image analysis pipelines applied to three-dimensional confocal z-stack images of CD31-immunolabelled omental tissue.

For tumor-bearing tissue, three spatially distinct tumor nodules per mouse were analyzed, and values were averaged to obtain a single per-mouse mean for each metric. For steady-state (tumor-free) omentum, imaging fields were classified a priori by anatomical compartment: milky spots (MS) were defined as discrete, densely vascularized aggregates of immune and stromal cells visible as hypervascular foci, and vascular areas (VA) were defined as the surrounding interstitial vasculature. All MS and VA regions within each mouse were analyzed separately and averaged within compartments to generate per-mouse means, with compartments analyzed independently throughout to account for their distinct baseline vascular architectures. The mouse was used as the experimental unit in all analyses.

For three-dimensional skeleton-based analysis, z-stack images were imported into Imaris (v 9.x, Oxford Instruments). Vessel segmentation was performed in Imaris with automated thresholding, and vascular volume was extracted from the Imaris Surfaces module. Skeletonized networks were generated in Fiji ImageJ and exported as topology (Results) and individual branch geometry (Branch_information) files. Total branch count was defined as the sum of all branches across all detected skeleton components within a field of view and served as a measure of vascular network complexity. Vascular density was calculated as the ratio of CD31-positive vessel volume to the total region-of-interest volume, expressed as a percentage.

For lacunarity analysis, maximum-intensity projection (MIP) images were exported from Imaris and processed in Python 3.12.3 (NumPy 2.4.2, Pillow 12.1.1). Each image was converted to an 8-bit grayscale representation of the CD31 (red) channel and binarized using Otsu’s method. Lacunarity was quantified using the gliding-box approach^105,106^, implemented with non-overlapping boxes of side r tiled across the image. Box sizes were sampled as a geometric series beginning at 2 pixels and increasing approximately 1.5-fold, up to 0.45 × the smaller image dimension. For each box size r, the number of foreground (vessel) pixels in each box (the box mass) was recorded across all non-empty boxes. Lacunarity was calculated as Λ(r) = 1 + σ²(r)/μ²(r), where μ(r) and σ²(r) are the mean and variance of box mass; equivalently, Λ(r) is one plus the squared coefficient of variation of box mass. The lacunarity reported for each image is the mean of Λ(r) across all box sizes. The custom analysis script is available at Zenodo (https://doi.org/10.5281/zenodo.20683730).

### Human single-cell RNA sequencing

Raw gene expression matrices were processed using the Seurat package in R (V5.1.0). Count matrices from each library were read in with Read10X and converted to Seurat objects using CreateSeuratObject (min.cells = 3, min.features = 200). Cells were renamed to ensure unique barcodes across samples by removing trailing “−1” suffixes, and individual Seurat objects were merged into a single object for joint analysis. The percentage of mitochondrial gene expression was calculated using PercentageFeatureSet(pattern = “^MT-“), and cells were filtered based on quality control metrics: only cells with fewer than 25,000 total counts, more than 500 and fewer than 6000 detected features, and less than 10% mitochondrial reads were retained. Filtered data were normalized with NormalizeData, and the top 3000 highly variable genes were identified using FindVariableFeatures. Data were scaled with ScaleData while regressing out nCount_RNA and percent.mt, and principal component analysis was performed with RunPCA. The first 25 principal components, accounting for approximately 88% of variance, were used to construct a shared-nearest-neighbor graph (FindNeighbors, dims = 1:25) and to perform graph-based clustering across resolutions 0.1 to 1.5 (FindClusters). UMAP embeddings were computed on these 25 PCs (RunUMAP, dims = 1:25) to visualize cell populations. Cluster identities and original sample labels (orig.ident) were preserved in metadata for downstream interpretation. UMAP plots were generated for each clustering resolution and grouped by original identity, and the fully processed Seurat object was saved for subsequent analyses.

Publicly available single-cell RNA-sequencing data from a published human omentum atlas^6^ spanning non-metastasized (benign) omentum and omental metastasis from OC were reprocessed and analyzed (GSE286057). This dataset comprises samples from 8 patients with benign omental nodules (P1-P8) and 7 patients with OC omental metastases (P9-P15). For the benign cases (P1-P8), proximal and distal regions from the nodules, as well as normal lesser omentum, were collected. For the OC cases (P9-P15), samples include metastatic tumor nodules and the proximal and distal omental regions relative to the tumor. Additional sample-level details are provided in the original publication^6^. Raw UMI count matrices were loaded using Read10X and converted into Seurat objects using CreateSeuratObject (min.cells = 3, min.features = 200). Samples were merged after appending sample-specific prefixes to cell barcodes. Cells were filtered based on standard quality-control metrics, retaining cells with nCount_RNA < 20,000, 300 <nFeature_RNA < 10,000, and percent.mt < 15. The filtered dataset was normalized using LogNormalize (scale factor = 10,000). Highly variable genes were identified using the VST method (*n* = 3000), and the data were scaled before principal component analysis (PCA). Batch correction across samples was performed using Harmony implemented through the Seurat v5 IntegrateLayers (HarmonyIntegration) workflow, generating an integrated embedding (“integrated.harmony”) from the PCA space. A shared nearest-neighbor graph was constructed using the top 30 principal components, followed by Louvain clustering (FindNeighbors/FindClusters). Clustering was evaluated at multiple resolutions, and the primary analyses used a resolution of 0.4. For visualization, UMAP embeddings were generated using the Harmony-corrected dimensions (dims = 1:30). Cell populations were annotated based on canonical marker genes using feature plots and dot plots. Anatomical region annotations provided by the source study were retained throughout the analysis.

### Statistics and reproducibility

The number of mice used in each experiment and the statistical analyses performed are indicated in the figure legends. Smaller pilot experiments informed the technical feasibility of the experiments and are consistent with sample sizes commonly used in the field. Co-housed age-matched littermate controls were used for all in vivo murine experiments with genetically altered mice. All littermates and genetically altered mice were treated identically and simultaneously throughout all experimental steps to control for confounding variables. Mice in each genotype were randomly assigned to treatment or control groups as needed. Investigators were formally blinded to genotype during data collection when possible. However, certain genotype-associated tissue differences (the presence or absence of mature adipocytes) were macroscopically apparent. Observer bias was minimized by using automated thresholding and particle analysis for tumor-nodule quantification and fixed regions of interest for bioluminescence quantification. Key findings were reproduced across multiple independent experiments and across multiple ovarian cancer models, as indicated in the figure legends. Experimental data were formally assessed for normality and residual distribution, and appropriate parametric or nonparametric tests were used accordingly. The statistical tests used for each experiment are described in the corresponding figure legends. All statistical tests are two-sided. Error bars represent standard error. All statistical calculations were performed with GraphPad Prism 10 (RRID: SCR_002798). *p*-values were adjusted for multiple comparisons as indicated. An adjusted *p-value*  <  0.05 was considered statistically significant. No animals were excluded from any in vivo experiment. Mice that died before the endpoint were included up to the time of death, with later timepoints treated as missing. Exclusion criteria were not pre-established and were based solely on technical validity, independent of experimental outcome. For single-cell RNA-sequencing, standard quality-control filters were applied as described in the Methods.

## Supplementary information

Supplementary Information

Description of Additional Supplementary Files

Supplementary Data 1

Reporting Summery

Transparent Peer Review file

## Source data

Source Data

## Data Availability

Murine single-cell sequencing data generated in this study have been deposited in the Gene Expression Omnibus (GEO) (RRID:SCR_005012) under accession code GSE281706. Human single-cell sequencing data generated in this study have been deposited in the GEO (RRID:SCR_005012) under accession code GSE300038. Publicly available single-cell RNA sequencing datasets can be found in references^6,35,57,62^ and under GEO accession numbers GSM4053741, GSE140348, GSE241627, and GSE286057. Source Data are provided with this paper. All other data supporting the findings of this study are provided in the Source Data file or within the Article and its Supplementary Information. Source data are provided with this paper.

## Code availability

Single-cell RNA-sequencing data were processed using publicly available workflows (Cell Ranger; Seurat v5.1.0 with Harmony integration; Capybara v0.0.0.9) as detailed in the Methods. The custom Python script used to quantify vascular lacunarity is available at Zenodo (https://doi.org/10.5281/zenodo.20683730). No other custom code central to the conclusions was generated.

## References

[1] Sopik, V., Iqbal, J., Rosen, B. & Narod, S. A. Why have ovarian cancer mortality rates declined? Part II. Case-fatality. *Gynecol. Oncol.* **138**, 750–756 (2015). 26080288 10.1016/j.ygyno.2015.06.016

[2] Lengyel, E. Ovarian Cancer Development and Metastasis. *Am. J. Pathol.* **177**, 1053–1064 (2010). 20651229 10.2353/ajpath.2010.100105 PMC2928939

[3] Wilkosz, S. et al. A comparative study of the structure of human and murine greater omentum. *Anat. Embryol. (Berl.)* **209**, 251–261 (2005). 15662530 10.1007/s00429-004-0446-6

[4] Standring, S. *Gray’s**Anatomy: The Anatomical Basis of Clinical Practice*. (Elsevier Limited, 2016).

[5] Pearce, O. M. T. et al. Deconstruction of a Metastatic Tumor Microenvironment Reveals a Common Matrix Response in Human Cancers. *Cancer Discov.* **8**, 304–319 (2018). 29196464 10.1158/2159-8290.CD-17-0284 PMC5837004

[6] Lischetti, U. et al. Ovarian cancer metastasis to the human omentum disrupts organ homeostasis and induces fundamental tissue reprogramming. *Nat. Commun*. **17**, 849 (2025). 10.1038/s41467-025-67557-z PMC12828041 41397972

[7] Arie, A. B., McNally, L., Kapp, D. S. & Teng, N. N. The omentum and omentectomy in epithelial ovarian cancer: a reappraisal: part II-The role of omentectomy in the staging and treatment of apparent early stage epithelial ovarian cancer. *Gynecol. Oncol.* **131**, 784–790 (2013). 24056005 10.1016/j.ygyno.2013.09.013

[8] Hao, Z., Yu, Y. & Yang, S. The impact of omentectomy on cause-specific survival of Stage I-IIIA epithelial ovarian cancer: A PSM-IPTW analysis based on the SEER database. *Front Surg.* **9**, 1052788 (2022). 36644529 10.3389/fsurg.2022.1052788 PMC9836003

[9] Etzerodt, A. et al. Tissue-resident macrophages in omentum promote metastatic spread of ovarian cancer. *J. Exp. Med.* **217**, e20191869 (2020). 10.1084/jem.20191869 PMC7144521 31951251

[10] Zhang, N. et al. LYVE1+ macrophages of murine peritoneal mesothelium promote omentum-independent ovarian tumor growth. *J. Exp. Med.* **218**, e20210924 (2021). 10.1084/jem.20210924 PMC8575007 34714329

[11] Nieman, K. M. et al. Adipocytes promote ovarian cancer metastasis and provide energy for rapid tumor growth. *Nat. Med.* **17**, 1498–1503 (2011). 22037646 10.1038/nm.2492 PMC4157349

[12] Schild, T., Low, V., Blenis, J. & Gomes, A. P. Unique Metabolic Adaptations Dictate Distal Organ-Specific Metastatic Colonization. *Cancer Cell* **33**, 347–354 (2018). 29533780 10.1016/j.ccell.2018.02.001 PMC5889305

[13] Choe, S. S., Huh, J. Y., Hwang, I. J., Kim, J. I. & Kim, J. B. Adipose Tissue Remodeling: Its Role in Energy Metabolism and Metabolic Disorders. *Front Endocrinol. (Lausanne)* **7**, 30 (2016). 27148161 10.3389/fendo.2016.00030 PMC4829583

[14] Bayraktar, E., Chen, S., Corvigno, S., Liu, J. & Sood, A. K. Ovarian cancer metastasis: Looking beyond the surface. *Cancer Cell* **42**, 1631–1636 (2024). 10.1016/j.ccell.2024.08.016 39270645

[15] Duong, M. N. et al. The fat and the bad: Mature adipocytes, key actors in tumor progression and resistance. *Oncotarget* **8**, 57622–57641 (2017). 28915700 10.18632/oncotarget.18038 PMC5593672

[16] Lengyel, E., Makowski, L., DiGiovanni, J. & Kolonin, M. G. Cancer as a Matter of Fat: The Crosstalk between Adipose Tissue and Tumors. *Trends Cancer* **4**, 374–384 (2018). 29709261 10.1016/j.trecan.2018.03.004 PMC5932630

[17] Attané, C. & Muller, C. Drilling for oil: tumor-surrounding adipocytes fueling cancer. *Trends Cancer* **6**, 593–604 (2020). 32610069 10.1016/j.trecan.2020.03.001

[18] Xiang, F. et al. Omental adipocytes enhance the invasiveness of gastric cancer cells by oleic acid-induced activation of the PI3K-Akt signaling pathway. *Int J. Biochem Cell Biol.* **84**, 14–21 (2017). 27956048 10.1016/j.biocel.2016.12.002

[19] Clement, E. et al. Adipocyte extracellular vesicles carry enzymes and fatty acids that stimulate mitochondrial metabolism and remodeling in tumor cells. *Embo j.* **39**, e102525 (2020). 31919869 10.15252/embj.2019102525 PMC6996584

[20] Coe, N. R. & Bernlohr, D. A. Physiological properties and functions of intracellular fatty acid-binding proteins. *Biochimica et Biophysica Acta (BBA) - Lipids Lipid Metab.* **1391**, 287–306 (1998). 10.1016/s0005-2760(97)00205-1 9555061

[21] Li, Z., Fang, X. & Wang, S. Omentum provides a special cell microenvironment for ovarian cancer. *Cancer Rep. (Hoboken)* **6**, e1858 (2023). 37605299 10.1002/cnr2.1858 PMC10598246

[22] Lee, D. Y. & Lee, T. S. Associations between metabolic syndrome and gynecologic cancer. *Obstet. Gynecol. Sci.* **63**, 215–224 (2020). 32489965 10.5468/ogs.2020.63.3.215 PMC7231948

[23] Duarte Mendes, A. et al. Adipocyte microenvironment in ovarian cancer: a critical contributor? *Int. J. Mol. Sci.* **24**, 16589 (2023). 10.3390/ijms242316589 PMC10706733 38068912

[24] Purcell, S. A., Elliott, S. A., Kroenke, C. H., Sawyer, M. B. & Prado, C. M. Impact of body weight and body composition on ovarian cancer prognosis. *Curr. Oncol. Rep.* **18**, 8 (2016). 26769113 10.1007/s11912-015-0488-3

[25] Yang, J. et al. Adipocytes promote ovarian cancer chemoresistance. *Sci. Rep.* **9**, 13316 (2019). 31527632 10.1038/s41598-019-49649-1 PMC6746782

[26] Zhou, S., Wang, R. & Xiao, H. Adipocytes induce the resistance of ovarian cancer to carboplatin through ANGPTL4. *Oncol. Rep.* **44**, 927–938 (2020). 32705217 10.3892/or.2020.7647 PMC7388553

[27] Previs, R. A. et al. Obesity is associated with worse overall survival in women with low-grade papillary serous epithelial ovarian cancer. *Int J. Gynecol. Cancer* **24**, 670–675 (2014). 24614825 10.1097/IGC.0000000000000109 PMC4545297

[28] Tworoger, S. S. & Huang, T. in *Obesity and Cancer* (eds Tobias Pischon & Katharina Nimptsch) 155–176 (Springer International Publishing, 2016).

[29] Backes, F. J. et al. The impact of body weight on ovarian cancer outcomes. *Int J. Gynecol. Cancer* **21**, 1601–1605 (2011). 21997171 10.1097/IGC.0b013e31822d2aa3 PMC4175395

[30] Münstedt, K., Wagner, M., Kullmer, U., Hackethal, A. & Franke, F. E. Influence of body mass index on prognosis in gynecological malignancies. *Cancer Causes Control* **19**, 909–916 (2008). 18392944 10.1007/s10552-008-9152-7

[31] Protani, M. M., Nagle, C. M. & Webb, P. M. Obesity and ovarian cancer survival: a systematic review and meta-analysis. *Cancer Prev. Res (Philos.)* **5**, 901–910 (2012). 10.1158/1940-6207.CAPR-12-0048 22609763

[32] Bae, H. S. et al. Obesity and epithelial ovarian cancer survival: a systematic review and meta-analysis. *J. Ovarian Res* **7**, 41 (2014). 24834130 10.1186/1757-2215-7-41 PMC4022349

[33] Tyler, C. P. et al. The effect of body mass index and weight change on epithelial ovarian cancer survival in younger women: a long-term follow-up study. *J. Women’s Health* **21**, 865–871 (2012). 10.1089/jwh.2012.3487 22663301

[34] Olsen, C. M. et al. Obesity and risk of ovarian cancer subtypes: evidence from the Ovarian Cancer Association Consortium. *Endocr. Relat. Cancer* **20**, 251–262 (2013). 23404857 10.1530/ERC-12-0395 PMC3857135

[35] Jackson-Jones, L. H. et al. Stromal cells covering omental fat-associated lymphoid clusters trigger formation of neutrophil aggregates to capture peritoneal contaminants. *Immunity* **52**, 700–715.e6 (2020). 32294409 10.1016/j.immuni.2020.03.011 PMC7156918

[36] Pellicciaro, M., Vella, I., Lanzoni, G., Tisone, G. & Ricordi, C. The greater omentum as a site for pancreatic islet transplantation. *CellR4 Repair Replace Regen. Reprogram* **5**, e2410 (2017). 33834082 PMC8025931

[37] Buechler, M. B. et al. A stromal niche defined by expression of the transcription factor WT1 mediates programming and homeostasis of cavity-resident macrophages. *Immunity* **51**, 119–130.e5 (2019). 31231034 10.1016/j.immuni.2019.05.010 PMC6814267

[38] Trim, W. V. & Lynch, L. Immune and non-immune functions of adipose tissue leukocytes. *Nat. Rev. Immunol.* **22**, 371–386 (2022). 34741167 10.1038/s41577-021-00635-7

[39] Rangel-Moreno, J. et al. Omental milky spots develop in the absence of lymphoid tissue-inducer cells and support B and T cell responses to peritoneal antigens. *Immunity* **30**, 731–743 (2009). 19427241 10.1016/j.immuni.2009.03.014 PMC2754314

[40] Bénézech, C. et al. Inflammation-induced formation of fat-associated lymphoid clusters. *Nat. Immunol.* **16**, 819–828 (2015). 26147686 10.1038/ni.3215 PMC4512620

[41] Zou, W. et al. Congenital lipodystrophy induces severe osteosclerosis. *PLoS Genet* **15**, e1008244 (2019). 31233501 10.1371/journal.pgen.1008244 PMC6611650

[42] Wang, Z. V., Deng, Y., Wang, Q. A., Sun, K. & Scherer, P. E. Identification and characterization of a promoter cassette conferring adipocyte-specific gene expression. *Endocrinology* **151**, 2933–2939 (2010). 20363877 10.1210/en.2010-0136 PMC2875825

[43] Straub, L. G. & Scherer, P. E. Metabolic messengers: adiponectin. *Nat. Metab.* **1**, 334–339 (2019). 32661510 10.1038/s42255-019-0041-z PMC7357716

[44] Jeffery, E. et al. Characterization of Cre recombinase models for the study of adipose tissue. *Adipocyte* **3**, 206–211 (2014). 25068087 10.4161/adip.29674 PMC4110097

[45] Chait, A. & den Hartigh, L. J. Adipose tissue distribution, inflammation and its metabolic consequences, including diabetes and cardiovascular disease. *Front. Cardiovasc. Med.* **7**, 22 (2020). 10.3389/fcvm.2020.00022 PMC7052117 32158768

[46] Wu, X. et al. Contribution of adipose-derived factor D/adipsin to complement alternative pathway activation: lessons from lipodystrophy. *J. Immunol.* **200**, 2786–2797 (2018). 29531168 10.4049/jimmunol.1701668 PMC5893424

[47] Maeda, N. et al. Diet-induced insulin resistance in mice lacking adiponectin/ACRP30. *Nat. Med.* **8**, 731–737 (2002). 12068289 10.1038/nm724

[48] Zhang, X. et al. A bone-specific adipogenesis pathway in fat-free mice defines key origins and adaptations of bone marrow adipocytes with age and disease. *eLife* **10**, e66275 (2021). 34378533 10.7554/eLife.66275 PMC8412938

[49] Iyer, S. et al. Genetically defined syngeneic mouse models of ovarian cancer as tools for the discovery of combination immunotherapy. *Cancer Discov.* **11**, 384–407 (2021). 33158843 10.1158/2159-8290.CD-20-0818 PMC8344888

[50] Liu, M. et al. Circulating tregs accumulate in omental tumors and acquire adipose-resident features. *Cancer Immunol. Res* **10**, 641–655 (2022). 35263766 10.1158/2326-6066.CIR-21-0880 PMC9064962

[51] Falandry, C. et al. Efficacy and safety of first-line single-agent carboplatin vs carboplatin plus paclitaxel for vulnerable older adult women with ovarian cancer: a GINECO/GCIG randomized clinical trial. *JAMA Oncol.* **7**, 853–861 (2021). 33885718 10.1001/jamaoncol.2021.0696 PMC8063137

[52] Stoeckius, M. et al. Cell Hashing with barcoded antibodies enables multiplexing and doublet detection for single cell genomics. *Genome Biol.* **19**, 224 (2018). 30567574 10.1186/s13059-018-1603-1 PMC6300015

[53] Emont, M. P. et al. A single-cell atlas of human and mouse white adipose tissue. *Nature* **603**, 926–933 (2022). 35296864 10.1038/s41586-022-04518-2 PMC9504827

[54] Kong, W. et al. Capybara: A computational tool to measure cell identity and fate transitions. *Cell Stem Cell* **29**, 635–649.e11 (2022). 35354062 10.1016/j.stem.2022.03.001 PMC9040453

[55] Bajwa, P. et al. Cancer-associated mesothelial cell-derived ANGPTL4 and STC1 promote the early steps of ovarian cancer metastasis. *JCI Insight* **8**, e163019 (2023). 10.1172/jci.insight.163019 PMC10070116 36795484

[56] Waters, J. A. et al. Omental preadipocytes stimulate matrix remodeling and IGF signaling to support ovarian cancer metastasis. *Cancer Res.* **84**, 2073–2089 (2024). 38635891 10.1158/0008-5472.CAN-23-2613 PMC11217736

[57] Rivera-Gonzalez, G. C. et al. Comparative single-cell lineage tracing identifies distinct adipocyte precursor dynamics in skin and inguinal fat. *Cell Stem Cell* **32**, 1267–1284.e8 (2025). 40744015 10.1016/j.stem.2025.07.004 PMC12338953

[58] Lee, M.-R. et al. The adipokine Retnla modulates cholesterol homeostasis in hyperlipidemic mice. *Nat. Commun.* **5**, 4410 (2014). 25022542 10.1038/ncomms5410

[59] White, R. T. et al. Human adipsin is identical to complement factor D and is expressed at high levels in adipose tissue. *J. Biol. Chem.* **267**, 9210–9213 (1992). 1374388

[60] Mukherjee, A. et al. Adipocyte-Induced FABP4 Expression in Ovarian Cancer Cells Promotes Metastasis and Mediates Carboplatin Resistance. *Cancer Res* **80**, 1748–1761 (2020). 32054768 10.1158/0008-5472.CAN-19-1999 PMC10656748

[61] Gharpure, K. M. et al. FABP4 as a key determinant of metastatic potential of ovarian cancer. *Nat. Commun.* **9**, 2923 (2018). 30050129 10.1038/s41467-018-04987-y PMC6062524

[62] Brulois, K. et al. A molecular map of murine lymph node blood vascular endothelium at single cell resolution. *Nat. Commun.* **11**, 3798 (2020). 32732867 10.1038/s41467-020-17291-5 PMC7393069

[63] Inouye, K. E. et al. Endothelial-derived FABP4 constitutes the majority of basal circulating hormone and regulates lipolysis-driven insulin secretion. *JCI Insight* **8**, e164642 (2023). 10.1172/jci.insight.164642 PMC10443803 37279064

[64] Tang, Y., Harrington, A., Yang, X., Friesel, R. E. & Liaw, L. The contribution of the Tie2+ lineage to primitive and definitive hematopoietic cells. *Genesis* **48**, 563–567 (2010). 20645309 10.1002/dvg.20654 PMC2944906

[65] Lee, W. et al. Neutrophils facilitate ovarian cancer premetastatic niche formation in the omentum. *J. Exp. Med.* **216**, 176–194 (2019). 30567719 10.1084/jem.20181170 PMC6314534

[66] Ko, S. Y., Ladanyi, A., Lengyel, E. & Naora, H. Expression of the Homeobox Gene HOXA9 in ovarian cancer induces peritoneal macrophages to acquire an M2 tumor-promoting phenotype. *Am. J. Pathol.* **184**, 271–281 (2014). 24332016 10.1016/j.ajpath.2013.09.017 PMC3873497

[67] Gerber, S. A. et al. Preferential attachment of peritoneal tumor metastases to omental immune aggregates and possible role of a unique vascular microenvironment in metastatic survival and growth. *Am. J. Pathol.* **169**, 1739–1752 (2006). 17071597 10.2353/ajpath.2006.051222 PMC1780209

[68] Cai, J. et al. Fibroblasts in omentum activated by tumor cells promote ovarian cancer growth, adhesion and invasiveness. *Carcinogenesis* **33**, 20–29 (2012). 22021907 10.1093/carcin/bgr230

[69] Clark, R. et al. Milky spots promote ovarian cancer metastatic colonization of peritoneal adipose in experimental models. *Am. J. Pathol.* **183**, 576–591 (2013). 23885715 10.1016/j.ajpath.2013.04.023 PMC3730760

[70] Hagiwara, A. et al. Milky spots as the implantation site for malignant cells in peritoneal dissemination in mice. *Cancer Res* **53**, 687–692 (1993). 8425204

[71] Krist, L. F. et al. Milky spots in the greater omentum are predominant sites of local tumour cell proliferation and accumulation in the peritoneal cavity. *Cancer Immunol. Immunother.* **47**, 205–212 (1998). 9875673 10.1007/s002620050522 PMC11037365

[72] Tsujimoto, H. et al. Role of milky spots as selective implantation sites for malignant cells in peritoneal dissemination in mice. *J. Cancer Res Clin. Oncol.* **122**, 590–595 (1996). 8879256 10.1007/BF01221190 PMC12200709

[73] Sorensen, E. W. et al. Omental immune aggregates and tumor metastasis within the peritoneal cavity. *Immunol. Res* **45**, 185–194 (2009). 19253004 10.1007/s12026-009-8100-2 PMC2891204

[74] Miranda, F. et al. Salt-Inducible Kinase 2 couples ovarian cancer cell metabolism with survival at the adipocyte-rich metastatic niche. *Cancer Cell* **30**, 273–289 (2016). 27478041 10.1016/j.ccell.2016.06.020

[75] Nieman, K. M., Romero, I. L., Van Houten, B. & Lengyel, E. Adipose tissue and adipocytes support tumorigenesis and metastasis. *Biochimica et Biophysica Acta (BBA) - Mol. Cell Biol. Lipids* **1831**, 1533–1541 (2013). 10.1016/j.bbalip.2013.02.010 PMC3742583 23500888

[76] Rupert, J., Cao, P. H. A., Frigo, D. E. & Kolonin, M. G. Lipids grease the chain of cancer progression. *Trends Cancer* **12**, 235–247 (2026). 41535142 10.1016/j.trecan.2025.11.012 PMC13250982

[77] Cook, D. P. et al. Comparative analysis of syngeneic mouse models of high-grade serous ovarian cancer. *Commun. Biol.* **6**, 1152 (2023). 37957414 10.1038/s42003-023-05529-z PMC10643551

[78] Shankaran, V. et al. IFNgamma and lymphocytes prevent primary tumour development and shape tumour immunogenicity. *Nature* **410**, 1107–1111 (2001). 11323675 10.1038/35074122

[79] Peng, Z., Li, M., Li, H. & Gao, Q. PD-1/PD-L1 immune checkpoint blockade in ovarian cancer: Dilemmas and opportunities. *Drug Discov. Today* **28**, 103666 (2023). 37302543 10.1016/j.drudis.2023.103666

[80] Wu, B. et al. Adipose PD-L1 Modulates PD-1/PD-L1 Checkpoint Blockade Immunotherapy Efficacy in Breast Cancer. *Oncoimmunology* **7**, e1500107 (2018). 30393583 10.1080/2162402X.2018.1500107 PMC6209395

[81] Hotamisligil, G. S. & Bernlohr, D. A. Metabolic functions of FABPs—mechanisms and therapeutic implications. *Nat. Rev. Endocrinol.* **11**, 592–605 (2015). 26260145 10.1038/nrendo.2015.122 PMC4578711

[82] Elmasri, H. et al. Fatty acid binding protein 4 is a target of VEGF and a regulator of cell proliferation in endothelial cells. *FASEB J.* **23**, 3865–3873 (2009). 19625659 10.1096/fj.09-134882 PMC2775007

[83] Yorek, M. et al. FABP4-mediated lipid accumulation and lipolysis in tumor-associated macrophages promote breast cancer metastasis. *Elife* **13**, RP101221 (2024). 10.7554/eLife.101221 PMC11548877 39513934

[84] Distel, R. J., Ro, H.-S., Rosen, B. S., Groves, D. L. & Spiegelman, B. M. Nucleoprotein complexes that regulate gene expression in adipocyte differentiation: direct participation of c-fos. *Cell* **49**, 835–844 (1987). 3555845 10.1016/0092-8674(87)90621-0

[85] Cao, H. et al. Adipocyte lipid chaperone AP2 is a secreted adipokine regulating hepatic glucose production. *Cell Metab.* **17**, 768–778 (2013). 23663740 10.1016/j.cmet.2013.04.012 PMC3755450

[86] Xu, A. et al. Adipocyte fatty acid-binding protein is a plasma biomarker closely associated with obesity and metabolic syndrome. *Clin. Chem.* **52**, 405–413 (2006). 16423904 10.1373/clinchem.2005.062463

[87] Ladanyi, A. et al. Adipocyte-induced CD36 expression drives ovarian cancer progression and metastasis. *Oncogene* **37**, 2285–2301 (2018). 29398710 10.1038/s41388-017-0093-z PMC5920730

[88] Febbraio, M. et al. Targeted disruption of the class B scavenger receptor CD36 protects against atherosclerotic lesion development in mice. *J. Clin. Invest* **105**, 1049–1056 (2000). 10772649 10.1172/JCI9259 PMC300837

[89] Son, N. H. et al. Endothelial cell CD36 optimizes tissue fatty acid uptake. *J. Clin. Invest* **128**, 4329–4342 (2018). 30047927 10.1172/JCI99315 PMC6159965

[90] Peche, V. S. et al. Endothelial cell CD36 regulates membrane ceramide formation, exosome fatty acid transfer and circulating fatty acid levels. *Nat. Commun.* **14**, 4029 (2023). 37419919 10.1038/s41467-023-39752-3 PMC10329018

[91] Daquinag, A. C. et al. Fatty acid mobilization from adipose tissue is mediated by CD36 posttranslational modifications and intracellular trafficking. *JCI Insight* **6**, e147057 (2021). 10.1172/jci.insight.147057 PMC8492349 34314388

[92] Huang, L. et al. SR-B1 drives endothelial cell LDL transcytosis via DOCK4 to promote atherosclerosis. *Nature* **569**, 565–569 (2019). 31019307 10.1038/s41586-019-1140-4 PMC6631346

[93] Le Master, E. et al. Caveolin-1 is a primary determinant of endothelial stiffening associated with dyslipidemia, disturbed flow, and ageing. *Sci. Rep.* **12**, 17822 (2022). 36280774 10.1038/s41598-022-20713-7 PMC9592578

[94] Bauer, P. M. et al. Endothelial-specific expression of caveolin-1 impairs microvascular permeability and angiogenesis. *Proc. Natl. Acad. Sci.* **102**, 204–209 (2005). 15615855 10.1073/pnas.0406092102 PMC544041

[95] Briand, N., Le Lay, S., Sessa, W. C., Ferré, P. & Dugail, I. Distinct roles of endothelial and adipocyte caveolin-1 in macrophage infiltration and adipose tissue metabolic activity. *Diabetes* **60**, 448–453 (2011). 21270257 10.2337/db10-0856 PMC3028344

[96] Harjes, U. et al. Antiangiogenic and tumour inhibitory effects of downregulating tumour endothelial FABP4. *Oncogene* **36**, 912–921 (2017). 27568980 10.1038/onc.2016.256 PMC5318662

[97] Abbott, K. L. et al. Nutrient requirements of organ-specific metastasis in breast cancer. *Nature* **649**, 1292–1301 (2026). 10.1038/s41586-025-09898-9 PMC12851942 41501456

[98] Gavrilova, O. et al. Surgical implantation of adipose tissue reverses diabetes in lipoatrophic mice. *J. Clin. Investig.* **105**, 271–278 (2000). 10675352 10.1172/JCI7901 PMC377444

[99] Roby, K. F. et al. Development of a syngeneic mouse model for events related to ovarian cancer. *Carcinogenesis* **21**, 585–591 (2000). 10753190 10.1093/carcin/21.4.585

[100] Vázquez-García, I. et al. Ovarian cancer mutational processes drive site-specific immune evasion. *Nature* **612**, 778–786 (2022). 36517593 10.1038/s41586-022-05496-1 PMC9771812

[101] Walton, J. et al. CRISPR/Cas9-Mediated Trp53 and Brca2 Knockout to Generate Improved Murine Models of Ovarian High-Grade Serous Carcinoma. *Cancer Res* **76**, 6118–6129 (2016). 27530326 10.1158/0008-5472.CAN-16-1272 PMC5802386

[102] Ueda, I., Shinoda, F. & Kamaya, H. Temperature-dependent effects of high pressure on the bioluminescence of firefly luciferase. *Biophys. J.* **66**, 2107–2110 (1994). 8075344 10.1016/S0006-3495(94)81005-7 PMC1275936

[103] Virtue, S. & Vidal-Puig, A. GTTs and ITTs in mice: simple tests, complex answers. *Nat. Metab.* **3**, 883–886 (2021). 34117483 10.1038/s42255-021-00414-7

[104] Lee, D. D. et al. ADAPT-3D:accelerated deep adaptable processing of tissue for 3-dimensional fluorescence tissue imaging for research and clinical settings. *Sci. Rep.* **15**, 31841 (2025). 40883418 10.1038/s41598-025-16766-z PMC12397274

[105] Allain, C. & Cloitre, M. Characterizing the lacunarity of random and deterministic fractal sets. *Phys. Rev. A* **44**, 3552–3558 (1991). 9906372 10.1103/physreva.44.3552

[106] Plotnick, R. E., Gardner, R. H. & O’Neill, R. V. Lacunarity indices as measures of landscape texture. *Landsc. Ecol.* **8**, 201–211 (1993).

