# Supplementary material for: Peritoneal ovarian cancer growth in the omentum proceeds independently of mature adipocytes
Source: Nat Commun. 2026 Aug 12;17:9673. doi: 10.1038/s41467-026-76471-x (PMC13558732; doi:10.1038/s41467-026-76471-x)
Supplement: Supplementary file 1 [file 41467_2026_76471_MOESM1_ESM.pdf]

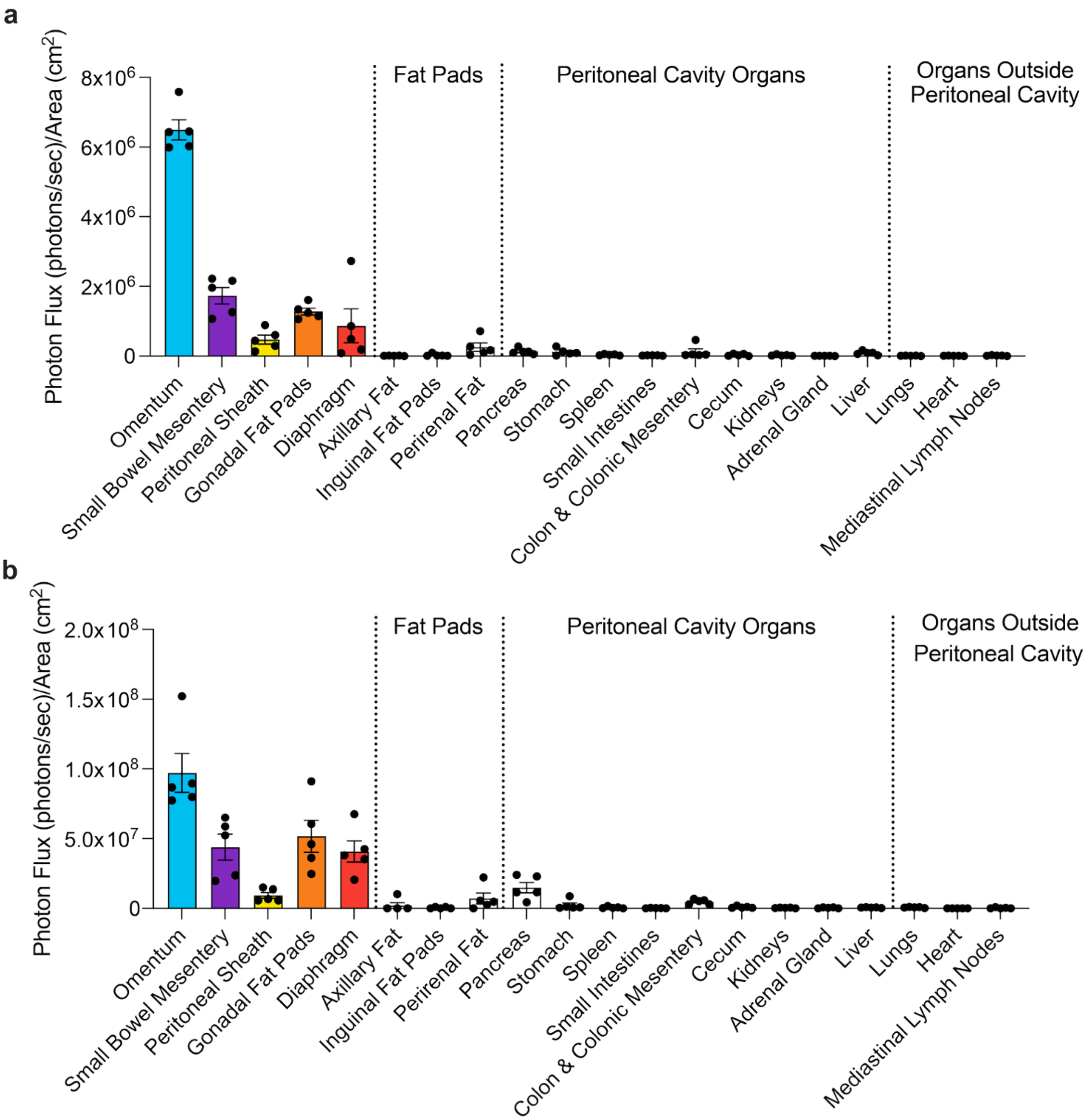

Supplementary Figure 1. OC cells exhibit omental tropism following intraperitoneal injection.

a-b, Distribution of tumor burden across organs in C57BL/6J WT mice (n=5) at **a**. one-week (Kruskal–Wallis, $p < 0.0001$) and **b**. six weeks (Kruskal–Wallis, $p < 0.0001$) post i.p. injection of 5×10^6 ID8p53$^{-/-}$Brca2$^{-/-}$ GFP Luc tumor cells. Tumor burden was quantified as photon flux per organ area from ex vivo bioluminescence images of pinned organs. Organs are grouped by anatomical location, including fat pads, organs within the peritoneal cavity, and organs outside the peritoneal cavity. Statistical comparisons were performed using a Kruskal–Wallis test followed by Dunn's multiple comparisons test. Significant pairwise comparisons (omentum versus organ) were, for a (one week): adrenal gland p = 0.0004, axillary fat p = 0.0013, heart p = 0.0017, lungs p = 0.0020, and mediastinal lymph nodes p = 0.021; and for b (six weeks): heart p = 0.0004, small intestines p = 0.0030, axillary fat p = 0.0058, mediastinal lymph nodes p = 0.023, and adrenal gland p = 0.036. Throughout, n indicates the number of individual mice, each an independent biological replicate.

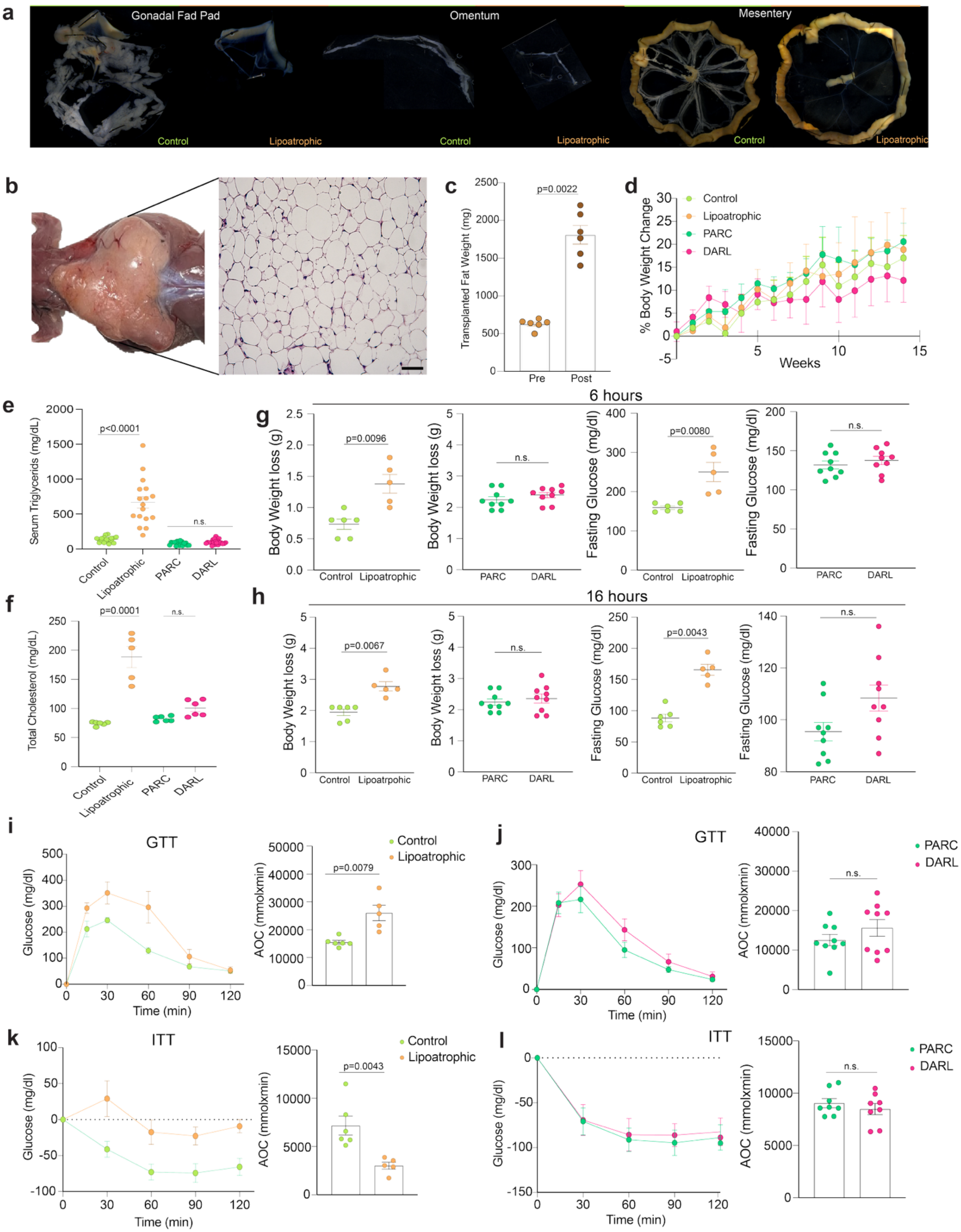
a
Gonadal Fad Pad
Omentum
Mesentery
Control
Lipoatrophic
b
c
p=0.0022
Transplanted Fat Weight (mg)
Pre
Post
d
Control
Lipoatrophic
PARC
DARL
% Body Weight Change
Weeks
e
p<0.0001
n.s.
Serum Triglycerids (mg/dL)
f
p=0.0001
Total Cholesterol (mg/dL)
6 hours
g
p=0.0096
p=0.0080
Body Weight loss (g)
Fasting Glucose (mg/dl)
16 hours
h
p=0.0067
p=0.0043
Lipoatrpohic
i
GTT
Glucose (mg/dl)
Time (min)
AOC (mmolxmin)
p=0.0079
j
k
ITT
p=0.0043
l

Supplementary Figure 2. Fat transplantation restores metabolic parameters in lipoatrophic mice.

a. Representative stereoscope images of peritoneal fat regions in control and lipoatrophic mice, including the gonadal fat pads, omentum, and mesentery. **b**. Stereoscope image and corresponding H&E staining of a transplanted fat in a lipoatrophic mouse 12 weeks after fat transplant surgery. Scale bar, 50 µm. **c**. Weight of transplanted fat pads pre- and 12 weeks post-transplant in lipoatrophic mice. Two-tailed Mann–Whitney test ($p = 0.0022$). **d**. Percent body weight change over time in control ($n = 15$) and lipoatrophic ($n = 6$) mice without surgery and corresponding PARC ($n = 9$) and DARL ($n = 8$) littermates that received fat transplantation. Data are normalized to body weight at the time of surgery. Area under the curve (AUC) was calculated per mouse and compared across genotypes using a Kruskal–Wallis test (n.s.). **e**. Serum triglyceride measurements in control (n=18) and lipoatrophic (n=17) mice pre-surgery and in PARC (n=17) and DARL (n=20) mice post-fat transplantation. Normality was determined with the Shapiro–Wilk test. One-way ANOVA ($p < 0.0001$) and post-hoc comparisons using Tukey's HSD, $p < 0.0001$. **f.** Serum cholesterol levels in control (n=6) and lipoatrophic (n=5) mice pre-surgery and PARC (n=6) and DARL (n=6) mice post-fat transplantation. Kruskal–Wallis test ($p = 0.0002$) with Dunn's multiple comparisons test (Control vs Lipoatrophic $p = 0.0001$, Control vs DARL $p = 0.016$, Lipoatrophic vs PARC $p = 0.026$). **g–h**. Body weight loss and fasting glucose levels following **g**. 6 h or **h.**16 h fasting in control (n= 5) and lipoatrophic mice (n= 5) before surgery and corresponding PARC (n= 9) and DARL (n= 9) mice after fat transplantation. Two-tailed Mann–Whitney test. Glucose tolerance test in **i**. control (n=8) and lipoatrophic (n=8) mice before surgery, and **j**. in PARC (n=8) and DARL (n=8) mice post fat transplant surgery. AOC (area of the curve) was calculated and compared by a two-tailed Mann–Whitney test. Insulin tolerance test in **k**. control (n=8) and lipoatrophic (n=8) mice before surgery, and **l**. PARC (n=8) and DARL (n=8) mice post fat transplant surgery. AOC was calculated and compared by a two-tailed Mann–Whitney test. Throughout, n indicates the number of individual mice, each an independent biological replicate.

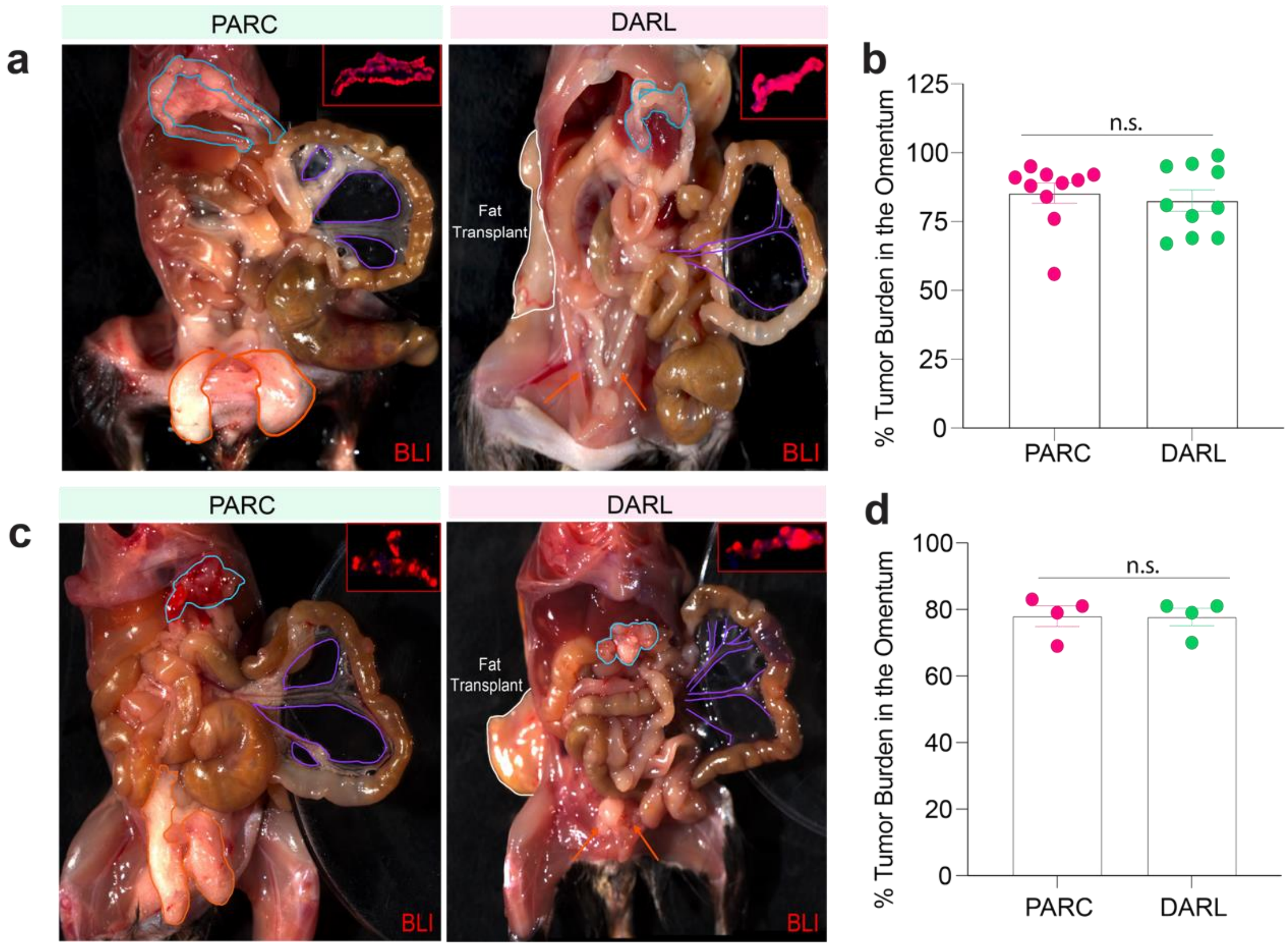

Supplementary Figure 3. Metastasis to the omentum is preserved across OC tumor models in the absence of mature adipocytes.

a–d. Representative images and ex vivo tumor quantification in the omentum of tumor-injected PARC and DARL mice using two OC models. Mice were injected intraperitoneally with $1x10^6$ KPCA-luc tumor cells **a,b** or $3x10^6$ BPPNM-luc tumor cells **c,d**. KPCA-injected mice were harvested at day 7, and BPPNM-injected mice at day 21. Gross images of the peritoneal cavity showing tumor encapsulation of the omentum in PARC and DARL mice. Blue outlines indicate the omental region. Purple outlines indicate the mesenteric region. Orange outlines indicate gonadal fat pads, and orange arrows indicate gonadal fat regions in DARL mice. White outlines indicate transplanted fat depots in DARL mice. Insets (top right) show ex vivo BLI of the omentum with tumor nodules shown in red. **b**,**d** Quantification of percent tumor burden in the omentum based on ex vivo BLI of the omentum. Two-tailed Mann–Whitney test, n.s. Throughout, n indicates the number of individual mice, each an independent biological replicate.

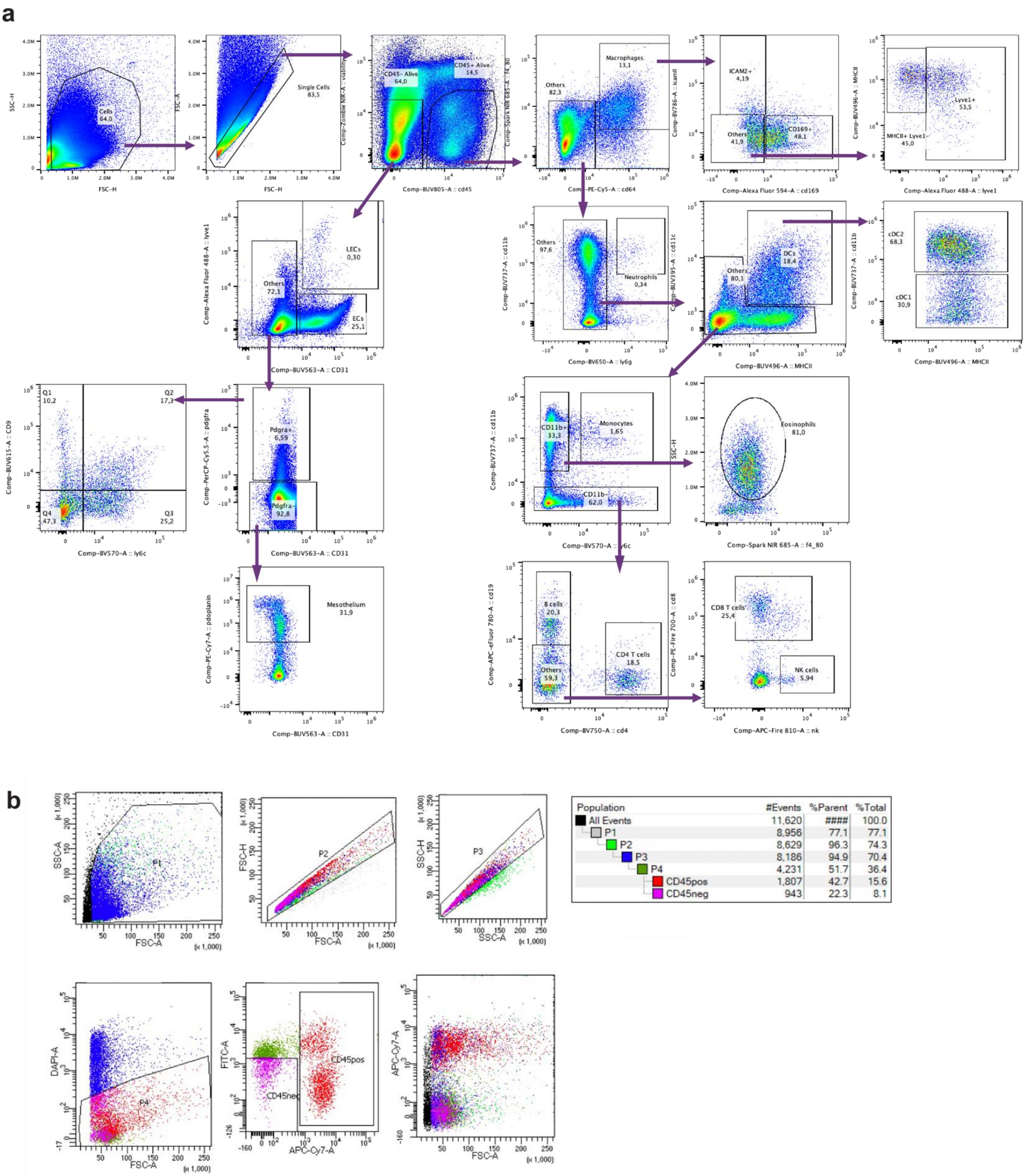

Supplementary Figure 4. Flow cytometry gating and cell sorting strategy.

a. Flow cytometry gating to classify CD45$^+$ immune cell populations in PARC (*n*=4) and DARL (*n*=4) mice at steady state. **b**. Fluorescence-activated cell sorting (FACS) strategy to isolate both CD45$^+$ and CD45$^-$ cell populations from omentum and mesentery compartments (peritoneal membrane or adipose region) for single-cell RNA sequencing. Throughout, n indicates the number of individual mice, each an independent biological replicate.

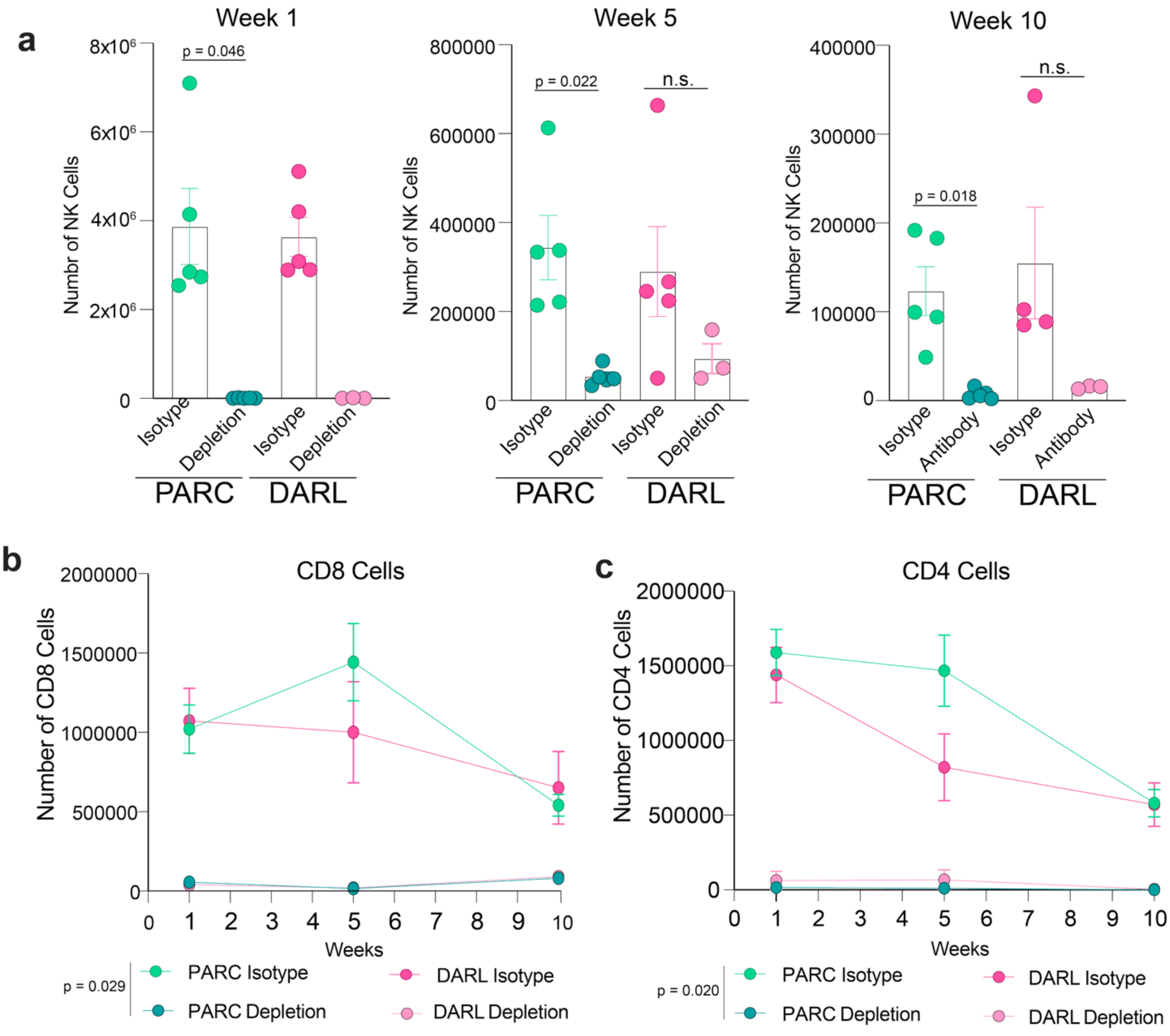

Supplementary Figure 5. Validation of in vivo immune cell depletion.

Cell numbers over time in PARC and DARL mice treated weekly with CD8, CD4, and NK1.1 depleting antibodies (PARC depletion, *n*=5, DARL depletion, *n*=3) or isotype controls (PARC Isotype, *n*=5, DARL isotype, *n*=5) starting one week before i.p. injection of $5x10^6$ ID8-Luc tumor cells. **a**. Natural Killer (NK) cells, Kruskal–Wallis (p = 0.004 at week 1, p = 0.015 at week 5, p = 0.006 at week 10) followed by Dunn's multiple comparison tests. **b**. $CD8^+$ cells, Area under the curve (AUC) values were calculated for each mouse and compared between groups using Kruskal–Wallis test (p = 0.005) with Dunn's multiple comparison tests **c**. $CD4^+$ cells, AUC values were calculated for each mouse and compared between groups using the Kruskal–Wallis test (p = 0.004), with Dunn's multiple comparisons test. Throughout, n indicates the number of individual mice, each an independent biological replicate.

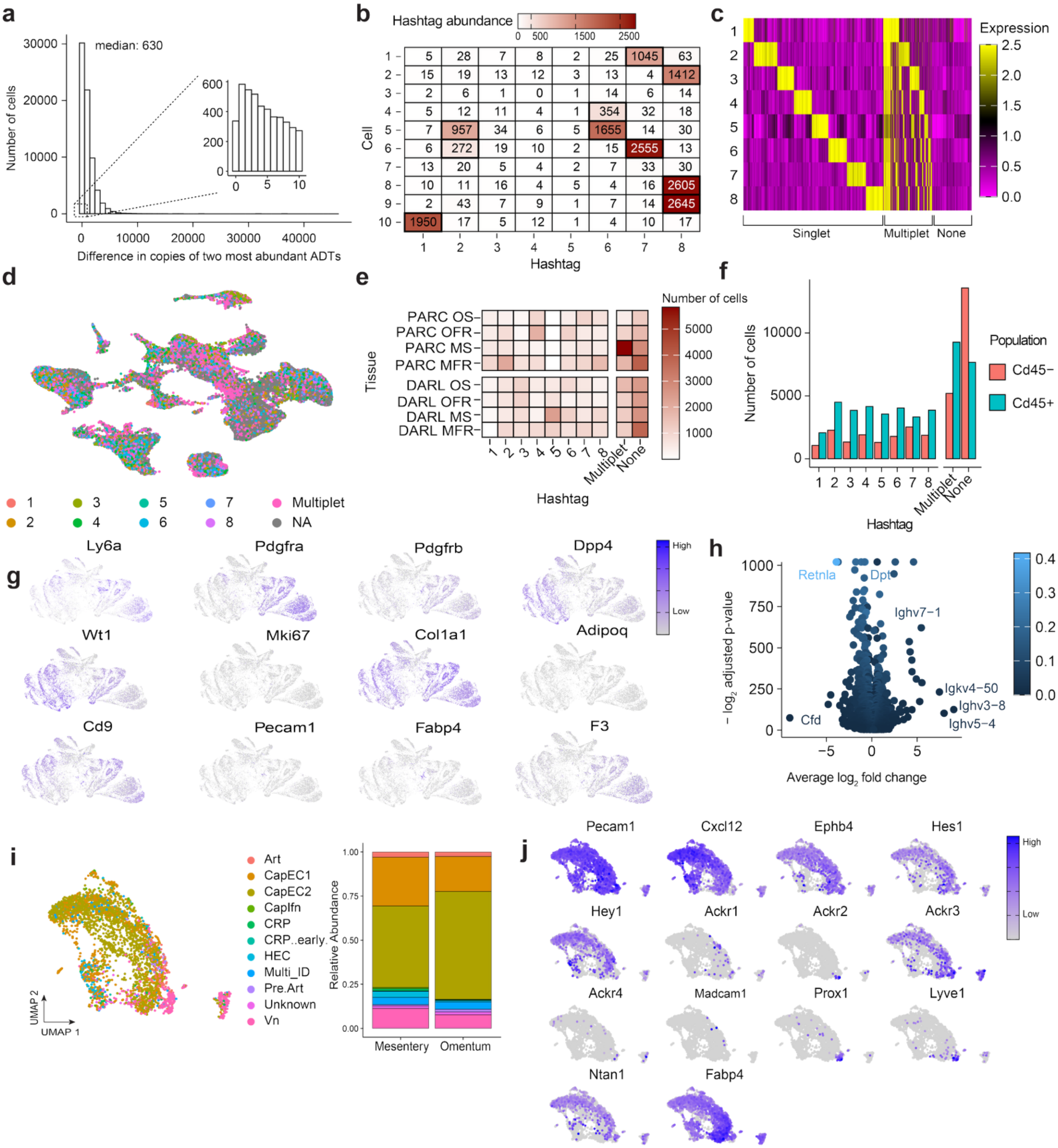
a
median: 630
Number of cells
Difference in copies of two most abundant ADTs
b
Hashtag abundance
0 500 1500 2500
Cell
Hashtag
1 – 5 28 7 8 2 25 1045 63
2 – 15 19 13 12 3 13 4 1412
3 – 2 6 1 0 1 14 6 14
4 – 5 12 11 4 1 354 32 18
5 – 7 957 34 6 5 1655 14 30
6 – 6 272 19 10 2 15 2555 13
7 – 13 20 5 4 2 7 33 30
8 – 10 11 16 4 5 4 16 2605
9 – 2 43 7 9 1 7 14 2645
10 – 1950 17 5 12 1 4 10 17
c
Expression
Singlet
Multiplet
None
d
Multiplet
NA
e
Number of cells
Tissue
PARC OS
PARC OFR
PARC MS
PARC MFR
DARL OS
DARL OFR
DARL MS
DARL MFR
Multiplet
None
Hashtag
f
Number of cells
Population
Cd45−
Cd45+
Multiplet
None
Hashtag
g
Ly6a
Pdgfra
Pdgfrb
Dpp4
Wt1
Mki67
Col1a1
Adipoq
Cd9
Pecam1
Fabp4
F3
High
Low
h
Retnla
Dpt
Ighv7−1
Igkv4−50
Ighv3−8
Ighv5−4
Cfd
− log2 adjusted p-value
Average log2 fold change
i
UMAP 2
UMAP 1
Art
CapEC1
CapEC2
CapIfn
CRP
CRP..early.
HEC
Multi_ID
Pre.Art
Unknown
Vn
Relative Abundance
Mesentery
Omentum
j
Pecam1
Cxcl12
Ephb4
Hes1
Hey1
Ackr1
Ackr2
Ackr3
Ackr4
Madcam1
Prox1
Lyve1
Ntan1
Fabp4
High
Low

Supplementary Figure 6. Hashtag-based sample assignment and endothelial characterization of PARC and DARL single-cell RNA-sequencing data.

a. Histogram showing the difference in copies between the two most abundant antibody-derived tags (ADTs) detected in each cell across all PARC (n=8) and DARL (n=8) tissue compartments, including the omentum adipose region (OAR), mesentery adipose region (MAR), omentum peritoneal membrane (OPM), and mesenteric peritoneal membrane (MPM). **b**. Representative table of the hashtag assignment for 10 randomized cells based on the number of oligo copies detected per cell. Bolded tiles indicate the hashtag assigned to each cell. Cells 3 and 7 were not assigned to any hashtag; cells 5 and 6 were assigned as multiplets of hashtags 2/6 and 2/7, respectively. **c**. Heat map of scaled (z-score) normalized antibody counts sorted by classification for a random sample of 5,000 cells. **d**. UMAP visualization of the hashtag distribution across all cells. **e**. Quantification of the cell numbers across the eight hashtag assignments across all tissues. **f**. Quantification of $CD45^+$ and $CD45^-$ cells across all PARC and DARL tissues (OAR, OPM, MAR, MPM). **g.** Validation of Capybara stromal sub-classifications of adipocyte lineage[57] and mesothelial cells[35] shown in Figure 5d based on the expression of canonical marker genes **h**. Differential gene expression between $CD45^-$ DARL and $CD45^-$ PARC cells after Bonferroni correction of p-values. Features with positive fold change are upregulated among DARL cells; features with negative fold change are upregulated among PARC cells. Features are colored by the absolute difference in the fraction of cells expressing each feature across the two groups. **i**. UMAP visualization of the Capybara comparison to cell identities classified in a murine lymph node blood vascular endothelium dataset[62] (left). Art, arterial endothelial cells; CapEC1, capillary endothelial cell subtype 1; CapEC2, capillary endothelial cell subtype 2; CapIfn, interferon-responsive capillary endothelial cells; CRP, cycling (proliferative) endothelial cells; CRP_early, early cycling endothelial cells; HEC, high endothelial cells; Pre.Art, pre-arterial endothelial cells; Vn, venous endothelial cells. Comparison of the endothelial cell types identified by Capybara between the omentum and mesentery endothelium (right). **j**. Validation of Capybara endothelial subclassification based on the expression of canonical marker genes and co-localization of *Fabp4* expression. Throughout, n indicates the number of individual mice (n = 8 per genotype), each an independent biological replicate.

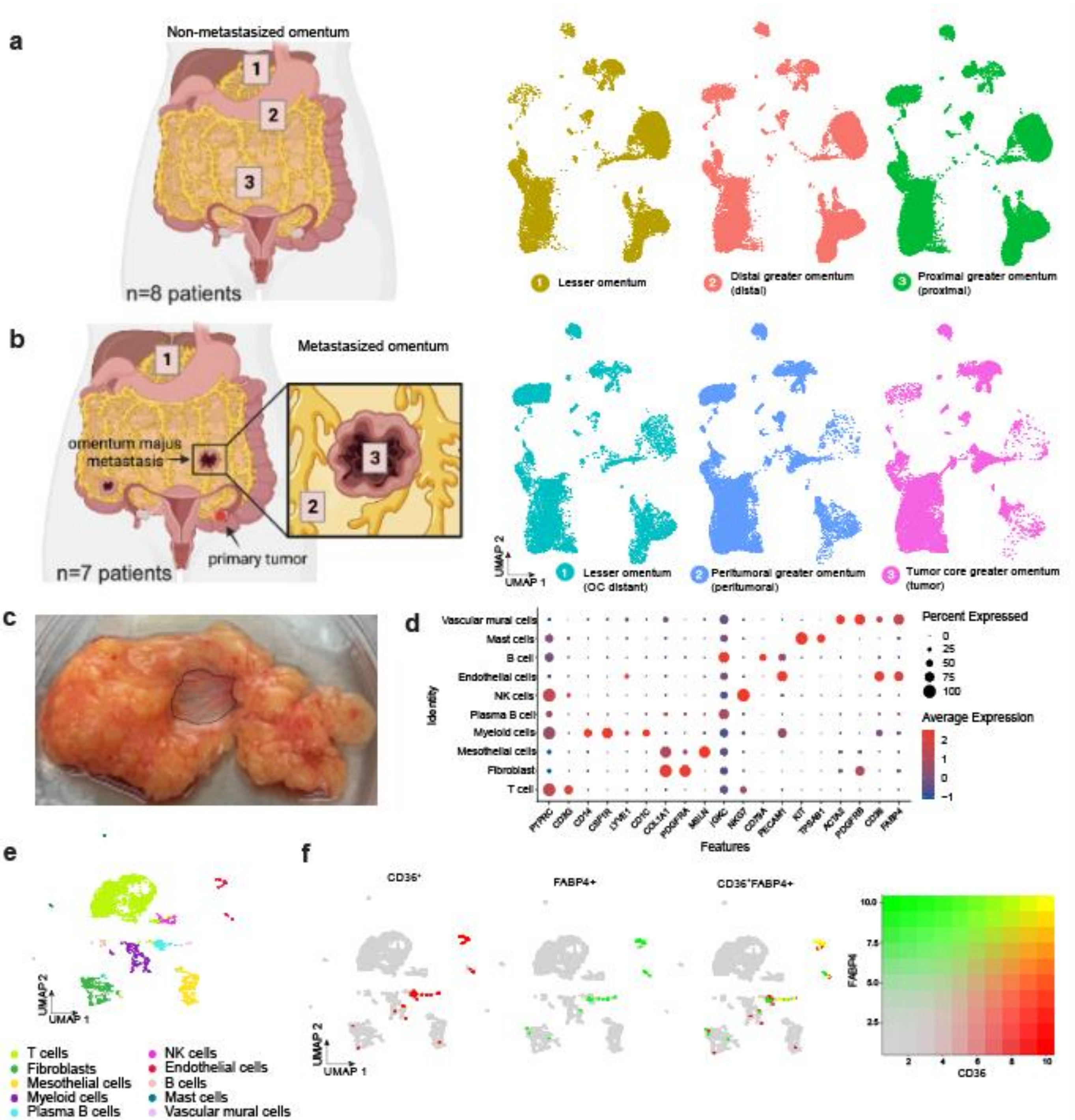

Supplementary Figure 7. Human omental single-cell RNA-seq and comparison with published datasets support FABP4⁺ endothelial populations.

a. Schematic of the sampling strategy for non-metastasized omentum and UMAP visualization of single-cell RNA-sequencing data from a previously published human dataset (GSE286057) of human omental tissue[6], stratified by sampling location as defined in Fig. 6m. For benign cases (P1–P8), samples include proximal and distal regions relative to benign or non-malignant nodules, as well as normal lesser omentum. Cells are shown separately for each location in non-metastasized (lesser omentum, distal greater omentum, proximal greater omentum). **b.** Schematic of the sampling strategy for metastasized omentum and UMAP visualization of single-cell RNA-sequencing data from the same previously published human dataset (GSE286057)[6] of human omental tissue, stratified by sampling location as defined in Fig. 6m. For OC cases (P9–P15), samples include metastatic tumor nodules and proximal and distal omental regions relative to the tumor. Cells are shown separately for each location in the metastasized omentum (lesser omentum, peritumoral greater omentum, and tumor core greater omentum). **c**. Benign human omentum tissue obtained for single-cell RNA sequencing.

The translucent peritoneal membrane (outlined in black) and adipocyte-rich regions were dissected and sequenced separately to capture distinct omental compartments. **d**. Dot plot of specific genes expressed in clustered cell types. *FABP4 is* highly expressed in endothelial cells and vascular mural cells. **e**. UMAP projection of all cells from human omental single-cell RNA-seq, showing 11 transcriptionally defined clusters. **f**. Feature plots showing the expression of CD36 and FABP4, and their co–expression across the human omental dataset. FABP4 and CD36 are co-expressed in a subset of endothelial cells, suggesting a conserved lipid-handling vascular phenotype. Supplementary Figures 7a and 7b are reused from Ref. 6 (GSE286057), released under a Creative Commons Attribution-NonCommercial-NoDerivs 4.0 International license (https://creativecommons.org/licenses/by-nc-nd/4.0/deed.en). Throughout, n indicates the number of individual patients.

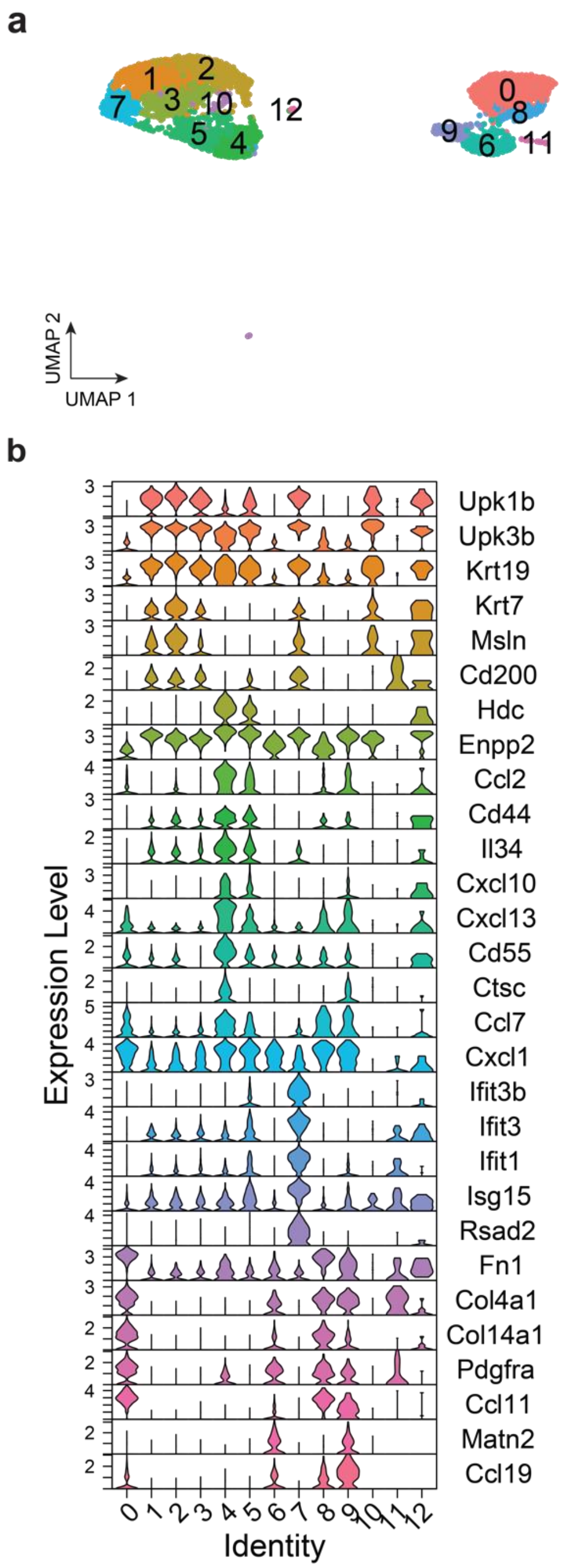

Supplementary Figure 8. Cell type classification of stromal cells in the mouse omentum

a. UMAP embedding of non-endothelial stromal cells from the omentum colored by Louvain clusters. Raw data from Jackson-Jones *et al*. were obtained from GEO and re-processed (GSM4053741)[35]. 3,817 cells were used to construct the reference after removing cells with fewer than 1,000 genes per cell (*nFeature_RNA*). Louvain clustering was performed using the default parameters of the Seurat function *FindClusters()* (Seurat package version 5.1.0). **b**. Violin plots showing expression of marker genes used to identify cell types. Louvain clusters 1, 2, 3, 4, 5, 7, 10, and 12 were identified as mesothelial, based on positive expression of epithelial and mesothelial markers *Upk1b*, *Upk3b*, *Krt19*, *Krt7*, *Msln*, and *Cd200*. A subset of these, clusters 4 and 5, were identified as Cxcl13+ mesothelium by positive expression of *Hdc*, *Enpp2*, *Ccl2*, *Cd44*, *Il34*, *Cxcl10*, *Cxcl13*, *Cd55*, *Ctsc*, *Ccl7*, and *Cxcl1*. Another subset of these, cluster 7, was identified as Ifit+ mesothelium by positive expression of *Ifit3b*, *Ifit3*, *Ifit1*, *Isg15*, and *Rsad2*. Louvain clusters 0, 8, and 9 were identified as Ccl11+/Pdgfra+ fibroblasts by positive expression of *Fn1*, *Col4a1*, *Col14a1*, and *Pdgfra*. Louvain cluster 6 was identified as Matn2+/Pdgfra+ fibroblasts by positive expression of *Matn2* and *Ccl19*. Finally, Louvain cluster 11 was not assigned by the authors.
