# Supplementary material for: Peritoneal ovarian cancer growth in the omentum proceeds independently of mature adipocytes
Source: Nat Commun. 2026 Aug 12;17:9673. doi: 10.1038/s41467-026-76471-x (PMC13558732; doi:10.1038/s41467-026-76471-x)
Supplement: Supplementary file 2 [file 41467_2026_76471_MOESM2_ESM.pdf]

Description of Additional Supplementary Files

Supplementary Data 1 (See Supplemental Table 1, xls file). Differential gene expression between PARC and DARL peritoneal cells. Pooled differential gene expression across all captured single cells from the omentum and mesentery of PARC (n = 8 mice) and DARL (n = 8 mice), rather than organ- or cell-type-specific comparisons. Differential expression was performed in Seurat (v5.1.0) using the FindMarkers function with a two-sided Wilcoxon rank-sum test; p-values were adjusted for multiple comparisons by Bonferroni correction across all genes tested, and the table lists the 3,498 genes reaching significance (adjusted $p < 0.05$). Columns: gene, gene symbol; p_val, unadjusted Wilcoxon rank-sum p-value; avg_log2FC, average log2 fold-change between genotypes (positive values, higher in DARL; negative values, higher in PARC); fraction of DARL cells and fraction of PARC cells, proportion of cells of each genotype expressing the gene (Seurat pct.1 and pct.2); p_val_adj, Bonferroni-adjusted p-value; pct_diff, difference in the fraction of expressing cells between genotypes; FC_rank, pct_diff_rank and rank_sum, gene rankings by fold-change, by difference in percent expression, and by the sum of those two ranks, respectively. Established adipokines (Cfd, Retnla) are among the genes most strongly enriched in PARC relative to DARL cells, serving as a positive control confirming the loss of mature adipocytes in DARL mice, whereas immunoglobulin variable-region transcripts are among those enriched in DARL cells.
