# Supplementary material for: Peritoneal ovarian cancer growth in the omentum proceeds independently of mature adipocytes
Source: Nat Commun. 2026 Aug 12;17:9673. doi: 10.1038/s41467-026-76471-x (PMC13558732; doi:10.1038/s41467-026-76471-x)
Supplement: Supplementary file 3 [file 41467_2026_76471_MOESM4_ESM.pdf]

nature portfolio

Corresponding author(s): Gwendalyn J. Randolph

Last updated by author(s): Jun 13, 2026

Reporting Summary

Nature Portfolio wishes to improve the reproducibility of the work that we publish. This form provides structure for consistency and transparency in reporting. For further information on Nature Portfolio policies, see our Editorial Policies and the Editorial Policy Checklist.

Statistics

For all statistical analyses, confirm that the following items are present in the figure legend, table legend, main text, or Methods section.

n/a	Confirmed	
☐	☒	The exact sample size (*n*) for each experimental group/condition, given as a discrete number and unit of measurement
☐	☒	A statement on whether measurements were taken from distinct samples or whether the same sample was measured repeatedly
☐	☒	The statistical test(s) used AND whether they are one- or two-sided<br>*Only common tests should be described solely by name; describe more complex techniques in the Methods section.*
☒	☐	A description of all covariates tested
☐	☒	A description of any assumptions or corrections, such as tests of normality and adjustment for multiple comparisons
☐	☒	A full description of the statistical parameters including central tendency (e.g. means) or other basic estimates (e.g. regression coefficient) AND variation (e.g. standard deviation) or associated estimates of uncertainty (e.g. confidence intervals)
☐	☒	For null hypothesis testing, the test statistic (e.g. F, t, r) with confidence intervals, effect sizes, degrees of freedom and P value noted<br>*Give P values as exact values whenever suitable.*
☒	☐	For Bayesian analysis, information on the choice of priors and Markov chain Monte Carlo settings
☐	☒	For hierarchical and complex designs, identification of the appropriate level for tests and full reporting of outcomes
☒	☐	Estimates of effect sizes (e.g. Cohen's *d*, Pearson's *r*), indicating how they were calculated

Our web collection on statistics for biologists contains articles on many of the points above.

Software and code

Policy information about availability of computer code

Data collection	Bioluminescence images were acquired using Living Image software (PerkinElmer; v4.3.1 and v2.6; RRID: SCR_014247) on the IVIS50 system, and ex vivo bioluminescence/colorimetric images using Image Lab on a ChemiDoc MP Imaging System (Bio-Rad). Spectral flow cytometry data were acquired with SpectroFlo (Cytek) on a Cytek Aurora (5-laser) spectral cytometer. Confocal z-stacks were acquired with Leica LAS X on an inverted Leica SP8 microscope. Leukocyte counts were obtained on a Nexcelom Cellometer Auto X4.
Data analysis	GraphPad Prism 10 (RRID: SCR_002798); FIJI/ImageJ v2.14.0/1.54p (RRID: SCR_003070); Imaris v10.1.1 and v9.x (Bitplane/Oxford Instruments); Python 3.12 (NumPy, Pillow); FlowJo v10.10 (RRID: SCR_008520); Cell Ranger v3.1.0, 6.1.1 and 7.0.1; R with Seurat v5.1.0 and Harmony integration; Capybara v0.0.0.9. The custom Python script used to quantify vascular lacunarity is available at Zenodo (https://doi.org/10.5281/zenodo.20683730). No other custom code central to the conclusions was generated.

For manuscripts utilizing custom algorithms or software that are central to the research but not yet described in published literature, software must be made available to editors and reviewers. We strongly encourage code deposition in a community repository (e.g. GitHub). See the Nature Portfolio guidelines for submitting code & software for further information.

Data

Policy information about availability of data

All manuscripts must include a data availability statement. This statement should provide the following information, where applicable:

- Accession codes, unique identifiers, or web links for publicly available datasets
- A description of any restrictions on data availability
- For clinical datasets or third party data, please ensure that the statement adheres to our policy

Murine single-cell sequencing data generated in this study have been deposited in the Gene Expression Omnibus (GEO) (RRID: SCR_005012) under accession code GSE281706. Human single-cell sequencing data generated in this study have been deposited in the GEO under accession code GSE300038. Publicly available single-cell RNA-sequencing datasets analysed in this study are available under GEO accession numbers GSM4053741, GSE140348, GSE241627, and GSE286057. Source Data are provided with this paper. All other data supporting the findings of this study are provided in the Source Data file or within the Article and its Supplementary Information.

Research involving human participants, their data, or biological material

Policy information about studies with human participants or human data. See also policy information about sex, gender (identity/presentation), and sexual orientation and race, ethnicity and racism.

Reporting on sex and gender	This study utilized female mice exclusively because ovarian cancer is a malignancy of the female reproductive system and peritoneal metastasis to the omentum occurs in the context of female reproductive physiology. The experimental model is designed to recapitulate ovarian cancer metastasis, for which female animals are the biologically necessary subject. Inclusion of male animals would be scientifically inappropriate and without biological justification. Sex-disaggregated analysis is not applicable. All animals used in this study were female, and genotype cohorts were matched by sex throughout all experiments. The human omentum donor was female; sex was self-reported. Only female tissue was studied, consistent with the sex-specific nature of ovarian cancer.
Reporting on race, ethnicity, or other socially relevant groupings	Race, ethnicity, and other socially relevant groupings were not reported, as samples were de-identified prior to research use.
Population characteristics	De-identified human omentum was obtained from a 43 year old female donor undergoing surgical staging for a granulosa cell tumor of the ovary, via the Washington University Ovarian Cancer Tissue Bank under IRB approval number 201903051. The omentum was grossly unremarkable, with no macroscopic or microscopic tumor involvement on final pathology. Sex was self-reported. No other demographic or clinical characteristics were collected or analyzed. All primary in vivo experiments were conducted in murine models.
Recruitment	Human omentum tissue was obtained through the Washington University Ovarian Cancer Tissue Bank under IRB approval number 201903051. Written informed consent was obtained and the donor received no compensation. Tissue was de-identified prior to research use; no prospective recruitment of human participants was performed, as the specimen was obtained during standard surgical staging.
Ethics oversight	Human omentum tissue used in this study was obtained from de-identified donors via the Washington University Ovarian Cancer Tissue Bank under IRB approval number 201903051.

Note that full information on the approval of the study protocol must also be provided in the manuscript.

Field-specific reporting

Please select the one below that is the best fit for your research. If you are not sure, read the appropriate sections before making your selection.

☒ Life sciences ☐ Behavioural & social sciences ☐ Ecological, evolutionary & environmental sciences

For a reference copy of the document with all sections, see nature.com/documents/nr-reporting-summary-flat.pdf

Life sciences study design

All studies must disclose on these points even when the disclosure is negative.

Sample size	Sample sizes were not predetermined using a formal power calculation. They were based on pilot experiments and prior studies using these models and are consistent with sample sizes commonly used in the field. The number of mice used in each experiment is indicated in the corresponding figure legend.
Data exclusions	Animals that died prior to the experimental endpoint were included in all analyses up to the time of death, with subsequent timepoints treated as missing. Bioluminescence imaging timepoints were excluded and repeated if possible when failed intraperitoneal luciferin injection precluded reliable signal quantification; all remaining timepoints for that animal were retained and included in the analysis. No animals were excluded from any in vivo experiment. Exclusion criteria were not pre-established prior to the study but were applied on the basis of technical validity independent of experimental outcome.

Replication	Key findings were reproduced across multiple independent experiments and across multiple ovarian cancer models, as indicated in the figure legends. All replication attempts were successful.
Randomization	Formal randomization was not applicable to genotype comparisons, as group allocation was determined by genotype. Within each genotype, mice were co-housed with littermate controls and treated simultaneously under identical experimental conditions. For tumor injection experiments, mice were injected in random order without regard to genotype, and cages contained mixed genotypes throughout the experimental period. For experiments requiring assignment to treatment or control conditions, mice were randomly allocated as described in the corresponding figure legends.
Blinding	Investigators were not formally blinded to genotype during in vivo data collection, because genotype-associated tissue differences (e.g., the presence or absence of mature adipocytes) are macroscopically apparent and were required to direct experimental procedures. Observer bias was minimized by quantifying tumor nodules with standardized automated thresholding and particle analysis and by using fixed regions of interest for bioluminescence quantification; animals were tracked by number and genotype identity was revealed only at the analysis and plotting stage.

Behavioural & social sciences study design

All studies must disclose on these points even when the disclosure is negative.

Study description	*Briefly describe the study type including whether data are quantitative, qualitative, or mixed-methods (e.g. qualitative cross-sectional, quantitative experimental, mixed-methods case study).*
Research sample	*State the research sample (e.g. Harvard university undergraduates, villagers in rural India) and provide relevant demographic information (e.g. age, sex) and indicate whether the sample is representative. Provide a rationale for the study sample chosen. For studies involving existing datasets, please describe the dataset and source.*
Sampling strategy	*Describe the sampling procedure (e.g. random, snowball, stratified, convenience). Describe the statistical methods that were used to predetermine sample size OR if no sample-size calculation was performed, describe how sample sizes were chosen and provide a rationale for why these sample sizes are sufficient. For qualitative data, please indicate whether data saturation was considered, and what criteria were used to decide that no further sampling was needed.*
Data collection	*Provide details about the data collection procedure, including the instruments or devices used to record the data (e.g. pen and paper, computer, eye tracker, video or audio equipment) whether anyone was present besides the participant(s) and the researcher, and whether the researcher was blind to experimental condition and/or the study hypothesis during data collection.*
Timing	*Indicate the start and stop dates of data collection. If there is a gap between collection periods, state the dates for each sample cohort.*
Data exclusions	*If no data were excluded from the analyses, state so OR if data were excluded, provide the exact number of exclusions and the rationale behind them, indicating whether exclusion criteria were pre-established.*
Non-participation	*State how many participants dropped out/declined participation and the reason(s) given OR provide response rate OR state that no participants dropped out/declined participation.*
Randomization	*If participants were not allocated into experimental groups, state so OR describe how participants were allocated to groups, and if allocation was not random, describe how covariates were controlled.*

Ecological, evolutionary & environmental sciences study design

All studies must disclose on these points even when the disclosure is negative.

Study description	*Briefly describe the study. For quantitative data include treatment factors and interactions, design structure (e.g. factorial, nested, hierarchical), nature and number of experimental units and replicates.*
Research sample	*Describe the research sample (e.g. a group of tagged Passer domesticus, all Stenocereus thurberi within Organ Pipe Cactus National Monument), and provide a rationale for the sample choice. When relevant, describe the organism taxa, source, sex, age range and any manipulations. State what population the sample is meant to represent when applicable. For studies involving existing datasets, describe the data and its source.*
Sampling strategy	*Note the sampling procedure. Describe the statistical methods that were used to predetermine sample size OR if no sample-size calculation was performed, describe how sample sizes were chosen and provide a rationale for why these sample sizes are sufficient.*
Data collection	*Describe the data collection procedure, including who recorded the data and how.*
Timing and spatial scale	*Indicate the start and stop dates of data collection, noting the frequency and periodicity of sampling and providing a rationale for these choices. If there is a gap between collection periods, state the dates for each sample cohort. Specify the spatial scale from which the data are taken*
Data exclusions	*If no data were excluded from the analyses, state so OR if data were excluded, describe the exclusions and the rationale behind them,*

Data exclusions	*indicating whether exclusion criteria were pre-established.*
Reproducibility	*Describe the measures taken to verify the reproducibility of experimental findings. For each experiment, note whether any attempts to repeat the experiment failed OR state that all attempts to repeat the experiment were successful.*
Randomization	*Describe how samples/organisms/participants were allocated into groups. If allocation was not random, describe how covariates were controlled. If this is not relevant to your study, explain why.*
Blinding	*Describe the extent of blinding used during data acquisition and analysis. If blinding was not possible, describe why OR explain why blinding was not relevant to your study.*

Did the study involve field work? ☐ Yes ☐ No

Field work, collection and transport

Field conditions	*Describe the study conditions for field work, providing relevant parameters (e.g. temperature, rainfall).*
Location	*State the location of the sampling or experiment, providing relevant parameters (e.g. latitude and longitude, elevation, water depth).*
Access & import/export	*Describe the efforts you have made to access habitats and to collect and import/export your samples in a responsible manner and in compliance with local, national and international laws, noting any permits that were obtained (give the name of the issuing authority, the date of issue, and any identifying information).*
Disturbance	*Describe any disturbance caused by the study and how it was minimized.*

Reporting for specific materials, systems and methods

We require information from authors about some types of materials, experimental systems and methods used in many studies. Here, indicate whether each material, system or method listed is relevant to your study. If you are not sure if a list item applies to your research, read the appropriate section before selecting a response.

Materials & experimental systems

n/a	Involved in the study	
☐	☒	Antibodies
☐	☒	Eukaryotic cell lines
☒	☐	Palaeontology and archaeology
☐	☒	Animals and other organisms
☒	☐	Clinical data
☒	☐	Dual use research of concern
☒	☐	Plants

Methods

n/a	Involved in the study	
☒	☐	ChIP-seq
☐	☒	Flow cytometry
☒	☐	MRI-based neuroimaging

Antibodies

Antibodies used	In vivo depletion (Leinco Technologies, functional-grade): anti-mouse CD4 (clone GK1.5; Cat# C1333, RRID: AB_2737452), anti-mouse CD8b.2 (clone 53-5.8; Cat# C2832, RRID: AB_2737471), and anti-mouse NK1.1 (clone PK136; Cat# N123, RRID: AB_2737553); mice received 250 μg/week (CD4, CD8) and 100 μg/week (NK1.1).<br>Spectral flow cytometry (1:300): CD45 (clone 30-F11; BD Biosciences Cat# 748370, RRID: AB_2872789), F4/80 (clone BM8; BioLegend Cat# 123168, RRID: AB_2924461), CD64 (clone X54-5/7.1; BioLegend Cat# 139313, RRID: AB_2563903), ICAM-2/CD102 (clone 3C4 [mIC2/4]; BD Biosciences Cat# 740864, RRID: AB_2740516), CD169 (clone 3D6.112; BioLegend Cat# 142416, RRID: AB_2565620), LYVE-1 (clone ALY7; Thermo Fisher/eBioscience Cat# 53-0443-82, RRID: AB_1633415), MHC II/I-A-I-E (clone M5/114.15.2; BD Biosciences Cat# 750281, RRID: AB_2874472), CD31 (clone MEC13.3; BD Biosciences Cat# 741262, RRID: AB_2870809), CD11b (clone M1/70; BD Biosciences Cat# 564443, RRID: AB_2738811), Ly-6G (clone 1A8; BioLegend Cat# 127641, RRID: AB_2565881), Ly-6C (clone HK1.4; BioLegend Cat# 128029, RRID: AB_10896061), CD11c (clone N418; Thermo Fisher/eBioscience Cat# 363-0114-82, RRID: AB_2925254), NK-1.1 (clone S17016D; BioLegend Cat# 156519, RRID: AB_2894654), CD4 (clone GK1.5; BioLegend Cat# 100467, RRID: AB_2734150), CD8a (clone 53-6.7; BioLegend Cat# 100791, RRID: AB_2876397), CD19 (clone eBio1D3 [1D3]; Thermo Fisher/eBioscience Cat# 47-0193-82, RRID: AB_10853189), podoplanin (clone 8.1.1; BioLegend Cat# 127411, RRID: AB_10613294), PDGFR-α/CD140a (clone APA5; BioLegend Cat# 135913, RRID: AB_2715986), and CD9 (clone KMC8; BD Biosciences Cat# 751253, RRID: AB_2875270).<br>scRNA-seq sorting/hashing: CD45 (clone 30-F11; BioLegend Cat# 103115, RRID: AB_312980), TER-119 (clone TER-119; BioLegend Cat# 116205, RRID: AB_313706), and TotalSeq cell-hashing antibodies (BioLegend Cat# 155861–155875; RRID: AB_2800693–AB_2819912).<br>3D confocal imaging: anti-CD31 (goat polyclonal; R&D Systems Cat# AF3628, RRID: AB_2161028; 1:200), anti-α-smooth muscle actin–Alexa Fluor 488 (clone 1A4; Thermo Fisher/eBioscience Cat# 53-9760-82, RRID: AB_2574461; 1:200), donkey anti-goat IgG–Alexa Fluor 568 secondary (polyclonal; Invitrogen Cat# A-11057, RRID: AB_2534104; 1:500), and DAPI (Sigma-Aldrich D5942; 1:500). Lot

	numbers were not recorded; each antibody is uniquely identified by its RRID.
Validation	All antibodies are commercially available and were validated by the manufacturers for the stated species (mouse) and applications (flow cytometry, immunofluorescence, or in vivo depletion); validation details are accessible via each RRID in the Antibody Registry (antibodyregistry.org). Flow cytometry specificity was confirmed by the expected cell populations in control samples and fluorescence-minus-one controls. The depleting anti-CD4, anti-CD8b.2, and anti-NK1.1 antibodies were validated in vivo by flow-cytometric confirmation of target-cell depletion in peripheral blood throughout the experiments (Supplementary Fig. 5). Imaging antibodies were confirmed by the expected anatomical distribution of each marker and by secondary-antibody-only controls.

Eukaryotic cell lines

Policy information about cell lines and Sex and Gender in Research

Cell line source(s)	ID8 cells were obtained from Katherine Roby, who created this cell line. ID8 p53–/– Brca2–/– cell lines were developed by the McNeish lab and provided by Dr. Khabele. BPPNM and KPCA cell lines were developed by the Weinberg lab. All cell lines were labeled with luciferase by Nan Zhang, who sorted them to ensure homogeneous luciferase expression.
Authentication	All cell lines were authenticated by confirmation of GFP and luciferase expression prior to use in experiments. Cell lines were obtained directly from the originating laboratories; independent STR profiling was not performed, as no standard STR profile exists for these murine lines.
Mycoplasma contamination	All cell lines tested negative for mycoplasma contamination. Cells were tested prior to experimental use and retested every six months throughout the study period.
Commonly misidentified lines (See ICLAC register)	No commonly misidentified cell lines were used.

Palaeontology and Archaeology

Specimen provenance	*Provide provenance information for specimens and describe permits that were obtained for the work (including the name of the issuing authority, the date of issue, and any identifying information). Permits should encompass collection and, where applicable, export.*
Specimen deposition	*Indicate where the specimens have been deposited to permit free access by other researchers.*
Dating methods	*If new dates are provided, describe how they were obtained (e.g. collection, storage, sample pretreatment and measurement), where they were obtained (i.e. lab name), the calibration program and the protocol for quality assurance OR state that no new dates are provided.*

☐ Tick this box to confirm that the raw and calibrated dates are available in the paper or in Supplementary Information.

Ethics oversight	*Identify the organization(s) that approved or provided guidance on the study protocol, OR state that no ethical approval or guidance was required and explain why not.*

Note that full information on the approval of the study protocol must also be provided in the manuscript.

Animals and other research organisms

Policy information about studies involving animals; ARRIVE guidelines recommended for reporting animal research, and Sex and Gender in Research

Laboratory animals	All in vivo experiments used female mice on the C57BL/6J background: wild-type C57BL/6J; AdiponectinCre/+ Rosa26-DTA fl/+ (lipoatrophic) mice with Adiponectin+/+ Rosa26-DTA fl/+ littermate controls; CD19Cre/+ Rosa-TdTomato (CD19Cre/+TD) reporter mice; B6.129S Rag2/Cd47/Il2rg triple-knockout mice with age-matched C57BL/6J controls; and Tie2-Cre+ Fabp4 fl/fl and Lyz2-Cre+ Fabp4 fl/fl mice with Fabp4 fl/fl littermate controls (provided through MTA with Gökhan S. Hotamişligil). Fat-transplant donor mice were ~14–18 weeks old; recipient lipoatrophic mice were 7–10 weeks old at surgery. Mice were housed at 20–22 °C, 30–70% humidity, on a 12-h light/dark cycle; lipoatrophic mice and their controls were maintained at thermoneutrality (30 °C).
Wild animals	No wild animals were collected.
Reporting on sex	All animals used in this study were female. See the sex and gender reporting section above for full justification.
Field-collected samples	No field-collected samples were used.
Ethics oversight	All animal procedures were conducted in accordance with protocols approved by the Washington University in St. Louis Institutional Animal Care and Use Committee (protocol number 25-0333).

Note that full information on the approval of the study protocol must also be provided in the manuscript.

Clinical data

Policy information about clinical studies

All manuscripts should comply with the ICMJE guidelines for publication of clinical research and a completed CONSORT checklist must be included with all submissions.

Clinical trial registration	*Provide the trial registration number from ClinicalTrials.gov or an equivalent agency.*
Study protocol	*Note where the full trial protocol can be accessed OR if not available, explain why.*
Data collection	*Describe the settings and locales of data collection, noting the time periods of recruitment and data collection.*
Outcomes	*Describe how you pre-defined primary and secondary outcome measures and how you assessed these measures.*

Dual use research of concern

Policy information about dual use research of concern

Hazards

Could the accidental, deliberate or reckless misuse of agents or technologies generated in the work, or the application of information presented in the manuscript, pose a threat to:

No	Yes	
☐	☐	Public health
☐	☐	National security
☐	☐	Crops and/or livestock
☐	☐	Ecosystems
☐	☐	Any other significant area

Experiments of concern

Does the work involve any of these experiments of concern:

No	Yes	
☐	☐	Demonstrate how to render a vaccine ineffective
☐	☐	Confer resistance to therapeutically useful antibiotics or antiviral agents
☐	☐	Enhance the virulence of a pathogen or render a nonpathogen virulent
☐	☐	Increase transmissibility of a pathogen
☐	☐	Alter the host range of a pathogen
☐	☐	Enable evasion of diagnostic/detection modalities
☐	☐	Enable the weaponization of a biological agent or toxin
☐	☐	Any other potentially harmful combination of experiments and agents

Plants

Seed stocks	*Report on the source of all seed stocks or other plant material used. If applicable, state the seed stock centre and catalogue number. If plant specimens were collected from the field, describe the collection location, date and sampling procedures.*
Novel plant genotypes	*Describe the methods by which all novel plant genotypes were produced. This includes those generated by transgenic approaches, gene editing, chemical/radiation-based mutagenesis and hybridization. For transgenic lines, describe the transformation method, the number of independent lines analyzed and the generation upon which experiments were performed. For gene-edited lines, describe the editor used, the endogenous sequence targeted for editing, the targeting guide RNA sequence (if applicable) and how the editor was applied.*
Authentication	*Describe any authentication procedures for each seed stock used or novel genotype generated. Describe any experiments used to assess the effect of a mutation and, where applicable, how potential secondary effects (e.g. second site T-DNA insertions, mosiacism, off-target gene editing) were examined.*

ChIP-seq

Data deposition

☐ Confirm that both raw and final processed data have been deposited in a public database such as GEO.

☐ Confirm that you have deposited or provided access to graph files (e.g. BED files) for the called peaks.

Data access links<br>*May remain private before publication.*	*For "Initial submission" or "Revised version" documents, provide reviewer access links. For your "Final submission" document, provide a link to the deposited data.*
Files in database submission	*Provide a list of all files available in the database submission.*
Genome browser session<br>(e.g. UCSC)	*Provide a link to an anonymized genome browser session for "Initial submission" and "Revised version" documents only, to enable peer review. Write "no longer applicable" for "Final submission" documents.*

Methodology

Replicates	*Describe the experimental replicates, specifying number, type and replicate agreement.*
Sequencing depth	*Describe the sequencing depth for each experiment, providing the total number of reads, uniquely mapped reads, length of reads and whether they were paired- or single-end.*
Antibodies	*Describe the antibodies used for the ChIP-seq experiments; as applicable, provide supplier name, catalog number, clone name, and lot number.*
Peak calling parameters	*Specify the command line program and parameters used for read mapping and peak calling, including the ChIP, control and index files used.*
Data quality	*Describe the methods used to ensure data quality in full detail, including how many peaks are at FDR 5% and above 5-fold enrichment.*
Software	*Describe the software used to collect and analyze the ChIP-seq data. For custom code that has been deposited into a community repository, provide accession details.*

Flow Cytometry

Plots

Confirm that:

☒ The axis labels state the marker and fluorochrome used (e.g. CD4-FITC).

☒ The axis scales are clearly visible. Include numbers along axes only for bottom left plot of group (a 'group' is an analysis of identical markers).

☒ All plots are contour plots with outliers or pseudocolor plots.

☒ A numerical value for number of cells or percentage (with statistics) is provided.

Methodology

Sample preparation	Single-cell suspensions were prepared from murine omentum and mesentery. Tissues were minced with scissors and digested using 1 mg/mL collagenase IV, 0.1 mg/mL DNase, and 0.5% BSA in RPMI 1640 at 37°C. Digestion was stopped with chilled FACS buffer, and suspensions were passed through a 70 µm filter before centrifugation at 500 g for 5 minutes at 4°C. For peripheral blood, red blood cells were lysed using BD PharmLyse lysis buffer. Cell pellets were resuspended in flow cytometry buffer (HBSS containing 2% FBS and 5 mM EDTA) with 20% BD Horizon Brilliant Stain Buffer and stained with the antibody panel for 30 minutes at 4°C. Cells were washed twice before acquisition.
Instrument	Flow cytometry data were acquired on a Cytek Aurora spectral cytometer with a 5-laser configuration equipped with full-spectral hybrid detectors.
Software	Spectral unmixing and flow cytometry data analysis, including unsupervised analysis, were conducted using FlowJo software (v10.10, RRID: SCR_008520). No custom code was deposited.
Cell population abundance	Manual flow cytometry gating was used to identify immune cell populations as described in Supplementary Figure 4a. Live cells were identified by DAPI exclusion. Cell population frequencies are reported as percentages of the parent gate as indicated in the corresponding figures.

Gating strategy

Cells were initially gated on FSC and SSC parameters to identify singlets and exclude debris. Dead cells were excluded by DAPI positivity. CD45 was used to identify the total immune cell population. Downstream lineage populations were identified using the antibody panel as described in the methods and depicted in the gating strategy figure provided in the Supplementary Information.

☒ Tick this box to confirm that a figure exemplifying the gating strategy is provided in the Supplementary Information.

Magnetic resonance imaging

Experimental design

Design type

Indicate task or resting state; event-related or block design.

Design specifications

Specify the number of blocks, trials or experimental units per session and/or subject, and specify the length of each trial or block (if trials are blocked) and interval between trials.

Behavioral performance measures

State number and/or type of variables recorded (e.g. correct button press, response time) and what statistics were used to establish that the subjects were performing the task as expected (e.g. mean, range, and/or standard deviation across subjects).

Acquisition

Imaging type(s)

Specify: functional, structural, diffusion, perfusion.

Field strength

Specify in Tesla

Sequence & imaging parameters

Specify the pulse sequence type (gradient echo, spin echo, etc.), imaging type (EPI, spiral, etc.), field of view, matrix size, slice thickness, orientation and TE/TR/flip angle.

Area of acquisition

State whether a whole brain scan was used OR define the area of acquisition, describing how the region was determined.

Diffusion MRI ☐ Used ☐ Not used

Preprocessing

Preprocessing software

Provide detail on software version and revision number and on specific parameters (model/functions, brain extraction, segmentation, smoothing kernel size, etc.).

Normalization

If data were normalized/standardized, describe the approach(es): specify linear or non-linear and define image types used for transformation OR indicate that data were not normalized and explain rationale for lack of normalization.

Normalization template

Describe the template used for normalization/transformation, specifying subject space or group standardized space (e.g. original Talairach, MNI305, ICBM152) OR indicate that the data were not normalized.

Noise and artifact removal

Describe your procedure(s) for artifact and structured noise removal, specifying motion parameters, tissue signals and physiological signals (heart rate, respiration).

Volume censoring

Define your software and/or method and criteria for volume censoring, and state the extent of such censoring.

Statistical modeling & inference

Model type and settings

Specify type (mass univariate, multivariate, RSA, predictive, etc.) and describe essential details of the model at the first and second levels (e.g. fixed, random or mixed effects; drift or auto-correlation).

Effect(s) tested

Define precise effect in terms of the task or stimulus conditions instead of psychological concepts and indicate whether ANOVA or factorial designs were used.

Specify type of analysis: ☐ Whole brain ☐ ROI-based ☐ Both

Statistic type for inference

(See Eklund et al. 2016)

Specify voxel-wise or cluster-wise and report all relevant parameters for cluster-wise methods.

Correction

Describe the type of correction and how it is obtained for multiple comparisons (e.g. FWE, FDR, permutation or Monte Carlo).

Models & analysis

n/a	Involved in the study
☐	☐ Functional and/or effective connectivity
☐	☐ Graph analysis
☐	☐ Multivariate modeling or predictive analysis

Functional and/or effective connectivity	*Report the measures of dependence used and the model details (e.g. Pearson correlation, partial correlation, mutual information).*
Graph analysis	*Report the dependent variable and connectivity measure, specifying weighted graph or binarized graph, subject- or group-level, and the global and/or node summaries used (e.g. clustering coefficient, efficiency, etc.).*
Multivariate modeling and predictive analysis	*Specify independent variables, features extraction and dimension reduction, model, training and evaluation metrics.*
