# Supplementary material for: Peritoneal ovarian cancer growth in the omentum proceeds independently of mature adipocytes
Source: Nat Commun. 2026 Aug 12;17:9673. doi: 10.1038/s41467-026-76471-x (PMC13558732; doi:10.1038/s41467-026-76471-x)
Supplement: Supplementary file 4 [file 41467_2026_76471_MOESM5_ESM.pdf]

nature portfolio

Peer Review File

Peritoneal ovarian cancer growth in the omentum proceeds independently of mature adipocytes

Corresponding Author: Dr Gwendalyn Randolph

This file contains all reviewer reports in order by version, followed by all author rebuttals in order by version.

Version 0:

Reviewer comments:

Reviewer #1

(Remarks to the Author)
The main finding of the work is that omental adipocytes are dispensable for ovarian cancer metastasis formation in the peritoneum. This finding stands in contrast to the long held belief in the field that adipocytes are crucial in fuelling ovarian cancer metastatic growth in the omentum. Additionally, the observation of omental tropism of peritoneal metastases is supported by these findings, as removing the adipocyte-free omentum decreased tumor expansion in the peritoneum, underlining the role of the omentum in peritoneal metastases independent of its adipocyte content. Several additional findings are presented:
- OC metastases seed toward adipocyte-containing regions in the peritoneum.
- Adipocytes do not affect therapeutic efficacy in OC metastasis in the peritoneum.
- NK cells and lymphocytes do not impact OC metastases growth in the peritoneum. Neither in the presence or absence of adipocytes.
- Metabolic syndrome does not affect OC metastases growth in the peritoneum.
- FABP4-KO in endothelial cells decreases OC metastases growth in the peritoneum. In addition, high FABP4 expression in omental endothelial cells could explain the organotropism of OC metastases towards the omentum. While previous research highlighted the importance of FABP4, the data here presented identifies a different source.
- A CD36+FABP4+ endothelial cell population similar to the population described in mice is also present in the human omentum.

Will the work be of significance to the field and related fields? How does it compare to the established literature? If the work is not original, please provide relevant references.

The work is of considerable significance to the field and related fields. The work successfully challenges the long-held notion that adipocytes drive OC peritoneal metastasis formation. The line of evidence supporting this previous notion is well-explained in the introduction. The finding that adipocytes are dispensable for metastasis in peritoneal metastasis of ovarian cancer is highly relevant. Furthermore, this finding may motivate researchers to re-asses the evidence concerning the role of adipocytes in cancer progression in other types of cancer as well.
In addition, the authors suggest an alternative mechanism: their data points to omental endothelial cells, via their high expression of the fatty acid transporter FABP4, as the key drivers of the omentum's pro-tumorigenic niche. The authors strengthen their central claim by systematically investigating other, possibly confounding, factors—such as response to chemotherapy , the presence of metabolic syndrome , or the activity of lymphocytes and NK cells.
Does the work support the conclusions and claims, or is additional evidence needed?
In general, the work presents strong evidence for the conclusions drawn, supported by relevant and elegant models of disease, yet some questions still remain.
The text mentions explains clearly that mature adipocytes are depleted. One questions of interest would be if any pre-adipocytes could take over the functions of these mature adipocytes. It might be a good idea to explain from what point in adipocyte differentiation the adiponectin promotor is active, and if depletion from that point is sufficient to exclude any adipocyte-related effects. Another question that could be important is whether FABP4 is expressed before adiponectin during the adipocyte differentiation process. If so, pre-adipocyte FABP4 could potentially compensate for/still play a role in the seeding and expansion of ovarian cancer metastases in the peritoneum.
The core model involves mice that congenitally lack mature adipocytes. One question that could be answered more completely is whether any developmental compensations have occurred in other cell types. E.g., are there any significant

changes in the gene expression of endothelial cells in PARC vs DARL mice. (We recommend a differential gene expression analysis of endothelial cells comparing PARC and DARL mice.) Is the expression of FABP4 in ECs the same in these two mouse models?

Similarly, one could ask whether the phenotype seen in Tie2-Cre+Fabp4fl/fl mice is a direct effect of FABP4 depletion in ECs, or if FABP4 depletion causes changes in EC development, which then in turn affects peritoneal tumor expansion. The authors propose that FABP4 may regulate angiogenesis or facilitate fatty acid transport to tumor cells, but these possibilities were not directly tested in the knockout models. While this presents a limitation to the current study, this limitation is adequately addressed in the discussion.

The study's central claim that adipocytes are "dispensable" is very strong. While the evidence shows that tumor growth proceeds without mature adipocytes, this doesn't entirely negate any possible role for them. The results could indicate that the function of adipocytes (e.g., providing lipids) is redundant and can be compensated for by other sources, such as the FABP4-expressing endothelium.

A final limitation of the study is the limited human data. The study is strengthened by the inclusion of scRNA-seq data of a human omentum. In this scRNA-seq data, the authors find that the FABP4+ population is conserved across mice and humans. However, this analysis was performed on benign omental tissue. Moreover, it is unclear what the indication for omentectomy was in this patient. Was this harvested from a patient with ovarian cancer, yet without evidence of macroscopic or microscopic omental metastasis? This should be mentioned, particularly due to the potential remodeling of the omentum before evidence of clinical metastasis in the case of cancer; confounding potential comparison. Secondly, the presence of a similar EC population in the human omentum is weak evidence to suggest that the same dependability on ECs is present in human OC metastases to the omentum. A first step to strengthen this link would be the analyses of cancerous human tissue. This would also allow for an analysis of other microenvironmental factors to rationalise the observed omental tropism of ovarian cancer cells.

Are there any flaws in the data analysis, interpretation and conclusions? Do these prohibit publication or require revision?

Significant flaws in the statistical analyses. While we do not believe this will fundamentally change the conclusions, more appropriate statistical tests should be performed before publication.

Additionally, we have severe issues with the way annotation was performed in this dataset. Firstly, the choice of references is limited to benign single cell atlases of adipose tissue or lymph nodes, not relevant to the omentum. These atlases are unable to capture the tumor-specific changes implicit in peritoneal metastasis of ovarian cancer, while datasets of syngeneic models of ovarian cancer are freely available, such as Rodriguez et. Al (GSE183368). While the data shown here is not of a tumor model, the reference above would possibly be very interesting to include. Additionally, the authors were unable to reliably delineate CD45- phenotypes using the Capybara approach. Expert annotation, especially in the absence of high-quality reference data is irreplaceable, and should be performed in conjunction with automated annotation efforts. Additionally, marker genes and signatures should be visualized and mentioned in the text, or in supplementary data. Please revise. Furthermore, the annotation label of “cd45- stromal1-5” does not add anything to the data or its further analysis, demonstrating the inadequacy of this approach. Are these fibroblasts? Preadipocyte progenitors? Smooth muscle-like cells? This requires major revision.

Is the methodology sound? Does the work meet the expected standards in your field?

The methodology is generally sound. The use of the PARC and DARL mice provides a well thought out, thorough control to isolate local effects of adipocyte depletion from systemic (e.g., metabolic) effects. Some additional comments are mentioned below.

Is there enough detail provided in the methods for the work to be reproduced?

In general, the methods section provides sufficient detail for the work to be reproduced. Some additional comments are written below.

Additional comments on the text:

Minor formatting errors

The text contains still contains quite some double spaces (“ “). These can be easily replaced by single spaces using the replace function in word.

In some figure descriptions, some of the letters referring to the figure panels are not in bold. These include:

- Fig. 1h
- Fig. 4c

There are some instances were a preceding space is present before a superscript citation number. Consistent absence of a space would improve the manuscript’s overall polis.

- Linea 343: single-cell data and analysis 42,43
- Linea 348: cell type classification of Merrick et al. 44
- Line 946: APC cells isolated from SVF of naive mice 43

Introduction

Line 65: The nomenclature of a “mesothelial sheet” is uncommon. Moreover, it is common knowledge that anatomically the omentum is covered by four layers of peritoneum, folded over themselves. As the peritoneum consists of a mesothelial monolayer and a submesothelial stroma, this term can be confusing, as it is not clear what layers of the peritoneum are

implicated with this term. We would prefer the more anatomically sound nomenclature “peritoneal membrane” or even “peritoneum” to be used, as subsequent references to a “sheet region” or “membranous sheet region” are confusing to the readership.

Line 97-98: Instead, our data suggest that endothelial cells within the omental vasculature are the primary sources of the fatty acid transport protein FABP4.
This claim could be worded more accurately to avoid confusion.
- It can be taken to mean that ECs are the primary producer of FABP4 in the omentum. However, this claim is not fully supported. No direct comparison was made between FABP4 expression between ECs and adipocytes. To make such a claim one would need to consider the total production of FABP4 of adipocyte to the total FABP4 production of endothelial cells. (Such a comparison would be welcomed.)
- Secondly, the claim can be taken to mean that ECs directly provide FABP4 for tumor cells, which may fuel them through its function as a fatty acid transporter. However, this specific mechanism has not been demonstrated. The study's results do not exclude an indirect role for endothelial FABP4 in promoting tumor growth through other means, such as regulating angiogenesis.
The claim should be reworded to more accurately align with the data presented.
In line 746-748 a similar claim is made: Within the omentum, endothelial cells were the primary source of FABP4 expression, both in the presence and absence of mature adipocytes.
This claim seems to be primarily based on the scRNA-seq data of the PARC and DARL mice. However, this scRNA-seq data does not contain adipocytes. (as would be expected from scRNA-seq data).
This claim could be revised to more accurately state that endothelial cells were the primary producers of FABP4 among the successfully profiled non-adipocyte cell populations in both adipocyte-replete (PARC) and adipocyte-deficient (DARL) mice.
Material and methods
Murine single cell sequencing
Line 307: While there is certainly a point to be made for enrichment of specific populations prior to ScRNAseq, it is a choice that should be discussed further on in the paper, particularly due to the observation that in the final dataset, roughly two thirds of the cells are Cd45+. Is this due to the observed higher failure of hashing in CD45- cells, shown in supplementary data? From the text, it is not clear whether unbiased counts were roughly half CD45+, or if that is a result of enrichment in the sorting stage.
Furthermore, in the interest of reproducibility, more detail should be added, particularly on the methods used in data processing. How were counts normalized? What were the parameters set for Harmony? What resolution was the definitive object clustered at?
Line 332: Capybara Analysis and Custom Reference Generation
In general, this section lacks sufficient detail and justification for the choices made. (please see comments above)
Line 336: “for a couple of reasons”
Please revise this wording.
Additionally, there is no reference to supplementary method 1 detailing the annotation of Jackson-Jones et al. in the text. It Is also unclear what the relevance of this reference is, as it is not detailed in the methods section. However, used references 43,45 and 60 are detailed in the results section as used for Capybara analysis, yet not detailed in the methods section. Furthermore, reference 60 and 44 seem to be duplicate references. Please revise these sections.

Line 339: Following reference construction, quadratic programming, discrete cell identity classification, and multiple identity scoring were performed on our query dataset with each reference independently.
As a non-expert, I cannot follow this. Please briefly explain, in terms accessible to a broad readership, what these analytical steps achieve.
Line 374: Parametric statistical tests were used for statistical analysis. Thus, distributions were assumed to be normal, but this was not formally tested.
This is a significant issue. Parametric tests are only reliable if the data is normally distributed and has equal variances. Biological data with small sample sizes often does not follow a normal distribution. In several cases throughout the manuscript, it is far from obvious that the data would be normally distributed. In a few cases, the data is clearly not normally distributed (E.g., Figure 5h).
We recommend the authors re-evaluate the statistical analysis for each figure, including the extended data. It may be appropriate to do this with the help of a statistician. In instances where data distribution is not demonstrably normal, appropriate non-parametric alternatives (such as the Mann-Whitney U test or Kruskal-Wallis test) should be employed. While we anticipate this will strengthen, rather than alter, the study's main conclusions, this revision is essential to ensure the statistical validity of the findings.
Line 376: Statistical significance was evaluated using two-tailed unpaired Student’s t-tests or two-way ANOVAs followed by Tukey’s Honestly Significant Difference (HSD) Test for pairwise multiple comparisons. A Chi-Square Test was performed on the capybara distribution analyses for each reference to determine if the cell type distribution varied between genotypes (= 0.05).
The authors mention multiple testing correction (HSD) for post-hoc comparisons following the two-way ANOVAs, which is appropriate. However, in numerous instances across multiple figures, multiple individual t-tests are performed for single experiment. The methods and figure legends provide no indication that a correction for multiple comparisons was applied in these cases.
Performing multiple uncorrected t-tests inflates the probability of Type I errors (false positives). To ensure statistical rigor, we strongly recommend applying a standard correction (e.g., Bonferroni or Benjamini-Hochberg) to the p-values derived from all sets of multiple t-tests and updating the results accordingly.
Line 383: A p-value < 0.05 was considered statistically significant.
Following the proposed revisions outlined above, this line could be made more clear by stating “An adjusted p-value < 0.05

was considered statistically significant."
Results
Ovarian cancer spatially seeds regions of adiposity within peritoneal organs
Were tumor weights not measured? While tumoral burden can be approximated by luminescence, it is subject to considerable variability.

Line 398: Ovarian cancer spatially seeds regions of adiposity within peritoneal organs
Minor suggestion: the title of this section could be improved. The phrase "spatially seeds" seems a bit redundant. "Regions of adiposity" is a bit clunky and unnatural sounding.
Line 416: -the mesentery is also organized..-: Please use anatomically consistent nomenclature. The translucent cellular tissue sheets are peritoneum, consisting of a mesothelial monolayer and underlying stroma.
Line 419: Are FALCs and milky spots in the omentum only structurally similar, or are there key differences? Many authors state that these are one and the same, e.g.

Trim, W. V. & Lynch, L. Immune and non-immune functions of adipose tissue leukocytes. Nat Rev Immunol 22, 371–386 (2022).
As such, the distinction could be only an anatomical one.

Line 423: In just days after i.p. injection of ID8p53–/–Brca2–/– GFP Luc tumor cells in CD19Cre/+Tomato (TD) mice, …
It is not obvious why a CD19-reporter mouse line was chosen in the context of these experiments. This choice could be explained succinctly for clarity.
Line 425: What about mesothelial cells? They are assumed to be the "point of first contact" between cancer cells and the peritoneum, also in milky spots, as described by
Jackson-Jones, L. H. et al. Stromal Cells Covering Omental Fat-Associated Lymphoid Clusters Trigger Formation of Neutrophil Aggregates to Capture Peritoneal Contaminants. Immunity 52, 700-715.e6 (2020).
However, the potential role of mesothelial cells is completely disregarded in this description. Assuming cancer cells need to clear the peritoneal barrier before seeding into deeper adipocyte-rich layers, how does this fit within the observation of homing to adipocyte-rich regions, only days after injection of tumor cells? Furthermore, the role of mesothelial cells in peritoneal metastasis has been elegantly described by Bajwa et al., -cited by the authors at reference 68- using a DTA-ablation model in Msln-Cre mice.
Bajwa, P. et al. Cancer-associated mesothelial cell-derived ANGPTL4 and STC1 promote the early steps of ovarian cancer metastasis. JCI Insight e163019 (2023) doi:10.1172/jci.insight.163019.
These seemingly opposing findings should be discussed and contrasted. Additionally, histological evaluation of the omentum should be performed in an axis perpendicular to the peritoneal covering of the omentum, to adequately demarcate the deeper adipocyte-rich omentum and its peritoneal covering. This seems to not be the case in Fig. 1g and h. Ideally, these are additionally supplemented by representative histological images showing massing of cancer cells in the adipocyte-rich regions, rather than preferential attachment to mesothelial cells.

Mature adipocytes are not required for OC seeding and growth within the peritoneal cavity
This section, and the manuscript in general, focuses on the role of mature adipocytes. Context on the role of pre-adipocytes in the peritoneal cavity, and their potential role in OC seeding and growth might be helpful. Secondly, it might be important to clarify at what point during adipocyte development Adiponectin is expressed.

Line 453-455: It has been estimated that only 30% of cells in adipose tissue are mature adipocytes, and the lipoatrophic mice should still have a network of mesothelial cells, fibroblasts, endothelial cells, macrophages, stem cells, and pre-adipocytes that comprise the tissues in these regions.
The authors refer to the literature for this claim. However, this claim could be supported by the authors own data, e.g., based on simple IHC analyses of their model, or flow cytometry.
Line 452-455: To normalize these metabolic parameters while retaining a complete loss of mature adipocytes in the omentum and other tissues within the peritoneal cavity, we performed subcutaneous fat pad transplants into the scruff of the lipoatrophic mice, leaving the peritoneal cavity devoid of any mature adipocytes, while supplying an adipocyte-rich tissue to the mice systemically. Sham surgeries were also performed in the littermate control mice, which retained their standard fat depots in the peritoneal cavity
We commend the researchers for this crucial, well-done control that allows the researchers to isolate the local functions of adipocytes in the peritoneal cavity from the systemic metabolic effects.
Line 483: The KPCA tumors quickly saturated the bioluminescence read-out and thus were harvested at day 7 …
The authors' use of multiple tumor models strengthens their conclusions. However, regarding the KPCA model, the observation that tumors saturated the signal within 7 days is striking. Could such an aggressive growth rate overshadow any potential modulatory effects of the omental adipocytes on initial tumor establishment? It would be helpful for the authors to comment on whether this rapid progression might preclude the detection of a true biological difference that might manifest in a less aggressive system. Is such a rapid model still a biologically relevant model of ovarian PM?
Perhaps performing this experiment again with fewer injected cells could slow the tumor kinetics, providing a wider experimental window to observe any potential differences between the groups. At a minimum, this potential limitation warrants discussion.
Similarly, in both DARL and PARC cohorts, nearly 30% of the mesenteric fat region was occupied by tumors, while the sheet was virtually spared (Fig. 2h).
Line 504-505: Similarly, in both DARL and PARC cohorts, nearly 30% of the mesenteric fat region was occupied by tumors, while the sheet was virtually spared (Fig. 2h).
The manuscript's description of the mesothelial 'sheet' as a passive entity may understate its critical role in metastasis.

Additionally, it is assumed that to gain access to the deeper adipose-rich regions, the mesothelial barrier needs to be breached. How do the authors explain this distinction? This process involves dynamic processes like mesothelial cell retraction and the mesothelium's innate wound-healing response.

The discussion would be strengthened by acknowledging this active gatekeeper function. For instance, the finding that the 'sheet' is spared could be contextualized by framing it as a transient site of invasion through which tumor cells pass to reach the more hospitable niche underneath. This would provide a more complete and accurate mechanistic picture.

Natural killer cell and lymphocyte deficiency do not impact omentum-centric OC growth in the peritoneal cavity

Natural killer cell and lymphocyte deficiency do not impact omentum-centric OC growth in the peritoneal cavity

Line 549 : Manual flow cytometry gating (Extended Data Fig. 4a) was used to identify CD45+ immune populations overlaid on a dimensionality reduction plot, identifying 12 clusters (Fig. 3b).

Why was there no simple analysis of stromal cells performed? This could easily answer the question if the DARL model influences for example mesothelial cell behavior.

Line 563: we observed no difference in the overall rate of tumor expansion between WT and triple KO mice

This is a result that immediately raises questions, which should be addressed in more detail. Additionally, figure 2e does not specify these experiments were carried out in triple knockout mice, rather only indicating PARC and DARL. Should this be fig. 3e? Additionally, following depletion, why were there no additional analyses performed, including survival experiments or measurement of tumor weights? An effect on BLI is insufficient to rule out the role of the adaptive immune system. Was immunohistochemistry/flow cytometry of tumors to further delineate these effects performed? As omentectomy itself influences the progression of OC vastly, and the omentum itself is described as an immunologically extremely active organ, these findings seem to be discordant to that, and deserve further analysis. Furthermore, this finding would be strengthened by the addition of an biological replicate of this experiment using a different cell line, as both of these experiments were carried out using variants of the ID8 cell line, such as the other syngeneic models used by the authors in previous experiments.

Metabolic syndrome does not exacerbate OC growth in the peritoneum

No comments.

PARC and DARL mice have similar cell composition and high FABP4 endothelial expression in the omentum compared to the mesentery

Line 622: DARL mice were clustered based on differentially expressed genes in each cluster using canonical markers for specific populations (Fig. 5a).

What canonical markers were used? Which references? Please provide these in the form of f.e. a bubble plot.

Line 626: Was the use of single-nucleus strategies not considered, as these technologies are able to capture adipocyte heterogeneity?

Line 627 – line 642, and figure 5c and 5d (Capybara analysis): The current analysis of the CD45-negative stromal populations is difficult to interpret. The use of the Capybara tool with three different reference datasets results in three distinct and seemingly inconsistent cell identity annotations (Figure 5d). This raises a critical concern: the identity of these crucial stromal populations remains ambiguous. While the likely intent was to demonstrate that cell composition is stable regardless of the reference used, this approach undermines confidence in what these cell clusters truly represent.

- We strongly suggest the authors perform a more robust, integrated annotation of the stromal clusters. (This could involve a careful manual annotation using established canonical markers, informed by the results from all three Capybara comparisons.) The authors should attempt to arrive at a more confident and robust set of cell identities.
- The authors should clarify the rationale for presenting three separate classifications. If the primary goal is to show compositional stability between PARC and DARL mice, this should be explicitly stated as the objective. However, this does not substitute for a clear and consistent annotation of the cell types being discussed.

In addition, there is a discrepancy between the text and data in fig 5:

- The text (line 634) states, "...there were no meaningful proportional shifts in any identified stromal cell population between the PARC and DARL tissues".
- The data in Figure 5c, however, clearly shows a statistically significant difference ($p<0.05$) in the proportion of the "CD45- Stromal 1" cluster when comparing PARC and DARL mice.
- The data in Figure 5d shows that there are statistically significant shifts between cell type distributions across conditions. To consider these shifts “not meaningful” seems subjective.

The authors must address this discrepancy. Please either amend the statement in the text to reflect the data or provide a clear biological justification for why this statistically significant shift is not considered "meaningful" in the context of the study. The current analysis focuses on whether the number of cells in each cluster is similar across conditions. However, a stable cell composition does not necessarily mean the cells are functionally equivalent. It is crucial to investigate whether the cells in the DARL mice have an altered transcriptional state compared to those in the PARC mice. To significantly strengthen the findings, we recommend the authors perform differential gene expression (DGE) analysis within the key stromal clusters (e.g., "CD45- Stromal 1-5") by comparing the PARC and DARL conditions. This would reveal potential changes in cellular function or signaling pathways that are independent of cell abundance and would provide a much deeper insight into the impact of lacking mature adipocytes on the tumor microenvironment.

Line 653 :Extended data fig 6j: this is not provided in the manuscript. Please revise.

Endothelial FABP4 promotes omental tumor burden and defines a conserved $FABP4^{+}CD36^{+}$ vascular population

Previous claims were shown with 3 distinct cell lines, if there is data about the additional cell lines, it might be good to include them to show that the effect is not cell line specific.

Line 694-line 699: Thus, endothelial cells of the omentum in both mice and humans express elevated mRNA encoding FABP4 and CD36. FABP4 within these cells promotes tumor growth in the omentum. When these data are combined with the evidence that we provided here that omental adipocytes do not affect the pace of OC growth in the omentum or peritoneal cavity, it seems reasonable to conclude that earlier implications that FABP4 promoted ovarian tumor growth in omentum may relate to its key role in the vasculature, rather than the adipocyte.

While including human scRNA-seq data is a commendable effort to establish relevance, the manuscript overstates its implications. The suggestion that endothelial FABP4 functionally promotes tumor growth in humans is a significant extrapolation, as the current data are purely descriptive and derived from benign, non-cancerous tissue. This evidence shows the conservation of a cell type but does not prove its function in human disease. (It might be of interest to go through the publicly available scRNA-seq datasets to see if any datasets are available of human omental tissue of OC patients.)
To improve scientific rigor, the authors should temper their conclusion. It would be more accurate to state that they have identified a conserved endothelial population in humans that, based on their strong murine data, represents a compelling candidate for future investigation into its role in the human tumor microenvironment.
We suggest he authors attempt to further strengthen clinical relevance of their findings in the following ways:
- Tissue analysis: Use IHC or spatial transcriptomics on human omental metastases to demonstrate the co-localization (and enrichment) of FABP4+ endothelial cells with tumor nodules.
- Functional Assays: Perform in vitro co-culture experiments using human (omental) endothelial cells and ovarian cancer cells to directly test for FABP4-dependent effects on lipid transfer, proliferation, or migration.
Discussion
The discussion is well-written and crucially suggests two potential explanations for the link between endothelial FABP4 and OC progression. To further strengthen the manuscript, the authors could expand on the potential limitations of the Adiponectin-Cre model used to ablate adipocytes.
A key point for clarification is the developmental timing of gene expression during adipogenesis. The Adiponectin promoter becomes active relatively late, primarily in mature, lipid-laden adipocytes. In contrast, FABP4 expression is known to begin earlier in the differentiation process.
This timing difference creates a potential developmental window where adipocyte progenitors or immature adipocytes could be FABP4 -positive but Adiponectin-Cre -negative. Consequently, these cells would survive the DTA-mediated ablation in the DARL mice. The authors should discuss whether this surviving population of early, FABP4-expressing cells within the adipocyte lineage could still contribute to tumor growth. Acknowledging this as a potential caveat would provide a more nuanced interpretation of the results.
Line 746: Within the omentum, endothelial cells were the primary source of FABP4 expression, both in the presence and absence of mature adipocytes.
See the previous comment (introduction). No direct evidence for this claim is shown. This claim seems to be primarily based on the scRNA-seq data of the PARC and DARL mice. However, this scRNA-seq data does not contain adipocytes. (as would be expected from scRNA-seq data).
This claim could be revised to more accurately state that endothelial cells were the primary producers of FABP4 among the successfully profiled non-adipocyte cell populations in both adipocyte-replete (PARC) and adipocyte-deficient (DARL) mice.

Figures:
Figure 1
Some of the images are very illustrative of the points being made. The location of the figures is not entirely logical (with panel g and h being at the top of the figure). Re-organizing the figure might make it easier for the reader to follow along. However, we understand it's not always possible given the space each panel takes.
Panel a and b: We recommend to add the individual data points to the bar chart. This increases transparency by revealing the data distribution.
Panel e: For the left panel, maybe add CD19 (in red) and ID8 (in green) on the figure as well additional clarity.
Figure 2
Panel b:
- We strongly suggest showing the data distribution by adding the individual data points to the bar chart.
- Multiple t-test are performed. Based on the text it is unclear if p-values were adjusted for multiple comparisons.
Figure 2c,d: these images are not clear, and tissue orientation is unclear. Please provide a higher magnification image, with clear orientation (e.g. cut perpendicular to the surface).

Panel f and g:
- How this particle analysis works is not very clear to me. By eye, I am not able to detect the tumors corresponding to the masks. This analyses should be explained in more detail in the figure legend or material and methods sections. Because I do not understand how the masks were obtained, and because it is not immediately obvious to what they correspond, the masks are unconvincing.
Panel h: Multiple t-test are performed. Based on the text it is unclear if p-values were adjusted for multiple comparisons. Also really not obvious that a normal distribution can be assumed for percentage data clustering near 0% or 100%.
Panel i: Would not assume normal distribution here. Also correct for multiple testing.
Panel j:
- I would suggest making it clearer on the graph which significances are the result of which groups being compared. For example, by re-organizing the legend and adding a small bar between PARC and PARC omentectomy with the appropriate significance.
- It is also not clear why only 2x a two-tailed t test has been done in this graph, why in K and l, a two-way anova has been used followed by multiple comparisons with p-value correction. The latter method seems more appropriate for panel j as well.
Figure 3:
Panel c: Suggestion: It's not easy to interpret these pseudocolor heatmaps. I wonder if splitting the UMAP from figure 3b would not provide a clearer picture?
The bar charts in figure 4d are very clear. Perhaps providing a stacked bar chart is an option?
Panel d: Multiple comparisons. Perhaps an ANOVA (or non-parametric alternative) followed by a post-hoc test for multiple comparisons might be a more appropriate test.

Panel e: The figure legend mentions that the statistical test was not significant. We suggest to also add this to the graph.
Panel g: The figure legend mentions that the statistical test was not significant. We suggest to also add this to the graph.

Figure 4:
Panel a: The figure legend mentions that the statistical test was not significant. We suggest to also add this to the graph.
Panel c:
- Write c in bold in the figure legend.
- Since the different anatomical locations add up to 100%, you are dealing with proportional data. Using a t-test on proportional data might not be appropriate, because the values over the anatomical locations are not independent (if the percentage in one location is higher, the percentage in another location must be lower).
- It’s also necessary to correct for multiple comparions.
Panel d: Again, not obvious that for percentage data near 100% or 0%, the data would be normally distributed. Non-parametric tests + correction for multiple testing might be more appropriate.
Figure 5:
Panel a: I would suggest increasing the font size of the cell types a little bit to make the legend more readable.
Panel c: P-values should be adjusted for the multiple t-tests done here. It is not clear there has been an appropriate correction for multiple testing. A non-parametric Mann-Whitney U test might be a safer choice if you are unsure if the data follows a normal distribution.
Panel d: This figure is very unclear to me. If I have understood it correctly, the scRNA-seq data was automatically annotated using Capybara based on 3 different references. However, Using each reference, a completely different annotation for the CD45- stromal cells was achieved. classifiers classify your data with very different results. It’s very confusing what the point of this analyses is and what conclusions I should draw. It seems to me that you require a very good, annotated reference for Capybara to work properly. I feel that the fact that these different references lead to such different results underlines that you actually have no idea of the identity of the stromal cells in your sample. We strongly suggest that the authors perform a more robust, manual annotation of the data. Is the point that independent of the reference, no strong changes were seen in cell type composition? Line 634 mentions “there were no meaningful proportional shifts in any identified stromal cell population between the PARC and DARL tissues (Fig. 5d), including pre-adipocyte and adipocyte progenitor populations, that may stimulate matrix remodeling to support OC metastasis61”. However, panel c in Fig. 5 clearly shows a statistically significant shift for “CD45- Stromal 1” when comparing PARC and DARL mice.
Panel h:
- Does the graph show (normalized) log-transformed counts, raw counts, please indicate unit of the y axis?
- The data distribution is clearly not normal here. While there seems to be a clear difference between endothelial Fabp4 expression in the mesentery compared to the omentum, a t-test is not appropriate here. A non-parametric test, e.g. Mann-Whitney U test, would be more appropriate here.
- If a comparison is done between endothelial cells and other immune and non-immune subtypes, as claimed in the figure legend, please show this comparison on your figure and adjust your p-values.

Figure 6:
Panel a, panel b, panel c: Please mention the statistical test used per panel. Be sure to correct for multiple testing if appropriate.
Panel e and g: Two endothelial cell clusters are shown in panels e and g. Is there a significant difference between them (e.g., vascular vs lymphatic)?
Fig. 6f: the ubiquitous presence of igkc in most clusters indicates a potential contamination with ambient RNA. We recommend ambient rna correction to be used for these data.

Reviewer #2

(Remarks to the Author)
I co-reviewed this manuscript with one of the reviewers who provided the listed reports. This is part of the Nature Communications initiative to facilitate training in peer review and to provide appropriate recognition for Early Career Researchers who co-review manuscripts.

Reviewer #3

(Remarks to the Author)
In manuscript “Adipocytes are dispensable in shaping the ovarian cancer tumor microenvironment in the omentum”, Mintz et al. study the role of omental adipocytes in OC. They show that OC cells retain a propensity to seed regions typically associated with adipocytes in mice even without mature adipocytes. However, adipocyte-free omentum was found to be important for OC expansion. Endothelial cell-selective deficiency of FABP4 reduced OC growth in the peritoneum. The authors conclude that that endothelial cells within the omental vasculature are the primary sources of the fatty acid transport protein FABP4.

The study improves understanding of the dynamic crosstalk between AT and tumor.

However, there are problems with data interpretation and missing controls, which limit this work.

Major Comments:

The importance of vasculature is well understood, and specifically for OC (studies by Anil Sood and others). FABP4, aka

adipocyte protein 2 (AP2), is a carrier protein for fatty acids that is primarily expressed in preadipocytes, adipocytes and macrophages. Silencing FABP4 in tumor endothelium has been previously shown to suppresses OC growth and peritoneal/omental metastatic burden in mouse models as cited (89,90). This limits novelty.
CD36 in the endothelium is also well known to mediate lipid transport (cite doi: 10.1172/jci.insight.147057) , so, not much is new there either.

The main issue is that Tie2-Cre was used for KO in EC. This model also KOs in hematopoietic cells. A cleaner, CDH5-Cre (inducible) or Tie2e-Cre would be better models for EC.

Tie2-Cre KOs FABP4 in macrophages, the other main expressing cell type. This issue is alleviated by LysM-Cre control KO that does not show the phenotype. However, Yorek M et al. (eLife 2024) showed that macrophage FABP4 deficiency reduces FA-driven lipid metabolism and metastasis. Therefore, macrophage FABP4 mediates lipid transport important for cancer progression. This key study is not cited / discussed and it is not clear how this discrepancy can be reconciled.

Also, adipose stromal / progenitor cells (ASCs) are known to express FABP4 and it shows in Fig. 5. FABP4 KO in them would also be important to perform for comparison.

As is, the main conclusion on the pivotal source of FABP4 cannot be made.

Lipoatrophic mice are well known to have hypertriglyceridemia, hypercholesterolemia, hyperglycemia and Hyperinsulinemia. Therefore, although adipocytes are missing from microenvironment, the reason tumor still grow well is probably because lipids are abundant from circulation. This is not fully addressed by AT transplants in the PARC / DARL model.

Other Comments:

In Figure 5 and gene table, nine of the top ten significantly increased genes are involved in immunoglobulin production and immune cell activation (Ighv's, Cxcl5). While overall immune cell counts may be unchanged (Figure 3), immune cell function may be altered.

The authors should measure changes to other fat depots such as gonadal fat. In Extended Data Figure 3, gonadal fat pads of DARL mice appear to be significantly smaller versus those of PARC
Mice. While the transplanted fat pad increases in size in DARL mice, the proximity of the gonadal fat depot to the primary tumor may cause it to be preferentially mobilized by the tumor versus the transplant fat pad.

Additionally, staining to visualize lipids within tumors (eg., oil red o) would be helpful to determine if lipid uptake is indeed altered in tumors of adipocyte depleted and Fabp4 knockout mice.

Did the authors measure other Fabp such as Fabp5? In the gene expression table Fabp5 is significantly increased while Fabp4 and Cd36 are decreased. This suggests the possibility of compensation by Fabp5 in the absence of Fabp4 and should be discussed.

Technical issue: Photon Flux bioluminescence signal can come from either more small tumors or fewer bigger tumors. It is not clear if/how this is reconciled throughout.

Manuscript needs proofreading.

“while” is missing after “However,”: “However, the lack of mature adipocytes did not suppress peritoneal OC expansion, whereas removing the adipocyte-free omentum did.”

Other ‘bugs’, such as: “Capybara function construct.high.res.reference().”

Inter-section spaces are missing, eg. Page 15.

Line 230: I think the authors mean FABP4 not “FABBP4”

Reviewer #4

(Remarks to the Author)
This is a provocative manuscript which challenges the concept that ovarian cancer cells metastasize to adipocytes in the peritoneal cavity (in particular to the omentum) and take up lipids by these (somehow mediated by FABP4 in adipocytes). The authors use a variety of mouse models to show that the lack mature adipocytes does not prevent metastasis to the omentum and subsequent tumor growth. Indeed the cancer cells invade the mature adipocyte-free microenvironment of white adipose tissue within the omentum. Several cell types could be involved in this process, e.g. immune cells. It is known that there is often strong immunosuppression in ovarian cancer. The authors tested the contribution of several cell types but did not find a responsible one. However, the authors detected that endothelial cells express high levels of FABP4 (which is not a new finding) and that deleting FABP4 in endothelial cells using the Tie2-Cre mouse leads to decreased tumor growth in the omentum. Tie2-Cre also targets some myeloid cells. The authors addressed this by deleting FABP4 in such a myeloid

cell population and this had no significant effect on tumor growth. As such this is an important contribution to the field and raises several questions.

The authors addressed the potential confounders using lipoatrophic mice by implanting subcutaneous fat. This is an excellent strategy. Together with surgery to remove the omentum it gives a strong and convincing model.

Major points:

1) The authors used Tie2-Cre Fabp4 deficient mice. Ideally, they would have used VE-Cadherin (Cdh5)-Cre mice to address this issue. I strongly advice the authors to consider this.

2) The endothelial Fabp4 deficient mice need a much deeper investigation. How does this approach alter the vascular bed within WAT and the omentum? What about vessel density, vessel coverage, permeability, perfusion, and perivascular immune cell composition? Maybe the whole vessel network is already disorganized at the time when cancer cells are injected. This may compromise the whole experiment.

3) What could be the function of FABP4 in endothelial cells? Is is needed for handling fatty acids? If yes from where are the fatty acids transported? Blood, other perivascular cells? How are lipid levels in these mice? Alternatively, it could be fatty acid independent functions of FABP4. This needs much deeper investigation.

4) The effect of endothelial Fabp4 on tumor growth (Fig 6A) is not very strong. It seems that tumor growth in knockout mice just starts a few days later but then tumor growth rates are similar. Please calculate this. The authors should compare this effect with the global Fapb4 deletion in the landmark Nat Med 2011 paper (ref 9).

5) Fapb4 alone and also in combination with CD36 is probably not sufficient to elevated transendothelial lipid transport. What about other crucial components such as lipases in the mouse models? Ideally, the authors should measure transport of circulating labeled lipids to tumor cells.

6) Does Fabp4 deletion affect the morphology of the mesothelium? Has it been shown that this Cre line does is not active in these cells.

7) Regarding the potential effects of immune cells for the observed phenotype. How does the lack of mature adipocytes alter the polarization of macrophages.

Version 1:

Reviewer comments:

Reviewer #1

(Remarks to the Author)
I appreciate the substantive efforts by the authors in addressing the questions and suggestions of the reviewers, particularly in delineating the role of FABP4 in adipocyte differentiation, and additional histological evaluation as detailed in figures 1 and 2. The result is a novel, interesting and well-performed study of interest to the readership.

There are, however, still several formatting errors to parse, particularly in referring to figures 6 and 7. I suggest to check these, as these diminish an otherwise well-written manuscript.

I have no additional comments.

Reviewer #2

(Remarks to the Author)
I co-reviewed this manuscript with one of the reviewers who provided the listed reports. This is part of the Nature Communications initiative to facilitate training in peer review and to provide appropriate recognition for Early Career Researchers who co-review manuscripts.

Reviewer #3

(Remarks to the Author)
The authors have done a lot of work to address the original critiques. The manuscript is greatly improved now. In discussion, the paper on the role of endothelial fatty acid transporter CD36 in cancer needs to be cited: doi: 10.1172/jci.insight.147057. Also, needs to be cited a comprehensive recent review on the roles of adipose tissue lipids in carcinoma progression doi: 10.1016/j.trecan.2025.11.012.

Reviewer #4

(Remarks to the Author)
The authors have substantially improved the manuscript. Remaining questions:

Concerning my previous comment #2. Does the endothelial knockout of FABP4 affect immune cell composition within the abdominal cavity? Since growth of ovarian cancer and metastases are strongly influenced by the immune response, this outstanding question should be answered. The new single cell data shown in Fig. 5 could be potentially be used to answer this question.

Concerning comment #3. The authors should add the cholesterol and triglyceride measurement into the manuscript. In addition, they should further explore endothelial Bodipy C16 uptake, at least under basal (non-tumor) conditions.

Open Access This Peer Review File is licensed under a Creative Commons Attribution 4.0 International License, which permits use, sharing, adaptation, distribution and reproduction in any medium or format, as long as you give appropriate credit to the original author(s) and the source, provide a link to the Creative Commons license, and indicate if changes were made.
In cases where reviewers are anonymous, credit should be given to 'Anonymous Referee' and the source.
The images or other third party material in this Peer Review File are included in the article's Creative Commons license, unless indicated otherwise in a credit line to the material. If material is not included in the article's Creative Commons license and your intended use is not permitted by statutory regulation or exceeds the permitted use, you will need to obtain permission directly from the copyright holder.
To view a copy of this license, visit https://creativecommons.org/licenses/by/4.0/

Nature Communications Review

Reviewer #1 (Remarks to the Author):

The main finding of the work is that omental adipocytes are dispensable for ovarian cancer metastasis formation in the peritoneum. This finding stands in contrast to the long held belief in the field that adipocytes are crucial in fuelling ovarian cancer metastatic growth in the omentum. Additionally, the observation of omental tropism of peritoneal metastases is supported by these findings, as removing the adipocyte-free omentum decreased tumor expansion in the peritoneum, underlining the role of the omentum in peritoneal metastases independent of its adipocyte content. Several additional findings are presented:
- OC metastases seed toward adipocyte-containing regions in the peritoneum.
- Adipocytes do not affect therapeutic efficacy in OC metastasis in the peritoneum.
- NK cells and lymphocytes do not impact OC metastases growth in the peritoneum. Neither in the presence or absence of adipocytes.
- Metabolic syndrome does not affect OC metastases growth in the peritoneum.
- FABP4-KO in endothelial cells decreases OC metastases growth in the peritoneum. In addition, high FABP4 expression in omental endothelial cells could explain the organotropism of OC metastases towards the omentum. While previous research highlighted the importance of FABP4, the data here presented identifies a different source.
- A CD36+FABP4+ endothelial cell population similar to the population described in mice is also present in the human omentum.

Will the work be of significance to the field and related fields? How does it compare to the established literature? If the work is not original, please provide relevant references.

The work is of considerable significance to the field and related fields. The work successfully challenges the long-held notion that adipocytes drive OC peritoneal metastasis formation. The line of evidence supporting this previous notion is well-explained in the introduction. The finding that adipocytes are dispensable for metastasis in peritoneal metastasis of ovarian cancer is highly relevant. Furthermore, this finding may motivate researchers to re-asses the evidence concerning the role of adipocytes in cancer progression in other types of cancer as well. In addition, the authors suggest an alternative mechanism: their data points to omental endothelial cells, via their high expression of the fatty acid transporter FABP4, as the key drivers of the omentum's pro-tumorigenic niche. The authors strengthen their central claim by systematically investigating other, possibly confounding, factors—such as response to chemotherapy , the presence of metabolic syndrome , or the activity of lymphocytes and NK cells.

Does the work support the conclusions and claims, or is additional evidence needed?
In general, the work presents strong evidence for the conclusions drawn, supported by relevant and elegant models of disease, yet some questions still remain.

(1) The text mentions explains clearly that mature adipocytes are depleted. One questions of interest would be if any pre-adipocytes could take over the functions of these mature adipocytes. It might be a good idea to explain from what point in adipocyte differentiation the adiponectin promoter is active, and if depletion from that point is sufficient to exclude any adipocyte-related effects.

In the Adiponectin-Cre × Rosa-DTA model, Cre activity is initiated exclusively in terminally differentiated adipocytes, as adiponectin expression is restricted to mature adipocytes and is not active in adipocyte progenitors or earlier lineage stages (doi: 10.1038/s42255-019-0041-z, doi: 10.4161/adip.29674, doi: 10.1093/jmcb/mjw011). Upon activation of the adiponectin promoter, diphtheria toxin A is expressed, resulting in cell-autonomous ablation of mature adipocytes. Adipocyte progenitors do not express adiponectin and are therefore not directly targeted. Although pre-adipocytes remain present, they do not restore mature adipocyte functions such as adipokine secretion or lipid storage capacity. Thus, this model specifically

eliminates differentiated adipocytes while preserving earlier lineage cells, enabling direct testing of whether mature adipocytes themselves are required for peritoneal tumor growth. We have revised the Results, Discussion, and Methods to explicitly define the differentiation stage targeted by Adiponectin-Cre and to consistently use "mature adipocytes" throughout the manuscript. Supporting visualization of adiponectin expression patterns has been included in Extended Data Fig. 6h.

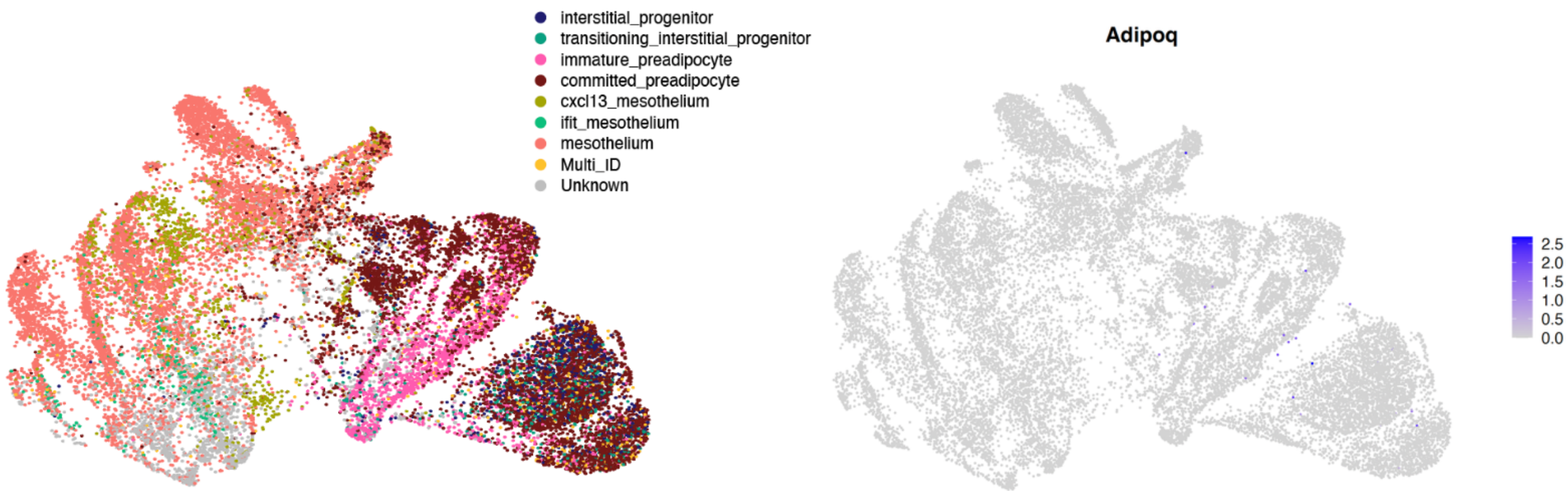

(2) Another question that could be important is whether FABP4 is expressed before adiponectin during the adipocyte differentiation process. If so, pre-adipocyte FABP4 could potentially compensate for/still play a role in the seeding and expansion of ovarian cancer metastases in the peritoneum.

FABP4 is induced earlier than adiponectin during adipogenesis, as it is reported to be expressed in a population of committed pre-adipocytes and early differentiating adipocytes (doi: 10.1096/fj. 12-211516). In our system, we examined Fabp4 expression across stromal populations in our single-cell RNA sequencing dataset. UMAP visualization and quantitative analysis (Extended Data Fig. 6h) demonstrated that Fabp4 expression outside the endothelial compartment was sparse and low-level. Only 5.8% of non-endothelial stromal cells exhibited detectable Fabp4 transcripts (Extended Data Fig. 6h). The major subcluster expressing *Fabp4* in this feature plot grouping, however, co-expressed low levels of *Pecam* encoding CD31 (Extended Data Fig. 6h). Thus, we cannot be sure that these cells do not represent a small fraction of endothelial cells that we had tried to analyze separately. In the Adiponectin-Cre × Rosa-DTA model, any adipocyte-lineage cell that progresses to terminal differentiation and activates the adiponectin promoter is constitutively ablated, preventing the persistence of mature adipocytes. Although earlier adipocyte-lineage cells remain present, the scarcity of FABP4^{+} stromal cells and the absence of a tumor phenotype in lipoatrophic mice together suggest that pre-adipocyte Fabp4 expression is unlikely to compensate for the loss of mature adipocytes in driving peritoneal tumor growth. The manuscript has been revised to consistently distinguish mature adipocytes from earlier cells of the adipocyte lineage. To proceed as cautiously as possible, we have added a sentence in the discussion that we cannot eliminate a role for preadipocytes carrying out functions that are independent of lipid storage and mobilization.

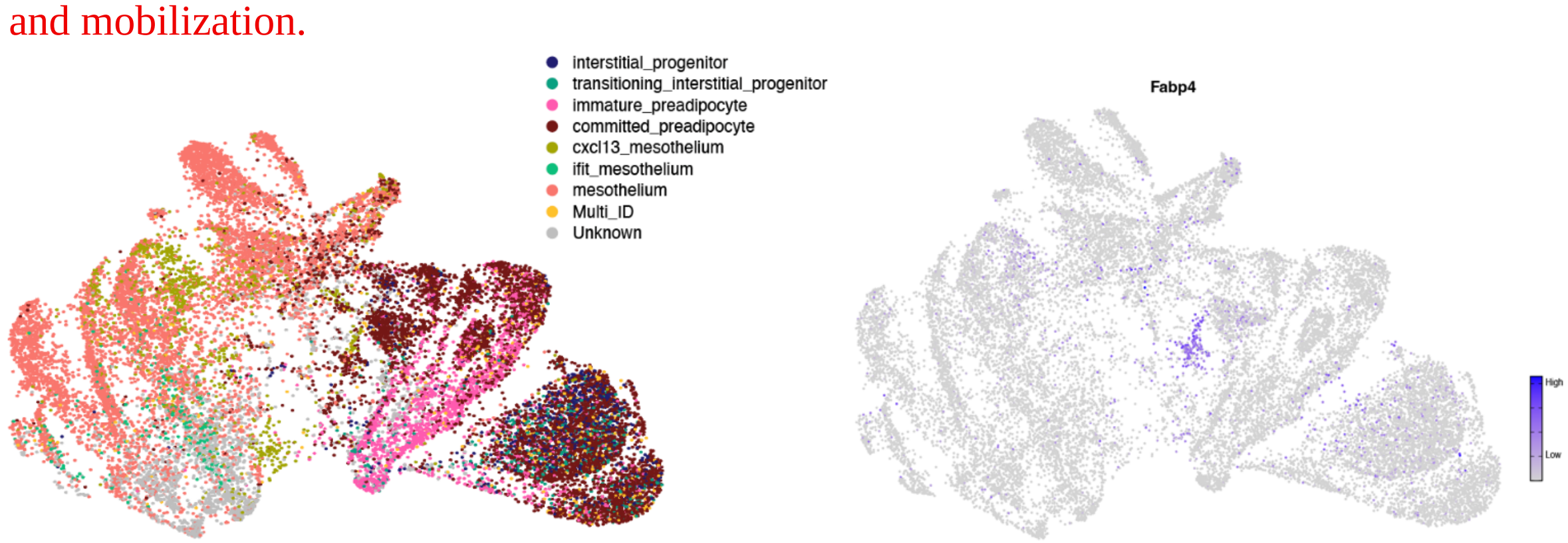

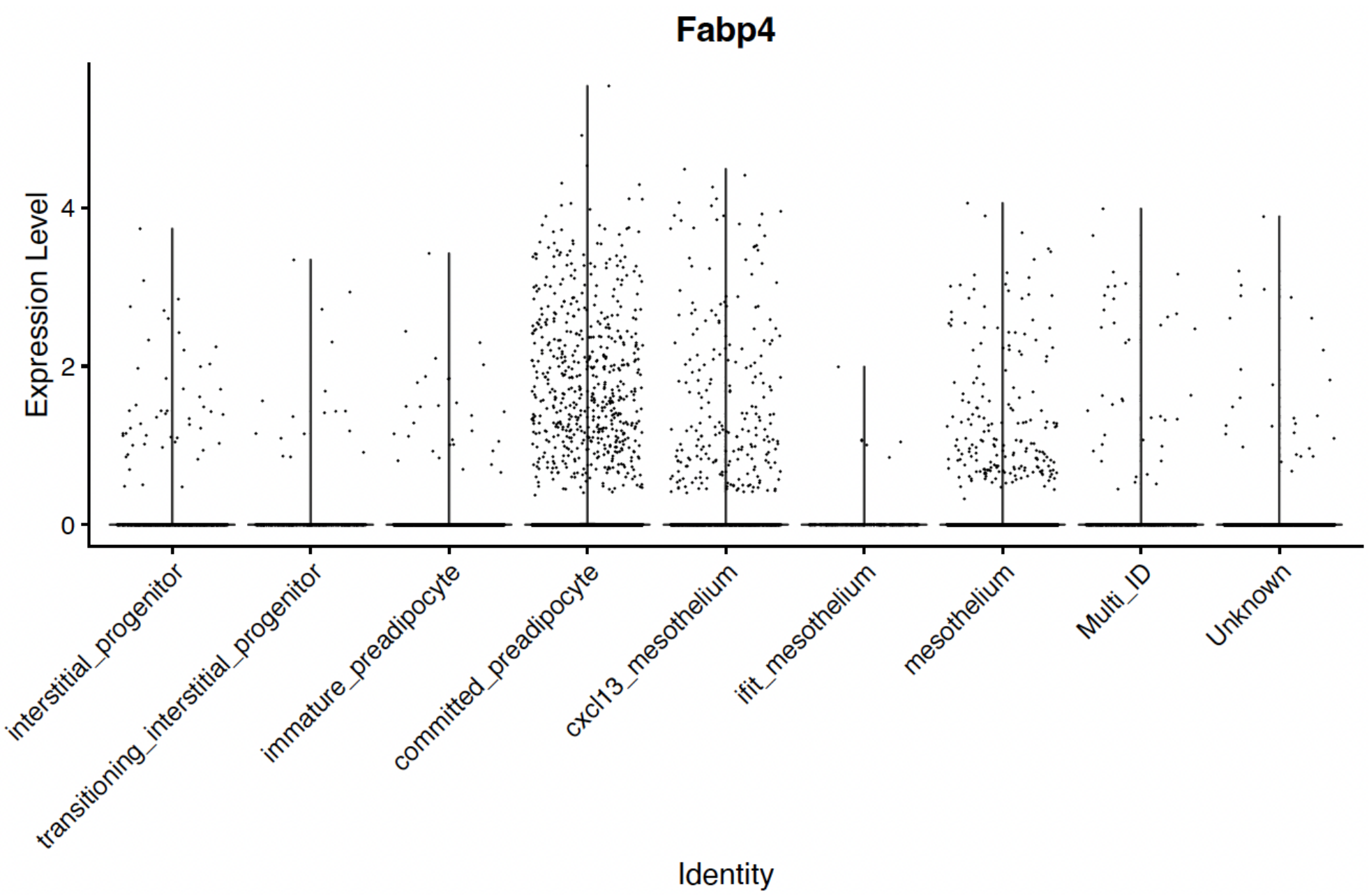

(3) The core model involves mice that congenitally lack mature adipocytes. One question that could be answered more completely is whether any developmental compensations have occurred in other cell types. E.g., are there any significant changes in the gene expression of endothelial cells in PARC vs DARL mice. (We recommend a differential gene expression analysis of endothelial cells comparing PARC and DARL mice.) Is the expression of FABP4 in ECs the same in these two mouse models?

The overall proportion of endothelial cells is comparable between PARC and DARL mice (Fig. 5). Sub-clustering using established endothelial atlases identified a modest shift in capillary endothelial composition in DARL mice, with relative enrichment of CapEC1 and reduction of CapEC2 populations. The magnitude of this shift is limited and does not indicate large-scale endothelial remodeling. Differential gene expression analysis revealed statistically significant but modest transcriptional differences, including genes associated with tissue-adapted endothelial identity, immune or stress-associated transcripts, and metabolic enzymes. We have provided the DEG table comparison with the revision for your review. The statistical significant changes do not form a coherent compensatory pathway suggestive of endothelial reprogramming substituting for the absence of mature adipocytes. With respect to FABP4 specifically, the proportion of FABP4-expressing endothelial cells is similar in PARC and DARL mice (76.2% versus 66.7%; log2FC −1.29), and FABP4 does not emerge as a defining feature of DARL endothelium. Tumor burden is unchanged between PARC and DARL mice, indicating that these endothelial transcriptional differences are unlikely to be functionally driving tumor growth. Together, these data argue against a major compensatory endothelial program in mice lacking mature adipocytes from birth.

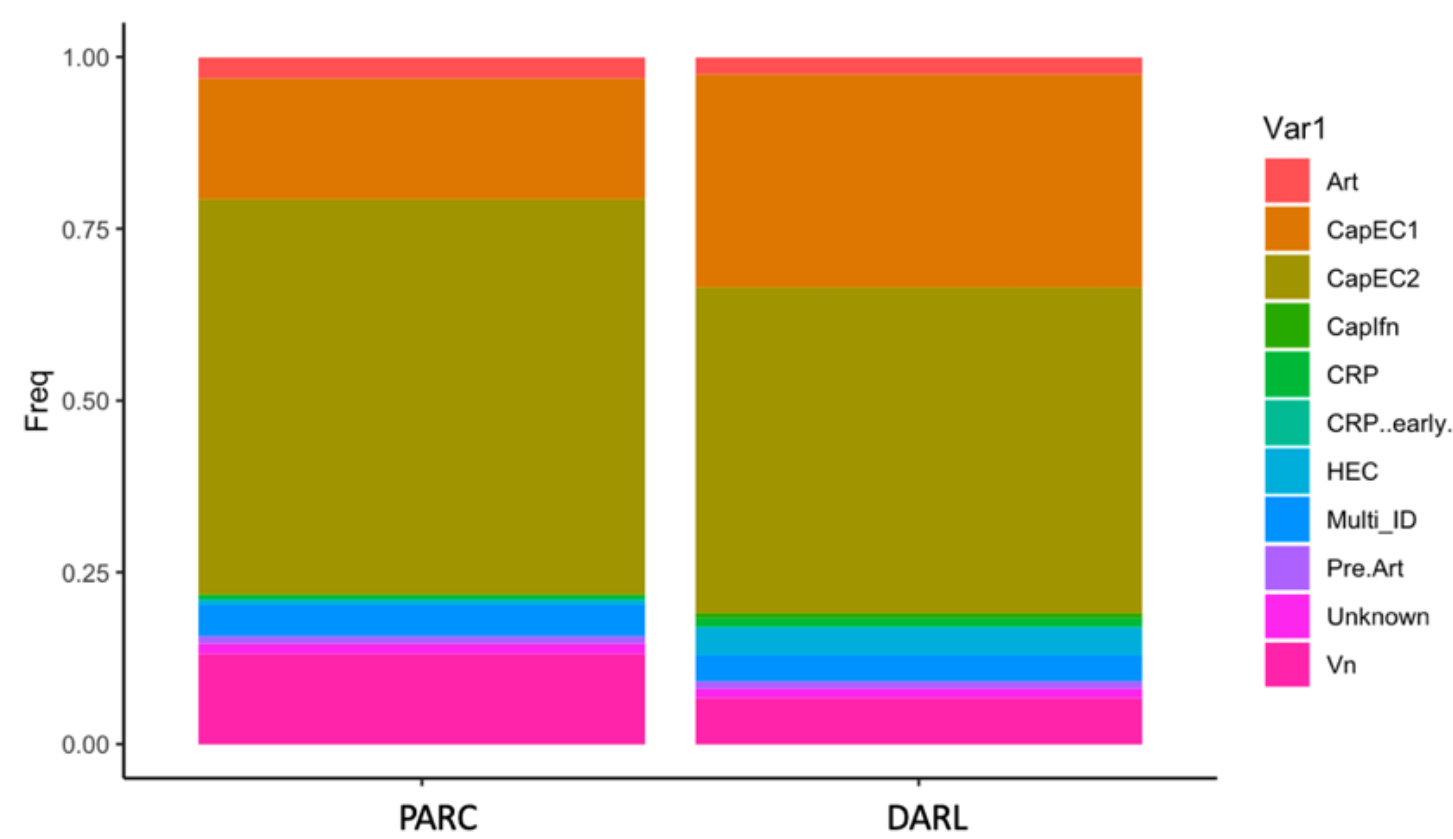

(4) Similarly, one could ask whether the phenotype seen in Tie2-Cre+Fabp4$^{fl/fl}$ mice is a direct effect of FABP4 depletion in ECs, or if FABP4 depletion causes changes in EC development, which then in turn affects peritoneal tumor expansion. The authors propose that FABP4 may regulate angiogenesis or facilitate fatty acid transport to tumor cells, but these possibilities were not directly tested in the knockout models. While this presents a limitation to the current study, this limitation is adequately addressed in the discussion.

We appreciate the reviewer's suggestion here. We were spurred to add more data to address this point and have extended the scope of the study to do so. Three-dimensional whole-mount imaging of tumor-naïve omentum in Tie2-Cre^{+} Fabp4$^{fl/fl}$ and littermate controls (Fig. 6d–j) demonstrated preserved baseline vascular architecture, arguing against developmental vascular defects resulting from endothelial Fabp4 deletion. However, in an exciting turn of events, in tumor-bearing omentum, endothelial Fabp4 deficiency was associated with reduced vascular branching and altered tumor-associated vascular remodeling. These data support an important context-dependent role for endothelial FABP4 during tumor-associated remodeling rather than a primary developmental defect. In addition to revising Results and Figures to include these new findings, we have clarified these points in the Discussion.

(5) The study's central claim that adipocytes are "dispensable" is very strong. While the evidence shows that tumor growth proceeds without mature adipocytes, this doesn't entirely negate any possible role for them. The results could indicate that adipocyte function (e.g., lipid provision) is redundant and can be compensated for by other sources, such as the FABP4-expressing endothelium.

The reviewer makes a good point and adds just the right qualifying word – *mature*. Yes, we agree that what we have shown is that tumor growth proceeds without *mature* adipocytes. It is true that they may still have a role but we do show that they are not essential, which is what we meant by "dispensable." We agree that our findings do not exclude the possibility that adipocyte-associated functions may be redundant within the peritoneal microenvironment or partially compensated for by other stromal cell types, including endothelial cells. To avoid overstatement, we have revised the manuscript throughout to replace language implying absolute dispensability with more precise wording ("not required") and have modified the title accordingly.

(6a) A final limitation of the study is the limited human data. The study is strengthened by the inclusion of scRNA-seq data of a human omentum. In this scRNA-seq data, the authors find that the FABP4+ population is conserved across mice and humans. However, this analysis was performed on benign omental tissue. Moreover, it is unclear what the indication for omentectomy was in this patient. Was this harvested from a patient with ovarian cancer, yet without evidence of macroscopic or microscopic omental metastasis? This should be mentioned, particularly due to the potential remodeling of the omentum before evidence of clinical metastasis in the case of cancer; confounding potential comparison.

(6b) Secondly, the presence of a similar EC population in the human omentum is weak evidence to suggest that the same dependability on ECs is present in human OC metastases to the omentum. A first step to strengthen this link would be the analyses of cancerous human tissue. This would also allow for an analysis of other microenvironmental factors to rationalize the observed omental tropism of ovarian cancer cells.

We have now added additional human data to substantially strengthen this point.

The human omentum sample was obtained during surgical staging for a patient with a granulosa cell tumor of the ovary. The omentum appeared grossly normal intra-operatively, and final pathology confirmed benign tissue with no macroscopic or microscopic tumor involvement; the primary tumor was confined to the ovary. While subtle pre-metastatic remodeling cannot be formally excluded, this specimen lacked histologic evidence of tumor-associated architectural or cellular reprogramming. Given that granulosa cell tumors typically present as localized disease and rarely involve the omentum at diagnosis, we consider this tissue a

reasonable representation of non-metastatic human omentum for identifying conserved endothelial and stromal populations. These details have been added to the Methods and referenced in the Results.

To strengthen translational relevance as per the reviewer's suggestion, we now incorporate a reanalysis of a recently published, spatially resolved single-cell RNA sequencing dataset of human omentum that includes benign tissue, non-metastasized omentum from ovarian cancer patients, and established omental metastases (Lischetti et al., Nature Communications, 2025). This dataset enabled spatial evaluation of endothelial populations across tumor-proximal, peritumoral, and distal regions. The $FABP4^{+}CD36^{+}$ blood endothelial population identified in murine models is conserved in human omentum, detectable prior to metastatic colonization, and retained within metastatic lesions with increased transcriptional heterogeneity. These findings support cross-species conservation of this endothelial program and strengthen the biological plausibility of an endothelial contribution to omental metastasis. The Results and Discussion have been revised accordingly.

Lischetti U, Liang C-Y, Carrara M, et al. Ovarian cancer metastasis to the human omentum disrupts organ homeostasis and induces fundamental tissue reprogramming. *Nature Communications*. 2025/12/16 2025;doi:10.1038/s41467-025-67557-z

Are there any flaws in the data analysis, interpretation and conclusions? Do these prohibit publication or require revision?

Significant flaws in the statistical analyses. While we do not believe this will fundamentally change the conclusions, more appropriate statistical tests should be performed before publication.

(7) Additionally, we have severe issues with the way annotation was performed in this dataset. Firstly, the choice of references is limited to benign single cell atlases of adipose tissue or lymph nodes, not relevant to the omentum. These atlases are unable to capture the tumor-specific changes implicit in peritoneal metastasis of ovarian cancer, while datasets of syngeneic models of ovarian cancer are freely available, such as Rodriguez et. Al (GSE183368). While the data shown here is not of a tumor model, the reference above would possibly be very interesting to include.

Our single-cell annotation strategy was intentionally designed to define the baseline cellular composition of the omental microenvironment in naïve PARC and DARL mice, prior to tumor implantation. Accordingly, we prioritized reference atlases derived from benign tissues rather than tumor-bearing models, as our goal was to identify intrinsic stromal and endothelial features that might predispose to altered tumor growth, rather than to characterize tumor-induced remodeling. We agree that tumor-associated remodeling can profoundly alter cellular states. However, because our subsequent functional experiments demonstrated comparable tumor growth between PARC and DARL mice, the present study did not aim to interrogate tumor-driven transcriptional reprogramming or metastatic niche evolution. These important questions extend beyond the scope of the current work.

In addition to adipose and lymphoid references, we included a published murine omentum single-cell dataset (Jackson-Jones et al., 2020), now explicitly labeled in the text and Figure 5 legend for clarity. We acknowledge that few benign murine omentum datasets exist with sufficient stromal resolution, which necessarily limits reference selection. In response to the reviewer's comment, we have removed the inguinal fat pad reference (Merrick et al., 2019), as it did not materially improve annotation depth. We retained the pre-adipocyte dataset (Rivera-Gonzalez and Butka, 2025), given the persistence of pre-adipocytes in the Adiponectin-Cre × DTA model and their relevance to our comparisons.

We appreciate the suggestion to incorporate tumor-bearing murine ovarian cancer datasets, such as Rodriguez et al. (GSE183368). While this resource is valuable for studying tumor-associated remodeling, its annotation framework does not align with the finer stromal and endothelial distinctions examined here. Rather than expanding murine tumor references, we instead incorporated spatially resolved human metastatic omentum data to enhance translational relevance. We have cited Rodriguez et al. in the Discussion as an important resource for future comparative analyses across syngeneic models.

(8) Additionally, the authors were unable to reliably delineate CD45- phenotypes using the Capybara approach. Expert annotation, especially in the absence of high-quality reference data is irreplaceable, and should be performed in conjunction with automated annotation efforts. Additionally, marker genes and signatures should be visualized and mentioned in the text, or in supplementary data. Please revise.

Initial annotation was performed using canonical lineage markers and established biological knowledge to define major stromal and immune populations. Capybara was subsequently applied in conjunction with curated, tissue-relevant reference datasets to increase annotation granularity, particularly for mesothelial and adipocyte progenitor populations. We have revised the Methods to clarify this sequential strategy and to specify that automated annotation was used to complement marker-based interpretation.

To improve transparency, we have reorganized Figure 5 to provide clearer stromal cell annotations (Fig. 5a) and added explicit visualization of the marker genes and signatures underlying each UMAP-defined population. A dot plot summarizing key lineage-defining genes is now included (Fig. 5b), and additional confirmation of assignments based on canonical markers is provided in Extended Data Fig. 5h. These revisions clarify the delineation of $CD45^{-}$ phenotypes and make the supporting marker evidence directly accessible in the figures.

(9) Furthermore, the annotation label of “cd45- stromal1-5” does not add anything to the data or its further analysis, demonstrating the inadequacy of this approach. Are these fibroblasts? Preadipocyte progenitors? Smooth muscle-like cells? This requires major revision.

We agree that the prior label “$CD45^{-}$ stromal 1–5” lacked biological specificity. In response, we performed differential gene expression analysis within this compartment and reassessed canonical marker expression to refine lineage identities. These populations have now been reclassified into fibroblasts and mesothelial cells in the revised UMAP (Fig. 5a), with further sub-classification using Capybara shown in Fig. 5d. Marker gene support for these assignments is provided in Fig. 5b and Extended Data Fig. 6h.

Is the methodology sound? Does the work meet the expected standards in your field?
The methodology is generally sound. The use of the PARC and DARL mice provides a well thought out, thorough control to isolate local effects of adipocyte depletion from systemic (e.g., metabolic) effects. Some additional comments are mentioned below.

Is there enough detail provided in the methods for the work to be reproduced?
In general, the methods section provides sufficient detail for the work to be reproduced. Some additional comments are written below.

Additional comments on the text:

Minor formatting errors
(1) The text contains still contains quite some double spaces (“ “). These can be easily replaced by single spaces using the replace function in word.
We thank the reviewer for this observation. All instances of double spaces in the manuscript have now been corrected and replaced with single spaces.

(2) In some figure descriptions, some of the letters referring to the figure panels are not in bold. These include:
- Fig. 1h
- Fig. 4c

Thank you for catching this. All letters in the legends are now uniformly bolded.

(3) There are some instances were a preceding space is present before a superscript citation number. Consistent absence of a space would improve the manuscript’s overall polish.

- Linea 343: single-cell data and analysis 42,43
- Linea 348: cell type classification of Merrick et al. 44
- Line 946: APC cells isolated from SVF of naive mice 43

We appreciate the reviewer's attention to detail. The unnecessary spaces before superscript citation numbers have been removed. We have reviewed the manuscript to ensure that such spacing inconsistencies are corrected throughout.

(4) Introduction
Line 65: The nomenclature of a "mesothelial sheet" is uncommon. Moreover, it is common knowledge that anatomically the omentum is covered by four layers of peritoneum, folded over themselves. As the peritoneum consists of a mesothelial monolayer and a submesothelial stroma, this term can be confusing, as it is not clear what layers of the peritoneum are implicated with this term. We would prefer the more anatomically sound nomenclature "peritoneal membrane" or even "peritoneum" to be used, as subsequent references to a "sheet region" or "membranous sheet region" are confusing to the readership.

We agree that the term "mesothelial sheet" is not standard anatomical nomenclature and may introduce ambiguity. The greater omentum consists of a four-layered fold of peritoneum, with each layer comprising a mesothelial monolayer and underlying submesothelial stroma. We have revised the Introduction to more accurately reflect this anatomy and have replaced "mesothelial sheet" with the anatomically appropriate terms "peritoneum" or "peritoneal membrane," as applicable, throughout the manuscript.

(5) Line 97-98: Instead, our data suggest that endothelial cells within the omental vasculature are the primary sources of the fatty acid transport protein FABP4.
This claim could be worded more accurately to avoid confusion.
- It can be taken to mean that ECs are the primary producer of FABP4 in the omentum. However, this claim is not fully supported. No direct comparison was made between FABP4 expression between ECs and adipocytes. To make such a claim one would need to consider the total production of FABP4 of adipocyte to the total FABP4 production of endothelial cells. (Such a comparison would be welcomed.)
- Secondly, the claim can be taken to mean that ECs directly provide FABP4 for tumor cells, which may fuel them through its function as a fatty acid transporter. However, this specific mechanism has not been demonstrated. The study's results do not exclude an indirect role for endothelial FABP4 in promoting tumor growth through other means, such as regulating angiogenesis.
The claim should be reworded to more accurately align with the data presented.

Thank you for this important comment. We agree that the original phrasing was overly broad and could be interpreted beyond the scope of our data. We have revised the manuscript to clarify this point.

Because droplet-based single-cell RNA sequencing does not efficiently capture mature adipocytes due to their size and fragility, mature adipocytes were not recovered in our datasets, including PARC samples. We now explicitly state this limitation and therefore do not claim that endothelial cells represent the dominant source of total FABP4 in the omentum. Rather, we clarify that among the non-adipocyte populations successfully profiled, endothelial cells and macrophages exhibited the highest Fabp4 expression, with endothelial cells representing the predominant FABP4-expressing stromal population within the analyzed compartment.

We have also revised the text to avoid implying a direct lipid transfer mechanism from endothelial cells to tumor cells. While endothelial Fabp4 deletion reduced early tumor expansion and altered tumor-associated vascular remodeling (see our new data in Fig. 6) , our data do not establish that endothelial FABP4 directly provides fatty acids to tumor cells. Potential indirect mechanisms, including effects on vascular or angiogenic function, are now discussed as future directions in the Discussion

(6) In lines 746-748, a similar claim is made: Within the omentum, endothelial cells were the primary source of FABP4 expression, both in the presence and absence of mature adipocytes.

This claim seems to be primarily based on the scRNA-seq data of the PARC and DARL mice. However, this scRNA-seq data does not contain adipocytes. (as would be expected from scRNA-seq data).
This claim could be revised to more accurately state that endothelial cells were the primary producers of FABP4 among the successfully profiled non-adipocyte cell populations in both adipocyte-replete (PARC) and adipocyte-deficient (DARL) mice.

We agree that the original phrasing could be misinterpreted, as droplet-based scRNA-seq does not reliably capture mature adipocytes due to their size and fragility. Our intent was to indicate that, among the cell populations successfully profiled, endothelial cells consistently represented the predominant source of Fabp4 transcripts in both PARC (adipocyte-replete) and DARL (adipocyte-deficient) omenta. We have revised the manuscript to explicitly state that this conclusion applies to the analyzed non-adipocyte compartment and does not reflect total FABP4 production within the tissue.

Material and methods
Murine single cell sequencing
(7) Line 307: While there is certainly a point to be made for enrichment of specific populations prior to ScRNAseq, it is a choice that should be discussed further on in the paper, particularly due to the observation that in the final dataset, roughly two thirds of the cells are Cd45+. Is this due to the observed higher failure of hashing in CD45- cells, shown in supplementary data? From the text, it is not clear whether unbiased counts were roughly half CD45+, or if that is a result of enrichment in the sorting stage.

$CD45^-$ cells were sorted first to prioritize recovery of the stromal compartment, which can be more fragile during tissue dissociation and sorting. However, sorting was performed to completion, and all viable $CD45^-$ and $CD45^+$ cells passing quality thresholds were collected. Final cell numbers were therefore not determined by sorting order or intentional enrichment.

The approximately 2:1 $CD45^+$:$CD45^-$ ratio observed in the final dataset likely reflects native tissue composition together with known differences in recovery efficiency between immune and stromal populations during dissociation and library preparation, rather than selective exclusion of $CD45^-$ cells or differential hashing efficiency. As shown in Extended Data Fig. 6f, hashtags 1–8 were detected in both $CD45^-$ and $CD45^+$ populations, arguing against systematic hashing failure in stromal cells.

We have revised the Methods to more clearly describe the sorting strategy and added clarification in the Results to guide interpretation of the observed cell-type proportions.

(8) Furthermore, in the interest of reproducibility, more detail should be added, particularly on the methods used in data processing. How were counts normalized? What were the parameters set for Harmony? What resolution was the definitive object clustered at?

We have expanded the Methods section describing murine single-cell RNA sequencing to provide full details of data processing and integration. Specifically, we now describe the normalization strategy for count data, the parameters used for Harmony batch correction (including the variables used for integration and convergence settings), and the selected clustering resolution.

(9) Line 332: Capybara Analysis and Custom Reference Generation
In general, this section lacks sufficient detail and justification for the choices made. (please see comments above)

We have revised the Capybara Analysis and Custom Reference Generation section to provide additional methodological detail and justification for our annotation strategy. The Methods now describe construction of the custom reference objects, parameters used for label transfer, and the rationale for increasing annotation granularity within mesothelial and adipocyte-progenitor compartments. We also explicitly justify selection of the two reference datasets based on their tissue relevance and stromal resolution.

(10) Line 336: "for a couple of reasons"
Please revise this wording.
Additionally, there is no reference to supplementary method 1 detailing the annotation of Jackson-Jones et al. in the text. It Is also unclear what the relevance of this reference is, as it is not detailed in the methods section. However, used references 43,45 and 60 are detailed in the results section as used for Capybara analysis, yet not detailed in the methods section. Furthermore, reference 60 and 44 seem to be duplicate references. Please revise these sections.

We have revised the wording in line 336 to remove the phrase "for a couple of reasons." Supplementary Method 1 is now explicitly referenced in the Methods section, and we have clarified the relevance of the Jackson-Jones et al. dataset, noting its tissue specificity to murine omentum and its inclusion of mesothelial subdivisions not captured in our initial classification.

We have also ensured that all references used for Capybara analysis (including refs. 43, 45, and 60) are described in the Methods section, in addition to the Results. Duplicate references (44 and 60) have been corrected, and the Rivera-Gonzalez et al. citation has been updated from the preprint to the published Cell Stem Cell article.

(11) Line 339: Following reference construction, quadratic programming, discrete cell identity classification, and multiple identity scoring were performed on our query dataset with each reference independently.
As a non-expert, I cannot follow this. Please briefly explain, in terms accessible to a broad readership, what these analytical steps achieve.

We have revised this section to clarify the purpose and outcome of these analytical steps rather than focusing on the underlying mathematical implementation. Briefly, reference-based mapping assigns each query cell a quantitative similarity score to annotated reference populations based on transcriptome-wide expression patterns. Each cell is then classified according to the population it most closely resembles, while retaining the ability to detect cells with shared or intermediate features. Performing this analysis independently across curated references increases robustness and avoids forcing all cells into a single predefined taxonomy. We have simplified the manuscript text accordingly and added citations to the primary Capybara and reference atlas publications for readers seeking technical detail.

(12) Line 374: Parametric statistical tests were used for statistical analysis. Thus, distributions were assumed to be normal, but this was not formally tested.
This is a significant issue. Parametric tests are only reliable if the data is normally distributed and has equal variances. Biological data with small sample sizes often does not follow a normal distribution. In several cases throughout the manuscript, it is far from obvious that the data would be normally distributed. In a few cases, the data is clearly not normally distributed (E.g., Figure 5h). We recommend the authors re-evaluate the statistical analysis for each figure, including the extended data. It may be appropriate to do this with the help of a statistician. In instances where data distribution is not demonstrably normal, appropriate non-parametric alternatives (such as the Mann-Whitney U test or Kruskal-Wallis test) should be employed. While we anticipate this will strengthen, rather than alter, the study's main conclusions, this revision is essential to ensure the statistical validity of the findings.

We agree that parametric tests require appropriate distributional assumptions and that our original submission did not explicitly document formal normality testing. In the revision, we systematically re-evaluated all datasets used for hypothesis testing.

Normality was formally assessed using the Shapiro–Wilk test for small sample sizes and the D'Agostino–Pearson omnibus test where appropriate. In selected cases, Anderson–Darling testing and Kolmogorov–Smirnov testing with Lilliefors correction were also applied. We additionally examined residual distributions to assess symmetry and variance assumptions.

When normality was not supported ($P < 0.05$), sample sizes were small, or residuals were not approximately symmetric, we reanalyzed the data using nonparametric alternatives matched to the experimental

design, including the Mann–Whitney U test for two-group comparisons and the Kruskal–Wallis test with Dunn's correction for multi-group analyses. The statistical test, sample size, and multiple-comparison correction are now specified in each figure legend.

For longitudinal tumor growth experiments, we implemented area-under-the-curve analysis to reduce repeated-measures multiple testing and increase robustness. The revised statistical workflow was reviewed with a biostatistician (Jing Wang, PhD), and her contribution is acknowledged in the manuscript.

These reanalyses did not alter the primary conclusions. Although some P values shifted with the use of more conservative nonparametric tests, effect sizes and overall interpretations remained consistent.

(13) Line 376: Statistical significance was evaluated using two-tailed unpaired Student's t-tests or two-way ANOVAs followed by Tukey's Honestly Significant Difference (HSD) Test for pairwise multiple comparisons. A Chi-Square Test was performed on the capybara distribution analyses for each reference to determine if the cell type distribution varied between genotypes (a= 0.05).
The authors mention multiple testing correction (HSD) for post-hoc comparisons following the two-way ANOVAs, which is appropriate. However, in numerous instances across multiple figures, multiple individual t-tests are performed for single experiment. The methods and figure legends provide no indication that a correction for multiple comparisons was applied in these cases.
Performing multiple uncorrected t-tests inflates the probability of Type I errors (false positives). To ensure statistical rigor, we strongly recommend applying a standard correction (e.g., Bonferroni or Benjamini-Hochberg) to the p-values derived from all sets of multiple t-tests and updating the results accordingly.

We have reviewed all statistics across the figures and updated them to account for multiple comparisons. The figure legends have been updated to reflect the information. We do not believe that this has altered the interpretation of the data.

(14) Line 383: A p-value < 0.05 was considered statistically significant.
Following the proposed revisions outlined above, this line could be made more clear by stating "An adjusted p-value < 0.05 was considered statistically significant."

To avoid ambiguity, we have revised the phrasing to: *"An adjusted p-value < 0.05 was considered statistically significant."*

Results
(15) Ovarian cancer spatially seeds regions of adiposity within peritoneal organs
Were tumor weights not measured? While tumoral burden can be approximated by luminescence, it is subject to considerable variability.

Tumor weight was not used as a primary endpoint because, in disseminated peritoneal metastasis models, tumor burden consists of numerous small nodules adherent to peritoneal surfaces and organs and, in some cases, diffusely coating structures such as the omentum. Under these conditions, tumor mass cannot be measured reproducibly without weighing entire organs (introducing variability related to organ size and fluid accumulation) or manually dissecting innumerable embedded nodules, which is inherently subjective

We did measure whole-organ weights of the omentum and mesentery at steady state and in tumor-bearing mice (Fig. 2k) to illustrate baseline anatomical differences between genotypes and the overall increase in organ mass associated with tumor presence. However, whole-organ weight does not distinguish tumor tissue from native tissue or ascites-associated fluid and therefore was not used as a quantitative measure of tumor burden.

Instead, tumor burden was assessed using longitudinal bioluminescence imaging, which enables normalization across animals and captures both solid tumor and fluid-associated disease. Tumor localization and morphology were corroborated by stereomicroscopy and histological analysis (Fig. 2h,i). In addition, ex vivo organ-specific bioluminescence imaging normalized to tissue area, along with automated particle-based quantification, was used to objectively assess regional tumor burden. These approaches yielded consistent

results across independent experiments and tumor models. This rationale has been clarified in the Results and Methods.

(16) Line 398: Ovarian cancer spatially seeds regions of adiposity within peritoneal organs
Minor suggestion: the title of this section could be improved. The phrase “spatially seeds” seems a bit redundant. “Regions of adiposity” is a bit clunky and unnatural sounding.

We agree that this title could be improved. We have revised the section title to: “Adipose-rich regions within peritoneal organs are preferential sites of OC localization.”

(17) Line 416: -the mesentery is also organized..-: Please use anatomically consistent nomenclature. The translucent cellular tissue sheets are peritoneum, consisting of a mesothelial monolayer and underlying stroma.

We agree that “peritoneum” should be used consistently to refer to the translucent cellular sheet. We have revised the manuscript to replace “sheet(s)” with peritoneal membrane (peritoneum) and refer to the fat-rich areas as the adipose region. These changes were applied at line 416 and throughout the text, figure panels, and captions to ensure consistency.

(18) Line 419: Are FALCs and milky spots in the omentum only structurally similar, or are there key differences? Many authors state that these are one and the same, e.g. Trim, W. V. & Lynch, L. Immune and non-immune functions of adipose tissue leukocytes. Nat Rev Immunol 22, 371–386 (2022).
As such, the distinction could be only an anatomical one.

We have reviewed the cited literature and revised the text accordingly. We now state explicitly that omental milky spots are widely regarded as the omental manifestation of fat-associated lymphoid clusters (FALCs), consistent with Trim and Lynch (2022) and related primary studies. We have removed language implying functional distinction and retain only the well-supported observation that these aggregates expand in response to inflammatory challenge. The section has been revised to reflect shared features while maintaining the relevant anatomical context.

(20) Line 423: In just days after i.p. injection of ID8p53–/–Brca2–/– GFP Luc tumor cells in CD19Cre/+Tomato (TD) mice, …
It is not obvious why a CD19-reporter mouse line was chosen in the context of these experiments. This choice could be explained succinctly for clarity.

We selected the $CD19^{Cre/+}$Tomato reporter line because CD19 is a B-cell marker that reliably demarcates the lymphoid-rich milky spots of the omentum, key omental anatomical structures. This provided a means to visualize the omentum's underlying architecture and contextualize tumor localization relative to these structures. To improve clarity and avoid implying a functional role for B cells, we revised the text.

(21) Line 425: What about mesothelial cells? They are assumed to be the “point of first contact” between cancer cells and the peritoneum, also in milky spots, as described by
Jackson-Jones, L. H. et al. Stromal Cells Covering Omental Fat-Associated Lymphoid Clusters Trigger Formation of Neutrophil Aggregates to Capture Peritoneal Contaminants. Immunity 52, 700-715.e6 (2020).
However, the potential role of mesothelial cells is completely disregarded in this description. Assuming cancer cells need to clear the peritoneal barrier before seeding into deeper adipocyte-rich layers, how does this fit within the observation of homing to adipocyte-rich regions, only days after injection of tumor cells?
Furthermore, the role of mesothelial cells in peritoneal metastasis has been elegantly described by Bajwa et al., -cited by the authors at reference 68- using a DTA-ablation model in Msln-Cre mice.
Bajwa, P. et al. Cancer-associated mesothelial cell-derived ANGPTL4 and STC1 promote the early steps of ovarian cancer metastasis. JCI Insight e163019 (2023) doi:10.1172/jci.insight.163019.
These seemingly opposing findings should be discussed and contrasted.

Mesothelial cells are indeed a critical interface in peritoneal metastasis, and we agree that their role should be explicitly addressed. In our single-cell RNA-seq analysis of tumor-naïve omentum, mesothelial cells were identified and further subclassified using Capybara with a tissue-relevant reference (Fig. 5a,d). We quantified mesothelial populations across genotypes and observed no significant difference in mesothelial cell abundance between PARC and DARL mice at steady state. These analyses reflect baseline mesothelial composition rather than tumor-induced reprogramming.

Prior studies by Jackson-Jones et al. and Bajwa et al. demonstrate that mesothelial cells actively participate in early metastatic events through functional reprogramming following tumor cell contact, including secretion of ANGPTL4 and STC1. Our study addresses a distinct question: whether depletion of mature adipocytes alters the baseline cellular architecture of the omentum or its intrinsic capacity to support tumor seeding and growth.

Our findings indicate that removal of mature adipocytes does not alter mesothelial cell abundance and does not impair ovarian cancer seeding or expansion within the omentum. These observations are therefore complementary rather than contradictory. Mesothelial cells may serve as the initial peritoneal interface, while subsequent localization to adipocyte-rich regions represents a downstream spatial pattern of tumor distribution. We have revised the Results to clarify preservation of mesothelial populations between genotypes and expanded the Discussion to explicitly contrast our findings with prior mesothelial ablation studies.

(22) Additionally, histological evaluation of the omentum should be performed in an axis perpendicular to the peritoneal covering of the omentum, to adequately demarcate the deeper adipocyte-rich omentum and its peritoneal covering. This seems to not be the case in Fig. 1g and h. Ideally, these are additionally supplemented by representative histological images showing massing of cancer cells in the adipocyte-rich regions, rather than preferential attachment to mesothelial cells.

All histological sections were collected in an orientation perpendicular to the peritoneal surface, such that each section includes the mesothelial layer, underlying adipocyte-rich compartment, and associated stromal and vascular structures. We acknowledge that differences in magnification between steady-state and tumor-bearing images may have contributed to confusion. In tumor-bearing omenta, extensive architectural remodeling limits the ability to capture the entire organ within a single low-magnification field.

In response, we have expanded the histological analysis to include both low- and high-magnification H&E images of PARC and DARL omenta under steady-state and tumor-bearing conditions (Fig. 2c–f). These images more clearly delineate the mesothelial covering and deeper adipocyte-rich regions and demonstrate tumor massing within adipocyte-rich compartments rather than preferential attachment restricted to mesothelial surfaces. Section orientation has been clarified in the figure legends to avoid ambiguity.

(23) Mature adipocytes are not required for OC seeding and growth within the peritoneal cavity
This section, and the manuscript in general, focuses on the role of mature adipocytes. Context on the role of pre-adipocytes in the peritoneal cavity, and their potential role in OC seeding and growth might be helpful. Secondly, it might be important to clarify at what point during adipocyte development Adiponectin is expressed.

We have clarified the developmental stage at which adiponectin is expressed and expanded the discussion of pre-adipocyte populations.

Adiponectin expression is restricted to terminally differentiated, lipid-laden adipocytes and is not detected in adipocyte progenitors or early pre-adipocytes. Accordingly, the Adiponectin-Cre × Rosa-DTA model selectively ablates mature adipocytes while preserving earlier adipocyte-lineage populations.

To more directly address the potential contribution of pre-adipocytes, we extended our single-cell RNA sequencing analysis using reference-based annotation of adipocyte-lineage states. This enabled explicit identification of adipocyte progenitor and pre-adipocyte populations (Fig. 5d; Extended Data Fig. 6h). These populations were present in both PARC and DARL mice and were comparable between genotypes, with only a modest difference confined to an immature pre-adipocyte subset.

We have revised the Results and Discussion to distinguish mature adipocytes from earlier adipocyte-lineage states more clearly and to explicitly state that preserved pre-adipocyte populations do not compensate for the loss of mature adipocytes in supporting peritoneal tumor growth.

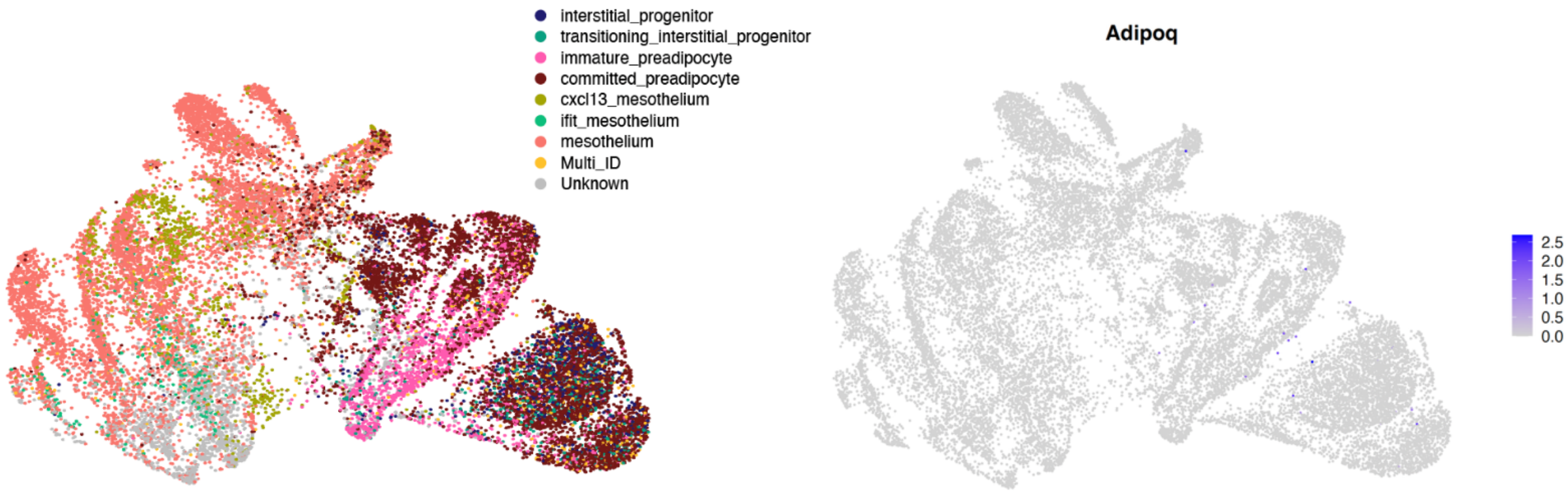

(24) Line 453-455: It has been estimated that only 30% of cells in adipose tissue are mature adipocytes, and the lipoatrophic mice should still have a network of mesothelial cells, fibroblasts, endothelial cells, macrophages, stem cells, and pre-adipocytes that comprise the tissues in these regions.
The authors refer to the literature for this claim. However, this claim could be supported by the authors own data, e.g., based on simple IHC analyses of their model, or flow cytometry.

The referenced statement was intended to provide general context that adipose tissue contains multiple stromal and immune populations beyond mature adipocytes. It was not meant to assert a specific quantitative proportion in our model. In the revised manuscript, we have simplified this section and retained the citation as background without implying numerical estimates.

Importantly, our single-cell RNA sequencing analysis directly demonstrates the presence of mesothelial cells, fibroblasts, endothelial cells, macrophages, and adipocyte-lineage progenitors in both PARC and DARL omenta. These populations are preserved following mature adipocyte depletion. Because our study was designed to test the functional requirement of mature adipocytes for tumor growth, rather than to comprehensively re-quantify all stromal compartments, we did not perform additional global compositional analyses by IHC or flow cytometry.

(25) Line 452-455: To normalize these metabolic parameters while retaining a complete loss of mature adipocytes in the omentum and other tissues within the peritoneal cavity, we performed subcutaneous fat pad transplants into the scruff of the lipoatrophic mice, leaving the peritoneal cavity devoid of any mature adipocytes, while supplying an adipocyte-rich tissue to the mice systemically. Sham surgeries were also performed in the littermate control mice, which retained their standard fat depots in the peritoneal cavity
We commend the researchers for this crucial, well-done control that allows the researchers to isolate the local functions of adipocytes in the peritoneal cavity from the systemic metabolic effects.

We sincerely thank the reviewer for this thoughtful and encouraging feedback. We agree that the fat pad transplantation experiments were an important control to help disentangle the local effects of adipocytes within the peritoneal cavity from systemic metabolic influences. These surgeries were technically demanding and required considerable effort, and we are grateful that the reviewer recognized their importance to the study's conclusions.

(26) Line 483: The KPCA tumors quickly saturated the bioluminescence read-out and thus were harvested at day 7 …
The authors' use of multiple tumor models strengthens their conclusions. However, regarding the KPCA model, the observation that tumors saturated the signal within 7 days is striking. Could such an aggressive growth rate

overshadow any potential modulatory effects of the omental adipocytes on initial tumor establishment? It would be helpful for the authors to comment on whether this rapid progression might preclude the detection of a true biological difference that might manifest in a less aggressive system. Is such a rapid model still a biologically relevant model of ovarian PM?
Perhaps performing this experiment again with fewer injected cells could slow the tumor kinetics, providing a wider experimental window to observe any potential differences between the groups. At a minimum, this potential limitation warrants discussion.

The KPCA model was performed using the lowest tumor cell dose reported in the original study establishing this system, and experimental parameters were matched to published conditions. As expected for this aggressive model, rapid tumor expansion combined with the dynamic range limitations of bioluminescent imaging led to early signal saturation, limiting the ability to resolve subtle early temporal differences.

In this experiment, our objective was to determine whether mature adipocyte depletion alters omental tumor localization under rapid growth conditions, rather than to quantify fine-scale kinetic differences in early establishment. At the analyzed time point, bioluminescent signal remained within a measurable range that clearly resolved robust omental tumor localization in both genotypes, indicating that mature adipocyte loss does not prevent omental seeding in this system.

Importantly, slower-growing models (BPPNM and ID8p53–/–Brca2–/–) provided a broader temporal window and similarly showed no requirement for mature adipocytes in tumor establishment or expansion. We have clarified this limitation and its interpretation in the Results section.

(27) Similarly, in both DARL and PARC cohorts, nearly 30% of the mesenteric fat region was occupied by tumors, while the sheet was virtually spared (Fig. 2h).
Line 504-505: Similarly, in both DARL and PARC cohorts, nearly 30% of the mesenteric fat region was occupied by tumors, while the sheet was virtually spared (Fig. 2h).
The manuscript's description of the mesothelial 'sheet' as a passive entity may understate its critical role in metastasis. Additionally, it is assumed that to gain access to the deeper adipose-rich regions, the mesothelial barrier needs to be breached. How do the authors explain this distinction? This process involves dynamic processes like mesothelial cell retraction and the mesothelium's innate wound-healing response.
The discussion would be strengthened by acknowledging this **active gatekeeper function**. For instance, the finding that the 'sheet' is spared could be contextualized by framing it as a transient site of invasion through which tumor cells pass to reach the more hospitable niche underneath. This would provide a more complete and accurate mechanistic picture.

We agree that mesothelial cells function as an active gatekeeper in peritoneal metastasis rather than a passive barrier. We have revised the Discussion to explicitly acknowledge their role as the first cellular interface encountered by disseminated tumor cells, including tumor-induced retraction, wound-healing responses, and transcriptional reprogramming described in prior studies.

In our study, mesothelial populations were rigorously annotated, sub-classified using an omentum-specific reference, and quantified in tumor-naïve tissue. We observed no significant differences in mesothelial cell abundance between PARC and DARL mice (Fig. 5), indicating that depletion of mature adipocytes does not alter baseline mesothelial composition.

The relative sparing of the peritoneal membrane in our tumor burden analyses therefore should not be interpreted as evidence of passivity. Rather, it is consistent with a model in which tumor cells transiently traverse the mesothelial layer to access the underlying adipose-rich stromal compartment that more robustly supports sustained expansion.

We have clarified this stage-dependent framework in the Results and Discussion, distinguishing baseline mesothelial composition from tumor-induced mesothelial reprogramming described in prior work. Together, these revisions provide a more complete mechanistic context while remaining aligned with the scope of our data.

(28) Natural killer cell and lymphocyte deficiency do not impact omentum-centric OC growth in the peritoneal

cavity Line 549 : Manual flow cytometry gating (Extended Data Fig. 4a) was used to identify CD45+ immune populations overlaid on a dimensionality reduction plot, identifying 12 clusters (Fig. 3b).
Why was there no simple analysis of stromal cells performed? This could easily answer the question of whether the DARL model influences, for example, mesothelial cell behavior.

We agree that stromal cell composition is an important consideration. Although our primary dimensionality reduction analysis focused on $CD45^+$ immune populations, we also analyzed $CD45^−$ stromal cells by manual gating (Extended Data Fig. 4a), including $CD31^−PDGFR\alpha^+$ fibroblasts and $podoplanin^+$ mesothelial cells. We did not detect significant differences in mesothelial cell abundance between PARC and DARL mice. Given the limited marker resolution of flow cytometry for stromal subsets, we further interrogated stromal heterogeneity using single-cell RNA sequencing (Fig. 5), which provides higher-resolution profiling. These analyses likewise showed no genotype-dependent differences in mesothelial representation, indicating that adipocyte depletion in the DARL model does not measurably alter baseline stromal or mesothelial cell composition.

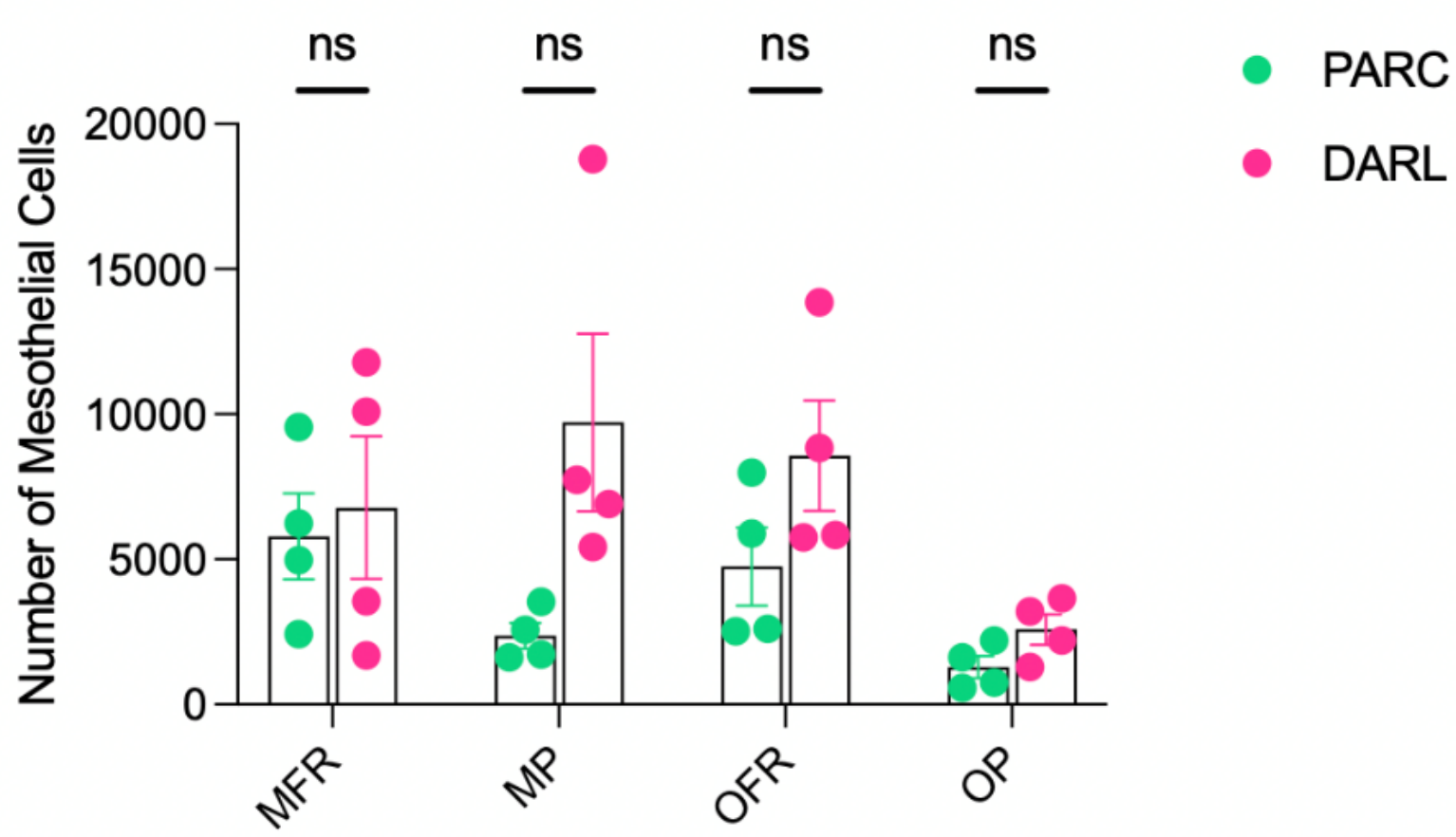

(29) Line 563: we observed no difference in the overall rate of tumor expansion between WT and triple KO mice. This is a result that immediately raises questions, which should be addressed in more detail. Additionally, figure 2e does not specify these experiments were carried out in triple knockout mice, rather only indicating PARC and DARL. Should this be fig. 3e? Additionally, following depletion, why were there no additional analyses performed, including survival experiments or measurement of tumor weights? An effect on BLI is insufficient to rule out the role of the adaptive immune system. Was immunohistochemistry/flow cytometry of tumors to further delineate these effects performed? As omentectomy itself influences the progression of OC vastly, and the omentum itself is described as an immunologically extremely active organ, these findings seem to be discordant to that, and deserve further analysis. Furthermore, this finding would be strengthened by the addition of an biological replicate of this experiment using a different cell line, as both of these experiments were carried out using variants of the ID8 cell line, such as the other syngeneic models used by the authors in previous experiments.

We agree that immune contributions to peritoneal ovarian cancer progression warrant careful consideration. Immune involvement was addressed in two independent experimental settings. First, tumor growth was evaluated in triple knockout mice using ID8p53−/−Brca2−/− cells (Fig. 3e; figure labeling has been clarified). Second, in PARC and DARL cohorts, adaptive immune cells were depleted using $CD4^+$, $CD8^+$, and NK cell–targeting antibodies in a slower-growing ID8-based model (Fig. 3g). Across both genetic immune deficiency and antibody-mediated depletion, we observed no difference in tumor seeding or overall expansion, indicating that adaptive immune cells are not required for tumor establishment in these baseline conditions.

These findings are consistent with prior syngeneic ovarian cancer models in which immune-dependent differences are most evident under immune checkpoint blockade or other immunomodulatory interventions

rather than during unperturbed tumor growth. Our use of multiple tumor models with distinct genetic drivers and growth kinetics further supports that this observation is not restricted to a single ID8 variant.

All experiments were conducted to a predefined humane endpoint (ascites development) under IACUC-approved protocols. Extended survival analyses were therefore not performed. Tumor weights were not used as a primary endpoint for disseminated peritoneal disease, as nodules frequently coat organs and invade tissue planes, rendering weight measurements operator-dependent and insensitive to spatial distribution (see response to Comment 15). However, the whole omentum and mesentery were weighed (Fig. 2k), and no genotype-dependent differences were observed between tumor-bearing PARC and DARL mice.

While additional immune profiling could further characterize tumor-infiltrating populations, the absence of functional differences in tumor establishment or expansion across immune-competent, immune-depleted, and immune-deficient settings indicates that mature adipocytes are not required for omental tumor growth under the conditions tested. We have revised the Results and figure legends to clarify these points and delineate the scope of the conclusions.

(30) Metabolic syndrome does not exacerbate OC growth in the peritoneum
No comments.

(31) PARC and DARL mice have similar cell composition and high FABP4 endothelial expression in the omentum compared to the mesentery
Line 622: DARL mice were clustered based on differentially expressed genes in each cluster using canonical markers for specific populations (Fig. 5a).
What canonical markers were used? Which references? Please provide these in the form of f.e. a bubble plot.

We have completely reorganized Figure 5 to present a single, consistent clustering scheme based on established marker genes, as detailed in the revised Methods. The dot plot shown previously in Fig. 5g has been updated to include all genes used for the initial cell type classification and repositioned earlier in the figure (Fig. 5b) to clearly indicate the markers underlying the clustering.

(32) Line 626: Was the use of single-nucleus strategies not considered, as these technologies are able to capture adipocyte heterogeneity?

Single-nucleus approaches are indeed valuable for examining adipocyte heterogeneity. However, our study was not designed to characterize adipocytes or their genetic diversity. Instead, our goal was to use scRNA-seq to capture a comprehensive transcriptomic profile of all cell types in these mice, including cytoplasmic transcripts, to assess how the absence of mature adipocytes in DARL mice affects other cellular compartments within the omentum and mesentery. Because DARL mice lack mature adipocytes, single-nucleus sequencing of adipocytes would not directly address our central research question and was therefore not pursued.

(33) Line 627 – line 642, and figure 5c and 5d (Capybara analysis): The current analysis of the CD45-negative stromal populations is difficult to interpret. The use of the Capybara tool with three different reference datasets results in three distinct and seemingly inconsistent cell identity annotations (Figure 5d). This raises a critical concern: the identity of these crucial stromal populations remains ambiguous. While the likely intent was to demonstrate that cell composition is stable regardless of the reference used, this approach undermines confidence in what these cell clusters truly represent.
(a) - We strongly suggest the authors perform a more robust, integrated annotation of the stromal clusters. (This could involve a careful manual annotation using established canonical markers, informed by the results from all three Capybara comparisons.) The authors should attempt to arrive at a more confident and robust set of cell identities.
(b) - The authors should clarify the rationale for presenting three separate classifications. If the primary goal is to show compositional stability between PARC and DARL mice, this should be explicitly stated as the objective. However, this does not substitute for a clear and consistent annotation of the cell types being

discussed.
(c) In addition, there is a discrepancy between the text and data in fig 5:
- The text (line 634) states, "...there were no meaningful proportional shifts in any identified stromal cell population between the PARC and DARL tissues".
- The data in Figure 5c, however, clearly shows a statistically significant difference ($p<0.05$) in the proportion of the "CD45- Stromal 1" cluster when comparing PARC and DARL mice.
- The data in Figure 5d shows that there are statistically significant shifts between cell type distributions across conditions. To consider these shifts "not meaningful" seems subjective.
The authors must address this discrepancy. Please either amend the statement in the text to reflect the data or provide a clear biological justification for why this statistically significant shift is not considered "meaningful" in the context of the study.

We agree that the original presentation of multiple Capybara-based annotations could create ambiguity regarding stromal cell identity. We have revised both the analysis and figure presentation to provide a clearer and consolidated annotation framework.

(a) To robustly define $CD45^{-}$ stromal populations, we implemented a two-step strategy combining de novo clustering with canonical marker-based annotation to establish broad stromal lineages (fibroblast, mesothelial, endothelial), followed by reference-guided refinement using Capybara. Each reference dataset was selected for a specific biological purpose: Brulois et al. to resolve endothelial specialization, Jackson-Jones et al. to define omentum-specific mesothelial states, and Rivera-Gonzalez et al. to distinguish fibroblast–adipocyte lineage relationships, including pre-adipocyte states relevant to the DARL model. The final identities shown in Figure 5 now reflect a single integrated annotation informed by both marker expression and reference mapping. The Methods have been updated to clarify this workflow.

(b) We agree that the rationale for presenting multiple reference mappings was not sufficiently clear. Our intent was to increase biological resolution within specific stromal compartments rather than to present alternative classifications. To avoid confusion, the main figures now display a consolidated annotation, and reference-specific mappings are described in the Methods and Supplementary Information.

(c) We acknowledge that small but statistically significant proportional differences were observed in select stromal subsets. However, these differences are modest in magnitude and do not indicate stromal reprogramming or large-scale compositional shifts between PARC and DARL tissues. We have revised the text to remove subjective language and now describe these differences quantitatively. Figure presentation has been adjusted to ensure consistency between the text and the proportional data shown in Fig. 5e.

(34) The current analysis focuses on whether the number of cells in each cluster is similar across conditions. However, a stable cell composition does not necessarily mean the cells are functionally equivalent. It is crucial to investigate whether the cells in the DARL mice have an altered transcriptional state compared to those in the PARC mice. To significantly strengthen the findings, we recommend the authors perform differential gene expression (DGE) analysis within the key stromal clusters (e.g., "CD45- Stromal 1-5") by comparing the PARC and DARL conditions. This would reveal potential changes in cellular function or signaling pathways that are independent of cell abundance and would provide a much deeper insight into the impact of lacking mature adipocytes on the tumor microenvironment.

We agree that similar cluster abundance does not imply functional equivalence and that transcriptional state analysis within stromal populations is essential. In response, we performed differential gene expression analyses within the $CD45^{-}$ stromal compartment, comparing PARC and DARL mice independently of cell abundance.

This analysis resolved four transcriptionally distinct stromal programs, $Retnla^{+}$ fibroblasts, matrix-producing fibroblasts, $PDGFRB^{+}$ perivascular fibroblasts, and mesothelial cells, and differential gene

expression was assessed within these defined populations. To improve clarity in the main figures, we consolidated these programs into two higher-level categories (fibroblasts and mesothelial cells) that align with the biological scope of the study. Differential gene expression results are now presented comparing fibroblast and mesothelial compartments between genotypes, thereby directly evaluating transcriptional differences not captured by compositional analysis alone.

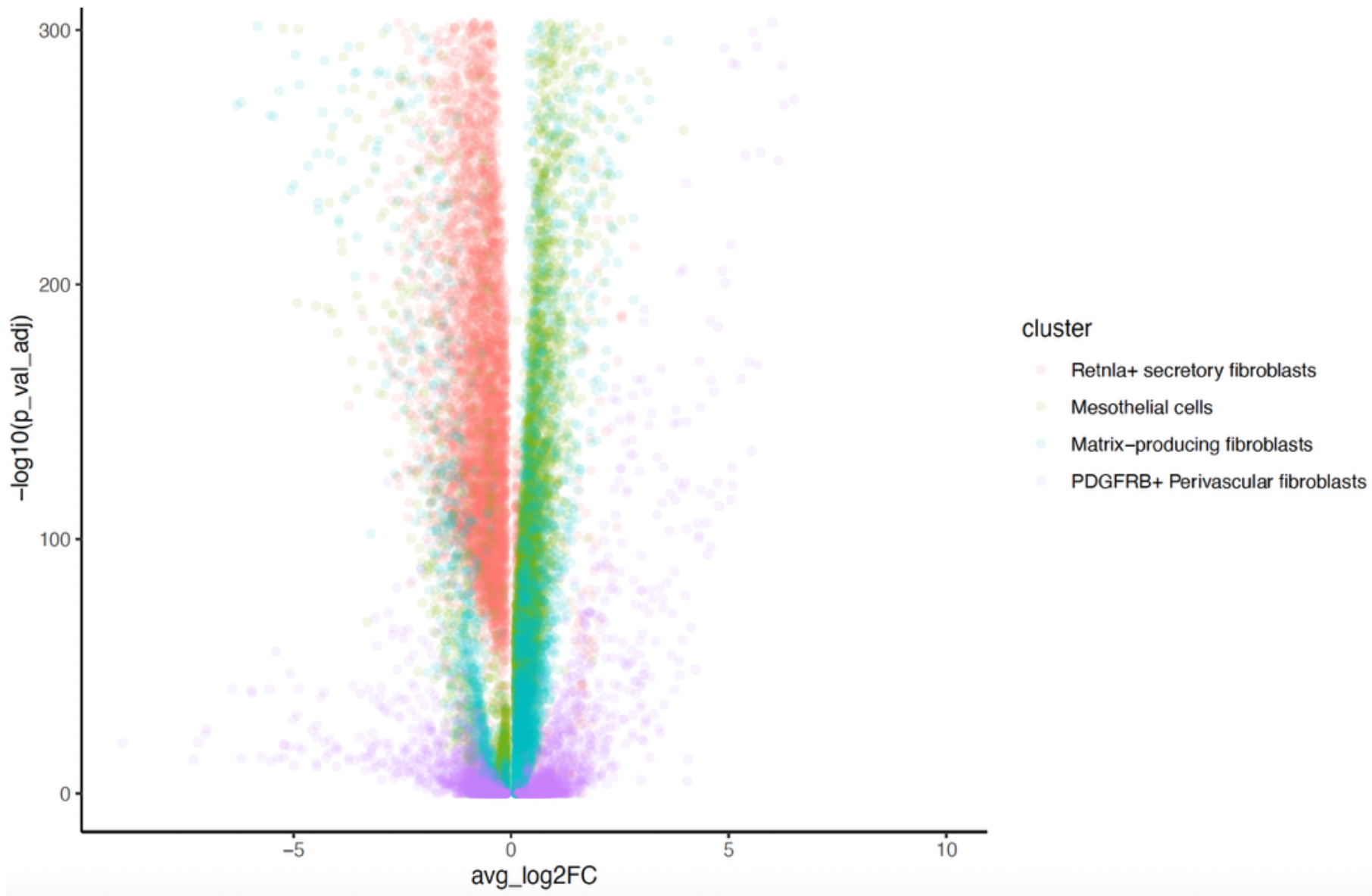

(35) Line 653 :Extended data fig 6j: this is not provided in the manuscript. Please revise.

We apologize for this typographic error. The correct references should have been Figures 6h and 6i. Now, Extended data Figure 6 has been revised, and the appropriate references have been updated in the manuscript.

(36) Endothelial FABP4 promotes omental tumor burden and defines a conserved FABP4$^+$CD36$^+$ vascular population
Previous claims were shown with 3 distinct cell lines, if there is data about the additional cell lines, it might be good to include them to show that the effect is not cell line specific.

We agree that assessing generalizability across tumor models is important. In this experiment, however, we are testing a host endothelial mechanism, specifically whether endothelial FABP4 supports omental tumor growth, rather than a tumor cell intrinsic pathway. Because the genetic manipulation is restricted to endothelial cells, changing the ovarian cancer cell line would not test a different biological mechanism but would reevaluate the same host-dependent axis in another tumor context.

Across the manuscript, we already demonstrate consistent omental tropism and adipocyte independence using multiple ovarian cancer models with distinct genetic drivers and growth kinetics. The endothelial FABP4 experiment was performed in the ID8p53$^{-/-}$Brca2$^{-/-}$ model, which captures key features of high grade serous ovarian cancer and provides a well characterized syngeneic system for mechanistic study.

To address broader relevance, we extended our analysis to human omental tissue and identified a conserved FABP4$^+$CD36$^+$ endothelial population in both benign and tumor associated samples. The conservation of this endothelial program across species strengthens the generalizability of the central conclusion. In this context, we do not believe that inclusion of additional murine tumor cell lines would materially change the interpretation of endothelial FABP4 function.

(37) Line 694-line 699: Thus, endothelial cells of the omentum in both mice and humans express elevated mRNA encoding FABP4 and CD36. FABP4 within these cells promotes tumor growth in the omentum. When these data are combined with the evidence that we provided here that omental adipocytes do not affect the pace of OC growth in the omentum or peritoneal cavity, it seems reasonable to conclude that earlier implications that

FABP4 promoted ovarian tumor growth in omentum may relate to its key role in the vasculature, rather than the adipocyte.
While including human scRNA-seq data is a commendable effort to establish relevance, the manuscript overstates its implications. The suggestion that endothelial FABP4 functionally promotes tumor growth in humans is a significant extrapolation, as the current data are purely descriptive and derived from benign, non-cancerous tissue. This evidence shows the conservation of a cell type but does not prove its function in human disease. (It might be of interest to go through the publicly available scRNA-seq datasets to see if any datasets are available of human omental tissue of OC patients.)
To improve scientific rigor, the authors should temper their conclusion. **It would be more accurate to state that they have identified a conserved endothelial population in humans that, based on their strong murine data, represents a compelling candidate for future investigation into its role in the human tumor microenvironment.**
We suggest the authors attempt to further strengthen clinical relevance of their findings in the following ways:
- Tissue analysis: Use IHC or spatial transcriptomics on human omental metastases to demonstrate the co-localization (and enrichment) of FABP4+ endothelial cells with tumor nodules.
- Functional Assays: Perform in vitro co-culture experiments using human (omental) endothelial cells and ovarian cancer cells to directly test for FABP4-dependent effects on lipid transfer, proliferation, or migration.

We agree that the human single-cell data are descriptive and do not demonstrate a functional role for endothelial FABP4 in human ovarian cancer. We have revised the manuscript to temper this conclusion and to clearly distinguish functional evidence derived from murine genetic models from descriptive observations in human tissue.

In the revised text, we now state that our data identify a conserved FABP4- and CD36-expressing endothelial population in the human omentum that parallels the population functionally implicated in murine studies and represents a candidate for future investigation in the human tumor microenvironment. To further strengthen clinical relevance, we analyzed publicly available single-cell datasets from tumor-bearing human omentum, including recent data published in Nature Communications, which confirm the presence of this endothelial population within metastatic tissue.

Regarding additional tissue level analyses, we agree that spatial characterization can add context. However, colocalization alone does not establish functional dependence. Indeed, previous assumptions regarding adipocyte-driven tumor growth were partly based on histologic proximity, an approach that our genetic data suggest can be misleading. For this reason, we have limited our conclusions to conservation of the endothelial population and avoided inferring functional activity in human disease without direct experimental evidence.

Discussion
(38) The discussion is well-written and crucially suggests two potential explanations for the link between endothelial FABP4 and OC progression. To further strengthen the manuscript, the authors could expand on the potential limitations of the Adiponectin-Cre model used to ablate adipocytes.
A key point for clarification is the developmental timing of gene expression during adipogenesis. *The Adiponectin promoter becomes active relatively late, primarily in mature, lipid-laden adipocytes. In contrast, FABP4 expression is known to begin earlier in the differentiation process.*
This timing difference creates a potential developmental window where adipocyte progenitors or immature adipocytes could be FABP4 -positive but Adiponectin-Cre -negative. Consequently, these cells would survive the DTA-mediated ablation in the DARL mice. The authors should discuss whether this surviving population of early, FABP4-expressing cells within the adipocyte lineage could still contribute to tumor growth. Acknowledging this as a potential caveat would provide a more nuanced interpretation of the results.

We agree that the developmental timing of adiponectin versus FABP4 expression warrants clarification and have expanded the Discussion accordingly. Adiponectin expression is restricted to terminally differentiated mature adipocytes, whereas FABP4 can be induced earlier during adipogenesis. The Adiponectin Cre Rosa

DTA model therefore selectively ablates mature adipocytes while preserving earlier adipocyte lineage cells, including pre adipocytes.

While FABP4 expression can precede adiponectin during differentiation, early adipocyte lineage cells are present in both PARC and DARL mice and are not selectively enriched in DARL animals. Our single cell analysis further shows that FABP4 positive stromal cells outside the endothelial compartment are rare and low in abundance. Together with the preserved tumor growth observed in the absence of mature adipocytes, these findings argue against a dominant functional contribution from pre adipocytes in this model.

We have revised the Discussion to acknowledge this potential caveat explicitly and to clarify that, although early adipocyte lineage cells persist, our data do not support a tumor promoting role for these populations under the conditions tested.

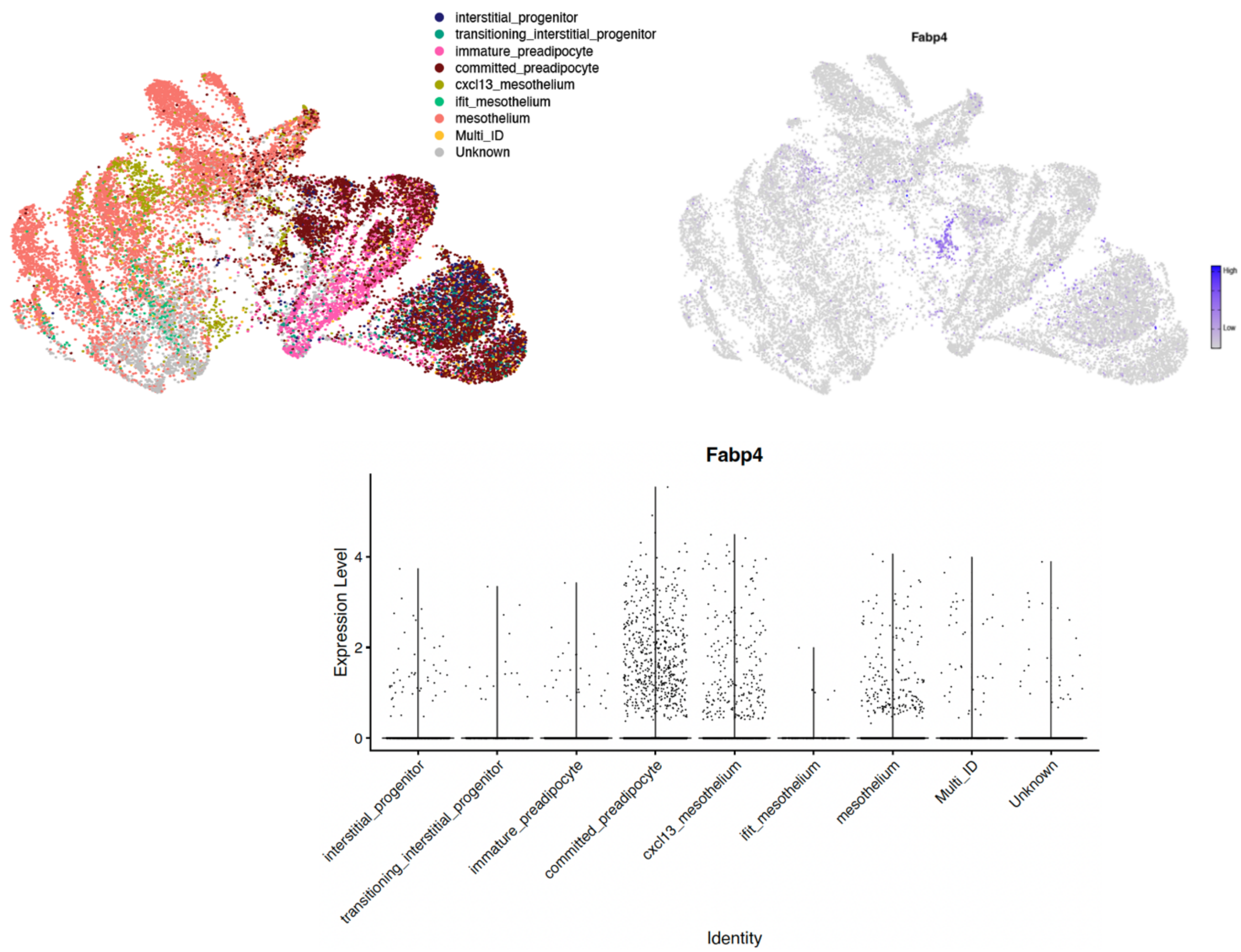

(39) Line 746: Within the omentum, endothelial cells were the primary source of FABP4 expression, both in the presence and absence of mature adipocytes.

See the previous comment (introduction). No direct evidence for this claim is shown. This claim seems to be primarily based on the scRNA-seq data of the PARC and DARL mice. However, this scRNA-seq data does not contain adipocytes. (as would be expected from scRNA-seq data).

This claim could be revised to more accurately state that endothelial cells were the primary producers of FABP4 among the successfully profiled non-adipocyte cell populations in both adipocyte-replete (PARC) and adipocyte-deficient (DARL) mice.

We appreciate the reviewer's careful reading and agree that our original phrasing could lead to misinterpretation, since scRNA-seq data typically excludes mature adipocytes due to their fragility and large size. Our intent was to highlight that, within the cell types successfully captured by scRNA-seq, endothelial cells consistently emerged as the predominant source of *Fabp4* transcripts in both PARC (adipocyte-replete) and DARL (adipocyte-deficient) omenta. To clarify this point, we have revised the sentence.

Figures:
Figure 1
(40) Some of the images are very illustrative of the points being made. The location of the figures is not entirely logical (with panel g and h being at the top of the figure). Re-organizing the figure might make it easier for the reader to follow along. However, we understand it's not always possible given the space each panel takes.
Panel a and b: We recommend to add the individual data points to the bar chart. This increases transparency by revealing the data distribution.

Thank you for the thoughtful suggestions. We kept the existing figure layout but revised the Results text and panel labeling to improve flow. Specifically, the panels previously labeled g–h are now Figure 1c–d**,** and they are discussed earlier in the text to align the narrative with their visual placement. We believe this reduces any discontinuity between the manuscript and the figure.

In addition, we overlaid individual data points in Figure 1a–b and Extended Data Figure 1a–b, making the underlying data distributions visible.

(41) Panel e: For the left panel, maybe adding CD19 (in red) and ID8 (in green) to the figure for additional clarity.

We have added CD19 (in red) and ID8 (in green) to the stereoscope image as well to match the 2-photon image we had already annotated. We also separated these images with their own panel to avoid confusion.

Figure 2
(42) Panel b:
- We strongly suggest showing the data distribution by adding the individual data points to the bar chart.

We have modified this figure to include data from an experiment with gonadal fat in the DARL group. We have now represented the data with individual points.

- Multiple t-test are performed. Based on the text it is unclear if p-values were adjusted for multiple comparisons.

We have updated the legend with the new statistical information. Pairwise comparisons of absolute photon flux between PARC and DARL mice were performed for each organ using two-tailed Mann–Whitney tests. Multiple comparisons were adjusted using the Holm-Šídák method (n.s. for any organ).

(43) Figure 2c,d: these images are not clear, and tissue orientation is unclear. Please provide a higher magnification image, with clear orientation (e.g. cut perpendicular to the surface).

We have clarified that the orientation for imaging is perpendicular. We have also provided updated images at different magnifications (please see comment 22).

(44) Panel f and g:
- How this particle analysis works is not very clear to me. By eye, I am not able to detect the tumors corresponding to the masks. This analyses should be explained in more detail in the figure legend or material and methods sections. Because I do not understand how the masks were obtained, and because it is not immediately obvious to what they correspond, the masks are unconvincing.

To distinguish numerous small nodules from a few large ones, we quantified tumor number and size using the FIJI Analyze Particles pipeline, which is described in the methods section. The masks represent the tumor nodules themselves, based on signal intensity in the bioluminescence images. The images were binarized, and automatic thresholds were applied to assess tumor coverage. The different colored masks correspond to tumor nodules.

(45) Panel h: Multiple t-test are performed. Based on the text it is unclear if p-values were adjusted for multiple comparisons. Also really not obvious that a normal distribution can be assumed for percentage data clustering near 0% or 100%.

We tested the data for normality, and it is not normal due to insufficient sample size. After consulting statistician Jing Wang, we conducted pairwise comparisons of percent tumor burden between PARC and DARL mice across organ regions using two-tailed Mann–Whitney tests. Multiple comparisons were accounted for using the Holm-Šídák method. The legend has been updated.

(46) Panel i: Would not assume normal distribution here. Also correct for multiple testing.

We formally tested for normal distribution, and given the sample size, the data did not meet the criteria. Thus, we did not assume normal distribution. We conducted a Kruskal-Wallis test followed by Dunn's multiple comparison tests. The legend has been updated.

(47) Panel j:
- I would suggest making it clearer on the graph which significances are the result of which groups being compared. For example, by re-organizing the legend and adding a small bar between PARC and PARC omentectomy with the appropriate significance.
- It is also not clear why only 2x a two-tailed t test has been done in this graph, why in K and l, a two-way anova has been used followed by multiple comparisons with p-value correction. The latter method seems more appropriate for panel j as well.

Following consultation with a biostatistician, we revised the statistical analysis for Panel j to better reflect the distributional properties of the data and to ensure consistency across similar experimental designs in the manuscript. Specifically, rather than relying on pairwise comparisons at individual time points, we quantified the area under the curve (AUC) of total photon flux over time for each mouse, which provides a robust, integrated measure of longitudinal signal and is less sensitive to deviations from normality observed in these data.

Group comparisons of AUC values were then performed using a Kruskal–Wallis test followed by Dunn's multiple-comparison post hoc tests, consistent with the analytical approach used for Panels k and l. This analysis revealed a highly significant overall group effect (****$P < 0.0001$).

In addition, we have clarified the figure annotations by reorganizing the legend and adding comparison bars to explicitly indicate which groups are being compared, as requested.

Figure 3:
(48) Panel c: Suggestion: It's not easy to interpret these pseudocolor heatmaps. I wonder if splitting the UMAP from figure 3b would not provide a clearer picture?
The bar charts in figure 4d are very clear. Perhaps providing a stacked bar chart is an option?

We appreciate the reviewer's suggestion and agree that pseudocolor heatmaps require orientation to the reference UMAP shown in Figure 3b. To address this, we have revised the text and figure legend to more explicitly state that the heatmaps in Figure 3c reflect the same cell populations delineated in Figure 3b, which is a common convention for visualizing single-cell data.

For readers who prefer quantitative bar representations, we have already included Figure 3d, where the same data are summarized as bar graphs for easier interpretation. Because flow cytometry data are not typically represented as stacked bar charts, and to avoid potential confusion from multiple overlapping visualization styles, we believe that the combination of UMAP-based pseudocolor heatmaps (Figure 3c) and bar graphs (Figure 3d) provides sufficient clarity while maintaining consistency with standard practices.

(49) Panel d: Multiple comparisons. Perhaps an ANOVA (or a nonparametric alternative) followed by a post hoc test for multiple comparisons would be a more appropriate test.

These data were not considered normal as per the D'Agostino-Pearson omnibus normality test, the Anderson-Darling test, the Shapiro-Wilk normality test, or the Kolmogorov-Smirnov normality test. Thus, we could not conduct an ANOVA. Instead, we did a Kruskal-Wallis test followed by Dunn's multiple comparison test. We have updated the legend accordingly.

(50) Panel e: The figure legend mentions that the statistical test was not significant. We suggest to also add this to the graph.

We have added this to the graph.

(51) Panel g: The figure legend mentions that the statistical test was not significant. We suggest also to add this to the graph.

We have added this to the graph.

Figure 4:
(52) Panel a: The figure legend mentions that the statistical test was not significant. We suggest to also add this to the graph.

We have added this to the graph.

(53) Panel c:
- Write c in bold in the figure legend.

We have bolded c in the figure legend.

- Since the different anatomical locations add up to 100%, you are dealing with proportional data. Using a t-test on proportional data might not be appropriate, because the values over the anatomical locations are not independent (if the percentage in one location is higher, the percentage in another location must be lower).
- It's also necessary to correct for multiple comparisons.

To address these concerns, we revised both the data presentation and statistical analysis. Specifically, we replaced the percentage-based data with absolute photon flux measurements from an experiment in which gonadal fat was collected from the Cre+ group. This change avoids analyzing values that must sum to 100% and allows direct comparison of tumor burden across organs, which better reflects the biological question of interest.

Because the absolute photon flux data were not normally distributed, and following consultation with biostatistician Jing Wang, we performed two-tailed Mann–Whitney tests comparing control and lipoatrophic mice for each organ. Multiple comparisons were corrected using the Holm–Šídák method, and no comparisons remained significant after correction (n.s. for all organs).

(54) Panel d: Again, not obvious that for percentage data near 100% or 0%, the data would be normally distributed. Nonparametric tests, along with multiple-testing correction, might be more appropriate.

The data are not normally distributed. Thus, we now have completed pairwise comparisons of percent tumor burden between PARC and DARL mice were performed for organ regions using two-tailed Mann–Whitney tests. Multiple comparisons were accounted for using the Holm-Šídák method. The figure legened is updated.

Figure 5:
(55) Panel a: I would suggest increasing the font size of the cell types a little bit to make the legend more readable.

We have increased the font size of the cell types to make the legend easier to read.

(56) Panel c: P-values should be adjusted for the multiple t-tests done here. It is not clear there has been an appropriate correction for multiple testing. A non-parametric Mann-Whitney U test might be a safer choice if you are unsure if the data follows a normal distribution.

We thank the reviewer for this comment. We conducted multiple Mann-Whitney U tests, given that the data were not parametric, and adjusted for multiple comparisons. The legend has been updated.

(57) Panel d: This figure is very unclear to me. If I have understood it correctly, the scRNA-seq data was automatically annotated using Capybara based on 3 different references. However, Using each reference, a completely different annotation for the CD45- stromal cells was achieved. classifiers classify your data with very different results. It's very confusing what the point of this analyses is and what conclusions I should draw. It seems to me that you require a very good, annotated reference for Capybara to work properly. I feel that the fact that these different references lead to such different results underlines that you actually have no idea of the identity of the stromal cells in your sample. We strongly suggest that the authors perform a more robust, manual annotation of the data. Is the point that independent of the reference, no strong changes were seen in cell type composition? Line 634 mentions "there were no meaningful proportional shifts in any identified stromal cell population between the PARC and DARL tissues (Fig. 5d), including pre-adipocyte and adipocyte progenitor populations, that may stimulate matrix remodeling to support OC metastasis61". However, panel c in Fig. 5 clearly shows a statistically significant shift for "CD45- Stromal 1" when comparing PARC and DARL mice.

We have updated Figure 5 significantly such that this panel no longer exists. We believe that the new Figure addresses these concerns as explained above.

(58) Panel h:
- Does the graph show (normalized) log-transformed counts, raw counts, please indicate unit of the y axis?
- The data distribution is clearly not normal here. While there seems to be a clear difference between endothelial Fabp4 expression in the mesentery compared to the omentum, a t-test is not appropriate here. A nonparametric test, e.g., the Mann-Whitney U test, would be more appropriate here.
- If a comparison is done between endothelial cells and other immune and non-immune subtypes, as claimed in the figure legend, please show this comparison on your figure and adjust your p-values.

First, we have clarified the y-axis to indicate that the values represent log-transformed gene expression counts. This information has been explicitly added to the axis label.

Second, given the non-normal distribution of the data, we replaced the parametric test with a two-sided non-parametric Wilcoxon rank-sum test (Mann–Whitney U test) with continuity correction. This test was used for all reported comparisons.

Third, although the original figure legend implied a comparison between endothelial cells and other immune and non-immune cell types, we have revised the legend to accurately reflect the analysis performed. The primary comparison shown is FABP4 expression in endothelial cells from the omentum versus the mesentery, which is the most biologically relevant contrast supporting our conclusion. To further aid interpretation, we additionally report the percentage of cells expressing FABP4 within each category.

We did not include comparisons across all cell types in the figure, as FABP4 expression in non-endothelial populations is minimal and does not meaningfully contribute to the biological interpretation. Therefore, correction for multiple comparisons was not required. Nevertheless, to address this concern conservatively, we performed a Bonferroni correction, and the result remained highly significant (adjusted p <

2.2×10^{-16}), consistent with the large number of cells in the dataset.

Figure 6:
(59) Panel a, panel b, panel c: Please mention the statistical test used per panel. Be sure to correct for multiple testing if appropriate.

We have added the statistical test used to the legends. We used non-parametric tests throughout, given that the data were not normally distributed, and we corrected for multiple comparisons where appropriate, as indicated in the legends.

(60) Panel e and g: Two endothelial cell clusters are shown in panels e and g. Is there a significant difference between them (e.g., vascular vs lymphatic)?

One of the two endothelial cell clusters circled in red below has too few cells to provide meaningful analysis or to support disparate annotation from the larger endothelial cell cluster. However, both clusters express PECAM1 and were therefore considered together as the endothelial cell population. We have incorporated a new human data set re-analyzed from *Nature Communications* into the text, which includes omenta from 15 patients and is robust for blood endothelial cells and lymphatic endothelial cells. We now include it in the paper to provide added specificity about these two endothelial subpopulations.

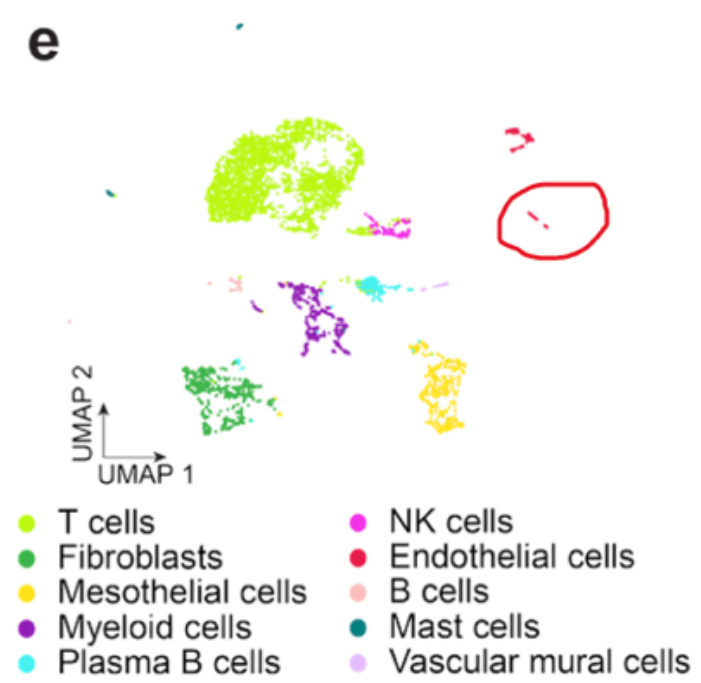

(61) Fig. 6f: the ubiquitous presence of igkc in most clusters indicates a potential contamination with ambient RNA. We recommend ambient rna correction to be used for these data.

In our data, IGKC expression is not ubiquitous but is restricted to B lineage clusters and co-localizes with other canonical B cell markers. We do not observe diffuse low-level IGKC signal across non-B cell populations, arguing against ambient RNA contamination. Therefore, ambient RNA correction was not applied.

Reviewer #2 (Remarks to the Author):

I co-reviewed this manuscript with one of the reviewers who provided the listed reports. This is part of the Nature Communications initiative to facilitate training in peer review and to provide appropriate recognition for Early Career Researchers who co-review manuscripts.

Reviewer #3 (Remarks to the Author):

In manuscript “Adipocytes are dispensable in shaping the ovarian cancer tumor microenvironment in the omentum”, Mintz et al. study the role of omental adipocytes in OC. They show that OC cells retain a propensity to seed regions typically associated with adipocytes in mice even without mature adipocytes. However, adipocyte-free omentum was found to be important for OC expansion. Endothelial cell-selective deficiency of FABP4 reduced OC growth in the peritoneum. The authors conclude that that endothelial cells within the omental vasculature are the primary sources of the fatty acid transport protein FABP4.

The study improves understanding of the dynamic crosstalk between AT and tumor.

However, there are problems with data interpretation and missing controls, which limit this work.

Major Comments:

(1) The importance of vasculature is well understood, and specifically for OC (studies by Anil Sood and others). FABP4, aka adipocyte protein 2 (AP2), is a carrier protein for fatty acids that is primarily expressed in preadipocytes, adipocytes and macrophages. Silencing FABP4 in tumor endothelium has been previously shown to suppresses OC growth and peritoneal/omental metastatic burden in mouse models as cited (89,90). This limits novelty.
CD36 in the endothelium is also well known to mediate lipid transport (cite doi: 10.1172/jci.insight.147057) , so, not much is new there either.

We appreciate the reviewer’s careful consideration of the prior literature. We fully agree that the importance of the vasculature in ovarian cancer progression is well established, and that FABP4 and CD36 mediated lipid handling have been implicated in tumor growth in previous studies. Our work does not seek to redefine these molecules or their biochemical functions.

The conceptual advance of this study lies in directly testing a long-standing assumption in the field: that mature adipocytes are required for omental metastatic growth and that previously observed FABP4 dependent effects primarily reflect adipocyte-derived lipid transfer. Prior studies demonstrating suppression of tumor growth through FABP4 perturbation did not resolve the specific stromal cell type responsible for these effects within the omental niche.

Here, we genetically eliminate terminally differentiated mature adipocytes while preserving systemic metabolic homeostasis and show that ovarian tumors retain robust omental tropism and expansion in their complete absence. This uncouples adipocyte abundance from omental metastatic competence and distinguishes adipose tissue as an anatomical niche from mature adipocytes as a required cellular component of that niche.

Within this framework, the identification of FABP4 expressing endothelial cells as contributors to early tumor expansion reframes prior findings. Rather than attributing FABP4 dependent tumor promotion to adipocytes, our data support a model in which non adipocyte stromal components, including the vasculature, can sustain tumor growth independently of mature adipocytes. Thus, the novelty is mechanistic and conceptual: we clarify the cellular source and functional necessity of adipocytes in omental metastasis, which has not been directly tested in previous models.

(2) The main issue is that Tie2-Cre was used for KO in EC. This model also KOs in hematopoietic cells. A cleaner, CDH5-Cre (inducible) or Tie2e-Cre would be better models for EC.

We agree that Tie2-Cre is not exclusively endothelial and can mediate recombination in subsets of hematopoietic cells. This limitation was acknowledged in the original manuscript, and we designed complementary experiments specifically to address this concern.

To test whether hematopoietic FABP4 contributes to the observed phenotype, we generated Lyz2-Cre $Fabp4^{fl/fl}$ mice to delete Fabp4 in myeloid lineages. In this setting, we observed no change in tumor burden,

including omental tumor growth. These data argue against a hematopoietic source of FABP4 driving the phenotype seen in Tie2-Cre Fabp4$^{fl/fl}$ mice.

In parallel, single cell RNA sequencing of murine omentum demonstrates that Fabp4 expression within the stromal compartment is strongly enriched in endothelial cells. Importantly, analysis of human omentum, including tumor-bearing samples, identifies a conserved FABP4 and CD36-expressing endothelial population. Thus, the cell type implicated genetically in mice corresponds to the dominant FABP4 expressing stromal population in both species.

While endothelial specific Cre drivers such as Cdh5-CreERT2 provide an alternative approach, the combination of myeloid specific deletion showing no phenotype, endothelial-enriched Fabp4 expression by single cell analysis, and conservation of this endothelial program in human omentum provides convergent evidence that the relevant source of FABP4 in this context is endothelial rather than hematopoietic.

We have revised the Discussion to more explicitly acknowledge the limitations of Tie2-Cre and to clarify the basis for our conclusion.

(3) Tie2-Cre KOs FABP4 in macrophages, the other main expressing cell type. This issue is alleviated by LysM-Cre control KO that does not show the phenotype. However, Yorek M et al. (eLife 2024) showed that macrophage FABP4 deficiency reduces FA-driven lipid metabolism and metastasis. Therefore, macrophage FABP4 mediates lipid transport important for cancer progression. This key study is not cited/discussed and it is not clear how this discrepancy can be reconciled.

We thank the reviewer for highlighting the study by Yorek et al. (eLife 2024), which demonstrates that macrophage FABP4 can promote lipid metabolism and metastasis in mammary tumor models. We have now cited and discussed this work in the revised Discussion.

Our findings do not contradict these observations but instead reflect tumor- and niche-specific differences. In our ovarian cancer models, myeloid-specific deletion of Fabp4 using LysM-Cre did not reduce tumor burden, including omental growth. In contrast, deletion using Tie2-Cre suppressed tumor progression, and single-cell transcriptomic analyses demonstrate that Fabp4 expression within the omental stromal compartment is enriched in endothelial cells relative to immune populations. Together, these data argue that macrophage-derived FABP4 is not required for tumor growth in this peritoneal context.

Importantly, macrophage identity and metabolic programming are highly tissue dependent. The omentum and peritoneal cavity contain four distinct resident peritoneal macrophage populations (PMID 25024137; PMID 27746181; PMID 34714329) that are transcriptionally and functionally distinct from the monocyte-derived tumor-associated macrophages commonly described in mammary and other solid tumor models. It is therefore plausible that FABP4-dependent lipid handling is functionally dominant in macrophages in certain tumor environments, while in the omental metastatic niche of ovarian cancer, endothelial FABP4 represents the primary contributor.

We have revised the Discussion to explicitly acknowledge this context dependence and to frame FABP4 function as varying across tumor types and metastatic sites.

(4) Also, adipose stromal / progenitor cells (ASCs) are known to express FABP4 and it shows in Fig. 5. FABP4 KO in them would also be important to perform for comparison. As is, the main conclusion on the pivotal source of FABP4 cannot be made.

We agree that adipose stromal and progenitor cells can express FABP4 during early adipocyte differentiation and therefore represent a potential source. We have edited our discussion to make the point that these cells are possible contributors but as they are not mature adipocytes that store and mobilize lipids, their functional role would be different than anticipated for mature adipocytes.

However, our single-cell RNA sequencing data from murine omentum and mesentery indicate that FABP4 expression within adipocyte-lineage progenitors is rare and low-level. Fewer than 5 percent of non-endothelial stromal cells exhibit detectable Fabp4 transcripts, and expression intensity in progenitor populations is modest. In contrast, Fabp4 expression is strongly enriched in endothelial cells and substantially exceeds that observed in adipocyte-lineage progenitors.

Functionally, tumor growth proceeds unchanged in the absence of mature adipocytes, indicating that adipocyte-lineage cells are not required for omental tumor expansion. Taken together, the low abundance and limited Fabp4 expression in adipose progenitors make it unlikely that they represent a major source of FABP4-mediated support in this context.

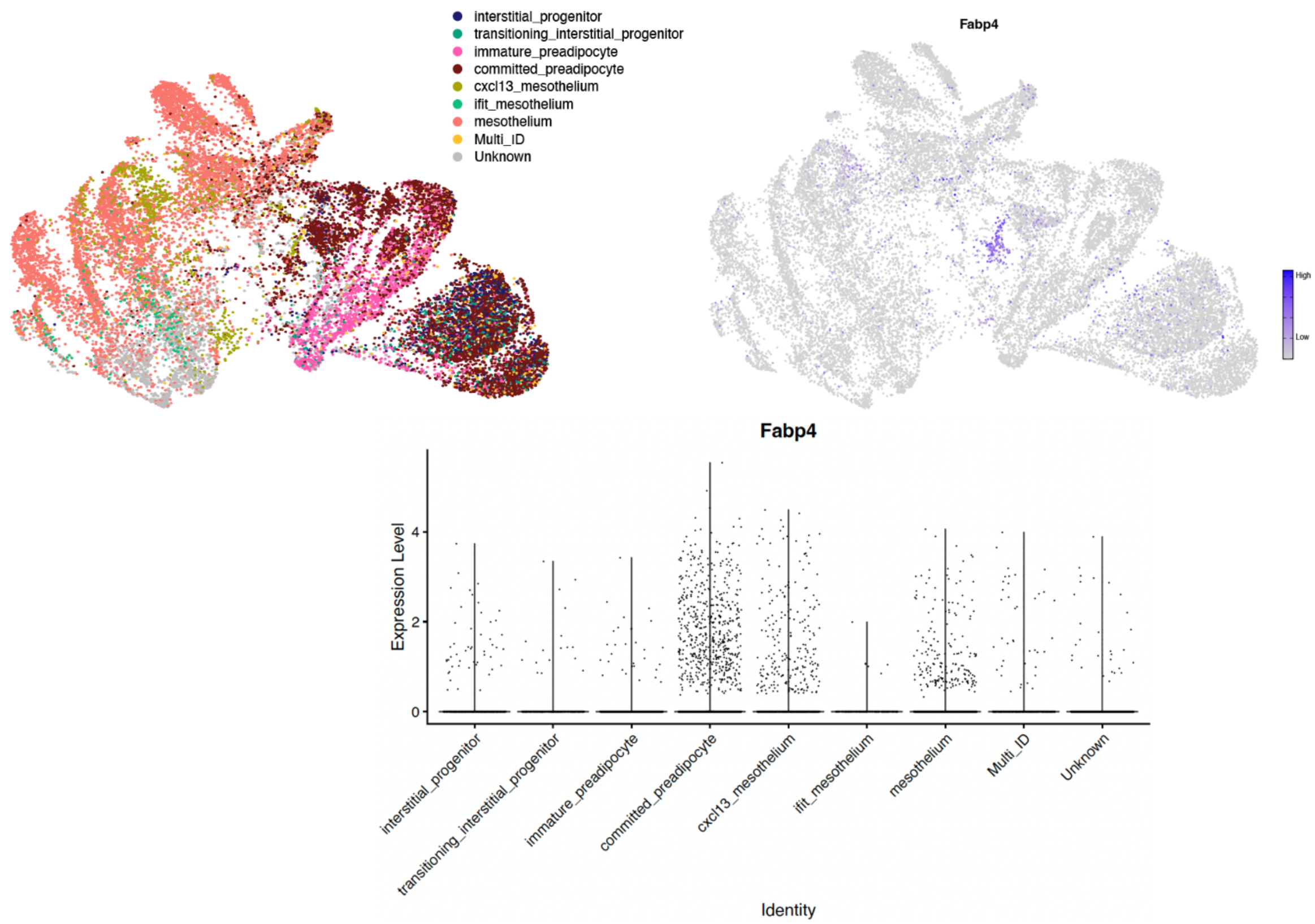

(5) Lipoatrophic mice are well known to have hypertriglyceridemia**,** hypercholesteremia, hyperglycemia and Hyperinsulinemia. Therefore, although adipocytes are missing from microenvironment, the reason tumor still grow well is probably because lipids are abundant from circulation. This is not fully addressed by AT transplants in the PARC / DARL model.

We agree that congenital adipocyte deficiency is associated with systemic metabolic alterations and that circulating lipids could, in principle, compensate for the absence of local adipocytes.

To directly address this possibility, we normalized systemic metabolic parameters in lipoatrophic mice via subcutaneous adipose tissue transplantation. As shown in Extended Data Fig. 2e–k, serum triglycerides, fasting glucose, glucose tolerance, and insulin sensitivity were restored to levels comparable to fat-bearing controls. Despite normalization of these systemic parameters, tumor growth kinetics and peritoneal distribution, including omental enrichment, remained unchanged between PARC and DARL mice (Fig. 4a–d).

Conversely, when tumor growth was assessed in lipoatrophic mice without metabolic correction, where hypertriglyceridemia and glucose intolerance persist, tumor burden and organ distribution again did not differ by genotype (Fig. 4a–d). We additionally measured total serum cholesterol (new data, Extended Data Fig. 2f) and found only modest differences that did not correlate with tumor phenotype.

Together, these experiments demonstrate that circulating lipid or metabolic alterations are not sufficient to explain preserved tumor growth in the absence of local adipocytes.

The reviewer points out the importance of lipids in the bloodstream as a possible source of nutrients for the tumor cells. We agree and argue that such a perspective makes our findings on the role of FABP4 in endothelial cells that line blood vessels all the more intriguing.

Other Comments:

(6) In Figure 5 and gene table, nine of the top ten significantly increased genes are involved in immunoglobulin production and immune cell activation (Ighv's, Cxcl5). While overall immune cell counts may be unchanged (Figure 3), immune cell function may be altered.

We agree that immune-related transcripts differ between PARC and DARL omenta and that altered immune function is possible even when overall immune cell abundance is similar. We now explicitly acknowledge this point in the Discussion. To assess functional relevance, we perturbed major lymphocyte-mediated immune axes, including antibody-mediated depletion of CD4 T cells, CD8 T cells, and NK cells, as well as genetic loss of adaptive and innate lymphocyte compartments. In all cases, tumor burden and omental enrichment were unchanged. These results indicate that the principal lymphocyte-mediated immune pathways tested are not rate limiting for tumor establishment or expansion in our models. We recognize that other immune programs, including myeloid activation states or cytokine signaling, may differ transcriptionally. However, such differences do not translate into measurable effects on tumor growth under the conditions examined here.

(7) The authors should measure changes to other fat depots such as gonadal fat. In Extended Data Figure 3, gonadal fat pads of DARL mice appear to be significantly smaller versus those of PARC Mice. While the transplanted fat pad increases in size in DARL mice, the proximity of the gonadal fat depot to the primary tumor may cause it to be preferentially mobilized by the tumor versus the transplant fat pad.

We agree that gonadal fat pads are reduced in size in lipoatrophic (DARL) mice relative to PARC controls, which is an expected feature of this model. Our primary quantitative analyses focused on the omentum and mesentery, as these represent the most consistent and clinically relevant sites of ovarian cancer metastasis within the peritoneal cavity.

With respect to adipose transplantation, transplanted fat pads were placed subcutaneously in the dorsal region, whereas tumors were established by intraperitoneal injection. The transplanted adipose tissue is therefore anatomically separated from disseminated tumor cells and does not directly interface with the peritoneal cavity. Differences in tumor growth cannot be attributed to preferential lipid mobilization based on proximity. We have clarified this anatomical separation in the Methods.

Gonadal fat is also an intraperitoneal depot and was assessed where appropriate in early characterization studies. However, ovarian cancer demonstrates a strong and reproducible tropism for the omentum, with less consistent involvement of other peritoneal fat depots. Accordingly, our mechanistic and spatial analyses were centered on the omentum, with the mesentery serving as a peritoneal comparator.

(8) Additionally, staining to visualize lipids within tumors (eg., oil red O) would be helpful to determine if lipid uptake is indeed altered in tumors of adipocyte-depleted and Fabp4 knockout mice.

We agree that assessing lipid handling within tumors is an important question. However, endpoint lipid staining of established omental tumors (e.g., Oil Red O) is difficult to interpret mechanistically in this setting. At advanced stages, tumors extensively remodel and encapsulate the omentum, generating fibrotic and heterogeneous architecture. Lipid accumulation within these lesions may reflect tumor cell uptake, stromal remodeling, necrosis, or passive deposition, and would not distinguish adipocyte-derived lipids from circulating or stromal sources. Thus, endpoint tumor lipid staining alone would not isolate adipocyte-specific contributions or directly measure active lipid flux.

To evaluate lipid availability in a more interpretable context, we performed whole-mount neutral lipid imaging at steady state using LipidTOX in tumor-naïve PARC and DARL omenta (red staining in images below). These data demonstrate detectable LipidTOX-positive lipid pools within omental milky spot regions in both genotypes. Although DARL samples show reduced adipocyte-associated architecture, consistent with

adipocyte depletion, neutral lipid signal remains present within the omental microenvironment, especially in milky spots.

Importantly, systemic lipid levels in the circulation did not correlate directly with preserved tumor growth. As shown in Figure 4, lipoatrophic mice exhibited elevated circulating triglycerides, yet tumor burden and omental enrichment remained unchanged. Conversely, endothelial-specific Fabp4 deletion did not alter circulating triglyceride levels (new data shown below), indicating that the tumor phenotype observed in Fabp4-deficient mice did not correlate with systemic metabolic changes.

Overall, lipid availability within the omentum is preserved even in the absence of mature adipocytes and that tumor growth in DARL or Fabp4-deficient mice does not correlate with circulating lipid levels. Rather, these data support a local microenvironmental mechanism independent of systemic triglyceride abundance. It is likely that circulating lipids contribute to the tumor’s nutritional demands. However, higher levels in the circulation does not lead to more tumor growth, as our data clearly show.

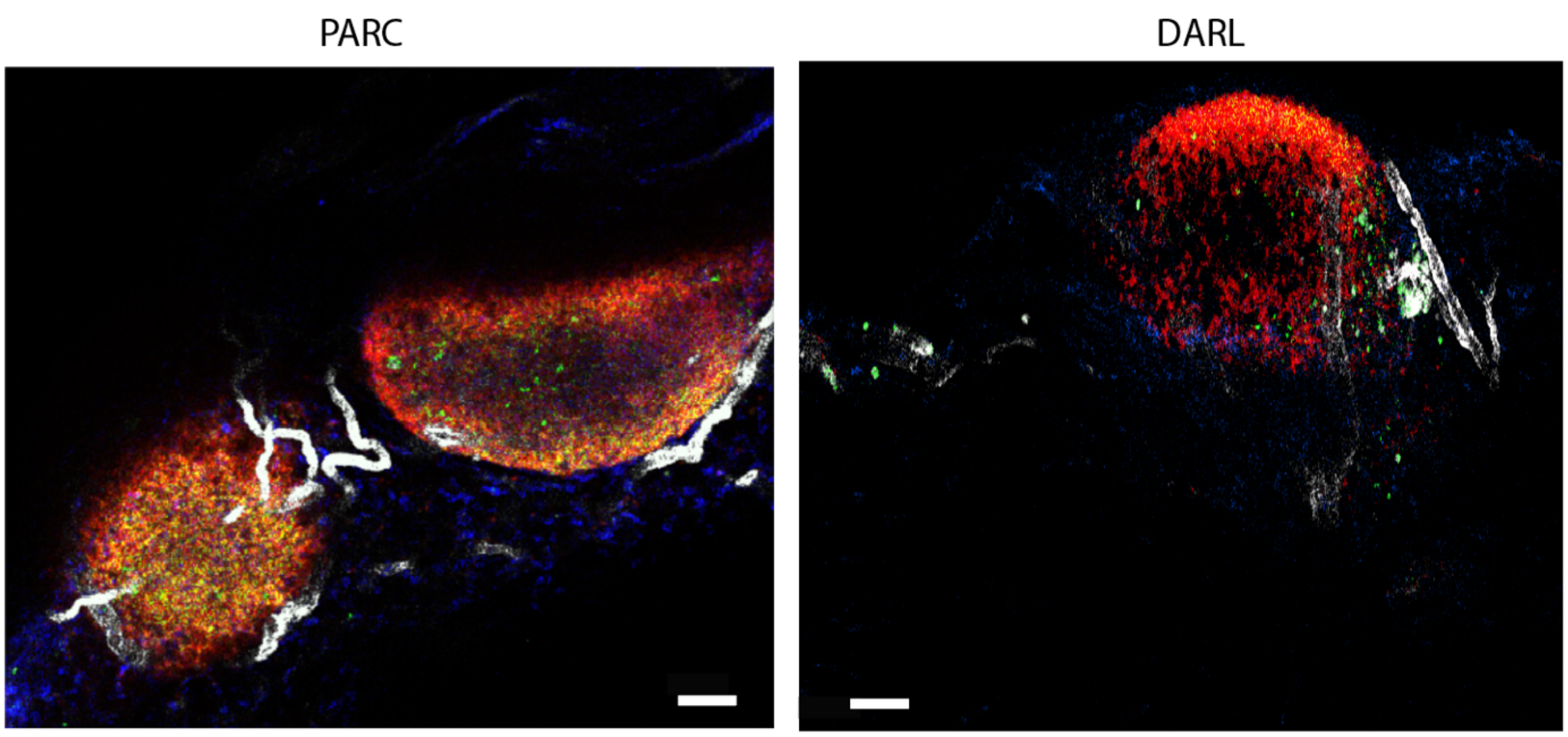

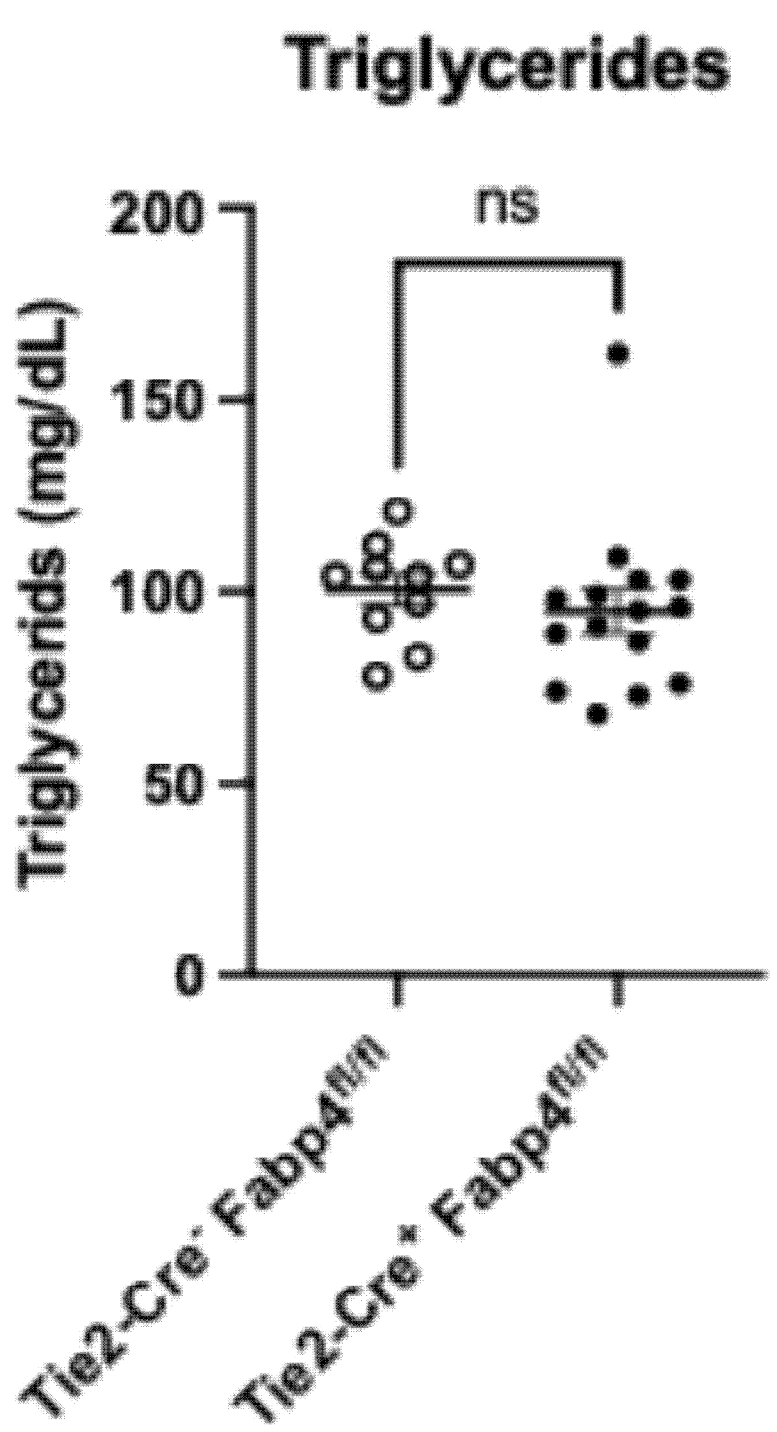

(9) Did the authors measure other Fabp such as Fabp5? In the gene expression table Fabp5 is significantly increased while Fabp4 and Cd36 are decreased. This suggests the possibility of Fabp5 compensation in the absence of Fabp4 and warrants discussion.

We thank the reviewer for raising this point. In pooled single cell RNA sequencing analysis across all captured cells, Fabp5 expression is increased in DARL relative to PARC mice, whereas Fabp4 and Cd36 are reduced, as shown in Extended Data Table 1. These results reflect aggregate transcriptional changes and do not resolve cell type specific expression within the omental niche. When examined at single cell resolution, Fabp5 is not enriched in endothelial cells and is instead detected primarily in non endothelial populations. In contrast, Fabp4 is selectively and robustly expressed in the omental endothelial compartment in both genotypes. Functionally, endothelial specific deletion of Fabp4 using Tie2 Cre significantly reduces omental tumor burden despite continued expression of other fatty acid binding proteins, including Fabp5. If Fabp5 were compensating for Fabp4 within endothelial cells, deletion of Fabp4 would not be expected to suppress tumor growth. However, tumor burden is reduced. These data therefore argue against redundant functional Fabp5 compensation in the endothelial compartment. However, only future studies can address whether Fabp5 also has a distinct role in tumor growth. Such analysis is beyond the scope of this work.

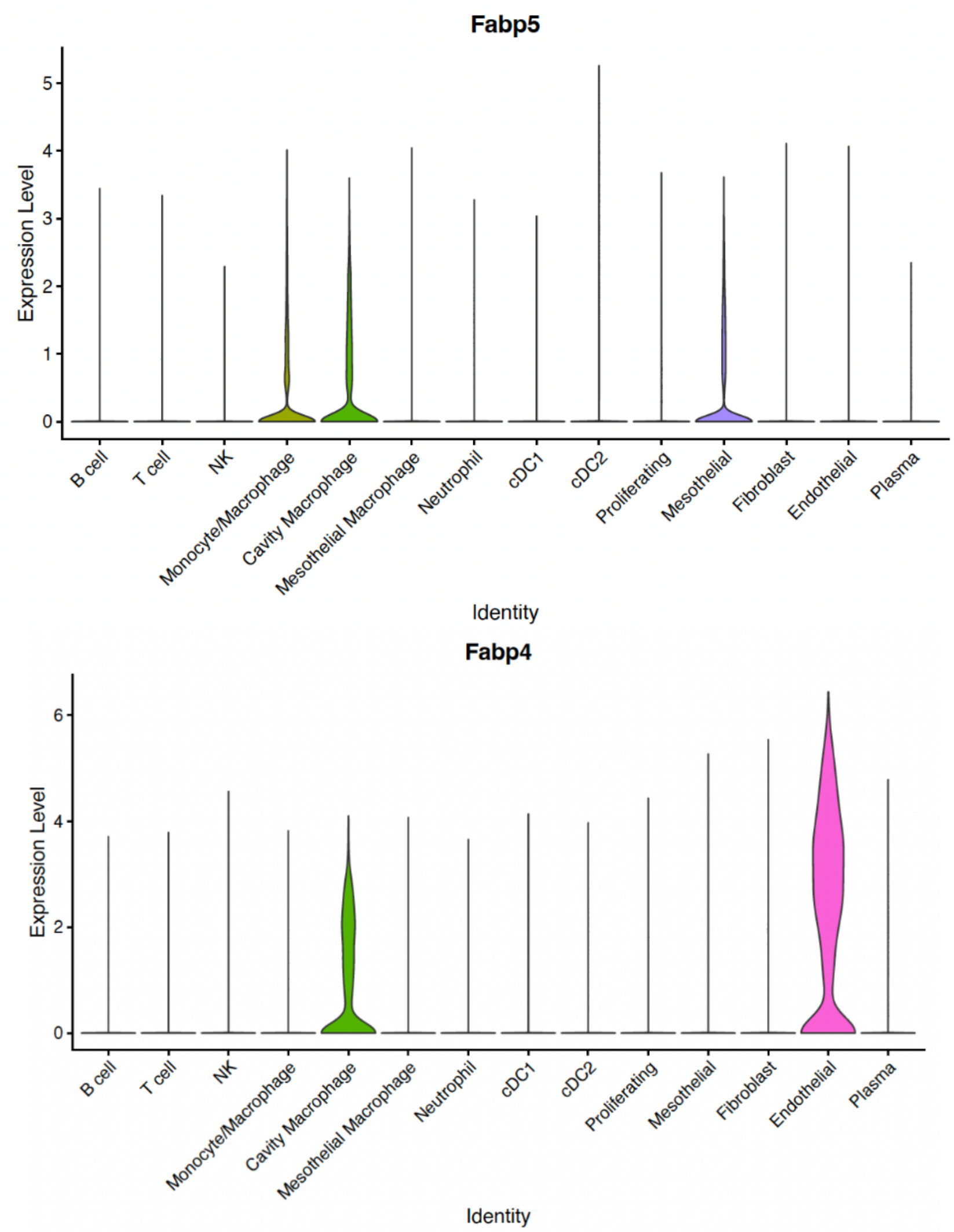

(10) Technical issue: Photon Flux bioluminescence signal can come from either more small tumors or fewer bigger tumors. It is not clear if/how this is reconciled throughout.

We agree that in vivo photon flux measurements can reflect either numerous small tumors or fewer larger tumors. To address this, we did not rely solely on integrated in vivo signal. Instead, at endpoint we

performed high-resolution ex vivo bioluminescence imaging of dissected organs, which allows visualization and spatial separation of individual tumor nodules.

Using region-of-interest segmentation and FIJI-based particle analysis, we quantified discrete nodules from ex vivo bioluminescence images, extracting independent metrics including nodule number, individual lesion area, cumulative tumor area, and percent tissue area occupied within anatomically defined regions. These data are shown in Fig. 2h–i.

Consistent with known patterns of ovarian cancer dissemination, the omentum typically contains fewer but larger nodules, whereas the mesentery contains more numerous smaller lesions. Importantly, organ-level photon flux normalized to tissue area and particle-based morphometric metrics yielded concordant between-group results. Thus, differences in nodule size distribution do not account for the observed phenotypes.

We have expanded the Methods section to clarify the ex vivo imaging resolution and quantitative analysis pipeline used throughout the study.

Manuscript needs proofreading.

(11) "while" is missing after "However,": "However, the lack of mature adipocytes did not suppress peritoneal OC expansion, whereas removing the adipocyte-free omentum did."

We have revised the sentence to emphasize the contrast more explicitly and added the word while.

(11) Other 'bugs', such as: "Capybara function construct.high.res.reference()."

We have revised the text so that the function name appears in monospace code formatting rather than italics.

(12) Intersection spaces are missing, eg, Page 15.

The missing inter-section spaces have been corrected throughout the manuscript to ensure consistent formatting.

(14) Line 230: I think the authors mean FABP4 not "FABBP4"

We have corrected "FABBP4" to "FABP4" in the revised manuscript.

Reviewer #4 (Remarks to the Author):

This is a **provocative** manuscript which challenges the concept that ovarian cancer cells metastasize to adipocytes in the peritoneal cavity (in particular to the omentum) and take up lipids by these (somehow mediated by FABP4 in adipocytes). The authors use a variety of mouse models to show that the lack mature adipocytes does not prevent metastasis to the omentum and subsequent tumor growth. Indeed the cancer cells invade the mature adipocyte-free microenvironment of white adipose tissue within the omentum. Several cell types could be involved in this process, e.g. immune cells. It is known that there is often strong immunosuppression in ovarian cancer. The authors tested the contribution of several cell types but did not find a responsible one. However, the authors detected that endothelial cells express high levels of FABP4 (which is not a new finding) and that deleting FABP4 in endothelial cells using the Tie2-Cre mouse leads to decreased tumor growth in the omentum. Tie2-Cre also targets some myeloid cells. The authors addressed this by deleting FABP4 in such a myeloid cell population and this had no significant effect on tumor growth. As such this is an important contribution to the field and raises several questions.

The authors addressed the potential confounders using lipoatrophic mice by implanting subcutaneous fat. This is an excellent strategy. Together with surgery to remove the omentum it gives a strong and convincing model.

Major points:
1) The authors used Tie2-Cre Fabp4 deficient mice. Ideally, they would have used VE-Cadherin (Cdh5)-Cre mice to address this issue. I strongly advice the authors to consider this.

We thank the reviewer for raising the important issue of Cre specificity. We agree that Tie2-Cre is not strictly endothelial-restricted and can mediate recombination in subsets of hematopoietic cells. For this reason, we directly tested the contribution of hematopoietic FABP4 using Lyz2-Cre Fabp4$^{fl/fl}$ mice. Myeloid-specific deletion of Fabp4 did not alter tumor burden or omental tumor growth, arguing against a hematopoietic source of FABP4 driving the phenotype observed in Tie2-Cre Fabp4$^{fl/fl}$ mice.

In parallel, single-cell RNA sequencing of murine and human omentum demonstrates that FABP4 expression within the omental microenvironment is strongly enriched in endothelial cells relative to immune populations, with co-expression of CD36 defining a conserved FABP4^{+}CD36^{+} vascular population. This endothelial enrichment is observed independently of genetic manipulation and supports an endothelial source of FABP4 in the omentum.

We agree that VE-cadherin (Cdh5)-Cre models can provide increased endothelial specificity. However, inducible VE-cadherin systems require tamoxifen administration and can influence vascular permeability and inflammatory tone, variables that are particularly relevant in peritoneal tumor models. Given the absence of a phenotype in myeloid-specific deletion, the endothelial-restricted expression pattern observed by single-cell transcriptomics, and the conserved endothelial FABP4 population in human omentum, we believe the central conclusion that endothelial FABP4 promotes omental tumor growth remains well supported.

We have clarified these points in the Discussion and explicitly acknowledged the limitations of Tie2-Cre in the revised manuscript.

2) The endothelial Fabp4 deficient mice need a much deeper investigation. How does this approach alter the vascular bed within WAT and the omentum? What about vessel density, vessel coverage, permeability, perfusion, and perivascular immune cell composition? Maybe the whole vessel network is already disorganized at the time when cancer cells are injected. This may compromise the whole study .

We agree that it is essential to determine whether endothelial Fabp4 deletion alters the vascular network prior to tumor implantation. To directly address this, we performed three-dimensional confocal whole-mount imaging and quantitative vascular reconstruction of tumor-naïve omentum from Tie2-Cre+ Fabp4$^{fl/fl}$ and littermate controls, as shown in Figure 6d–f. These analyses demonstrate that baseline vascular architecture, vessel density, and perivascular coverage are preserved in endothelial Fabp4-deficient mice prior to tumor

injection, arguing against pre-existing vascular disorganization that could compromise tumor seeding experiments.

In contrast, analysis of tumor-bearing omentum Figure 6d, g,h-j, reveals a distinct tumor-associated vascular remodeling phenotype in the absence of endothelial FABP4, consistent with a context-dependent role during metastatic growth rather than maintenance of steady-state vascular integrity. These data are described in the Results and incorporated into the Discussion.

3) What could be the function of FABP4 in endothelial cells? Is is needed for handling fatty acids? If yes from where are the fatty acids transported? Blood, other perivascular cells? How are lipid levels in these mice? Alternatively, it could be fatty acid independent functions of FABP4. This needs much deeper investigation.

We agree that defining the precise mechanistic role of FABP4 in endothelial cells is an important question. However, the primary objective of this study was to determine whether mature adipocytes are required for omental tumor growth and to resolve the long-standing assumption that adipocyte-derived lipid support is essential for peritoneal metastasis. Our data demonstrate that tumor growth proceeds in the complete absence of mature adipocytes, and that endothelial FABP4, rather than adipocyte FABP4, contributes to tumor burden. We understand the reviewer wants to dig deeper in the mechanism. We have now added new data in that regard.

First, we show that endothelial Fabp4 deletion does not disrupt baseline vascular architecture in tumor-naïve omentum (Fig. 6f–j), arguing against the presence of pre-existing vascular defects. However, after tumor seeding, although vascular density is not significantly altered, the branching of blood vessels and their spatial organization in tumors are significantly altered.

Circulating triglyceride and cholesterol levels were unchanged in Tie2-Cre+ Fabp4$^{fl/fl}$ mice, indicating that systemic lipid availability was not altered (please see the new data below). Together, these findings suggest that the phenotype is not driven by global lipid deficiency or baseline vascular instability.

Given FABP4's established role as an intracellular lipid chaperone, a plausible model is that endothelial FABP4 facilitates fatty acid handling or metabolic adaptation during tumor-associated vascular remodeling in the omental niche. Fatty acids could derive from circulating lipoproteins or local stromal sources; however, our data do not directly resolve the source. We briefly explored endothelial lipid uptake using oral Bodipy C16 administration and observed detectable endothelial uptake at baseline, but technical limitations precluded rigorous quantification under tumor conditions, and these exploratory data were not included in the manuscript.

We acknowledge in the revised Discussion that distinguishing lipid-dependent from potential lipid-independent functions of endothelial FABP4 will require dedicated mechanistic studies. Our conclusions are therefore limited to the functional observation that endothelial FABP4 contributes to tumor-associated vascular remodeling and omental tumor growth, while mature adipocytes themselves are not required.

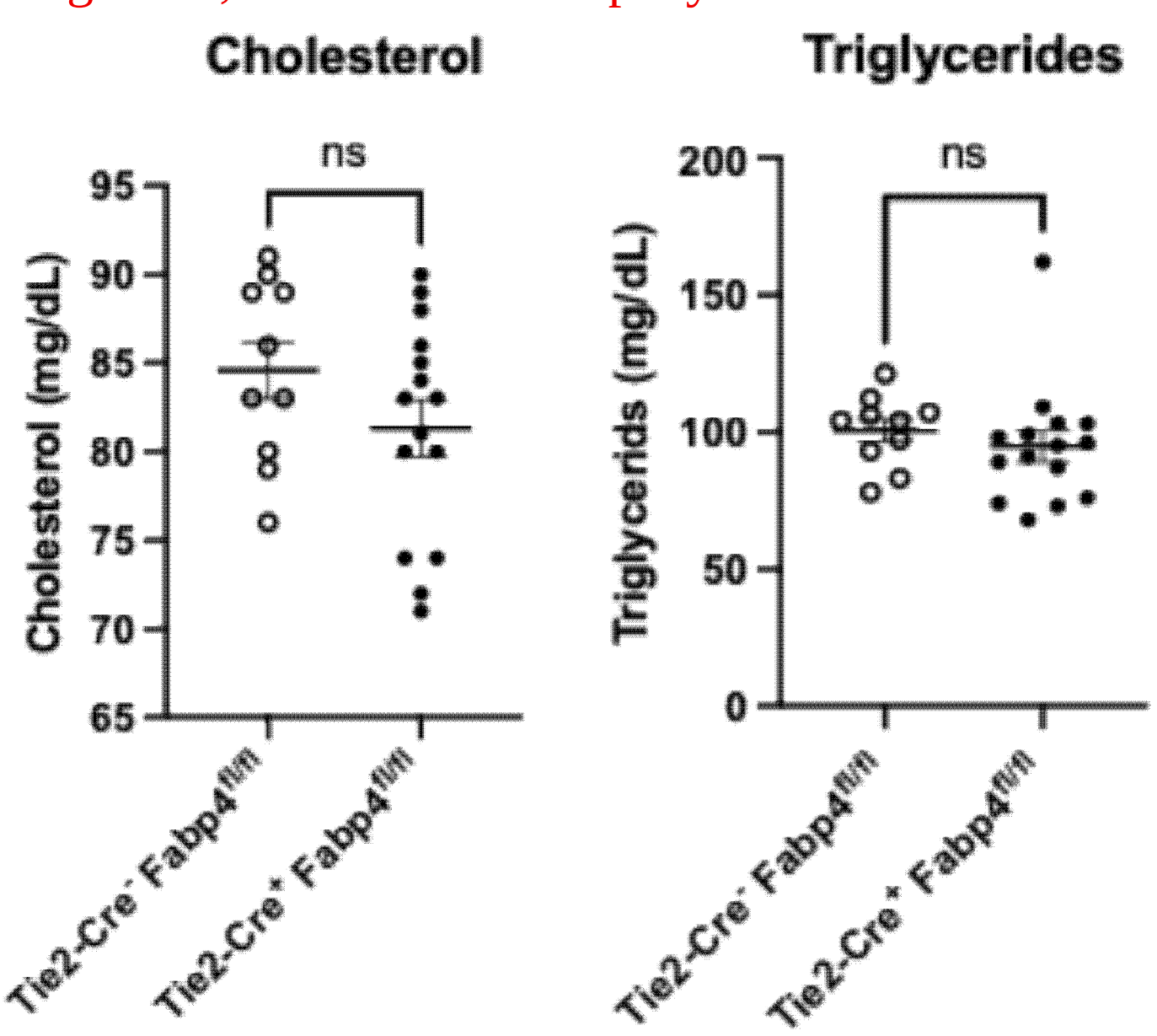

4) The effect of endothelial Fabp4 on tumor growth (Fig 6A) is not very strong. It seems that tumor growth in knockout mice just starts a few days later but then tumor growth rates are similar. Please calculate this. The authors should compare this effect with the global Fapb4 deletion in the landmark Nat Med 2011 paper (ref 9).

To address this rigorously, we calculated the area under the curve of the entire time course rather than relying on single time-point comparisons. This analysis demonstrates a significant reduction in cumulative tumor expansion in Tie2-Cre+ Fabp4$^{fl/fl}$ mice, resulting in a 1.6 decrease in tumor burden at the endpoint. We now explicitly describe this temporal dynamic in the Results.

A direct kinetic comparison to the Nat Med 2011 global Fabp4 deletion study is not feasible, as that work relied on endpoint tumor weights and surface nodule counts without longitudinal growth modeling, precluding direct comparison of growth rates. In addition, global Fabp4 deletion produces systemic metabolic effects that confound attribution to endothelial-specific mechanisms. We have revised the manuscript to clarify both the magnitude and timing of the endothelial Fabp4 effect and to distinguish it from prior global knockout studies.

To further contextualize this distinction, we have revised the Introduction to specify that the 2011 study quantified tumor burden using endpoint surface nodule counts and tumor weights, clarifying that differences in quantitative approach preclude direct kinetic comparison with our longitudinal bioluminescence-based modeling from the beginning of the paper as well.

5) Fapb4 alone and also in combination with CD36 is probably not sufficient to elevated transendothelial lipid transport. What about other crucial components such as lipases, in the mouse models? Ideally, the authors should measure transport of circulating labeled lipids to tumor cells.

We agree that FABP4 and CD36 alone are unlikely to account for the full capacity of transendothelial lipid transport and that additional lipid-processing components must be considered. To address this, we examined endothelial lipid-handling machinery in our single-cell RNA-seq dataset from PARC and DARL mice. In addition to Fabp4 and Cd36, omental and mesenteric endothelial cells express key genes required for lipoprotein processing and fatty acid handling, including Gpihbp1, which anchors lipoprotein lipase for intravascular triglyceride hydrolysis. Importantly, this endothelial lipid-processing program remains intact in DARL mice despite the complete absence of mature adipocytes, indicating that the omental vasculature retains intrinsic lipid-handling capacity independent of adipocyte-derived input.

We agree that direct quantification of lipid transport from the circulation into tumor cells would require dedicated tracer-based metabolic flux studies. We explored this experimentally by administering oral Bodipy C16 to fasted mice. However, technical limitations and rapid lipid turnover precluded reliable quantification under tumor conditions, and these exploratory data were not included in the manuscript.

Taken together, our data support the conclusion that endothelial lipid-handling machinery persists in adipocyte-deficient mice and that mature adipocytes are not required to maintain this vascular metabolic program. However, we acknowledge in the revised Discussion that resolving the precise quantitative contribution of endothelial FABP4 to lipid flux will require future dedicated metabolic tracing studies.

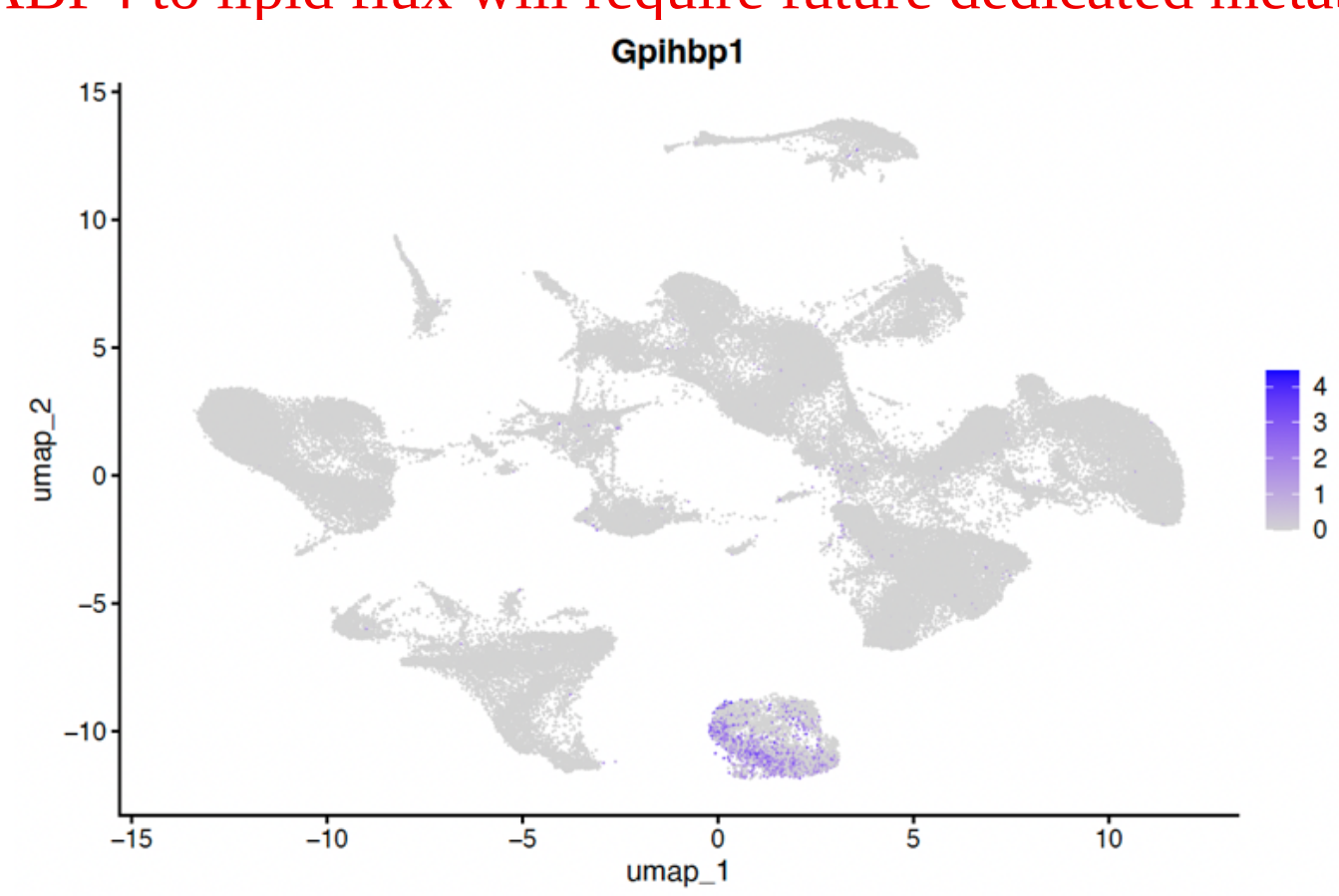

6) Does Fabp4 deletion affect the morphology of the mesothelium? Has it been shown that this Cre line does is not active in these cells.

Tie2-Cre (Tek-Cre) is widely used to target endothelial cells and subsets of hematopoietic lineages but has not been reported to recombine mesothelial cells, which are typically defined by Wt1, Pdpn, and Msln expression and are manipulated using Wt1-based Cre drivers rather than endothelial Cre lines. Consistent with this, our single-cell RNA-seq analysis shows that Fabp4 is not enriched in mesothelial populations at baseline, with only rare mesothelial cells exhibiting low-level transcripts, whereas endothelial cells show robust expression. Accordingly, endothelial-specific Fabp4 deletion is not expected to affect mesothelial cells directly. Moreover, we do not observe structural abnormalities of the mesothelial layer in endothelial Fabp4-deficient mice by histologic or whole-mount imaging, supporting the conclusion that the observed phenotype does not arise from unintended mesothelial recombination or altered mesothelial morphology.

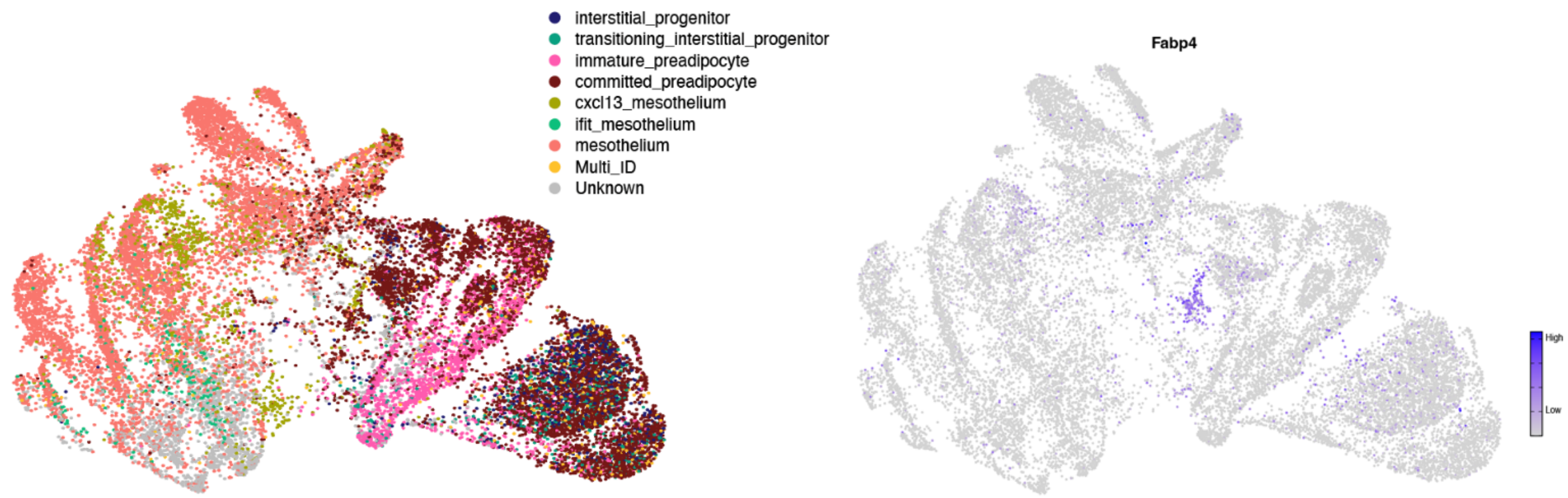

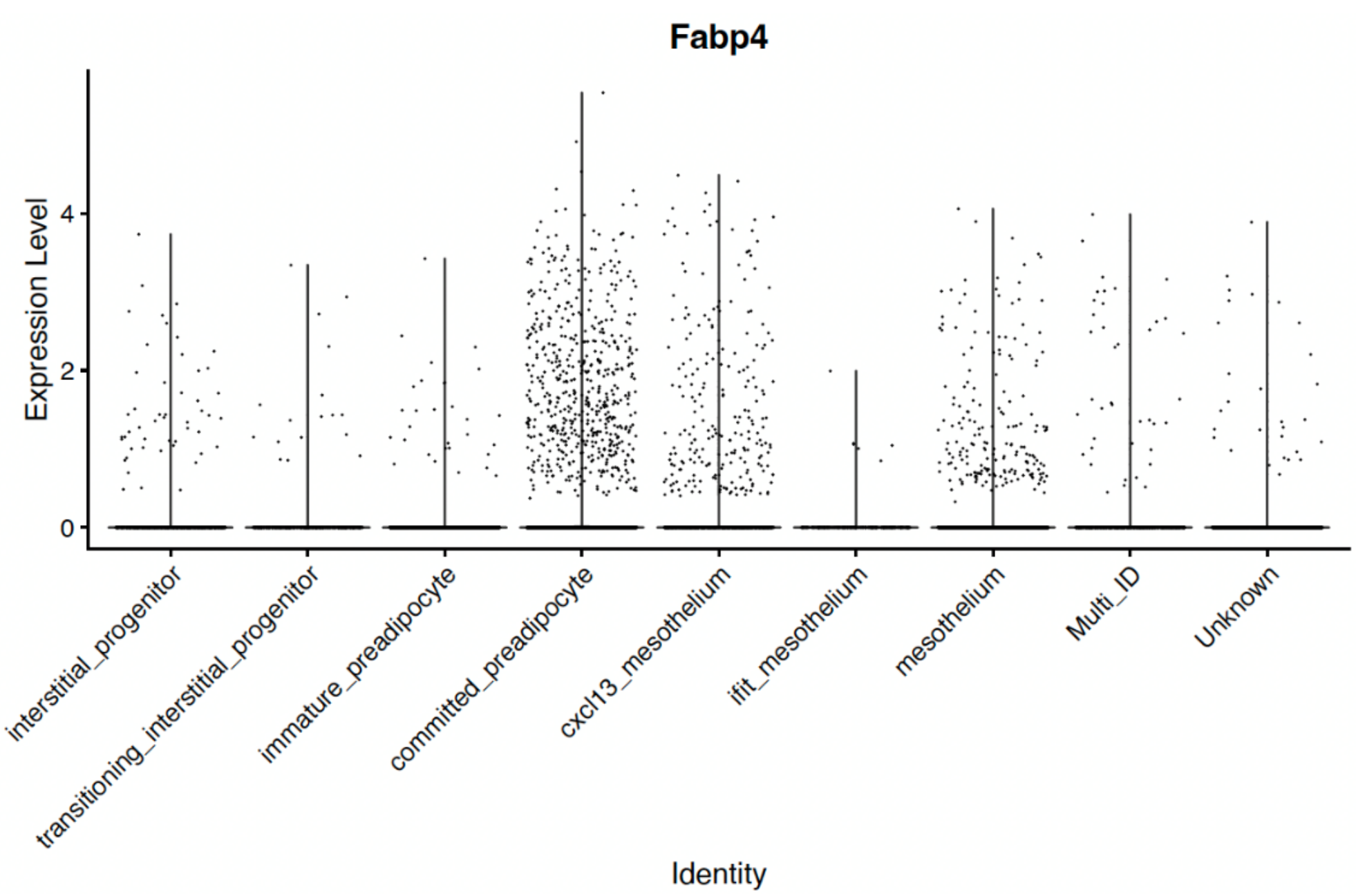

7) Regarding the potential effects of immune cells for the observed phenotype. How does the lack of mature adipocytes alter the polarization of macrophages.

We thank the reviewer for raising the possibility that altered immune cell states, including macrophage polarization, could contribute to the observed phenotype. While our primary objective was to determine whether mature adipocytes are required for omental tumor growth rather than to characterize immune activation states comprehensively, we directly examined macrophage populations to assess this possibility. We note that classical "M1/M2" polarization terminology is increasingly recognized as insufficient for describing tissue-resident peritoneal macrophages, which exhibit niche-imposed transcriptional programs and constitutive

expression of markers such as MRC1 that do not reliably denote activation state. Using single-cell RNA-seq (Fig. 5), we analyzed cavity macrophages, mesothelial-associated macrophages, and monocyte-derived macrophage clusters. We compared the expression of commonly used activation markers between PARC and DARL mice. We did not observe consistent genotype-associated shifts across macrophage subsets that would indicate a broad change in polarization or activation state in the absence of mature adipocytes. Together, these analyses suggest that the phenotypes described here are unlikely to be driven by major alterations in macrophage polarization. However, a more granular dissection of immune-state heterogeneity lies beyond the scope of the present study.

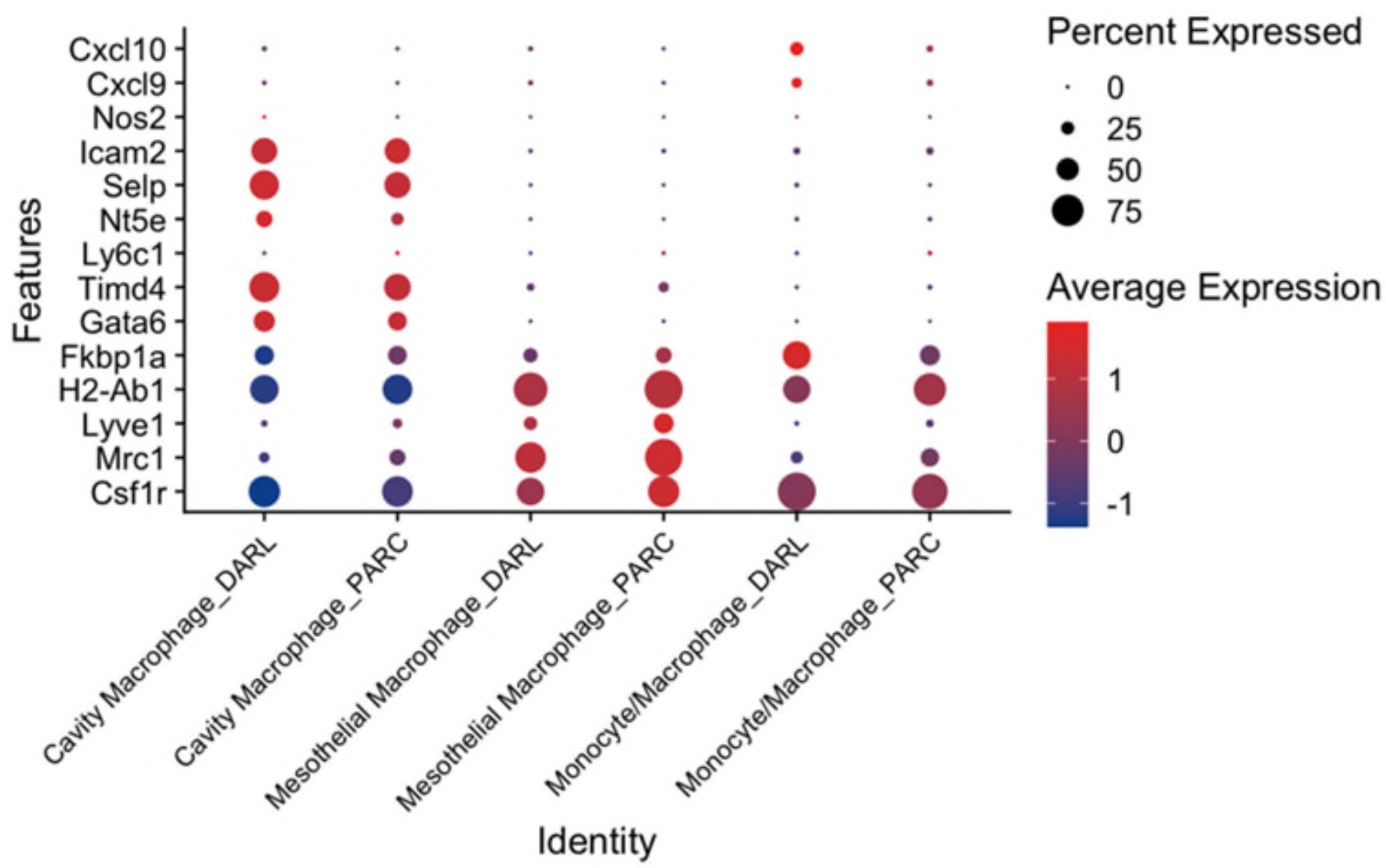

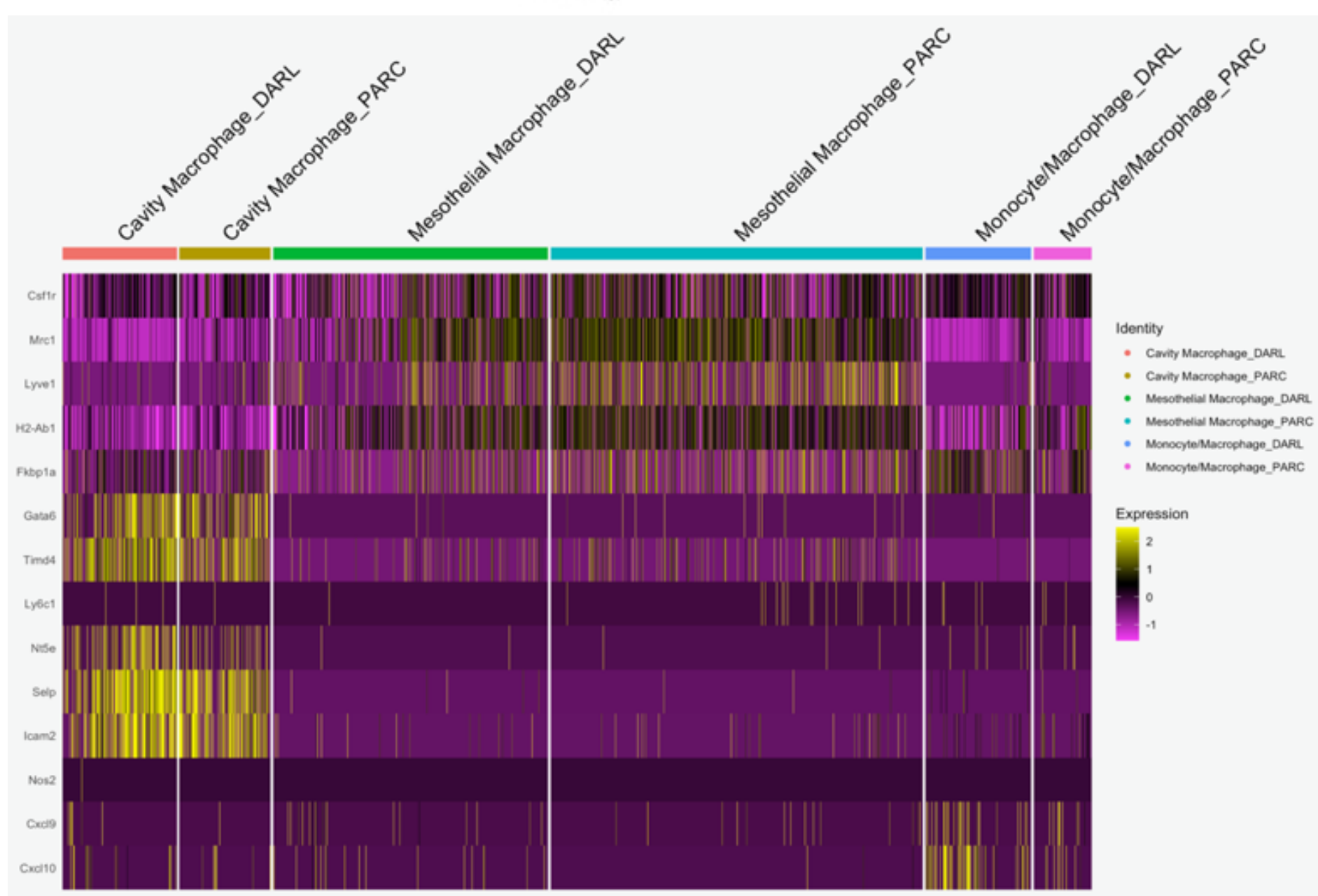

Point-by-point response to the reviewers

Reviewer #1

I appreciate the substantive efforts by the authors in addressing the questions and suggestions of the reviewers, particularly in delineating the role of FABP4 in adipocyte differentiation, and additional histological evaluation as detailed in Figures 1 and 2. The result is a novel, interesting and well-performed study of interest to the readership.

There are, however, still several formatting errors to parse, particularly in referring to figures 6 and 7. I suggest to check these, as these diminish an otherwise well-written manuscript.

I have no additional comments

We thank the reviewer for catching these. We have carefully re-checked every figure and corrected the cross-referencing errors. Specifically, the human single-cell data were incorrectly cited as Fig. 6d–f/6e/6g,h. These now correctly cite the human panels Fig. 6l–n. The steady-state and tumor-associated 3D vascular images were reconciled with the figure legend lettering.

Reviewer #2

I co-reviewed this manuscript with one of the reviewers who provided the listed reports. This is part of the Nature Communications initiative to facilitate training in peer review and to provide appropriate recognition for Early Career Researchers who co-review manuscripts.

We thank the co-reviewer for their time and contribution to the assessment of our work.

Reviewer #3

The authors have done a lot of work to address the original critiques. The manuscript is greatly improved now. In discussion, the paper on the role of endothelial fatty acid transporter CD36 in cancer needs to be cited: doi: 10.1172/jci.insight.147057. Also, needs to be cited a comprehensive recent review on the roles of adipose tissue lipids in carcinoma progression doi: 10.1016/j.trecan.2025.11.012.

We thank the reviewer. Both references have been added to the Discussion and to the reference list.

Reviewer #4

The authors have substantially improved the manuscript. Concerning my previous comment #2. Does the endothelial knockout of FABP4 affect immune cell composition within the abdominal cavity? Since growth of ovarian cancer and metastases are strongly influenced by the immune response, this outstanding question should be answered. The new single cell data shown in Fig. 5 could be potentially be used to answer this question.

We appreciate this point. We would like to clarify the scope of our single-cell dataset: the Fig. 5 scRNA-seq compares adipocyte-replete (PARC) and adipocyte-deficient (DARL) peritoneal tissues, and our human datasets compare cancer versus non-cancer omentum; none of these datasets profile the endothelial-specific FABP4 knockout (Tie2-Cre^{+} Fabp4$^{fl/fl}$) mice, so they cannot directly resolve immune shifts caused by endothelial FABP4 deletion.

Importantly, the central immunological conclusion of the manuscript is that adaptive immunity does not drive omental tumor homing in this system. Tumor burden was unchanged in Rag2/Cd47/Il2rg triple-knockout mice (lacking T, B and NK cells) and after antibody-mediated depletion of CD4, CD8, and NK1.1 cells, in both adipocyte-replete and adipocyte-deficient hosts (Fig. 3e–g). Spectral flow cytometry and scRNA-seq further showed that immune composition was largely preserved after mature adipocyte loss (Fig. 3, Fig. 5). We also did complete flow cytometry of the peritoneal fluid in Tie2-Cre^{+} Fabp4$^{fl/fl}$ and Tie2-Cre- Fabp4$^{fl/fl}$ mice and did not

observe any significant differences in the immune composition. We therefore expect that broad changes in immune composition are unlikely to account for the endothelial-FABP4 phenotype. However, we agree that the interplay between endothelial FABP4 and the local immune compartment is an interesting question for further research.

Concerning comment #3. The authors should add the cholesterol and triglyceride measurement into the manuscript. In addition, they should further explore endothelial Bodipy C16 uptake, at least under basal (non-tumor) conditions.

These measurements are already included. Serum triglycerides (normalized after fat transplantation; Supplementary Fig. 2e) and total serum cholesterol (elevated in lipoatrophic mice, below hypercholesterolemic ranges, reduced toward control after transplantation; Supplementary Fig. 2f) are reported in the Results and Methods. We have verified that both are described in the text and shown in the figure.

In addition, they should further explore endothelial Bodipy C16 uptake, at least under basal (non-tumor) conditions.

We performed BODIPY-C16 uptake experiments under basal (non-tumor) conditions. However, at steady state, the omental endothelial population is very small, and the number of endothelial cells recovered was insufficient to reliably quantify uptake or detect shifts between genotypes using our flow-cytometry readout. Thus, we did not pursue this data further.
